# Indium-Mediated Preparation of Bis(α-hydroxyallenes) or α,α′-Dihydroxyallenynes and Further Gold-Catalyzed Cyclizations

**DOI:** 10.1021/acs.joc.4c01648

**Published:** 2024-09-17

**Authors:** Teresa Martínez del Campo, Daniel San Martín, Laura Gamarra, Eva Cerrón, Sara Cembellín, Hikaru Yanai, Pedro Almendros

**Affiliations:** † Grupo de Lactamas y Heterociclos Bioactivos, Departamento de Química Orgánica, Unidad Asociada al CSIC, Facultad de Química, Universidad Complutense de Madrid, Madrid 28040, Spain; § School of Pharmacy, Tokyo University of Pharmacy and Life Sciences, 1432-1 Horinouchi, Hachioji ,Tokyo192-0392, Japan; ‡ Instituto de Química Orgánica General, IQOG, CSIC, Juan de la Cierva 3, Madrid 28006, Spain

## Abstract

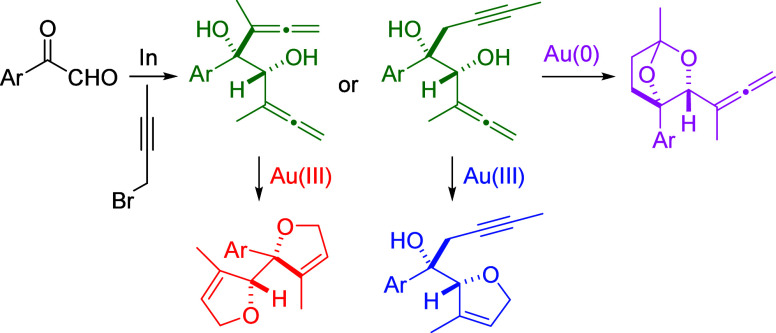

We present the regio- and diastereoselective Barbier-type allenylation reaction of glyoxals mediated by indium to furnish highly valuable *syn*-bis(α-hydroxyallenes) and *syn*-α,α′-dihydroxyallenynes. The gold-catalyzed controlled cyclization of these unsaturated diols enables the divergent preparation of three types of oxacycles.

## Introduction

**Scheme 1:**
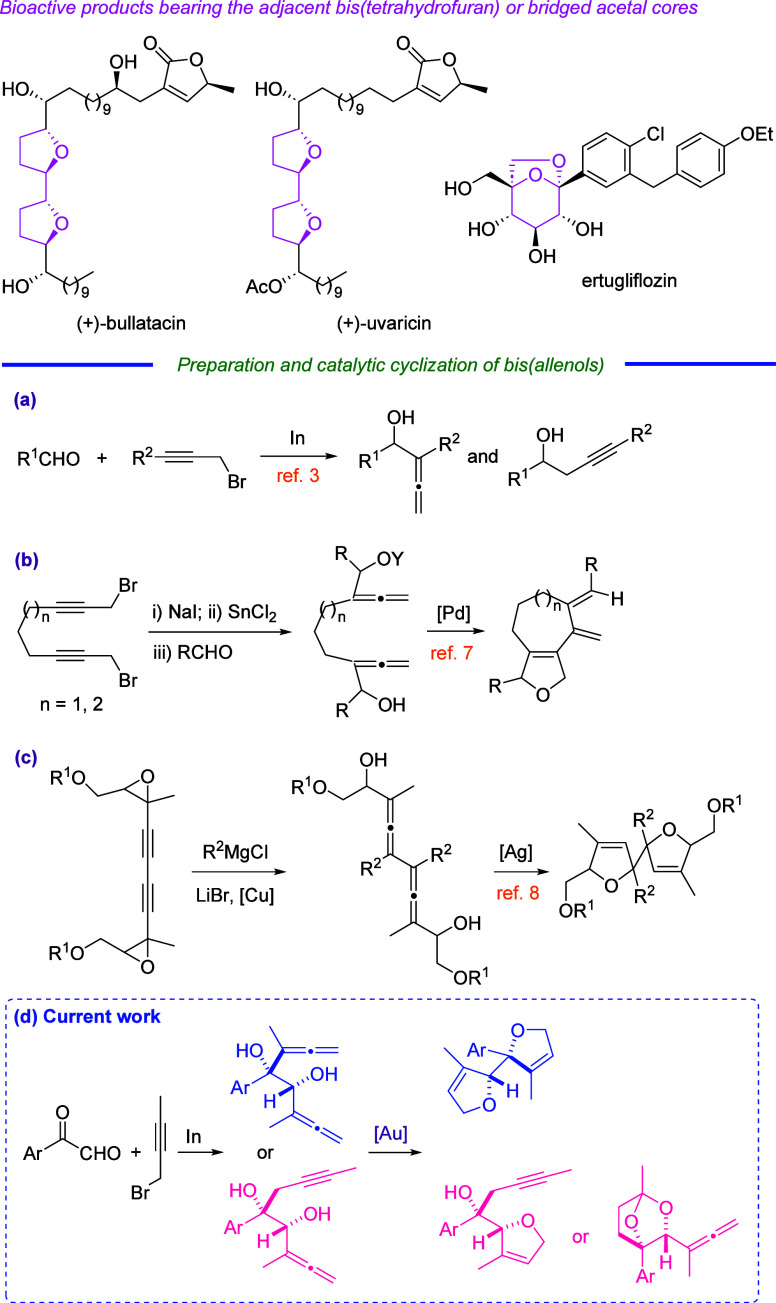
(a–d) Background and Synopsis of the Current Study

Dihydrofuran and tetrahydrofuran derivatives are key scaffolds of widespread existence in many bioactive natural products such as bullatacin and uvaricin, which displayed the adjacent bis(tetrahydrofuran) core (Scheme 1, top). Bridged acetal motifs are also ubiquitous in drugs such as ertugliflozin (Scheme 1, top). Allenols are a particular class of allenes, which display a great synthetic utility.^1,2^ The Barbier allenylation of carbonyl compounds has been revealed as an attractive protocol for the preparation of allenols, albeit the propargyl/allenyl metallotropic rearrangement that makes it difficult to control the regioselectivity, usually resulting in mixtures of allenols and homopropargylic alcohols (Scheme 1a).^3^ Despite considerable advances, no reports have discussed the use of glyoxals as carbonyl reagents for the preparation of challenging bis(α-allenols) through allenylation reactions until today.^4^ Gold catalysis has been widely employed for the conversion of α-allenols into functionalized molecules.^5,6^ By contrast, the metal-catalyzed cyclization of bis(allenols) has received less attention. Notable exceptions are the palladium-catalyzed cyclization of 1,ω-bisallenols to afford 2,5-dihydrofuran-fused bicyclic skeletons described by Ma et al. (Scheme 1b)^7^ and the silver-catalyzed cycloisomerization of bis(α-allenols) with unassigned relative configuration to provide adjacent bis(dihydrofurans) described by Poonoth and Krause (Scheme 1c).^8^ Herein, we report the indium-mediated Barbier-type allenylation reaction of arylglyoxals to synthesize in a regiocontrolled and diastereoselective manner in both *syn*-bis(α-hydroxyallenes) and *syn*-α,α′-dihydroxyallenynes together with its applicability to gold-catalyzed cyclization reactions (Scheme 1d).

## Results and Discussion

**Scheme 2:**
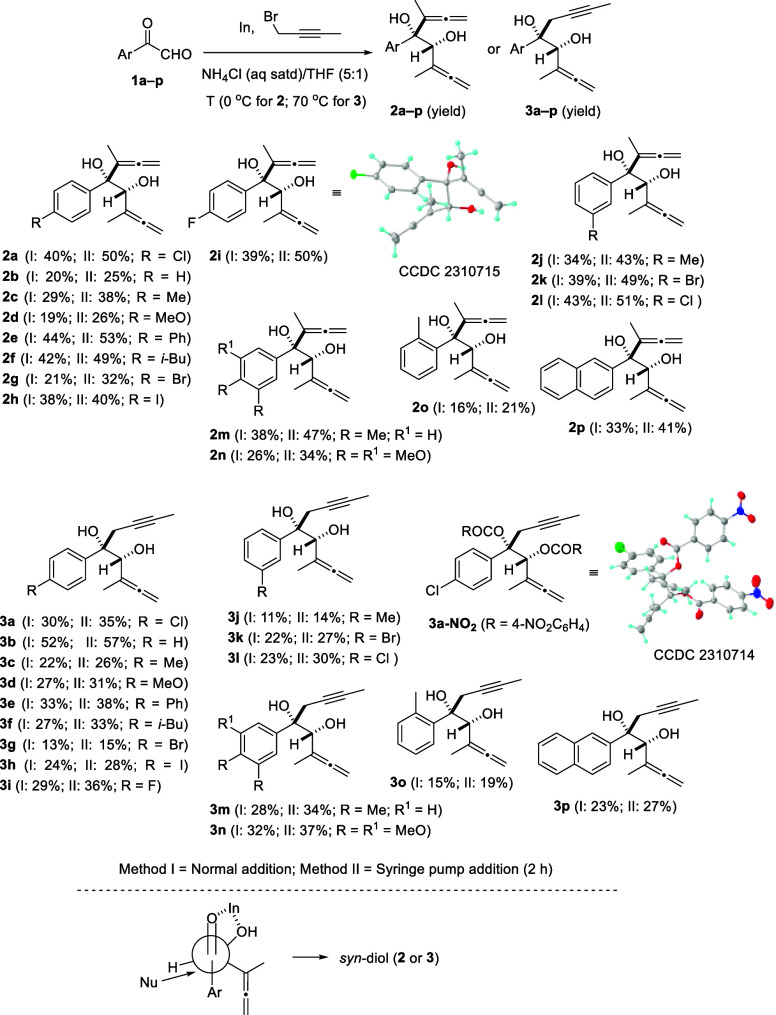
Indium-Mediated Controlled Preparation of Bis(α-hydroxyallenes) **2a**–**p** and α,α′-Dihydroxyallenynes **3a**–**p** Yield of a pure, isolated product with correct analytical and spectral data.

We initiated our carbonyl allenylation studies by using arylglyoxal **1a** as a model substrate. The screening results of Barbier-type coupling are sketched in Table 1. Notably, despite the formation of up to 12 different products (four monofunctionalizated regioisomers and eight regioisomeric bifunctionalization adducts, including *syn*- or *anti*-diastereomers) being theoretically generated, the indium-mediated reaction between arylglyoxal **1a** and 1-bromobut-2-yne just resulted in the formation of *syn*-bis(α-allenol) **2a** as major component (entry 9, Table 1) or *syn*-α,α′-dihydroxyallenyne **3a** as an exclusive product (entry 11, Table 1). Nicely, the slow addition of 1-bromobut-2-yne using a syringe pump resulted in higher yields of diols **2a** and **3a** (entries 10 and 12, Table 1). The use of propargyl bromide instead of 1-bromobut-2-yne resulted in complicated mixtures. Indeed, the indium-promoted coupling of arylglyoxal **1a** with propargyl bromide was messy and monopropargylated 1-(4-chlorophenyl)-2-hydroxypent-4-yn-1-one was isolated as the major component in a pyrrhic 8% yield (see the Supporting Information). The indium-mediated Barbier-type coupling was superior to the use of different zerovalent metals (Zn and Sn). The solvent of choice was H_2_O/THF (5:1) with the addition of NH_4_Cl, while different solvents such as H_2_O/THF (1:1) or H_2_O/methanol (5:1) with or without additives (LiCl and HfCl_4_) provided the worse outcome. The temperature proved to be essential for directing the reaction toward the formation of diol **2a** (*T* = 0 °C) or diol **3a** (*T* = 70 °C). The [TiClCp_2_]-assisted reaction^9^ between arylglioxal **1a** and 1-bromobut-2-yne was not productive because the main product was 1-(4-chlorophenyl)ethan-1-one (see Table S1, Supporting Information). Despite the fact that the yields are moderate, the allenylation of arylglyoxals **1** under Barbier conditions was totally diastereoselective and highly regioselective (Scheme 2, top). Arylglyoxals **1** bearing a variety of substituents (Me, *i*-Bu, Ph, MeO, Br, Cl, I, and F) at different positions (*ortho*, *meta*, and *para*) on the aryl rings were tolerated. When the reaction did not proceed with complete regioselectivity, isomeric *syn*-bis(α-hydroxyallenes) **2** and *syn*-α,α′-dihydroxyallenynes **3** were easily isolated by column chromatography. The structures of bis(α-hydroxyallene) **2i** and the 4-nitrobenzoate of α,α′-dihydroxyallenyne **3a** were assigned through X-ray crystallography. The diastereoselectivity of the indium-mediated addition of 1-bromobut-2-yne to arylglyoxals to give *syn*-diols **2** and **3** can be rationalized in terms of the Cram chelation model for attacking the ketone site after the first allenylation at the aldehyde moiety (Scheme 2, bottom).

**Table 1:**
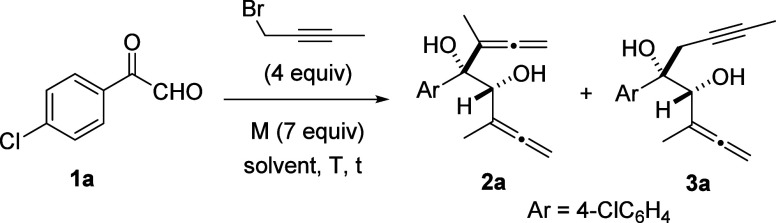
Synthesis of *syn*-Bis(α-hydroxyallene) **2a** and *syn*-α,α′-Dihydroxyallenyne **3a** under Modified Barbier-Type Conditions

entry	M	conditions^a^	yield **2a**/**3a** (%)^b^
1	In	method I, H_2_O/methanol (1:1), rt, 24 h	
2	In	method I, H_2_O/THF (1:1), rt, 24 h	27/16
3	In	method I, H_2_O/THF (5:1), LiCl, rt, 24 h	25/14
4	In	method I, H_2_O/THF (5:1), HfCl_4_, rt, 24 h	19/23
5	In (3 equiv)	method I, H_2_O/THF (5:1), NH_4_Cl, rt, 24 h	22/16^c^
6	Sn	method I, H_2_O/THF (5:1), NH_4_Cl, rt, 24 h	
7	Zn	method I, H_2_O/THF (5:1), NH_4_Cl, rt, 24 h	7/13
8	In	method I, H_2_O/THF (5:1), NH_4_Cl, rt, 24 h	32/25
9	In	method I, H_2_O/THF (5:1), NH_4_Cl, 0 °C, 30 h	40/12
10	In	method II, H_2_O/THF (5:1), NH_4_Cl, 0 °C, 30 h	50/14
11	In	method I, H_2_O/THF (5:1), NH_4_Cl, 70 °C, 6 h	0/30
12	In	method II, H_2_O/THF (5:1), NH_4_Cl, 70 °C, 6 h	0/35

a Method I = normal addition. Method II = syringe pump addition (2 h).

b Yield of a pure, isolated product with correct analytical and spectral data.

c 1.5 equiv of 1-bromobut-2-yne was used. The monoallenol was formed in a 12% yield.

**Scheme 3:**
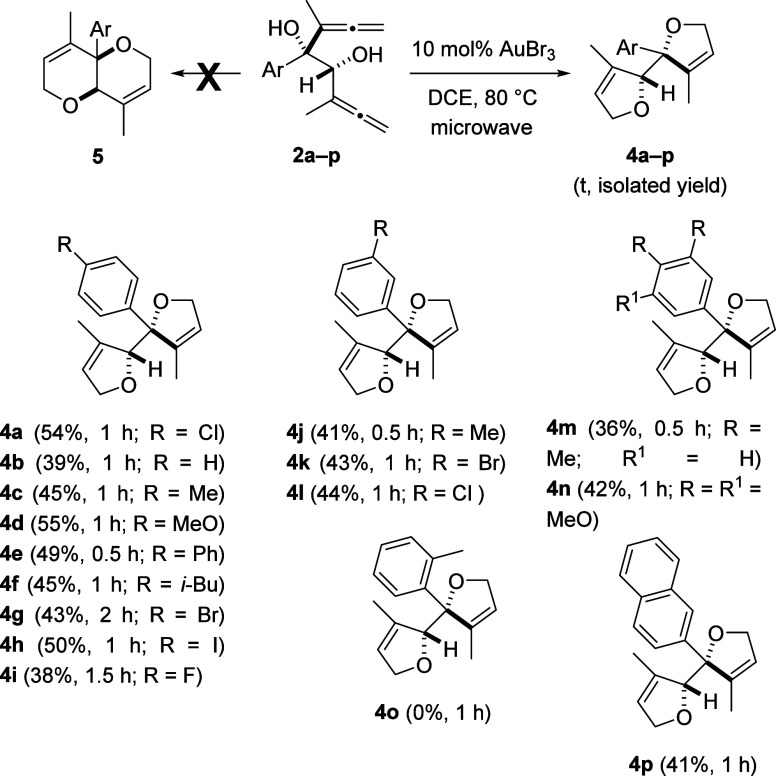
Gold-Catalyzed Synthesis of Bis(dihydrofurans) **4a**–**p** Yield of a pure, isolated product with correct analytical and spectral data.

At first, we attempted the gold-catalyzed cycloisomerization reaction of bis(α-hydroxyallene) **2a** employing different Au(I)-based precatalysts (see Table S2, Supporting Information), but complex reaction mixtures were detected. Next, we replaced the soft Lewis acid gold(I) complexes with a hard gold(III) salt. Fortunately, the use of AuCl_3_ provided the adjacent bis(dihydrofuran) **4a** in 20% yield. We obtained the target product in an isolated yield of around 40% with both (Pic)AuCl_2_ and AuBr_3_ in DCE at 80 °C in a microwave reactor (Scheme 3). Improved yields of 52 and 54% were achieved by performing the reaction at 0 °C or at a higher concentration (0.1 M), respectively (see Table S2, Supporting Information). Despite the fact that complete conversion was observed (TLC and ^1^H NMR) in the crude reaction mixture of bis(allene) **2a**, and no side products were detected, efforts to improve the yield of bis(heterocycle) **4a** were in vain. Consequently, we began to explore the substrate scope of the above 2-fold cyclization (Scheme 3). The electronic nature of the substituent attached to the aromatic ring does not affect the cycloisomerization, and the desired bis(dihydrofurans) **4** were isolated in similar yields bearing both electron-donating (MeO, Me, and *i*-Bu) or electron-withdrawing (F, Cl, and Br) moieties. 4-Biphenyl- and 2-naphthyl-substituted bis(oxacycles) **4e** and **4p** were also forged in a related fashion. Substitution in the *para*- and *meta*-positions was tolerated and resulted in adducts **4a**–**m** and **4p**. By contrast, *ortho*-substituted product **4o** was not formed, pointing to a great influence due to steric hindrance. It should be noted that the Au(III)-catalyzed cycloisomerization of bis(hydroxyallenes) **2** to build bis(dihydrofurans) **4** is totally chemoselective and proceeded through a 2-fold 5-*endo*-cyclization. The formation of isomeric tetrahydropyrano[3,2-*b*]pyrans **5** via double 6-*endo*-cyclization was not detected.

**Scheme 4:**
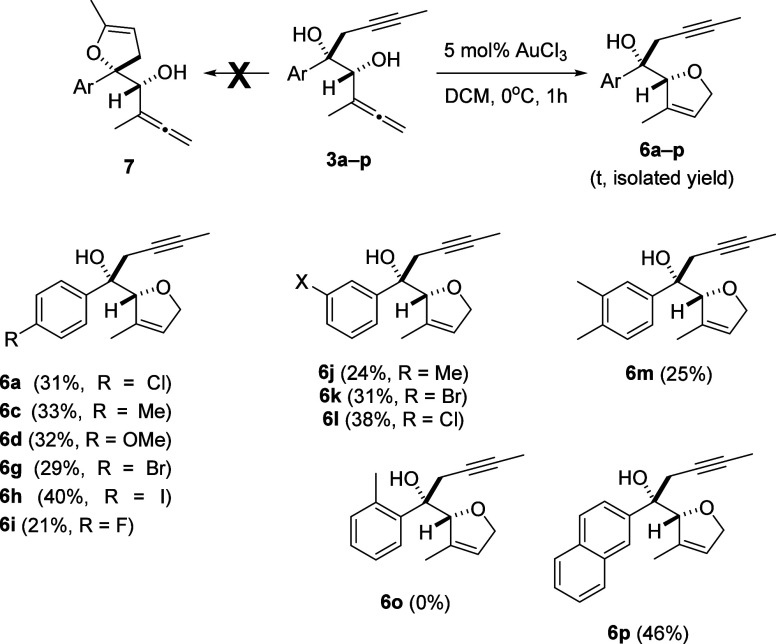
Gold-Catalyzed Synthesis of Alkynol-Tethered Dihydrofurans **6a**–**p** Yield of a pure, isolated product with correct analytical and spectral data.

Subsequently, the gold-catalyzed reactivity of dihydroxyallenynes **3** was evaluated. The reaction of diol **3a** with AuCl_3_ under catalytic conditions resulted in controlled formation of functionalized dihydrofuran **6a** by exclusive cyclization of the allenol moiety, while isomeric dihydrofuran **7a** arising from the cycloisomerization of the alkynol moiety was not detected. The best result was obtained by mixing the reagents in DCM (0.1 M) at 0 °C (see Table S3, Supporting Information). Various acyclic precursors **3** also underwent this selective monocyclization toward the allene functionality to provide functionalized dihydrofurans **6** in low to moderate yields (Scheme 4).

**Scheme 5:**
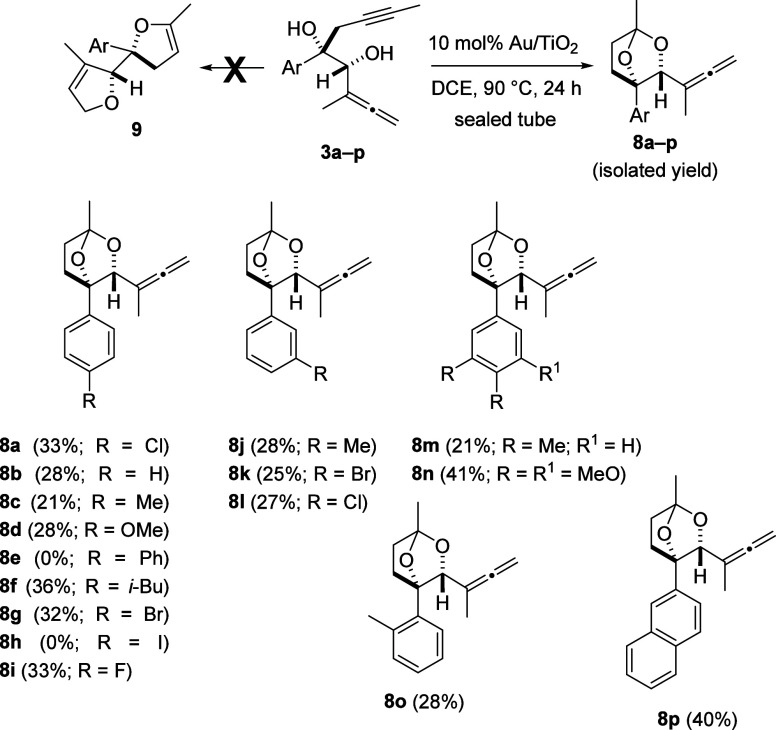
Gold-Catalyzed Synthesis of Bridged Ketals **8a**–**p** under Heterogeneous Conditions Yield of a pure, isolated product with correct analytical and spectral data.

The alkyne oxycyclization stage was not feasible under Au(III) catalysis. Noteworthy, the electrophilic character of the alkyne and allene moieties in α,α′-dihydroxyallenynes **3** was modulated by the effect of a Au nanoparticle (NP)-based catalyst by granting a selective cycloketalization toward the alkyne moiety, keeping unaltered the allene group. The lack of 2-fold cycloetherification toward the formation of adducts **9** was observed, while the only productive way under heterogeneous conditions (Au/TiO_2_)^10^ was the geminal bis(oxycyclization) path to provide bridged ketals **8**. In this way, acyclic precursors **3** smoothly provided the desired 1,3,4-trisubstituted-2,7-dioxabicyclo[2.2.1]heptanes **8** (Scheme 5).^11^ The cycloketalization reaction was very sensitive to steric hindrance, and the presence of bulky substituents at the arene ring such as 4-phenyl or 4-iodo moieties in α,α′-dihydroxyallenynes **3e** and **3h** was detrimental. The AuNP treatment of **3e** and **3h** resulted in the recovery of the starting material, while forcing the reaction conditions (120 °C) resulted in a complex mixture. Nicely, a 2-naphthyl group was tolerated, and bridged ketal **8p** was smoothly achieved under the standard conditions (Scheme 5).

**Scheme 6:**
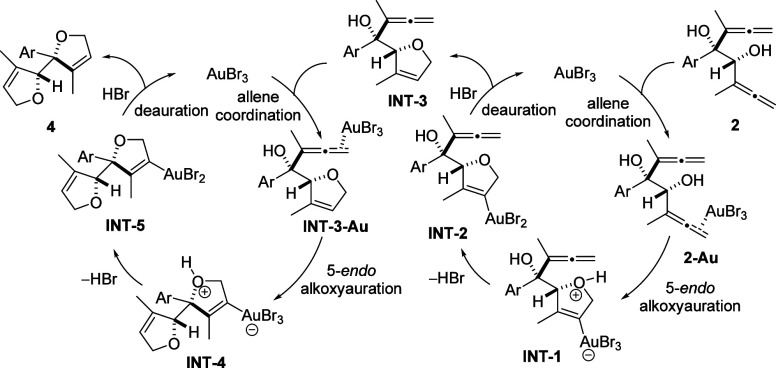
Proposed Mechanism for the Formation of Bicycles **4**

A possible pathway for the formation of adjacent bis(dihydrofurans) **4** from *syn*-bis(α-allenols) **2**, which should involve two gold-based catalytic cycles, is outlined in Scheme 6. Initial coordination of the gold salt with the distal double bond of the allene moiety should lead to complexes **2-Au** and **INT-3-Au**, which after 5-*endo* cycloetherification should build zwitterionic intermediates **INT-1** and **INT-4**. Next, HBr release should form neutral alkenylgold species **INT-2** and **INT-5**. Successive breakage of the gold–carbon bond in **INT-2** and **INT-5** by protonolysis assisted by HBr should result in intermediate **INT-3** and final product **4** with concomitant regeneration of the gold catalyst.

**Scheme 7:**
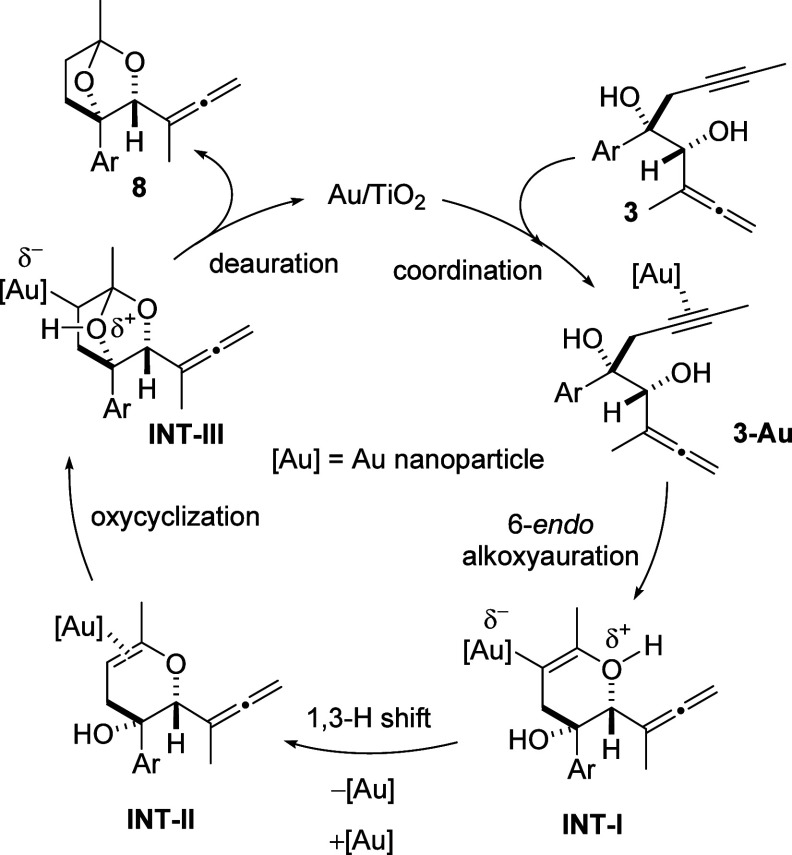
Plausible Pathway for the Formation of Bridged Acetals **8**

In Scheme 7, we propose a plausible mechanistic picture for the formation of bridged acetals **8**. The alkynophilicity of gold initiates the preferred coordination of the triple bond in α,α′-dihydroxyallenynes **3** to the gold(0) nanoparticle [Au], leading to gold complex **3-Au**. Subsequently, a 6-*endo* alkoxyauration occurs by the nucleophilic attack of the hydroxyl moiety, giving rise to dihydropyranol intermediate **INT-I**. In consonance with organogold nanoclusters,^12^ we propose a partial negative charge for [Au] in **INT-I**. Although merely speculative at this time, the preferential activation of the alkyne moiety over the allene functionality in α,α′-dihydroxyallenynes **3** may be ascribed to the stabilization of the partially polarized zwitterion-type intermediate **INT-I** imparted by the polar support (TiO_2_) of the gold nanocatalyst. Further deprotonation, deauration, and complexation resulted in complex **INT-II**, which elicits a second cycloetherification to generate species **INT-III**. Loss of protons linked to deauration provides acetals **8** with concurrent regeneration of the gold catalyst.

## Conclusions

To sum up, we demonstrated that the challenging Barbier-type allenylation reaction of glyoxals proceeds smoothly using indium as a promoter. More importantly, the so-obtained products, namely, *syn*-bis(α-hydroxyallenes) and *syn*-α,α′-dihydroxyallenynes, suffered from controlled cyclization under gold catalysis to enable the divergent preparation of three types of oxacycles in a totally selective manner.

## Data Availability

The data underlying this study are available in the published article and its Supporting Information.

## Supporting Information Available

The Supporting Information is available free of charge at https://pubs.acs.org.doi/10.1021/acs.orglett.
Experimental procedures, characterization data of new compounds, copies of NMR spectra, and crystallographic data (PDF)
