# Supplementary material for: Indium-Mediated Preparation of Bis(α-hydroxyallenes) or α,α′-Dihydroxyallenynes and Further Gold-Catalyzed Cyclizations
Source: J Org Chem. 2024 Sep 17;89(19):14228–32. doi: 10.1021/acs.joc.4c01648 (PMC11460725; doi:10.1021/acs.joc.4c01648)

Supplementary Information for the paper

Indium-Mediated Preparation of Bis(α-hydroxyallenes) or α,α'-Dihydroxyallenynes and further Gold-Catalyzed Cyclizations

Teresa Martínez del Campo,*,† Daniel San Martín,† Laura Gamarra,† Eva Cerrón,† Sara Cembellín,† Hikaru Yanai,§ and Pedro Almendros*,‡

†Grupo de Lactamas y Heterociclos Bioactivos, Departamento de Química Orgánica, Unidad Asociada al CSIC, Facultad de Química, Universidad Complutense de Madrid, 28040-Madrid, Spain

§School of Pharmacy, Tokyo University of Pharmacy and Life Sciences, 1432-1 Horinouchi, Hachioji, Tokyo 192-0392, Japan

‡Instituto de Química Orgánica General, IQOG-CSIC, Juan de la Cierva 3, 28006-Madrid, Spain

E-mail: teremart@ucm.es; palmendros@iqog.csic.es

Table of Contents

General Methods	S1
Table S1	S2
Experimental Section	S3–S45
Crystallographic Data	S46–S57
^{1}H NMR, and ^{13}C NMR Spectra	S58–S131

General Methods: ^{1}H NMR and ^{13}C NMR spectra were recorded on a Bruker Avance AVIII-700 with cryoprobe, Bruker AMX-500, Bruker Avance-300, or Varian VRX-300S. NMR spectra were recorded in $CDCl_3$ solutions, except otherwise stated. Chemical shifts are given in ppm relative to TMS (^{1}H, 0.0 ppm), or $CDCl_3$ (^{1}H, 7.27 ppm; ^{13}C, 76.9 ppm), or acetone-d_6 (^{1}H, 2.05 ppm; ^{13}C, 206.3 ppm), or C_6D_6 (^{1}H, 7.16 ppm; ^{13}C, 128.0 ppm), or CD_3CN (^{1}H, 1.94 ppm; ^{13}C, 118.2 ppm), or DMSO-d_6 (^{1}H, 2.50 ppm; ^{13}C, 39.5 ppm). Low and high resolution mass spectra were taken on an AGILENT 6520 Accurate-Mass QTOF LC/MS spectrometer using the electronic impact (EI) or electrospray modes (ES) unless otherwise stated. IR spectra were recorded on a Bruker Tensor 27 spectrometer. All commercially available compounds were used without further purification. For reactions that require heating, a heat-on block or a microwave reactor were used. Microwave irradiation was carried out in a Monowave 300 from Anton Paar GmbH. Reactions in a microwave reactor are all performed in a sealed condition of a closed vessel system. The reaction temperatures during microwave heating were measured with an internal infrared sensor. Column chromatography was carried out using silica gel 60, 0.04-0.06 mm, for flash chromatography (230-

400 mesh ASTM) provided by Scharlau. For reactions that require heating, a heating-on block was used. All commercially available compounds were used without further purification.

Table S1. Synthesis of *syn*-bis(α-hydroxyallene) **2a** and *syn*-α,α'-dihydroxyallenyne **3a** under modified Barbier-type conditions

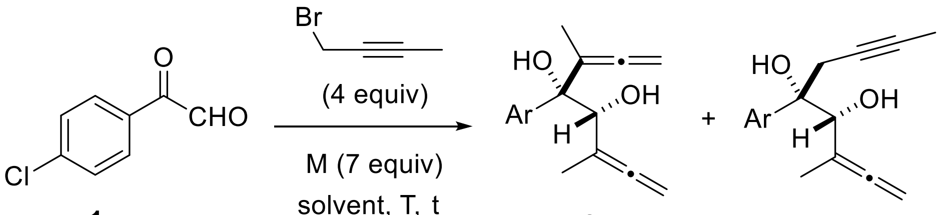

1a **2a** **3a**

Ar = 4-ClC_6H_4

entry	M	Conditions[a]	yield **2a**/**3a** (%)[b]
1	In	method I, H_2O/methanol (1:1), rt, 24 h	-----
2	In	method I, H_2O/THF (1:1), rt, 24 h	27/16
3	In	method I, H_2O/THF (5:1), LiCl, rt, 24 h	25/14
4	In	method I, H_2O/THF (5:1), $HfCl_4$, rt, 24 h	19/23
5	In (3 equiv)	method I, H_2O/THF (5:1), NH_4Cl, rt, 24 h	22/16[c]
6	Sn	method I, H_2O/THF (5:1), NH_4Cl, rt, 24 h	-----
7	Zn	method I, H_2O/THF (5:1), NH_4Cl, rt, 24 h	7/13
8	In	method I, H_2O/THF (5:1), NH_4Cl, rt, 24 h	32/25
9	In	method I, H_2O/THF (5:1), NH_4Cl, 0 °C, 30 h	40/12
10	In	method II, H_2O/THF (5:1), NH_4Cl, 0 °C, 30 h	50/14
11	In	method I, H_2O/THF (5:1), NH_4Cl, 70 °C, 6 h	0/30
12	In	method II, H_2O/THF (5:1), NH_4Cl, 70 °C, 6 h	0/35
13	In	method I, H_2O/THF (5:1), NH_4Cl, 0 °C, 30 h	35/9[d]
14	In	method I, H_2O/THF (5:1), NH_4Cl, 0 °C, 30 h	40/12[e]
15	In (5 equiv)	method I, H_2O/THF (5:1), NH_4Cl, 0 °C, 30 h	37/11
16	In	method I, H_2O/DMF (1:1), rt, 24 h	-----
17	In	method I, H_2O/1,4-dioxane (1:1), rt, 24 h	-----
18	[$TiCl_2Cp_2$]/Mn	THF, Me_3SiCl, 2,4,6-collidine, rt, 6 h	-----[f]

[a]Method I = Normal addition. Method II = Syringe pump addition (2 h). [b]Yield of pure, isolated product with correct analytical and spectral data. [c]1.5 equivalents of 1-bromobut-2-yne were used. The monoallenol was formed in a 12% yield. [d]3 equivalents of 1-bromobut-2-yne were used. [e]5 equivalents of 1-bromobut-2-yne were used. [f]1-(4-Chlorophenyl)ethan-1-one was formed in a 12% yield.

Experimental Section

Arylglyoxals 1 were prepared as hydrate derivatives according to the method reported by Moffett (Moffett, R. B.; Tiffany, B. D.; Aspergren, B. D.: Heinzelman, R. V. Antiviral Compounds. II. Aromatic Glyoxals. *J. Am. Chem. Soc.* **1957**, *79*, 1687–1690; Eftekhari-Sis, B.; Zirak, M.; Akbari, A. Arylglyoxals in Synthesis of Heterocyclic Compounds. *Chem. Rev.* **2013**, *113*, 2958–3043; Javahershenas, R.; Khalafy, J.; Prager, R. H. Application of Arylglyoxals in Synthesis of Pyrrolo[2,3-*d*] pyrimidines via Multicomponent Reactions. *J. Chem. Rev.* **2019**, *1*, 233–242); **using the following procedure:** A stirred mixture of selenium dioxide (267 mg, 1.5 mmol) in 1,4-dioxane/water (10:1) (33 mL) was heated at 90 ºC until complete dissolution of the solid. Next, the appropriate acetophenone (1 mmol) was added and the resulting mixture was stirred at reflux temperature for 24 h. The mixture was allowed to cool to rt before the formed Se(0) and the excess of SeO_2 were removed by filtration through a celite pad. The filtrate was concentrated under reduced pressure to provide the arylglyoxals, which were not purified and were used as crude products for the indium-mediated coupling step.

General procedure for the synthesis of *syn*-bis(α-hydroxyallenes) 2 and *syn*-α,α'-dihydroxyallenynes 3.

Method I: 1-Bromo-2-butyne (532 mg, 4.0 mmol) was added to a stirred suspension of indium powder (804 mg, 7.0 mmol) and the corresponding 2-oxo-2-arylacetaldehyde (1.0 mmol) in THF/NH_4Cl (aq., sat.) (1:5) (5 mL). The reaction was stirred at the appropriate temperature (rt for diols **2** and 70 ºC for diols **3**) until disappearance of the starting material (TLC), and the crude was extracted with ethyl acetate (3 x 5mL). The organic extracts were dried ($MgSO_4$) and concentrated under reduced pressure. Purification of the residue by column chromatography using ethyl acetate/hexanes mixtures, afforded pure compounds. Spectroscopic and analytical data for compounds **2a–p** and **3a–p** follow.

Method II: A solution of 1-bromo-2-butyne (532 mg, 4.0 mmol) in THF (4 mL) was added (over a period of 2 h using a syringe pump) to a stirred suspension of indium powder (804 mg, 7.0

mmol) and the corresponding 2-oxo-2-arylacetaldehyde (1.0 mmol) in THF/NH_4Cl (aq., sat.) (1:5) (1 mL). The reaction was stirred at the appropriate temperature (rt for diols **2** and 70 °C for diols **3**) until disappearance of the starting material (TLC), and the crude was extracted with ethyl acetate (3 x 5mL). The organic extracts were dried ($MgSO_4$) and concentrated under reduced pressure. Purification of the residue by column chromatography using ethyl acetate/hexanes mixtures, afforded pure compounds **2** and **3**.

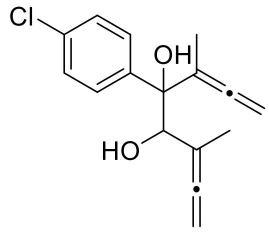

2a

Diol 2a. From 110 mg (0.59 mmol) of 2-oxo-2-arylacetaldehyde **1a**, and after column chromatography using ethyl acetate/hexanes (1:30) as eluent gave compound **2a** (Method I: 64 mg, 40%; Method II: 80 mg, 50%) as colorless solid; m.p. 59–61 ºC; ^{1}H NMR (300 MHz, $CDCl_3$, 25 ºC): δ 7.35–7.27 (m, 2H), 7.21 (dt, 2H, *J* = 8.1, 1.9 Hz), 4.98–4.81 (m, 2H), 4.57 (s, 1H), 4.51–4.40 (m, 1H), 4.31–4.22 (m, 1H), 3.07 (s, 1H), 2.37 (s, 1H), 1.47 (t, 3H, *J* = 3.1 Hz), 1.33 (t, 3H, *J* = 3.2 Hz); ^{13}C{1H} NMR (75 MHz, $CDCl_3$, 25 ºC): δ 207.4, 204.1, 139.8, 132.5, 127.8 (2C), 127.4 (2C), 103.1, 97.6, 80.1, 77.0, 76.2, 74.9, 15.3, 14.4; IR ($CHCl_3$) : ν 1014, 1954, 3460 cm^{-1}; HRMS (ESI) *m/z*: $[M+H]^+$ calcd for $C_{16}H_{18}ClO_2$: 277.0990; found 277.0971.

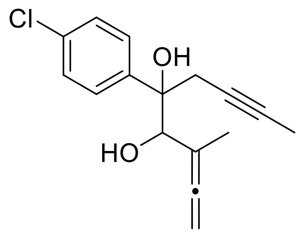

3a

Diol 3a. From 110 mg (0.59 mmol) of 2-oxo-2-arylacetaldehyde **1a**, and after column chromatography using ethyl acetate/hexanes (1:30) as eluent gave compound **3a** (Method I: 49

mg, 30%; Method II: 57 mg, 35%) as colorless solid; m.p. 64–65 ºC; ^{1}H NMR (300 MHz, $CDCl_3$, 25 ºC): δ 7.40–7.35 (m, 2H), 7.33–7.27 (m, 2H), 4.60–4.43 (m, 2H), 4.33 (s, 1H), 2.98–2.73 (m, 2H), 2.79 (m, 1H), 2.50 (d, 1H, J = 17.2 Hz), 1.72 (t, 3H, J = 2.6 Hz), 1.45 (t, 3H, J = 3.2 Hz); ^{13}C{1H} NMR (75 MHz, $CDCl_3$, 25 ºC): δ 206.4, 140.7, 132.8, 127.7 (2C), 127.1 (2C), 98.5, 86.1, 80.0, 77.8, 75.8, 73.7, 30.6, 15.3, 3.2; IR ($CHCl_3$): ν 1091, 1957, 3507 cm^{-1}; HRMS (ESI) *m/z*: $[M+Na]^+$ calcd for $C_{16}H_{17}NaClO_2$: 299.0809; found 299.0817.

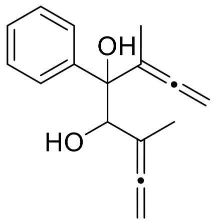

2b

Diol 2b. From 200 mg (1.31 mmol) of 2-oxo-2-arylacetaldehyde **1b**, and after column chromatography using ethyl acetate/hexanes (1:15) as eluent gave compound **2b** (Method I: 64 mg, 20%; Method II: 80 mg, 25%) as colorless oil; ^{1}H NMR (300 MHz, $CDCl_3$, 25 ºC): δ 7.39 (m, 2H), 7.24 (m, 3H), 5.00–4.86 (m, 2H), 4.60 (d, 1H, J = 2.6 Hz), 3.05 (s, 1H), 4.52–4.23 (m, 2H), 2.40 (d, 1H, J = 3.9 Hz), 1.51 (q, 3H, J = 2.9 Hz), 1.31 (t, 3H, J = 3.2 Hz); ^{13}C{1H} NMR (75 MHz, $CDCl_3$, 25 ºC): δ 207.8, 204.5, 141.3, 127.7 (2C), 127.1, 125.9 (2C), 104.2, 98.1, 80.9, 76.8, 76.4, 74.9, 15.5, 14.5; IR ($CHCl_3$) : ν 1016, 1957, 3462 cm^{-1}; HRMS (ESI) *m/z*: $[M+H]^+$ calcd for $C_{16}H_{19}O_2$: 243.1380; found 243.1372.

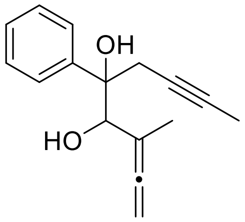

3b

Diol 3b. From 200 mg (1.31 mmol) of 2-oxo-2-arylacetaldehyde **1b**, and after column chromatography using ethyl acetate/hexanes (1:15) as eluent gave compound **3b** (Method I: 167 mg, 52%; Method II: 183 mg, 57%) as colorless oil; ^{1}H NMR (300 MHz, $CDCl_3$, 25 ºC): δ 7.43 (m, 2H), 7.32 (m, 3H), 4.59–4.43 (m, 2H), 4.35 (d, 1H, J = 6.8 Hz), 2.95–2.91 (m, 2H), 2.86 (q,

1H, J = 2.5 Hz), 2.58 (d, 1H, J = 7.3 Hz), 1.72 (t, 3H, J = 2.6 Hz), 1.42 (t, 3H, J = 3.2 Hz); $^{13}C\{1H\}$ NMR (75 MHz, $CDCl_3$, 25 °C): δ 207.1, 142.4, 127.7 (2C), 127.1, 125.8 (2C), 98.9, 79.7, 78.1, 77.1, 75.8, 74.4, 30.7, 15.7, 3.5; IR ($CHCl_3$): ν 1090, 1955, 3505 cm^{-1}; HRMS (ESI) *m/z*: $[M+Na]^+$ calcd for $C_{16}H_{18}NaO_2$: 265. 1199; found 265.1199.

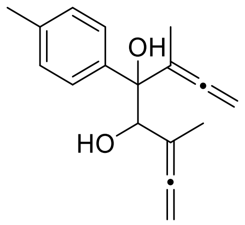

2c

Diol 2c. From 178 mg (1.20 mmol) of 2-oxo-2-arylacetaldehyde **1c**, and after column chromatography using ethyl acetate/hexanes (1:15) as eluent gave compound **2c** (Method I: 89 mg, 29%; Method II: 117 mg, 38%) as yellow oil; ^1H NMR (300 MHz, $CDCl_3$, 25 °C): δ 7.31 (d, 2H, J = 8.2 Hz), 7.11 (d, 2H, J = 7.9 Hz), 5.04–4.88 (m, 2H), 4.62 (s, 1H), 4.54 (dtd, 1H, J = 7.4, 3.2, 1.0 Hz), 4.37 (dtt, 1H, , J = 10.0, 3.2, 1.6 Hz), 3.09 (s, 1H), 2.33 (s, 4H), 1.56 (t, 3H, , J = 3.2 Hz), 1.35 (t, 3H, J = 3.2 Hz); $^{13}C\{1H\}$ NMR (75 MHz, $CDCl_3$, 25 °C): δ 207.8, 204.4, 138.3, 136.6, 128.4 (2C), 125.8 (2C), 104.2, 98.3, 80.7, 76.8, 76.4, 74.9, 21.0, 15.6, 14.5; IR ($CHCl_3$): ν 1094, 1958, 3508 cm^{-1}; HRMS (ESI) *m/z*: $[M+H]^+$ calcd for $C_{17}H_{21}O_2$: 257.1536; found 257.1523.

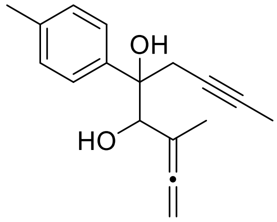

3c

Diol 3c. From 178 mg (1.20 mmol) of 2-oxo-2-arylacetaldehyde **1c**, and after column chromatography using ethyl acetate/hexanes (1:15) as eluent gave compound **3c** (Method I: 69 mg, 22%; Method II: 82 mg, 26%) as yellow oil; ^1H NMR (300 MHz, $CDCl_3$, 25 °C): δ 7.31 (d, 2H, J = 8.3 Hz), 7.13 (d, 2H, J = 8.0 Hz), 4.58–4.44 (m, 2H), 4.33 (s, 1H), 3.00–2.87 (m, 2H),

2.86–2.82 (m, 1H), 2.63 (d, 1H, J = 2.6 Hz), 2.34 (s, 3H), 1.75–1.68 (m, 3H), 1.46–1.37 (m, 3H); ^{13}C{1H} NMR (75 MHz, $CDCl_3$, 25 °C): δ 207.0, 139.4, 136.6, 128.4 (2C), 125.6 (2C), 99.0, 79.6, 78.0, 77.1, 75.8, 74.5, 30.7, 21.0, 15.7, 3.5; IR ($CHCl_3$): ν 1094, 1959, 3504 cm^{-1}; HRMS (ESI) *m/z*: $[M+Na]^+$ calcd for $C_{17}H_{20}NaO_2$: 279.1356; found 279.1349.

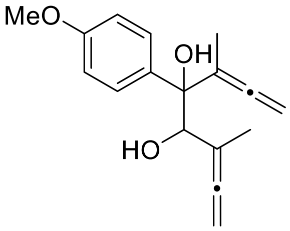

2d

Diol 2d. From 202.4 mg (1.23 mmol) of 2-oxo-2-arylacetaldehyde **1d**, and after column chromatography using ethyl acetate/hexanes (1:15) as eluent gave compound **2d** (Method I: 63 mg, 19%; Method II: 86 mg, 26%) as yellow oil; ^{1}H NMR (300 MHz, $CDCl_3$, 25 °C): δ 7.37–7.22 (m, 2H), 6.85–6.72 (m, 2H), 4.99–4.79 (m, 2H), 4,53 (s, 1H), 4.59–4.42 (m, 1H), 4.37–4.29 (m, 1H), 3.73 (s, 3H), 3.03 (s, 1H), 2.43 (s, 1H), 1.49 (t, 3H, J = 3.1 Hz), 1.29 (t, 3H, J = 3.2 Hz); ^{13}C{1H} NMR (75 MHz, $CDCl_3$, 25 °C): δ 207.7, 204.4, 133.4, 131.3, 127.1 (2C), 113.0 (2C), 104.2, 98.3, 80.5, 76.7, 76.4, 74.9, 55.1, 15.6, 14.5; IR ($CHCl_3$): ν 1092, 1956, 3507 cm^{-1}; HRMS (ESI) *m/z*: $[M+H]^+$ calcd for $C_{17}H_{21}O_3$: 273.1485; found 273.1500.

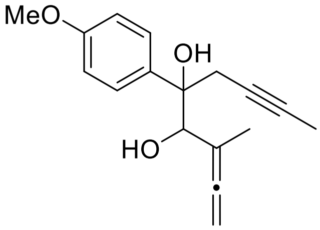

3d

Diol 3d. From 202.4 mg (1.23 mmol) of 2-oxo-2-arylacetaldehyde **1d**, and after column chromatography using ethyl acetate/hexanes (1:15) as eluent gave compound **3d** (Method I: 90 mg, 27%; Method II: 103 mg, 31%) as yellow oil; ^{1}H NMR (300 MHz, $CDCl_3$, 25 °C): δ 7.37–7.31 (m, 2H), 6.90–6.83 (m, 2H), 4.60–4.47 (m, 2H), 4.31 (s, 1H), 3.80 (s, 3H), 2.97–2.75 (m,

2H), 2.80 (dq, 1H, J = 16.5, 2.5 Hz), 2.60 (s, 1H), 1.72 (t, 3H, J = 2.5 Hz), 1.42 (t, 3H, J = 3.2 Hz); $^{13}C\{1H\}$ NMR (75 MHz, $CDCl_3$, 25 °C): δ 207.0, 158.6, 134.5, 127.0 (2C), 113.0 (2C), 99.0, 79.6, 77.9, 77.1, 75.8, 74.5, 55.1, 30.6, 15.7, 3.5; IR ($CHCl_3$): ν 1095, 1958, 3503 cm^{-1}; HRMS (ESI) *m/z*: $[M+Na]^+$ calcd for $C_{17}H_{20}NaO_3$: 295.1305; found 295.1307.

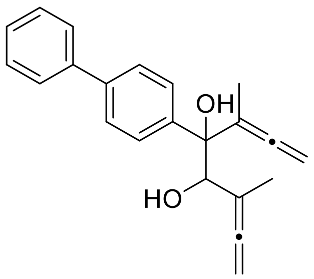

2e

Diol 2e. From 250 mg (1.19 mmol) of 2-oxo-2-arylacetaldehyde **1e**, and after column chromatography using ethyl acetate/hexanes (1:17) as eluent gave compound **2e** (Method I: 138 mg, 44%; Method II: 166 mg, 53%) as colorless solid; mp 96–98 °C ^1H NMR (300 MHz, acetone-d_6, 25 °C): δ 7.67 (m, 2H), 7.58 (m, 2H), 7.45 (m, 2H), 7.34 (m, 1H), 4.93 (m, 2H), 4.84 (m, 1H), 4.47 (dd, 1H, J = 4.3, 1.5 Hz), 4.39 (dqd, 1H, J = 9.6, 3.2, 0.8 Hz), 4.08 (dqd, 1H, J = 9.7, 3.2, 1.0 Hz), 4.02 (s, 1H), 1.55 (t, 3H, J = 3.1 Hz), 1.52 (t, 3H, J = 3.2 Hz); $^{13}C\{1H\}$ NMR (75 MHz, acetone-d_6, 25 °C): δ 209.2, 206.2, 143.2, 141.7, 140.1, 129.7 (2C), 128.0, 127.6 (2C), 127.5 (2C), 126.6 (2C), 104.9, 99.8, 81.3, 76.7, 76.6, 73.5, 15.0, 14.7; IR ($CHCl_3$): ν 1091, 1955, 3508 cm^{-1}; HRMS (ESI) *m/z*: $[M+Na]^+$ calcd for $C_{22}H_{22}NaO_2$: 341.1512; found 341.1509.

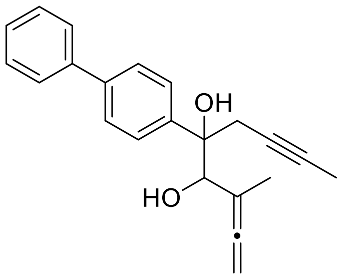

3e

Diol 3e. From 250 mg (1.19 mmol) of 2-oxo-2-arylacetaldehyde **1e**, and after column chromatography using ethyl acetate/hexanes (1:17) as eluent gave compound **3e** (Method I: 106

mg, 33%; Method II: 122 mg, 38%) as colorless solid; m.p. 102–103 ºC; ^{1}H NMR (300 MHz, acetone-d$_6$, 25 ºC): δ 7.65 (m, 6H), 7.45 (m, 2H), 7.34 (m, 1H), 4.57 (d, 1H, J = 5.1 Hz), 4.46 (m, 2H), 4.27 (ddq, 1H, J = 7.8, 3.2, 1.6 Hz), 3.98 (s, 1H), 2.65 (dq, 1H, J = 16.5, 2.6 Hz), 2.81 (dq, 1H, J = 16.5, 2.6 Hz), 1.65 (t, 3H, J = 2.6 Hz), 1.52 (t, 3H, J = 3.2 Hz); ^{13}C{1H} NMR (75 MHz, acetone-d$_6$, 25 ºC): δ 208.8, 143.8, 141.7, 139.8, 130.0 (2C), 128.0, 127.8 (2C), 127.6 (2C), 126.4 (2C), 99.9, 79.4, 78.6, 77.3, 76.5, 74.1, 31.9, 15.1, 3.4; IR (CHCl$_3$): ν 1095, 1957, 3501 cm^{-1}; HRMS (ESI) *m/z*: [M+Na]$^+$ calcd for $C_{22}H_{22}NaO_2$: 341.1512; found 341.1511.

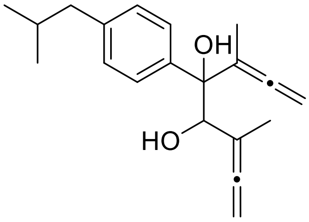

2f

Diol 2f. From 300 mg (1.60 mmol) of 2-oxo-2-arylacetaldehyde **1f**, and after column chromatography using ethyl acetate/hexanes (1:17) as eluent gave compound **2f** (Method I: 198 mg, 42%; Method II: 231 mg, 49%) as yellow oil; ^{1}H NMR (300 MHz, acetone-d$_6$, 25 ºC): δ 7.37 (d, 2H, J = 8.3 Hz), 7.09 (d, 2H, J = 8.3 Hz), 4.90 (m, 2H), 4.74 (s, 1H), 4.36 (m, 2H), 4.02 (dqd, 1H, J = 9.7, 3.2, 1.1 Hz), 3.88 (s, 1H), 2.46 (m, 2H), 1.86 (hept, 1H, J = 6.7 Hz), 1.51 (t, 3H, J = 3.1 Hz), 1.47 (t, 3H, J = 3.2), 0.88 (m, 6H); ^{13}C{1H} NMR (75 MHz, acetone-d$_6$, 25 ºC): δ 209.0, 206.2, 141.1, 140.7, 128.9 (2C), 126.7 (2C), 104.9, 99.8, 81.4, 76.7, 76.5, 73.3, 45.6, 31.0, 22.6, 22.5, 15.0, 14.7; IR (CHCl$_3$): ν 1093, 1953, 3506 cm^{-1}; HRM S (ESI) *m/z*: [M+Na]$^+$ calcd for $C_{20}H_{26}NaO_2$: 321.1825; found 321.1825.

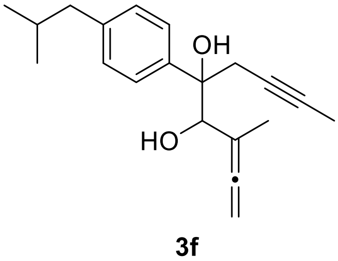

3f

Diol 3f. From 300 mg (1.60 mmol) of 2-oxo-2-arylacetaldehyde **1f**, and after column chromatography using ethyl acetate/hexanes (1:17) as eluent gave compound **3f** (Method I: 131 mg, 27%; Method II: 160 mg, 33%) as yellow oil; ^{1}H NMR (300 MHz, acetone-d$_6$, 25 ^{o}C): δ 7.44 (d, 2H, *J* = 8.3 Hz), 7.08 (d, 2H, *J* = 8.3 Hz), 4.43 (m, 2H), 4.27 (d, 1H, *J* = 5.8 Hz), 4.20 (dqd, 1H, *J* = 9.7, 3.3, 1.3 Hz), 2.87 (dq, 1H, *J* = 16.4, 2.7 Hz), 3.83 (s, 1H), 2.76 (dq, 1H, *J* = 16.5, 2.6 Hz), 2.5 (d, 2H, *J* = 7.2 Hz), 1.85 (hept, 1H, *J* = 6.8 Hz), 1.63 (t, 3H, *J* = 2.6 Hz), 1.44 (t, 3H, *J* = 3.2 Hz), 0.89 (d, 6H, *J* = 6.6 Hz); ^{13}C{1H} NMR (75 MHz, acetone-d$_6$, 25 ^{o}C): δ 208.6, 141.9, 140.4, 128.7 (2C), 126.9 (2C), 100.0, 79.3, 78.4, 77.5, 76.7, 74.0, 45.6, 31.8, 31.0, 22.7, 22.6, 15.2, 3.4; IR (CHCl$_3$): ν 1097, 1955, 3504 cm^{-1}; HRM S (ESI) *m/z*: [M+Na]$^{+}$ calcd for $C_{20}H_{26}NaO_2$: 321.1825; found 321.1830.

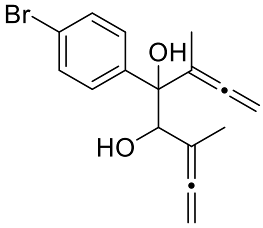

2g

Diol 2g. From 250 mg (1.17 mmol) of 2-oxo-2-arylacetaldehyde **1g**, and after column chromatography using ethyl acetate/hexanes (1:17) as eluent gave compound **2g** (Method I: 78 mg, 21%; Method II: 119 mg, 32%) as yellow solid; m.p. 66–69 ^{o}C; ^{1}H NMR (300 MHz, acetone-d$_6$, 25 ^{o}C): δ 7.47 (d, 2H, *J* = 8.9 Hz), 7.41 (d, 2H, *J* = 8.9 Hz), 4.91 (m, 2H), 4.78 (d, 1H, *J* = 3.0 Hz), 4.50 (d, 1H, *J* = 3.9 Hz), 4.39 (m, 1H), 4.09 (m, 2H), 1.50 (m, 6H); ^{13}C{1H} NMR (75 MHz, acetone-d$_6$, 25 ^{o}C): δ 209.1, 205.0, 143.5, 131.2 (2C), 129.1 (2C), 121.0, 104.6, 99.6, 81.2, 76.9, 76.5, 73.5, 14.9, 14.5; IR (CHCl$_3$): ν 1092, 1955, 3506 cm^{-1}; HRMS (ESI) *m/z*: [M-H]$^{-}$ calcd for $C_{16}H_{16}BrO_2$ 319.0339; found 319.0339.

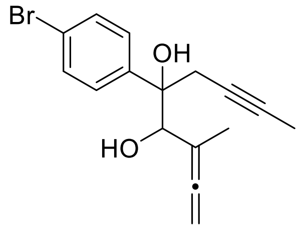

3g

Diol 3g. From 250 mg (1.17 mmol) of 2-oxo-2-arylacetaldehyde **1g**, and after column chromatography using ethyl acetate/hexanes (1:17) as eluent gave compound **3g** (Method I: 51 mg, 13%; Method II: 59 mg, 15%) as yellow solid; m.p. 73–75 ºC; ^{1}H NMR (300 MHz, acetone-d_6, 25 ºC): δ 7.48 (m, 4H), 4.46 (m, 3H), 4.25 (dqd, 1H, *J* = 9.9, 3.2, 1.0 Hz), 4.02 (s, 1H), 2.87 (dq, 1H, *J* = 16.5, 2,6 Hz), 2.73 (dq, 1H, *J* = 16.5, 2.6 Hz), 1.63 (t, 3H, *J* = 2.6 Hz), 1.49 (t, 3H, *J* = 3.2 Hz); ^{13}C{1H} NMR (75 MHz, acetone-d_6, 25 ºC): δ 208.8, 144.0, 130.9, 129.5, 120.9, 99.7, 79.3, 78.8, 77.0, 76.2, 74.1, 31.8, 15.0, 3.3; IR ($CHCl_3$): ν 1094, 1954, 3505 cm^{-1}; HRMS (ESI) *m/z*: [M-H]$^-$ calcd for $C_{16}H_{16}BrO_2$: 319.0339; found 319.0337.

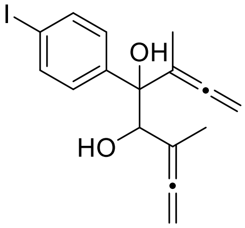

2h

Diol 2h. From 250 mg (0.96 mmol) of 2-oxo-2-arylacetaldehyde **1h**, and after column chromatography using ethyl acetate/hexanes (1:17) as eluent gave compound **2h** (Method I: 134 mg, 38%; Method II: 141 mg, 40%) as yellow solid; m.p. 77–79 ºC; ^{1}H NMR (300 MHz, acetone-d_6, 25 ºC): δ 7.67 (d, 2H, *J* = 8.6 Hz), 7.28 (d, 2H, *J* = 8.6 Hz), 4.91 (m, 2H), 4.77 (m, 1H), 4.58 (d, 1H, *J* = 4.4 Hz), 4.39 (m, 1H), 4.08 (m, 2H), 1.50 (m, 6H); ^{13}C{1H} NMR (75 MHz, acetone-d_6, 25 ºC): δ 209.1, 205.0, 144.1, 137.3 (2C), 129.3 (2C), 104.6, 99.6, 92.5, 81.2, 76.9, 76.4, 73.5, 14.9, 14.5; IR ($CHCl_3$): ν 1091, 1953, 3503 cm^{-1}; HRMS (ESI) *m/z*: [M-H]$^-$ calcd for $C_{16}H_{16}IO_2$: 367.0200; found 367.0199.

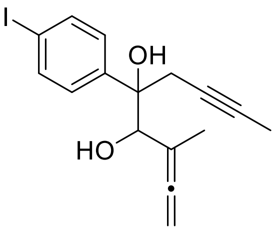

3h

Diol 3h. From 250 mg (0.96 mmol) of 2-oxo-2-arylacetaldehyde **1h**, and after column chromatography using ethyl acetate/hexanes (1:17) as eluent gave compound **3h** (Method I: 83 mg, 24%; Method II: 97 mg, 28%) as yellow solid; m.p. 81–84 ºC; ^{1}H NMR (300 MHz, acetone-d_6, 25 ºC): δ 7.66 (d, 2H, *J* = 8.6 Hz), 7.36 (d, 2H, *J* = 8.6 Hz), 4.47 (m, 3H), 4.25 (dq, 1H, *J* = 9.8, 3.2 Hz), 4.02 (s, 1H), 2.87 (m, 1H), 2.73 (m, 1H), 1.63 (t, 3H, *J* = 2.6 Hz), 1.49 (t, 3H, *J* = 3.2 Hz); $^{13}C\{1H\}$ NMR (75 MHz, acetone-d_6, 25 ºC): δ 208.8, 144.6, 137.0 (2C), 129.7 (2C), 99.7, 92.5, 79.3, 78.7, 77.0, 76.2, 74.1, 31.7, 15.0, 3.3; IR ($CHCl_3$): ν 1096, 1955, 3507 cm^{-1}; HRMS (ESI) *m/z*: $[M-H]^-$ calcd for $C_{16}H_{16}IO_2$: 367.0200; found 367.0200.

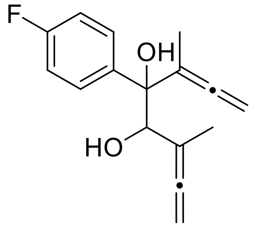

2i

Diol 2i. From 250 mg (1.65 mmol) of 2-oxo-2-arylacetaldehyde **1i**, and after column chromatography using ethyl acetate/hexanes (1:17) as eluent gave compound **2i** (Method I: 169 mg, 39%; Method II: 217 mg, 50%) as yellow solid; m.p. 68–70 ºC; ^{1}H NMR (300 MHz, CD_3CN, 25 ºC): δ 7.44 (dd, 2H, *J* = 9.0, 5.5 Hz), 7.03 (t, 2H, *J* = 9.0 Hz), 4.93 (m, 2H), 4.65 (s, 1H), 4.45 (m, 1H), 4.14 (m, 1H), 3.59 (s, 1H), 3.46 (s, 1H), 1.48 (t, 3H, *J* = 3.1 Hz), 1.43 (t, 3H, *J* = 3.2 Hz); $^{13}C\{1H\}$ NMR (75 MHz, CD_3CN, 25 ºC): δ 209.0, 205.9, 162.8 (d, 1C, *J* = 242.7 Hz), 139.7 (d, 1C, *J* = 3.0 Hz), 128.9 (d, 2C, *J* = 8.0 Hz), 115.0 (d, 2C, *J* = 21.5 Hz), 104.8, 99.4, 81.2, 77.3, 76.8,

74.0, 14.94, 14.87; IR ($CHCl_3$): ν 1094, 1954, 3505 cm^{-1}; HRMS (ESI) *m/z*: $[M+H]^+$ calcd for $C_{16}H_{18}FO_2$: 261.1285; found 261.1307.

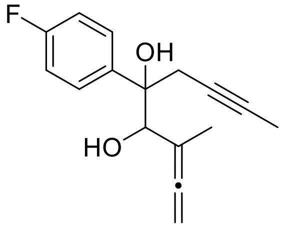

3i

Diol 3i. From 250 mg (1.65 mmol) of 2-oxo-2-arylacetaldehyde **1i**, and after column chromatography using ethyl acetate/hexanes (1:17) as eluent gave compound **3i** (Method I: 123 mg, 29%; Method II: 157 mg, 36%) as yellow oil; ^1H NMR (300 MHz, CD_3CN, 25 °C): δ 7.47 (dd, 2H, *J* = 9.0, 5.5 Hz), 7.05 (t, 2H, *J* = 9.0 Hz), 4.48 (m, 1H), 4.30 (m, 2H), 3.45 (s, 2H), 2.61 (m, 2H), 1.64 (t, 3H, *J* = 2.6 Hz), 1.41 (t, 3H, *J* = 3.1 Hz); $^{13}C\{1H\}$ NMR (75 MHz, CD_3CN, 25 °C): δ 208.5, 162.7 (d, 1C, *J* = 242.7 Hz), 140.4 (d, 1C, *J* = 2.9 Hz), 129.2 (d, 2C, *J* = 8.0 Hz), 114.9 (d, 2C, *J* = 21.1 Hz), 99.6, 79.42, 79.38, 77.6, 76.3, 74.8, 31.5, 15.4, 3.4; IR ($CHCl_3$): ν 1092, 1953, 3503 cm^{-1}; HRMS (ESI) *m/z*: $[M+H]^+$ calcd for $C_{16}H_{18}FO_2$: 261.1285; found 261.1317.

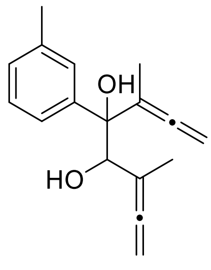

2j

Diol 2j. From 120 mg (0.81 mmol) of 2-oxo-2-arylacetaldehyde **1j**, and after column chromatography using ethyl acetate/hexanes (1:15) as eluent gave compound **2j** (Method I: 108 mg, 34%; Method II: 137 mg, 43%) as yellow oil; ^1H NMR (300 MHz, acetone-d_6, 25 °C): δ 7.40 (d, 1H, *J* = 0.7 Hz), 7.37 (d, 1H, *J* = 1.1 Hz), 7.28 (t, 1H, *J* = 7.6 Hz), 7.14 (d, 1H, *J* = 7.4 Hz), 5.10–4.93 (m, 2H), 4.91 (d, 1H, *J* = 4.3 Hz), 4.54–4.44 (m, 1H), 4.17 (dqd, 1H, *J* = 7.4, 3.2, 0.9

Hz), 4.02 (s, 1H, OH), 2.94 (s, 1H, OH), 2.43 (s, 3H, Me), 1.62 (t, 3H, J = 3.1 Hz), 1.58 (t, 3H, J = 3.2 Hz); ^{13}C{1H} NMR (75 MHz, acetone-d$_6$, 25 ^{o}C): δ 209.9, 207.0, 144.4, 137.9, 128.6 (2C), 128.1, 124.6, 105.6, 100.3, 81.8, 77.2, 77.1, 73.8, 22.3, 15.6, 15.1; IR (CHCl$_3$): ν 1096, 1952, 3506 cm^{-1}; HRMS (ESI) *m/z*: [M+Na]$^+$ calcd for $C_{17}H_{20}NaO_2$: 279.1356; found 279.1358.

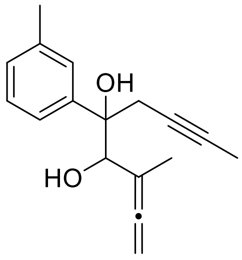

3j

Diol 3j. From 120 mg (0.81 mmol) of 2-oxo-2-arylacetaldehyde **1j**, and after column chromatography using ethyl acetate/hexanes (1:15) as eluent gave compound **3j** (Method I: 36 mg, 11%; Method II: 46 mg, 14%) as yellow oil; ^{1}H NMR (300 MHz, acetone-d$_6$, 25 ^{o}C): δ 7.47 (dd, 1H, J = 6.0, 1.1 Hz), 7.43 (d, 1H, J = 0.5 Hz), 7.28 (dd, 1H J = 9.4, 5.8 Hz), 7.14 (dd, 1H, J = 7.5, 0.6 Hz), 4.56 (dqd, 1H, J = 7.3, 3.1, 1.0 Hz), 4.45 (d, 1H, J = 5.8 Hz), 4.35 (dqd, 1H, J = 7.6, 3.2, 1.2 Hz), 3.03–2.92 (m, 2H), 2.87 (q, 1H, J = 2.6 Hz), 2.82 (q, 1H, J = 2.6 Hz), 2.44 (s, 3H), 1.76 (t, 3H, J = 2.6 Hz), 1.57 (t, 3H, J = 3.2 Hz); ^{13}C{1H} NMR (75 MHz, acetone-d$_6$, 25 ^{o}C): δ 209.8, 145.2, 137.9, 129.0, 128.8, 128.7, 125.1, 100.8, 80.1, 79.3, 78.1, 77.4, 74.7, 32.9, 22.6, 15.9, 4.2; IR (CHCl$_3$): ν 1094, 1954, 3506 cm^{-1}; HRMS (ESI) *m/z*: [M+Na]$^+$ calcd for $C_{17}H_{20}NaO_2$: 279.1356; found 279.1355.

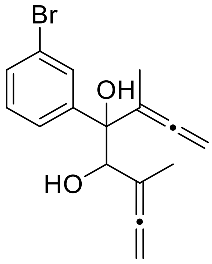

2k

Diol 2k. From 250 mg (1.17 mmol) of 2-oxo-2-arylacetaldehyde **1k**, and after column chromatography using ethyl acetate/hexanes (1:17) as eluent gave compound **2k** (Method I: 146 mg, 39%; Method II: 183 mg, 49%) as yellow oil; ^{1}H NMR (300 MHz, acetone-d$_6$, 25 ^{o}C): δ 7.64 (m, 1H), 7.42 (m, 2H), 7.25 (td, 1H, *J* = 7.9, 0.4 Hz), 4.93 (m, 2H), 4.79 (s, 1H), 4.56 (s, 1H), 4.40 (dqd, 1H, *J* = 10.2, 3.2, 0.8 Hz), 4.14 (s, 1H), 4.08 (dqd, 1H, *J* = 9.9, 3.2, 1.0 Hz), 1.51 (m, 6H); ^{13}C{1H} NMR (75 MHz, acetone-d$_6$, 25 ^{o}C): δ 209.2, 206.2, 146.8, 130.5, 130.2, 129.9, 125.9, 122.1, 104.6, 99.5, 81.1, 77.0, 76.5, 73.5, 14.9, 14.4; IR ($CHCl_3$): ν 1094, 1953, 3508 cm^{-1}; HRMS (ESI) *m/z*: [M-H]$^-$ calcd for $C_{16}H_{16}BrO_2$: 319.0339; found 319.0339.

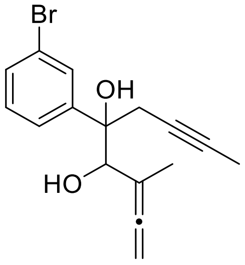

3k

Diol 3k. From 250 mg (1.17 mmol) of 2-oxo-2-arylacetaldehyde **1k**, and after column chromatography using ethyl acetate/hexanes (1:17) as eluent gave compound **3k** (Method I: 81 mg, 22%; Method II: 99 mg, 27%) as yellow oil; ^{1}H NMR (300 MHz, acetone-d$_6$, 25 ^{o}C): **δ** 7.74 (t, 1H, *J* = 1.8 Hz), 7.53 (ddd, 1H, *J* = 7.8, 1.7, 1.1 Hz), 7.40 (ddd, 1H, *J* = 8.0, 2.1, 1.1 Hz), 7.25 (t, 1H, *J* = 7.9 Hz), 4.52 (s, 2H), 4.47 (m, 1H), 4.25 (m, 1H), 4.10 (s, 1H), 2.90 (dq, 1H, *J* = 16.6, 2.6 Hz), 2.73 (dq, 1H, *J* = 16.6, 2.6 Hz), 1.64 (t, 3H, *J* = 2.6 Hz), 1.50 (t, 3H, *J* = 3.2 Hz); ^{13}C{1H} NMR (75 MHz, acetone-d$_6$, 25 ^{o}C): δ 208.9, 147.3, 130.5, 130.2, 129.9, 126.2, 121.9, 99.6, 79.3, 78.9, 77.0, 76.2, 74.0, 31.8, 14.9, 3.0; IR ($CHCl_3$): ν 1091, 1952, 3504 cm^{-1}; HRMS (ESI) *m/z*: [M-H]$^-$ calcd for $C_{16}H_{16}BrO_2$: 319.0339; found 319.0337.

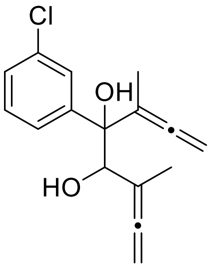

2l

Diol 2l. From 250 mg (1.5 mmol) of 2-oxo-2-arylacetaldehyde **1l**, and after column chromatography using ethyl acetate/hexanes (1:17) as eluent gave compound **2l** (Method I: 176 mg, 43%; Method II: 209 mg, 51%) as yellow oil; H NMR (300 MHz, acetone-d_6, 25 °C): δ 7.49 (m, 1H), 7.39 (dt, 1H, *J* = 7.7, 1.6 Hz), 7.32 (td, 1H, *J* = 7.7, 0.5 Hz), 7.25 (ddd, 1H, *J* = 7.8, 2.1, 1.5 Hz), 4.93 (m, 2H), 4.79 (s, 1H), 4.55 (s, 1H), 4.39 (m, 1H), 4.14 (s, 1H), 4.07 (dqd, 1H, *J* = 9.8, 3.2, 1.0 Hz), 1.51 (m, 6H); ^{13}C{1H} NMR (75 MHz, acetone-d_6, 25 °C): δ 209.2, 206.2, 146.6, 133.9, 129.9, 127.5, 126.9, 125.5, 104.6, 99.5, 81.1, 77.0, 76.5, 73.4, 14.9, 14.4; IR ($CHCl_3$): ν 1095, 1953, 3507 cm^{-1}; HRMS (ESI) *m/z*: [M-H]$^-$ calcd for $C_{16}H_{16}ClO_2$: 275.0844; found 275.0848.

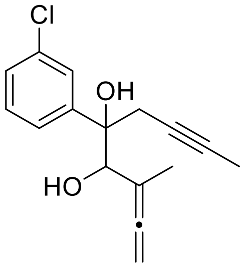

3l

Diol 3l. From 250 mg (1.5 mmol) of 2-oxo-2-arylacetaldehyde **1l**, and after column chromatography using ethyl acetate/hexanes (1:17) as eluent gave compound **3l** (Method I: 96 mg, 23%; Method II: 125 mg, 30%) as yellow oil; ^{1}H NMR (300 MHz, acetone-d_6, 25 °C): δ 7.59 (m, 1H), 7.48 (dt, 1H, *J* = 7.6, 1.5 Hz), 7.30 (m, 1H), 7.25 (ddd, 1H, *J* = 7.9, 2.1, 1.3 Hz), 4.52 (s, 1H), 4.46 (dq, 1H, *J* = 9.7, 3.2 Hz), 4.24 (m, 1H), 4.10 (s, 1H), 2.91 (dq, 1H, *J* = 16.5, 2.6 Hz), 2.75 (dq, 1H, *J* = 16.6, 2.6 Hz), 1.64 (t, 3H, *J* = 2.6 Hz), 1.50 (t, 3H, *J* = 3.2 Hz); ^{13}C{1H} NMR (75 MHz, acetone-d_6, 25 °C): δ 208.8, 147.1, 133.6, 129.6, 127.3, 125.8, 99.6, 79.3, 78.8, 77.1, 76.2,

74.0, 31.7, 15.0, 3.3; IR ($CHCl_3$): ν 1095, 1953, 3506 cm^{-1}; HRMS (ESI) *m/z*: $[M-H]^-$ calcd for $C_{16}H_{16}ClO_2$: 275.0844; found 275.0846.

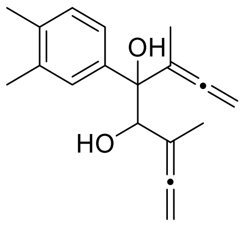

2m

Diol 2m. From 250 mg (1.54 mmol) of 2-oxo-2-arylacetaldehyde **1m**, and after column chromatography using ethyl acetate/hexanes (1:17) as eluent gave compound **2m** (Method I: 157 mg, 38%; Method II: 194 mg, 47%) as yellow oil; ^1H NMR (300 MHz, acetone-d_6, 25 °C): δ 7.19 (m, 2H), 7.03 (d, 1H, *J* = 7.8 Hz), 4.88 (m, 1H), 4.78 (s, 1H), 4.38 (m, 2H), 4.07 (dqd, 1H, *J* = 9.7, 3.2, 1.0 Hz), 3.85 (s, 1H), 2.23 (s, 3H), 2.21 (s, 3H), 1.50 (t, 3H, *J* = 3.1 Hz), 1.46 (t, 3H, *J* = 3.2 Hz); $^{13}C\{1H\}$ NMR (75 MHz, acetone-d_6, 25 °C): δ 209.3, 206.7, 141.2, 135.8, 135.3, 129.4, 128.1, 124.4, 105.2, 99.9, 81.0, 76.6, 76.5, 73.3, 20.0, 19.4, 15.0, 14.6; IR ($CHCl_3$): ν 1093, 1951, 3506 cm^{-1}; HRMS (ESI) *m/z*: $[M+Na]^+$ calcd for $C_{18}H_{22}NaO_2$: 293.1512; found 293.1511.

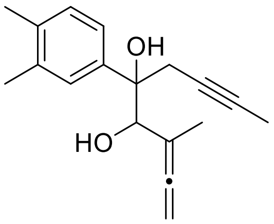

3m

Diol 3m. From 250 mg (1.54 mmol) of 2-oxo-2-arylacetaldehyde **1m**, and after column chromatography using ethyl acetate/hexanes (1:17) as eluent gave compound **3m** (Method I: 117 mg, 28%; Method II: 142 mg, 34%) as yellow oil; ^1H NMR (300 MHz, acetone-d_6, 25 °C): δ 7.25 (m, 2H), 7.03 (m, 1H), 4.46 (m, 2H), 4.27 (m, 2H), 3.79 (s, 1H), 2.83 (m, 1H), 2.70 (dq, 1H, *J* = 16.4, 2.6 Hz), 2.21 (s, 3H), 2.24 (s, 3H), 1.64 (t, 3H, *J* = 2.6 Hz), 1.44 (t, 3H, *J* = 3.2 Hz); $^{13}C\{1H\}$ NMR (75 MHz, acetone-d_6, 25 °C): δ 208.9, 141.9, 135.5, 135.0, 129.2, 128.4, 124.6, 100.1, 79.0,

78.4, 77.2, 76.7, 73.9, 32.1, 20.1, 19.4, 15.1, 3.4; IR ($CHCl_3$): ν 1093, 1952, 3504 cm^{-1}; HRMS (ESI) *m/z*: $[M+Na]^+$ calcd for $C_{18}H_{22}NaO_2$: 293.1512; found 293.1512.

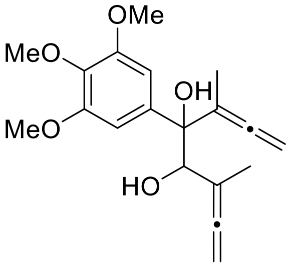

2n

Diol 2n. From 250 mg (1.12 mmol) of 2-oxo-2-arylacetaldehyde **1n**, and after column chromatography using ethyl acetate/hexanes (1:10) as eluent gave compound **2n** (Method I: 96 mg, 26%; Method II: 128 mg, 34%) as colorless oil; ^{1}H NMR (300 MHz, acetone-d$_6$, 25 °C): δ 6.76 (s, 2H), 4.91 (m, 2H), 4.76 (s, 1H), 4.41 (m, 2H), 4.13 (dqd, 1H, *J* = 9.7, 3.2, 1.0 Hz), 3.98 (s, 1H), 3.79 (s, 6H), 3.71 (s, 3H), 1.55 (t, 3H, *J* = 3.1 Hz), 1.52 (t, 3H, *J* = 3.2 Hz); ^{13}C{1H} NMR (75 MHz, acetone-d$_6$, 25 °C): δ 209.3, 206.2, 153.6 (2C), 139.5, 138.1, 104.9, 104.7 (2C), 99.8, 81.2, 76.7, 76.6, 73.2, 60.5, 56.4 (2C), 15.1, 14.6; IR ($CHCl_3$): ν 1092, 1952, 3504 cm^{-1}; HRMS (ESI) *m/z*: $[M+Na]^+$ calcd for $C_{19}H_{24}NaO_5$: 355.1516; found 355.1510.

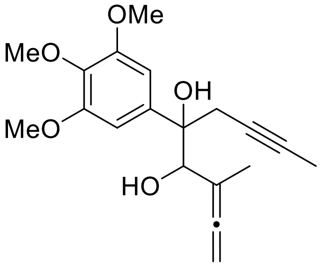

3n

Diol 3n. From 250 mg (1.12 mmol) of 2-oxo-2-arylacetaldehyde **1n**, and after column chromatography using ethyl acetate/hexanes (1:10) as eluent gave compound **3n** (Method I: 120 mg, 32%; Method II: 139 mg, 37%) as colorless oil; ^{1}H NMR (300 MHz, acetone-d$_6$, 25 °C): δ 6.89 (s, 2H), 4.57 (d, 1H, *J* = 5.8 Hz), 4.46 (dqd, 1H, *J* = 9.6, 3.2, 1.0 Hz), 4.38 (d, 1H, *J* = 5.8 Hz), 4.29 (dqd, 1H, *J* = 9.8, 3.2, 1.1 Hz), 3.93 (s, 1H), 3.80 (s, 6H), 3.72 (s, 3H), 2.88 (m, 1H),

2.67 (dq, 1H, J = 16.3, 2.6 Hz), 1.69 (t, 3H, J = 2.6 Hz), 1.51 (t, 3H, J = 3.2 Hz); ^{13}C{1H} NMR (75 MHz, acetone-d$_6$, 25 °C): δ 209.0, 153.4 (2C), 140.1, 138.0, 105.0 (2C), 100.0, 79.1, 78.6, 76.9, 76.8, 73.8, 60.5, 56.4 (2C), 32.2, 15.0, 3.4; IR ($CHCl_3$): ν 1091, 1953, 3503 cm^{-1}; HRMS (ESI) *m/z*: [M+Na]$^+$ calcd for $C_{19}H_{24}NaO_5$: 355.1516; found 355.1514.

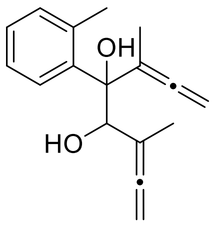

2o

Diol 2o. From 349 mg (2.35 mmol) of 2-oxo-2-arylacetaldehyde **1o**, and after column chromatography using ethyl acetate/hexanes (1:15) as eluent gave compound **2o** (Method I: 96 mg, 16%; Method II: 126 mg, 21%) as yellow oil; ^{1}H NMR (300 MHz, acetone-d$_6$, 25 °C): δ 7.95 (dd, 1H, J = 7.3, 2.0 Hz), 7.28–7.25 (m, 1H), 7.23 (dd, 1H, J = 5.3, 3.3 Hz), 7.21–7.17 (m, 1H), 5.03–4.92 (m, 2H), 4.67 (d, 1H, J = 4.4 Hz), 4.57 (m, 1H), 4.31–4.25 (m, 1H), 2.98 (s, 1H), 2.59 (s, 1H), 2.49 (s, 3H, J = 3.7 Hz), 1.60 (t, 3H, J = 3.2 Hz), 1.52 (t, 3H, J = 3.0 Hz); ^{13}C{1H} NMR (75 MHz, acetone-d$_6$, 25 °C): δ 208.7, 207.6, 141.4, 136.9, 133.1, 128.9, 128.6, 126.7, 103.3, 100.6, 82.2, 77.3, 76.4, 74.2, 22.8, 15.7, 14.8; IR ($CHCl_3$): ν 1093, 1951, 3505 cm^{-1}; HRMS (ESI) *m/z*: [M+Na]$^+$ calcd for $C_{17}H_{20}NaO_2$: 279.1356; found 279.1350.

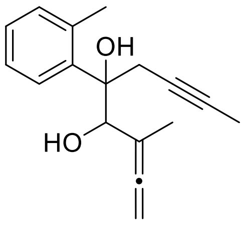

3o

Diol 3o. From 349 mg (2.35 mmol) of 2-oxo-2-arylacetaldehyde **1o**, and after column chromatography using ethyl acetate/hexanes (1:15) as eluent gave compound **3o** (Method I: 90 mg, 15%; Method II: 114 mg, 19%) as yellow oil; ^{1}H NMR (300 MHz, acetone-d$_6$, 25 °C): δ 8.07

(d, 1H, J = 8.1 Hz), 7.70–7.66 (m, 1H), 7.60–7.54 (m, 1H), 7.42 (d, 1H, J = 7.9 Hz), 4.57 (m, 1H), 4.42–4.36 (m, 1H), 3.98 (s, 1H), 3.06 (dq, 2H, J = 6.7, 2.6 Hz), 2.99 (d, 1H, J = 2.6 Hz), 2.71 (s, 1H), 2.64 (s, 3H), 1.69 (t, 3H, J = 2.6 Hz), 1.64 (t, 3H, J = 3.2 Hz); ^{13}C{1H} NMR (75 MHz, acetone-d$_6$, 25 ºC): δ 209.1, 142.7, 137.1, 133.5, 129.8, 128.3, 126.3, 101.0, 82.0, 79.1, 77.2, 77.1, 75.0, 31.9, 24.0, 4.1, 16.0; IR (CHCl$_3$): ν 1093, 1955, 3507 cm^{-1}; HRMS (ESI) *m/z*: [M+Na]$^+$ calcd for $C_{17}H_{20}NaO_2$: 279.1356; found 279.1346.

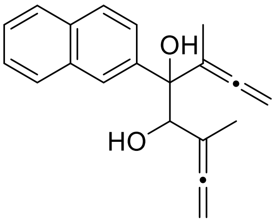

2p

Diol 2p. From 250 mg (1.35 mmol) of 2-oxo-2-arylacetaldehyde **1p**, and after column chromatography using ethyl acetate/hexanes (1:17) as eluent gave compound **2p** (Method I: 133 mg, 33%; Method II: 165 mg, 41%) as orange solid; m.p. 91–93 ºC; ^{1}H NMR (300 MHz, acetone-d$_6$, 25 ºC): δ 8.10 (m, 1H), 7.86 (m, 3H), 7.49 (m, 3H), 4.96 (m, 1H), 4.56 (d, 1H, J = 4.3 Hz), 4.35 (m, 1H), 4.13(s, 1H), 3.91 (dqd, 1H, J = 9.7, 3.2, 0.9 Hz), 1.53 (t, 3H, J =3.1 Hz), 1.49 (t, 3H, J = 3.2 Hz); ^{13}C{1H} NMR (75 MHz, acetone-d$_6$, 25 ºC): δ 209.1, 205.0, 141.5, 133.9, 133.5, 129.0, 128.3, 127.5, 126.7, 126.5, 125.6, 125.3, 104.9, 99.9, 81.4, 76.8, 76.5, 73.5, 14.9, 14.4; IR (CHCl$_3$): ν 1092, 1953, 3505 cm^{-1}; HRMS (ESI) *m/z*: [M+Na]$^+$ calcd for $C_{20}H_{20}NaO_2$: 315.1356; found 315.1356.

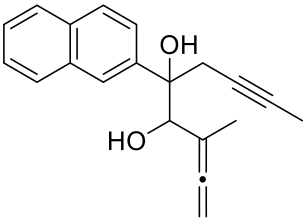

3p

Diol 3p. From 250 mg (1.35 mmol) of 2-oxo-2-arylacetaldehyde **1p**, and after column chromatography using ethyl acetate/hexanes (1:17) as eluent gave compound **3p** (Method I: 92 mg, 23%; Method II: 108 mg, 27%) as orange solid; m.p. 102–104 °C; ^{1}H NMR (300 MHz, acetone-d_6, 25 °C): δ 8.09 (m, 1H), 7.86 (m, 3H), 7.69 (dd, 1H, *J* = 8.7, 1.8 Hz), 7.46 (m, 2H), 4.65 (m, 1H, *J* = 4.65 Hz), 4.49 (d, 1H, *J* = 5.8 Hz), 4.41 (m, 1H), 4.12 (m, 1H), 4.08 (s, 1H), 3.01 (dq, 1H, *J* = 16.5, 2.6 Hz), 2.86 (m, 1H), 1.60 (t, 3H, *J* = 2.6 Hz), 1.48 (t, 3H, *J* = 3.2 Hz); ^{13}C{1H} NMR (75 MHz, acetone-d_6, 25 °C): δ 208.8, 142.2, 133.9, 133.4, 129.0, 128.2, 127.2, 126.5, 126.4, 126.0, 125.9, 100.0, 79.6, 78.7, 77.3, 76.5, 74.0, 32.0, 15.0, 3.3; IR ($CHCl_3$): ν 1092, 1954, 3506 cm^{-1}; HRMS (ESI) *m/z*: $[M+Na]^+$ calcd for $C_{20}H_{20}NaO_2$: 315.1356; found 315.1350.

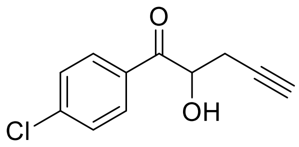

Ia

Synthesis of 1-(4-chlorophenyl)-2-hydroxypent-4-yn-1-one Ia. Propargyl bromide (281 mg, 2.36 mmol) was added to a stirred suspension of indium powder (474 mg, 4.13 mmol) and 2-oxo-2-arylacetaldehyde **1a** (100 mg, 0.59 mmol) in THF/NH_4Cl (aq., sat.) (1:5) (3 mL). The reaction was stirred at room temperature until disappearance of the starting material (TLC, 24 h), and the crude was extracted with ethyl acetate (3 x 4 mL). The organic extracts were dried ($MgSO_4$) and concentrated under reduced pressure. Purification of the residue by column chromatography eluting with ethyl acetate/hexanes (1:30), afforded compound **Ia** (9.8 mg, 8 %) as a yellow oil; ^{1}H NMR (300 MHz, $CDCl_3$, 25 °C): δ 7.88 (d, 2H, *J* = 8.63 Hz), 7.50 (d, 2H, *J* = 8.6 Hz), 5.17 (q, 1H, *J* = 5.4 Hz), 3.88 (d, 1H, *J* = 7.3 Hz), 2.81 (ddd, 1H, *J* = 17.1, 4.8, 2.7 Hz), 2.66 (ddd, 1H, *J* = 17.1, 5.4, 2.7 Hz), 2.03 (t, 1H, *J* = 2.7 Hz); ^{13}C{1H} NMR (75 MHz, $CDCl_3$, 25 °C): δ 198.4, 140.9, 131.7, 130.0 (2C), 129.4 (2C), 78.2, 72.2, 71.0, 26.2; HRMS (ESI) *m/z*: $[M-H]^-$ calcd for $C_{11}H_8ClO_2$: 207.0218; found 207.0219.

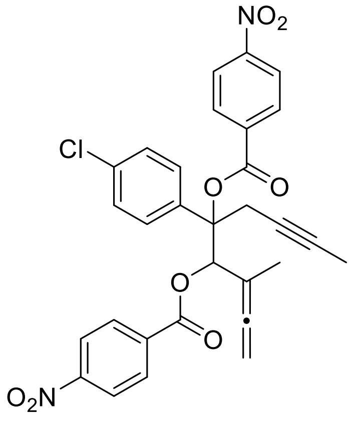

3a-NO$_2$

Synthesis of bis(4-nitrobenzoate) 3a-NO$_2$. 4-Nitrobenzoyl chloride (67 mg, 0.36 mmol) was added to a stirring solution of diol **3a** (34.0 mg, 0.12 mmol), NEt_3 (61 mg, 0.60 mmol) and DMAP (1.5 mg, 0.012 mmol) in DCM (1.8 mL). The reaction was stirred at rt until disappearance of the starting material (TLC), quenched with a saturated aqueous $NaHCO_3$ solution (1.5 mL) and extracted with DCM (3 x 5 mL). The organic extracts were dried ($MgSO_4$) and concentrated under reduced pressure. The crude reaction was purified by column chromatography using ethyl acetate/hexanes (1:90) as eluent to give compound **3a-NO$_2$** (45.3 mg, 71%) as a colorless solid; m.p. 191–193 °C; ^{1}H NMR (300 MHz, acetone-d$_6$, 25 °C): δ 8.38 (s, 4H), 8.37 (d, 2H, *J* = 9.1 Hz), 8.31 (d, 2H, *J* = 9.1 Hz), 7.60 (d, 2H, *J* = 8.9 Hz), 7.47 (d, 2H, *J* = 8.9 Hz), 6.19 (t, 1H, *J* = 1.5 Hz), 3.72 (m, 2H), 1.59 (m, 6H); ^{13}C{1H} NMR (75 MHz, acetone-d$_6$, 25 °C): δ 209.1, 164.3, 163.6, 151.8, 151.7, 138.2, 136.9, 136.0, 134.6, 131.9 (2C), 131.7 (2C), 129.3 (2C), 128.9 (2C), 124.8 (2C), 124.7 (2C), 95.4, 86.6, 80.7, 79.2, 77.1, 74.1, 26.6, 17.0, 3.2; IR ($CHCl_3$): ν 1341, 1530, 1746 cm^{-1}; HRMS (ESI) *m/z*: [M+Na]$^+$ calcd for $C_{30}H_{23}ClN_2NaO_8$: 597.1035, found 597.1033.

Table S2. Synthesis of bis(dihydrofuran) **4a** under modified gold-catalyzed conditions

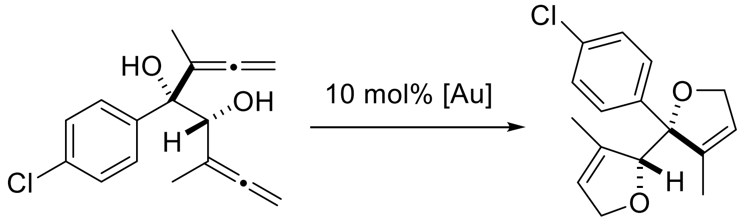

2a **4a**

Entry	[Au]	Reaction Conditions[a]	Yield (%)[b]
1	$[(PPh_3)AuCl]/AgOTf$	DCE (0.01 M), MW, 80 ^{0}C, 2 h	---
2	$[(PPh_3)AuCl]/AgSbF_6$	DCE (0.01 M), MW, 80 ^{0}C, 2 h	---
3	$[(Ph_3P)AuNTf_2]$	DCE (0.01 M), MW, 80 ^{0}C, 2 h	---
4	$XPhosAuNTf_2$	DCE (0.01 M), MW, 80 ^{0}C, 2 h	---
5	**A**/AgOTf	DCE (0.01 M), MW, 80 ^{0}C, 2 h	---
6	**A**/$AgSbF_6$	DCE (0.01 M), MW, 80 ^{0}C, 2 h	---
7	**B**/AgOTf	DCE (0.01 M), MW, 80 ^{0}C, 1 h	6
8	**B**/$AgSbF_6$	DCE (0.01 M), MW, 80 ^{0}C, 1 h	7
9	**C**/AgOTf	DCE (0.01 M), MW, 80 ^{0}C, 2 h	---
10	$AuCl_3$	DCE (0.01 M), MW, 80 ^{0}C, 1 h	20
11	$AuBr_3$	DCE (0.01 M), MW, 80 ^{0}C, 1 h	43
12	$AuBr_3$	MeCN (0.01 M), MW, 80 ^{0}C, 1 h	22
13	$AuBr_3$	toluene (0.01 M), MW, 80 ^{0}C, 1 h	19
14	$AuBr_3$	DCE (0.1 M), MW, 80 ^{0}C, 1 h	54
15	$AuBr_3$	DCE (0.005 M), MW, 80 ^{0}C, 1.5 h	41
16	$AuBr_3$	DCE (0.01 M), 0 ^{0}C, 4 h	52
17	$AuBr_3$	DCE (0.1 M), 0 ^{0}C, 3 h	51
18	$AuBr_3$/2,2'-Bipyridyl	DCE (0.01 M), MW, 80 ^{0}C, 5 h	---
19	$AuBr_3/P(OMe)_3$	DCE (0.01 M), MW, 80 ^{0}C, 5 h	---
20	$AuBr_3$/AgOTf	DCE (0.01 M), MW, 80 ^{0}C, 1 h	17
21	(Pic)$AuCl_2$	DCE (0.01 M), MW, 80 ^{0}C, 1 h	40
22	$AuCl_3$·Py	DCE (0.01 M), MW, 80 ^{0}C, 5 h	---

[a] MW = Reactions were carried out under microwave irradiation. [b] Yield of pure, isolated product with correct analytical and spectral data.

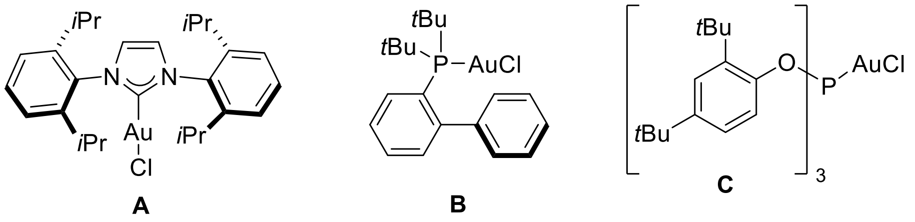

General procedure for the synthesis of bis(dihydrofurans) 4. $AuBr_3$ (44 mg, 0.10 mmol) was added to a solution of the appropriate diol **2** (1.0 mmol) in 1,2-dichloroethane (DCE) (10 mL) under argon atmosphere. Then, the reaction was heated at 80 ^oC under microwave irradiation until

disappearance of the starting material (TLC). Next, the mixture was filtered through a celite pad, washed with DCM, and the solvent was evaporated under reduced pressure. The crude reaction was purified by column chromatography using ethyl acetate/hexanes mixtures. Spectroscopic and analytical data for compounds **4a–p** follow.

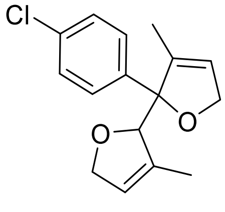

4a

Bis(dihydrofuran) 4a. From 50 mg (0.18 mmol) of diol **2a**, and after column chromatography using ethyl acetate/hexanes (1:10) as eluent, gave compound **4a** (26.9 mg, 54%) as a yellow oil; ^{1}H NMR (300 MHz, $CDCl_3$, 25 ºC): δ 7.35 (d, 2H, *J* = 8.8 Hz), 7.26 (d, 2H, *J* = 8.8 Hz), 5.58 (dt, 1H *J* = 3.3, 1.6 Hz), 5.50 (dd, 1H, *J* = 3.1, 1.6 Hz), 4.74–4.61 (m, 2H), 5.18 (s, 1H), 4.60–4.46 (m, 2H), 1.76 (dd, 3H, *J* = 3.8, 2.1 Hz), 1.49 (s, 3H); ^{13}C{1H} NMR (75 MHz, $CDCl_3$, 25 ºC): δ 138.4, 136.4, 133.0, 130.5, 126.0 (2C), 124.2 (2C), 121.5, 118.7, 92.2, 86.3, 72.8, 72.4, 13.6, 11.6; IR ($CHCl_3$) : ν 1075 cm^{-1}; HRMS (ESI) *m/z*: [M+Na]$^+$ calcd for $C_{16}H_{18}ClO_2$: 299.0809; found 299.0794.

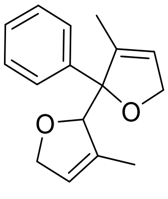

4b

Bis(dihydrofuran) 4b. From 35 mg (0.14 mmol) of diol **2b**, and after column chromatography using ethyl acetate/hexanes (1:10) as eluent, gave compound **4b** (13.6 mg, 39%) as a brown oil; ^{1}H NMR (300 MHz, $CDCl_3$, 25 ºC): δ 7.42 (t, 1H, *J* = 1.8 Hz), 7.39 (d, 2H, *J* = 1.1 Hz), 7.34–7.24 (m, 2H), 5.57 (dt, 1H *J* = 3.3, 1.6 Hz), 5.48 (dd, 1H, *J* = 3.1, 1.6 Hz), 5.24 (s, 1H, *J* = 1.7 Hz), 4.78–4.63 (m, 2H), 4.61–4.46 (m, 2H), 1.78 (dd, 3H, *J* = 3.8, 2.1 Hz), 1.08 (dd, 3H, *J* = 1.4, 1.0

Hz); $^{13}C\{1H\}$ NMR (75 MHz, $CDCl_3$, 25 °C): δ 142.2, 139.2, 135.7, 128.3 (2C), 127.0, 125.1 (2C), 123.7, 120.7, 94.9, 88.8, 75.3, 74.8, 14.5, 12.8; IR ($CHCl_3$) : ν 1074 cm^{-1}; HRMS (ESI) *m/z*: $[M+H]^+$ calcd for $C_{16}H_{19}O_2$: 243.1380; found 243.1370.

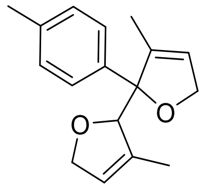

4c

Bis(dihydrofuran) 4c. From 30.6 mg (0.12 mmol) of diol **2c**, and after column chromatography using ethyl acetate/hexanes (1:10) as eluent, gave compound **4c** (13.7 mg, 45%) as a brown oil; ^1H NMR (300 MHz, $CDCl_3$, 25 °C): δ 7.35 (d, 2H, J = 7.7 Hz), 7.16 (d, 2H, J = 7.7 Hz), 5.64 (s, 1H), 5.54 (s, 1H), 5.29 (s, 1H), 4.75 (m, 2H), 4.66–4.55 (m, 2H), 2.37 (s, 3H), 1.84 (t, 3H, J = 6.0 Hz), 1.18 (s, 3H); $^{13}C\{1H\}$ NMR (175 MHz, $CDCl_3$, 25 °C): δ 136.6, 135.8, 132.3, 128.9, 124.9 (2C), 123.5 (2C), 120.5, 113.7, 94.8, 88.7, 75.2, 74.7, 29.7, 14.6, 12.7; IR ($CHCl_3$) : ν 1076 cm^{-1}; HRMS (ESI) *m/z*: $[M+Na]^+$ calcd for $C_{17}H_{20}NaO_2$: 279.1361; found 279.1356.

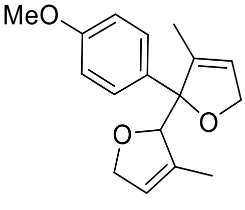

4d

Bis(dihydrofuran) 4d. From 36.4 mg (0.13 mmol) of diol **2d**, and after column chromatography using ethyl acetate/hexanes (1:10) as eluent, gave compound **4d** (19.2 mg; 55%) as a yellow oil; ^1H NMR (300 MHz, $CDCl_3$, 25 °C): δ 7.41–7.36 (m, 2H), 6.92–6.86 (m, 2H), 5.64 (dt, 1H, J = 3.2, 1.6 Hz), 5.54 (dd, 1H, J = 3.1, 1.5 Hz), 5.27 (s, 1H), 4.81–4.67 (m, 2H), 4.65–4.53 (m, 2H), 3.80 (s, 3H), 1.55 (s, 3H), 1.21–1.18 (m, 3H); $^{13}C\{1H\}$ NMR (175 MHz, $CDCl_3$, 25 °C): δ 139.3, 135.8, 134.3, 127.5, 126.2 (2C), 123.5 (2C), 120.4, 113.6, 89.7, 88.7, 75.2, 74.6, 55.2, 14.6, 12.7;

IR ($CHCl_3$) : ν 1075 cm^{-1}; HRMS (ESI) *m/z*: $[M+Na]^+$ calcd for $C_{17}H_{20}NaO_3$: 295.1305; found 295.1312.

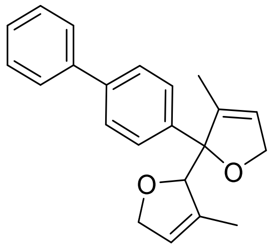

4e

Bis(dihydrofuran) 4e. From 50 mg (0.19 mmol) of diol **2e**, and after column chromatography using ethyl acetate/hexanes (1:10) as eluent, gave compound **4e** (29.6 mg, 49%) as a yellow oil; ^1H NMR (300 MHz, acetone-d_6, 25 °C): δ 7.66 (m, 6H), 7.46 (m, 2H), 7.35 (m, 1H), 5.59 (hept, 1H, J = 1.7 Hz), 5.54 (h, 1H, J = 1.6 Hz), 5.33 (m, 1H), 4.66 (m, 2H), 4.49 (m, 2H), 1.81 (q, 3H, J = 2.0 Hz), 1.23 (s, 3H); $^{13}C\{1H\}$ NMR (75 MHz, acetone-d_6, 25 °C): δ 143.1, 141.5, 140.2, 136.8, 129.7 (2C), 128.1, 127.6 (2C), 127.3 (2C), 127.0 (2C), 124.2, 121.7, 95.6, 89.6, 75.3, 74.9, 14.8, 13.0; IR ($CHCl_3$) : ν 1077 cm^{-1}; HRMS (ESI) *m/z*: $[M+Na]^+$ calcd for $C_{22}H_{22}NaO_2$: 341.1512; found 341.1506.

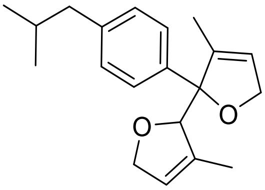

4f

Bis(dihydrofuran) 4f. From 50 mg (0.17 mmol) of diol **2f**, and after column chromatography using ethyl acetate/hexanes (1:10) as eluent, gave compound **4f** (22.7 mg, 45%) as a yellow oil; ^1H NMR (300 MHz, acetone-d_6, 25 °C): δ 7.43 (d, 2H, J = 8.3 Hz), 7.14 (d, 2H, J = 8.1 Hz), 5.56 (hept, 1H, J = 1.7 Hz), 5.49 (h, 1H, J = 1.6 Hz), 5.26 (dtd, 1H, J = 5.9, 2.0, 1.1 Hz), 4.63 (m, 2H), 4.45 (m, 2H), 2.47 (d, 2H, J = 7.2 Hz), 1.85 (n, 1H, J = 6.9 Hz), 1.77 (td, 3H, J = 2.2, 1.6 Hz), 1.15 (qd, 3H, J = 2.2, 1.1 Hz), 0.88 (d, 6H, J = 6.6 Hz); $^{13}C\{1H\}$ NMR (75 MHz, acetone-d_6, 25 °C): δ 141.2, 140.8, 140.4, 136.9, 129.5 (2C), 126.1(2C), 124.1, 121.3, 95.6, 89.6, 75.2, 74.7, 45.5, 31.0,

22.6 (2C), 14.7, 13.0; IR ($CHCl_3$) : ν 1074 cm^{-1}; HRMS (ESI) *m/z*: $[M+Na]^+$ calcd for $C_{20}H_{26}NaO_2$: 321.1825; found 321.1822.

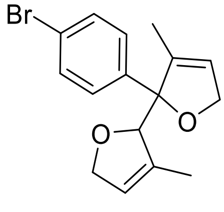

4g

Bis(dihydrofuran) 4g. From 60 mg (0.19 mmol) of diol **2g**, and after column chromatography using ethyl acetate/hexanes (1:10) as eluent, gave compound **4g** (24.4 mg, 43%) as a yellow oil; ^{1}H NMR (300 MHz, acetone-d_6, 25 °C): δ 7.52 (m, 4H), 5.59 (hept, 1H, *J* = 1.7 Hz), 5.54 (h, 1H, *J* = 1.6 Hz), 5.25 (dtt, 1H, *J* = 5.4, 2.0, 1.0 Hz), 4.63 (m, 2H), 4.46 (m, 2H), 1.76 (td, 3H, *J* = 2.2, 1.5 Hz), 1.20 (qd, 3H, *J* = 2.2, 1.1 Hz); ^{13}C{1H} NMR (75 MHz, acetone-d_6, 25 °C): δ 143.3, 139.8, 136.5, 131.9 (2C), 128.6 (2C), 124.4, 122.0, 121.2, 95.4, 89.4, 75.3, 74.9, 14.7, 12.9; IR ($CHCl_3$) : ν 1075 cm^{-1}; HRMS (ESI) *m/z*: $[M+Na]^+$ calcd for $C_{16}H_{17}NaBrO_2$: 343.0304; found 343.0301.

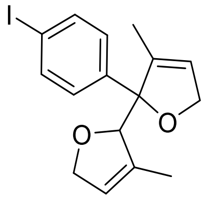

4h

Bis(dihydrofuran) 4h. From 50 mg (0.14 mmol) of diol **2h**, and after column chromatography using ethyl acetate/hexanes (1:10) as eluent, gave compound **4h** (25.8 mg, 50%) as a yellow oil; ^{1}H NMR (300 MHz, acetone-d_6, 25 °C): δ 7.73 (d, 2H, *J* = 8.6 Hz), 7.36 (d, 2H, *J* = 8.6 Hz), 5.59 (hept, 1H, *J* = 1.6 Hz), 5.54 (h, 1H, *J* = 1.6 Hz), 5.24 (dtt, 1H, *J* = 5.4, 2.0, 1.1 Hz), 4.46 (m, 2H), 4.63 (m, 2H), 1.76 (td, 3H, *J* = 2.2, 1.6 Hz), 1.20 (m, 3H); ^{13}C{1H} NMR (75 MHz, acetone-d_6, 25 °C): δ 143.9, 139.8, 137.9 (2C), 136.5, 128.8 (2C), 124.4, 122.0, 95.4, 92.7, 89.4, 75.3, 74.9, 14.8, 12.9; IR ($CHCl_3$) : ν 1075 cm^{-1}; HRMS (ESI) *m/z*: $[M+Na]^+$ calcd for $C_{16}H_{17}NaIO_2$: 391.0165; found 391.0156.

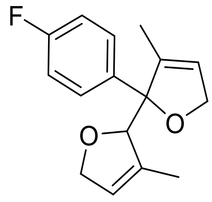

4i

Bis(dihydrofuran) 4i. From 50.0 mg (0.19 mmol) of diol **2i**, and after column chromatography using ethyl acetate/hexanes (1:10) as eluent, gave compound **4i** (18.8 mg, 38%) as a yellow oil; ^{1}H NMR (300 MHz, CD_3CN, 25 ^{o}C): δ 7.51 (dd, 2H, *J* = 9.0, 5.5 Hz), 7.09 (t, 2H, *J* = 9.0 Hz), 5.59 (hept, 1H, *J* = 1.7 Hz), 5.53 (h, 1H, *J* = 1.6 Hz), 5.24 (dtt, 1H, *J* = 5.4, 2.0, 1.0 Hz), 4.62 (m, 2H), 4.47 (m, 2H), 1.74 (td, 3H, *J* = 2.2, 1.6 Hz), 1.17 (tdd, 3H, *J* = 2.2, 1.5, 1.0 Hz); ^{13}C{1H} NMR (75 MHz, CD_3CN, 25 ^{o}C): δ 162.8 (d, 1C, *J* = 243.2 Hz), 140.2, 139.9, 136.8, 128.4 (d, 2C, *J* = 7.9 Hz), 124.6, 122.0, 115.6 (d, 2C, *J* = 21.4 Hz), 95.5, 89.5, 75.4, 75.0, 14.7, 12.9; IR ($CHCl_3$) : ν 1073 cm^{-1}; HRMS (ESI) *m/z*: $[M+H]^+$ calcd for $C_{16}H_{18}FO_2$: 261.1285; found 261.1295.

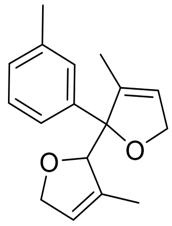

4j

Bis(dihydrofuran) 4j. From 36 mg (0.14 mmol) of diol **2j**, and after column chromatography using ethyl acetate/hexanes (1:10) as eluent, gave compound **4j** (14.7 mg; 41%) as a brown oil; ^{1}H NMR (300 MHz, $CDCl_3$, 25 ^{o}C): δ 7.31 (s, 1H), 7.25 (d, 1H, *J* = 1.1 Hz), 7.23 (d, 1H, *J* = 1.6 Hz), 7.10–7.06 (m, 1H), 5.64 (dd, 1H, *J* = 3.3, 1.6 Hz), 5.55 (dd, 1H, *J* = 3.1, 1.5 Hz), 5.29 (d, 1H, *J* = 3.6 Hz), 4.84–4.69 (m, 2H), 4.66–4.52 (m, 2H), 2.36 (s, 3H), 1.57 (s, 3H), 1.25 (s, 3H); ^{13}C{1H} NMR (175 MHz, $CDCl_3$, 25 ^{o}C): δ 138.0, 135.7, 128.0, 127.7, 125.8, 123.6, 122.2, 122.0, 120.7, 114.2, 94.9, 88.7, 75.3, 74.8, 21.7, 14.6, 12.8; IR ($CHCl_3$) : ν 1076 cm^{-1}; HRMS (ESI) *m/z*: $[M+Na]^+$ calcd for $C_{17}H_{20}NaO_2$: 279.1356; found 279.1358.

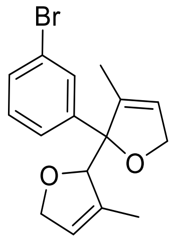

4k

Bis(dihydrofuran) 4k. From 50 mg (0.15 mmol) of diol **2k**, and after column chromatography using ethyl acetate/hexanes (1:10) as eluent, gave compound **4k** (20.7 mg, 43%) as a yellow oil; ^{1}H NMR (300 MHz, acetone-d$_6$, 25 °C): δ 7.72 (t, 1H, *J* = 1.9 Hz), 7.54 (ddd, 1H, *J* = 7.8, 1.7, 1.1 Hz), 7.45 (ddd, 1H, *J* = 7.9, 2.0, 1.1 Hz), 7.32 (t, 1H, *J* = 7.8 Hz), 5.60 (hept, 1H, 1.7 Hz), 5.57 (h, 1H, *J* = 1.6 Hz), 5.26 (dddd, 1H, *J* = 5.4, 3.1, 2.0, 1.1 Hz), 4.65 (m, 2H), 4.47 (m, 2H), 1.78 (td, 3H, *J* = 2.2, 1.5 Hz), 1.21 (dq, 3H, *J* = 3.3, 1.1 Hz); ^{13}C{1H} NMR (75 MHz, acetone-d$_6$, 25 °C): δ 146.6, 139.6, 136.4, 130.8, 130.7, 129.4, 125.4, 124.5, 122.9, 122.3, 95.3, 89.5, 75.3, 74.9, 14.7, 12.9; IR (CHCl$_3$) : ν 1075 cm^{-1}; HRMS (ESI) *m/z*: [M+Na]$^+$ calcd for $C_{16}H_{17}NaBrO_2$: 343.0304; found 343.0307.

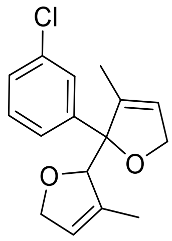

4l

Bis(dihydrofuran) 4l. From 50 mg (0.18 mmol) of diol **2l**, and after column chromatography using ethyl acetate/hexanes (1:10) as eluent, gave compound **4l** (22 mg, 44%) as a yellow oil; ^{1}H NMR (300 MHz, acetone-d$_6$, 25 °C): δ 7.57 (m, 1H), 7.49 (m, 1H), 7.38 (td, 1H, *J* = 7.8, 0.5 Hz), 7.30 (ddd,1H, *J* = 7.9, 2.1, 1.2 Hz), 5.6 (hept, 1H, *J* = 1.7 Hz), 5.57 (h, 1H, *J* = 1.6 Hz), 5.27 (dddd, 1H, *J* = 5.4, 3.1, 2.0, 1.0 Hz), 4.47 (m, 2H), 4.65 (m, 2H), 1.78 (td, 3H, *J* = 2.2, 1.5 Hz), 1.21 (qd, 3H, *J* = 2.2, 1.1 Hz); ^{13}C{1H} NMR (75 MHz, acetone-d$_6$, 25 °C): δ 146.4, 139.6, 136.4, 134.7, 130.5, 127.7, 126.5, 125.0, 124.5, 122.2, 95.3, 89.5, 75.3, 74.9, 14.7, 12.9; IR (CHCl$_3$) : ν 1074 cm^{-1}; HRMS (ESI) *m/z*: [M+Na]$^+$ calcd for $C_{16}H_{17}NaClO_2$: 299.0809; found 299.0808.

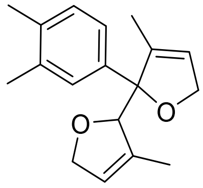

4m

Bis(dihydrofuran) 4m. From 50 mg (0.18 mmol) of diol **2m**, and after column chromatography using ethyl acetate/hexanes (1:10) as eluent, gave compound **4m** (17.5 mg, 36%) as a yellow oil; ^{1}H NMR (300 MHz, acetone-d$_6$, 25 ^{o}C): δ 7.30 (d, 1H, *J* = 2.0 Hz), 7.21 (dd, 1H, *J* = 7.9, 2.0 Hz), 7.09 (d, 1H, *J* = 7.9 Hz), 5.55 (hept, 1H, *J* = 1.7 Hz), 5.47 (h, 1H, *J* = 1.6 Hz), 5.26 (dtd, 1H, *J* = 5.8, 2.0, 1.0 Hz), 4.64 (m, 2H), 4.44 (m, 2H), 2.25 (s, 3H), 2.22 (s, 3H), 1.75 (td, 3H, *J* = 2.2, 1.5 Hz), 1.17 (m, 3H); ^{13}C{1H} NMR (75 MHz, acetone-d$_6$, 25 ^{o}C): δ 141.4, 140.1, 137.0, 136.7, 135.5, 129.9, 127.5, 124.0, 123.8, 121.1, 95.5, 89.5, 75.2, 74.7, 20.1, 19.3, 14.9, 13.1; IR ($CHCl_3$) : ν 1075 cm^{-1}; HRMS (ESI) *m/z*: [M+Na]$^+$ calcd for $C_{18}H_{22}NaO_2$: 293.1512; found 293.1513.

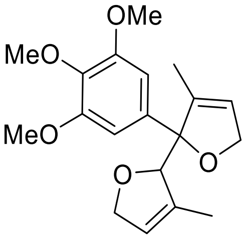

4n

Bis(dihydrofuran) 4n. From 35 mg (0.10 mmol) of diol **2n**, and after column chromatography using ethyl acetate/hexanes (1:5) as eluent, gave compound **4n** (14 mg, 42%) as a yellow oil; ^{1}H NMR (300 MHz, acetone-d$_6$, 25 ^{o}C): δ 6.81 (s, 2H), 5.57 (p, 1H, *J* = 1.7 Hz), 5.49 (q, 1H, *J* = 5.4 Hz), 5.29 (d, 1H, *J* = 5.4 Hz), 4.65 (m, 2H), 4.48 (m, 2H), 3.83 (s, 6H), 3.71 (s, 3H), 1.82 (q, 3H, *J* = 2.0 Hz), 1.22 (m, 3H); ^{13}C{1H} NMR (75 MHz, acetone-d$_6$, 25 ^{o}C): δ 153.2 (2C), 139.4, 138.4, 137.4, 136.0, 123.2, 120.5, 103.2 (2C), 94.8, 88.5, 74.4, 73.9, 59.7, 55.4 (2C), 13.7, 12.3; IR

(CHCl3) : ν 1077 cm^{-1}; HRMS (ESI) *m/z*: $[M+Na]^+$ calcd for $C_{19}H_{24}NaO_5$: 355.1516; found 355.1515.

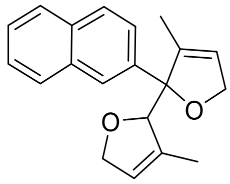

4p

Bis(dihydrofuran) 4p. From 50 mg (0.17 mmol) of diol **2p**, and after column chromatography using ethyl acetate/hexanes (1:10) as eluent, gave compound **4p** (20.3 mg, 41%) as a yellow oil; ^{1}H NMR (300 MHz, acetone-d$_6$, 25 °C): δ 8.06 (d, 1H, *J* = 1.7 Hz), 7.90 (m, 3H), 7.71 (dd, 1H, *J* = 8.6, 1.9 Hz), 7.48 (m, 2H), 5.59 (hept, 1H, *J* = 1.6 Hz), 5.55 (h, 1H, , *J* = 1.6 Hz), 5.46 (m, 1H), 4.72 (m, 2H), 4.51 (m, 2H), 1.82 (td, 3H, , *J* = 2.2, 1.6 Hz), 1.15 (qd, 3H, *J* = 2.2, 1.1 Hz); ^{13}C{1H} NMR (75 MHz, acetone-d$_6$, 25 °C): δ 141.6, 140.4, 136.8, 134.2, 133.5, 129.1, 128.4, 128.3, 126.8, 126.6, 125.1 (2C), 124.3, 121.7, 95.8, 89.6, 75.3, 75.0, 14.8, 13.1; IR (CHCl3) : ν 1075 cm^{-1}; HRMS (ESI) *m/z*: $[M+Na]^+$ calcd for $C_{20}H_{20}NaO_2$: 315.1356; found 315.1348.

Synthetic procedure for scale-up reaction of bis(dihydrofuran) 4a

To a solution of diol **2a** (332 mg, 1.2 mmol) in 1,2-dichloroethane (DCE) (12 mL) under argon atmosphere was added $AuBr_3$ (53 mg, 0.12 mmol). Then, the mixture was heated for 1 hour at 80 °C under microwave irradiation. Next, the reaction was allowed to cool at rt before was filtered through a celite pad and washed with DCM (6 mL). The resulting mixture was concentrated in vacuo and the crude residue was purified using silica gel column flash chromatography eluting with hexanes/ethyl acetate (1:10) to get the target product **4a** as a yellow oil (169 mg, 51% yield; which is comparable to the experiment on a lesser scale in Scheme 3, main manuscript).

Table S3. Synthesis of dihydrofuran **6a** under modified gold-catalyzed conditions

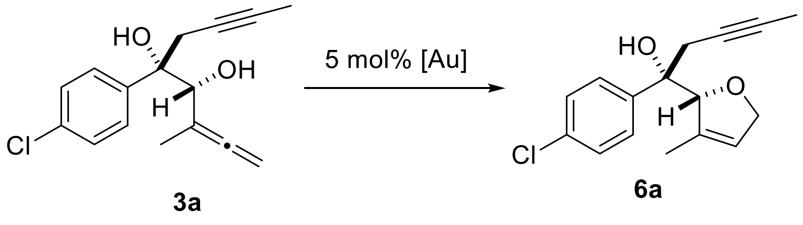

Entry	[Au]	Reaction Conditions	Yield (%)[b]
1	[(PPh_3)AuCl]/AgOTf	DCM (0.01 M), rt, 2 h	---
2	[(Ph_3P)$AuNTf_2$]	DCM (0.01 M), rt, 2 h	---
3	**A**/AgOTf	DCM (0.01 M), rt, 2 h	---
4	**A**/$AgSbF_6$	DCM (0.01 M), rt, 2 h	---
5	$AuCl_3$	DCE (0.01 M), MW, 80 ^{0}C, 0.5 h[a]	14
6	$AuBr_3$	DCE (0.01 M), MW, 80 ^{0}C, 0.5 h[a]	10
7	$AuCl_3$	DCM (0.01 M), rt, 1 h	23
8	$AuCl_3$	toluene (0.01 M), rt, 2 h	14
9	$AuCl_3$	MeCN (0.01 M), rt, 1 h	16
10	$AuCl_3$/AgOTf	DCM (0.01 M), rt, 1 h	7
11	(Pic)$AuCl_2$	DCE (0.01 M), rt, 24 h	14
12	$AuCl_3$	DCM (0.1 M), rt, 1 h	26
13	$AuCl_3$	DCM (0.01 M), 0 ^{0}C, 1.5 h	30
14	$AuCl_3$	DCM (0.1 M), 0 ^{0}C, 1 h	31

[a] MW = Reactions were carried out under microwave irradiation. [b] Yield of pure, isolated product with correct analytical and spectral data.

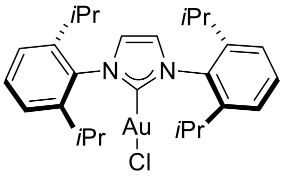

A

General procedure for the synthesis of dihydrofurans 6. $AuCl_3$ (15 mg, 0.05 mmol) was added to a solution of the appropriate diol **3** (1.0 mmol) in DCE (10 mL) at 0°C under argon atmosphere. Then, the reaction was stirred at 0°C until disappearance of the starting material (TLC). Next, the mixture was filtered through a celite pad, washed with DCM, and the solvent was evaporated under reduced pressure. The crude reaction was purified by column chromatography using ethyl acetate/hexanes mixtures. Spectroscopic and analytical data for compounds **6a–p** follow**.**

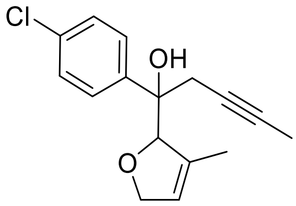

6a

Dihydrofuran 6a. From 40 mg (0.14 mmol) of diol **3a**, and after column chromatography using ethyl acetate/hexanes (1:10) as eluent, gave compound **6a** (12.1 mg, 31%) as a yellow oil; ^{1}H NMR (300 MHz, acetone-d$_6$, 25 ºC): δ 7.64 (d, 2H, *J* = 8.7 Hz), 7.35 (d, 2H, *J* = 8.7 Hz), 5.63 (hept, 1H, *J* = 1.6 Hz), 4.94 (tdt, 1H, *J* = 4.5, 2.0, 1.0 Hz), 4.60 (m, 1H), 4.42 (m, 1H), 4.04 (s, 1H), 2.81 (m, 1H), 2.64 (dq, 1H, *J* = 16.4, 2.5 Hz), 1.62 (t, 3H, *J* = 2.6 Hz), 1.26 (qd, 3H, *J* = 2.2, 1.1 Hz); ^{13}C{1H} NMR (75 MHz, acetone-d$_6$, 25 ºC): δ 143.9, 136.6, 132.9, 129.0 (2C), 128.2 (2C), 125.4, 92.5, 78.6, 77.7, 76.1, 75.0, 31.1, 14.9, 3.3; IR ($CHCl_3$): ν 1081, 2121 cm^{-1}; HRMS (ESI) *m/z*: [M+H]$^+$ calcd for $C_{16}H_{18}ClO_2$: 277.0990; found 277.0989.

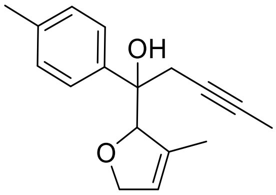

6c

Dihydrofuran 6c. From 30 mg (0.12 mmol) of diol **3c**, and after column chromatography using ethyl acetate/hexanes (1:10) as eluent, gave compound **6c** (10 mg, 33%) as a yellow oil; ^{1}H NMR (300 MHz, CD_3CN, 25 ºC): δ 7.40 (d, 2H, *J* = 8.2 Hz), 7.16 (d, 2H, *J* = 8.2 Hz), 5.60 (p, 1H, *J* = 1.7 Hz), 4.87 (m, 1H), 4.55 (dddd, 1H, *J* = 11.7, 5.8, 2.2, 1.6 Hz), 4.41 (m, 1H), 3.14 (s, 1H), 2.76 (dq, 1H, *J* = 16.5, 2.6 Hz), 2.64 (dq, 1H, *J* = 16.5, 2.6 Hz), 1.63 (t, 3H, *J* = 2.6 Hz), 2.32 (s, 3H), 1.19 (dq, 3H, *J* = 2.2, 1.0 Hz); ^{13}C{1H} NMR (75 MHz, CD_3CN, 25 ºC): δ 141.5, 137.3, 136.9, 129.2 (2C), 127.0 (2C), 125.4, 92.7, 78.9, 78.0, 76.6, 75.1, 31.2, 21.0, 14.8, 3.4; IR ($CHCl_3$): ν 1083, 2122 cm^{-1}; HRMS (ESI) *m/z*: [M+Na]$^+$ calcd for $C_{17}H_{20}NaO_2$: 279.1356; found 279.1359.

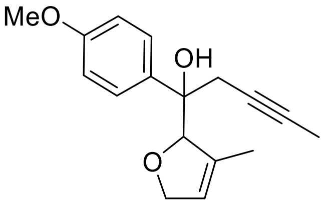

6d

Dihydrofuran 6d. From 30 mg (0.11 mmol) of diol **3d**, and after column chromatography using ethyl acetate/hexanes (1:10) as eluent, gave compound **6d** (9.6 mg, 32%) as a yellow oil; ^{1}H NMR (300 MHz, acetone-d$_6$, 25 °C): δ 7.53 (d, 2H, *J* = 9.0 Hz), 6.88 (d, 2H, *J* = 8.9 Hz), 5.60 (p, 1H, *J* = 1.7 Hz), 4.94 (dddd, 1H, *J* = 4.5, 3.3, 1.9, 1.0 Hz), 4.58 (dddd, 1H, *J* = 12.1, 5.9, 2.2, 1.6 Hz), 4.41 (ddt, 1H, *J* = 12.2, 3.5, 2.0 Hz), 3.79 (s, 3H), 3.76 (s, 1H), 2.80 (m, 1H), 2.62 (dq, 1H, *J* = 16.3, 2.6 Hz), 1.63 (t, 3H, *J* = 2.6 Hz), 1.23 (qt, 3H, *J* = 2.1, 1.0 Hz); ^{13}C{1H} NMR (75 MHz, acetone-d$_6$, 25 °C): δ 159.4, 137.0, 136.8, 128.1 (2C), 125.0, 113.5 (2C), 92.7, 78.3, 77.5, 76.6, 74.9, 55.4, 31.4, 14.9, 3.4; IR ($CHCl_3$): ν 1082, 2122 cm^{-1}; HRMS (ESI) *m/z*: [M+Na]$^{+}$ calcd for $C_{17}H_{20}NaO_3$: 295.1305; found 295.1303.

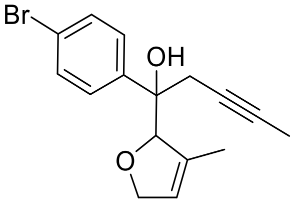

6g

Dihydrofuran 6g. From 25 mg (0.08 mmol) of diol **3g**, and after column chromatography using ethyl acetate/hexanes (1:10) as eluent, gave compound **6g** (7.4 mg, 29 %) as a yellow oil; ^{1}H NMR (300 MHz, CD_3CN, 25 °C): δ 7.48 (d, 4H, *J* = 4.2 Hz), 5.63 (p, 1H, *J* = 1.7 Hz), 4.86 (dq, 1H, *J* = 3.6, 1.0 Hz), 4.55 (m, 1H), 4.42 (ddt, 1H, *J* = 12.4, 3.8, 2.0 Hz), 3.29 (s, 1H), 2.76 (dq, 1H, *J* = 16.6, 2.6 Hz), 2.64 (dq, 1H, *J* = 16.5, 2.6 Hz), 1.63 (t, 3H, *J* = 2.6 Hz), 1.21 (tdt, 3H, *J* = 2.2, 1.6, 1.0 Hz); ^{13}C{1H} NMR (75 MHz, CD_3CN, 25 °C): δ 143.5, 136.0, 131.1, 128.9 (2C), 125.3, 120.8, 92.1, 78.8, 77.6, 75.7, 74.7, 30.6, 14.4, 3.0; IR ($CHCl_3$): ν 1081, 2124 cm^{-1}; HRMS (ESI) *m/z*: [M+H]$^{+}$ calcd for $C_{16}H_{18}BrO_2$: 321.0485; found 321.0494.

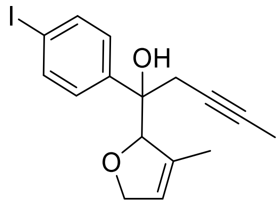

6h

Dihydrofuran 6h. From 30 mg (0.08 mmol) of diol **6h**, and after column chromatography using ethyl acetate/hexanes (1:10) as eluent, gave compound **6h** (12.1 mg, 40%) as a yellow oil; ^{1}H NMR (300 MHz, acetone-d$_6$, 25 °C): δ 7.70 (d, 2H, *J* = 8.6 Hz), 7.45 (d, 2H, *J* = 8.6 Hz), 5.63 (hept, 1H, *J* = 1.8 Hz), 4.93 (tdd, 1H, *J* = 4.6, 2.1, 1.0 Hz), 4.59 (m, 1H), 4.42 (m, 1H), 4.03 (s, 1H), 2.81 (m, 1H), 2.63 (dq, 1H, *J* = 16.5, 2.6 Hz), 1.62 (t, 3H, *J* = 2.6 Hz), 1.26 (dq, 3H, *J* = 3.4, 1.1 Hz); ^{13}C{1H} NMR (75 MHz, acetone-d$_6$, 25 °C): δ 144.9, 137.3 (2C), 136.6, 130.6, 129.6 (2C), 125.3, 92.7, 92.4, 78.7, 77.7, 76.1, 75.0, 31.0, 14.9, 3.3; IR ($CHCl_3$): ν 1083, 2125 cm^{-1}; HRMS (ESI) *m/z*: $[M+Na]^+$ calcd for $C_{16}H_{17}NaIO_2$: 391.0165; found 391.0148.

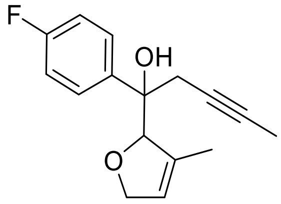

6i

Dihydrofuran 6i. From 39 mg (0.15 mmol) of diol **6i**, and after column chromatography using ethyl acetate/hexanes (1:10) as eluent, gave compound **6i** (8.3 mg, 21%) as a yellow oil; ^{1}H NMR (300 MHz, acetone-d$_6$, 25 °C): δ 7.65 (dd, 2H, *J* = 9.0, 5.5 Hz), 7.08 (t, 2H, *J* = 9.0 Hz), 5.63 (h, 1H, *J* = 1.7 Hz), 4.95 (dddd, 1H, *J* = 6.7, 3.2, 2.0, 1.0 Hz), 4.60 (m, 1H), 4.42 (ddt, 1H, *J* = 12.3, 3.8, 2.0 Hz), 3.98 (s, 1H), 2.86 (dq, 1H, *J* = 16.4, 2.6 Hz), 2.63 (dq, 1H, *J* = 16.4, 2.6 Hz), 1.62 (t, 3H, *J* = 2.6 Hz), 1.25 (m, 3H); ^{13}C{1H} NMR (75 MHz, acetone-d$_6$, 25 °C): δ 162.6 (d, 1C, *J* = 242.8 Hz), 140.9 (d, 1C, *J* = 3.0 Hz), 136.7, 129.0 (d, 2C, *J* = 7.9), 125.3, 114.8 (d, 2C, *J* = 21.0 Hz), 92.6, 78.5, 77.6, 76.3, 74.9, 31.3, 14.9, 3.3; IR ($CHCl_3$): ν 1080, 2122 cm^{-1}; HRMS (ESI) *m/z*: $[M+H]^+$ calcd for $C_{16}H_{18}FO_2$: 261.1285; found 261.1283.

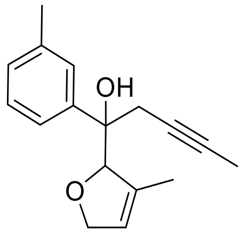

6j

Dihydrofuran 6j. From 25 mg (0.12 mmol) of diol **3j**, and after column chromatography using ethyl acetate/hexanes (1:10) as eluent, gave compound **6j** (5.9 mg, 24%) as a yellow oil; ^{1}H NMR (300 MHz, CD_3CN, 25 °C): δ 7.37 (s, 1H), 7.30 (dd, 1H, *J* = 1.3, 0.7 Hz), 7.22 (t, 1H, *J* = 7.6 Hz), 7.09 (m, 1H), 5.61 (p,1H, *J* = 1.7 Hz), 4.89 (dtd, 1H, *J* = 5.5, 2.2, 1.0 Hz), 4.55 (m, 1H), 4.42 (ddt, 1H, *J* = 12.3, 3.6, 1.9 Hz), 3.15 (s, 1H), 2.76 (dq, 1H, *J* = 16.5, 2.6 Hz), 2.66 (dq, 1H, *J* = 16.5, 2.6 Hz), 2.15 (s, 3H), 1.63 (t, 3H, *J* = 2.6 Hz), 1.17 (tdd, 3H, *J* = 2.2, 1.5, 1.0 Hz); ^{13}C{1H} NMR (75 MHz, acetone-d_6, 25 °C): δ 149.6, 144.7, 135.3, 128.1, 128.0, 127.7, 125.0, 124.2, 92.6, 78.3, 77.7, 76.6, 74.9, 31.4, 21.7, 14.8, 3.4; IR ($CHCl_3$): ν 1082, 2121 cm^{-1}; HRMS (ESI) *m/z*: $[M+Na]^+$ calcd for $C_{17}H_{20}NaO_2$: 279.1356; found 279.1359.

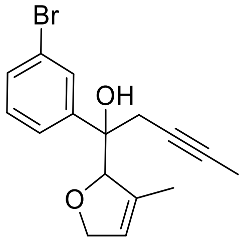

6k

Dihydrofuran 6k. From 20 mg (0.06 mmol) of diol **3k**, and after column chromatography using ethyl acetate/hexanes (1:10) as eluent, gave compound **6k** (6.2 mg, 31%) as a yellow oil; ^{1}H NMR (300 MHz, acetone-d_6, 25 °C): δ 7.84 (m, 1H), 7.61 (ddd, 1H, *J* = 7.9, 1.7, 1.1 Hz), 7.43 (ddd, 1H, *J* = 7.9, 2.0, 1.1 Hz), 7.28 (t, 1H, *J* = 7.9 Hz), 5.65 (hept, 1H, *J* = 1.7 Hz), 4.93 (m, 1H), 4.61 (m, 1H), 4.42 (m, 1H), 4.13 (s, 1H), 2.81 (m, 1H), 2.65 (dq, 1H, *J* = 16.5, 2.6 Hz), 1.63 (t, 3H, *J* = 2.6 Hz), 1.28 (dt, 3H, *J* = 2.2, 1.7 Hz); ^{13}C{1H} NMR (75 MHz, acetone-d_6, 25 °C): δ 147.7, 136.5,

130.4, 130.3, 130.2, 126.1, 125.5, 122.3, 92.5, 78.8, 77.7, 76.0, 75.0, 30.9, 14.9, 3.3; IR ($CHCl_3$): ν 1083, 2124 cm^{-1}; HRMS (ESI) *m/z*: $[M-H]^-$ calcd for $C_{16}H_{16}BrO_2$: 319.0339; found 319.0335.

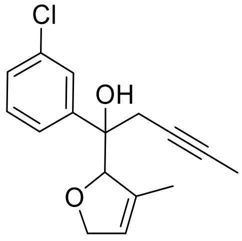

6l

Dihydrofuran 6l. From 29 mg (0.10 mmol) of diol **3l**, and after column chromatography using ethyl acetate/hexanes (1:10) as eluent, gave compound **6l** (11.2 mg, 38%) as a yellow oil; ^1H NMR (300 MHz, acetone-d_6, 25 °C): δ 7.68 (m, 1H), 7.57 (m, 1H), 7.35 (td, 1H, *J* = 7.8, 0.5 Hz), 7.28 (ddd, 1H, *J* = 7.9, 2.1, 1.3 Hz), 5.65 (hept, 1H, *J* = 1.6 Hz), 4.94 (dddt, 1H, *J* = 5.5, 3.0, 2.1, 1.0 Hz), 4.61 (m, 1H), 4.42 (ddt, 1H, *J* = 12.3, 3.5, 2.0 Hz), 4.12 (s, 1H), 2.83 (m, 1H), 2.66 (dq, 1H, *J* = 16.5, 2.6 Hz), 1.62 (t, 3H, *J* = 2.6 Hz), 1.28 (m, 3H); $^{13}C\{1H\}$ NMR (75 MHz, acetone-d_6, 25 °C): δ 147.5, 136.5, 134.0, 129.9, 127.4, 127.3, 125.7, 125.4, 92.5, 78.8, 77.7, 76.0, 75.0, 30.9, 14.9, 3.3; IR ($CHCl_3$): ν 1080, 2120 cm^{-1}; HRMS (ESI) *m/z*: $[M+H]^+$ calcd for $C_{16}H_{18}ClO_2$: 277.0990; found 277.0996.

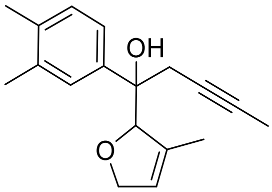

6m

Dihydrofuran 6m. From 40 mg (0.16 mmol) of diol **3m**, and after column chromatography using ethyl acetate/hexanes (1:10) as eluent, gave compound **6m** (10.1 mg, 25%) as a yellow oil; ^1H NMR (300 MHz, acetone-d_6, 25 °C): δ 7.39 (d, 1H, *J* = 2.0 Hz), 7.31 (dd, 1H, *J* = 7.9, 2.1 Hz), 7.06 (d, 1H, *J* = 7.9 Hz), 5.59 (hept, *J* = 1.7 Hz), 4.95 (dddd, 1H, *J* = 5.6, 3.2, 2.0, 1.0 Hz), 4.59 (dddd, 1H, *J* = 12.0, 5.9, 2.2, 1.6 Hz), 4.41 (ddt, 1H, *J* = 12.2, 3.4, 2.0 Hz), 3.70 (s, 1H), 2.79 (m,

1H), 2.62 (dq, 1H, J = 16.3, 2.6 Hz), 2.25 (s, 3H), 2.23 (s, 3H), 1.63 (t, 3H, J = 2.6 Hz), 1.21 (qd, 3H, J = 2.2, 1.1 Hz); ^{13}C{1H} NMR (75 MHz, acetone-d$_6$, 25 ^{o}C): δ 142.2, 137.1, 135.9, 135.2, 129.5, 128.2, 124.9, 124.5, 92.6, 78.2, 77.6, 76.7, 74.9, 31.4, 20.1, 19.4, 14.9, 3.4; IR ($CHCl_3$): ν 1079, 2122 cm^{-1}; HRMS (ESI) *m/z*: $[M+Na]^+$ calcd for $C_{18}H_{22}NaO_2$: 293.1512; found 293.1501.

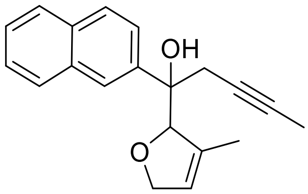

6p

Dihydrofuran 6p. From 30 mg (0.10 mmol) of diol **3p**, and after column chromatography using ethyl acetate/hexanes (1:10) as eluent, gave compound **6p** (13.9 mg, 46%) as a yellow oil; ^{1}H NMR (300 MHz, acetone-d$_6$, 25 ^{o}C): δ 8.15 (s, 1H), 7.90 (m, 2H), 7.85 (s, 1H), 7.80 (dd, 1H, J = 8.7, 1.8 Hz), 7.48 (m, 2H), 5.63 (hept, 1H , J = 1.7 Hz), 5.10 (dddd, 1H, J = 5.6, 3.3, 2.0, 1.0 Hz), 4.64 (dddd, 1H, J = 12.1, 5.8, 2.2, 1.6 Hz), 4.44 (m, 1H), 4.04 (s, 1H), 2.97 (dq, 1H, J = 16.4, 2.6 Hz), 2.77 (m, 1H), 1.58 (t, 3H, J = 2.6 Hz), 1.19 (m, 3H); ^{13}C{1H} NMR (75 MHz, acetone-d$_6$, 25 ^{o}C): δ 142.5, 136.9, 134.0, 133.4, 129.1, 128.3, 127.6, 126.6, 126.4, 125.9, 125.7, 125.2, 92.7, 78.5, 78.1, 76.4, 75.0, 31.3, 14.9, 3.3; IR ($CHCl_3$): ν 1080, 2124 cm^{-1}; HRMS (ESI) *m/z*: $[M+Na]^+$ calcd for $C_{20}H_{20}NaO_2$: 315.1356; found 315.1349.

General procedure for the synthesis of bridged ketals 8. Au/TiO_2 (0.2 g, 0.10 mmol; 1.0 mol % in Au) was added to a solution of the appropriate diol **3** (1.0 mmol) in DCE (10 mL) under argon atmosphere. Then, the reaction was heated at 90 ^{o}C in a sealed tube until disappearance of the starting material (TLC). Next, the mixture was filtered through a celite pad, washed with DCM, and the solvent was evaporated under reduced pressure. The crude reaction was purified by column chromatography using ethyl acetate/hexanes mixtures. Spectroscopic and analytical data for compounds **8a–p** follow.

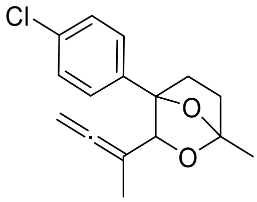

8a

Bridged ketal 8a. From 36 mg (0.13 mmol) of diol **3a**, and after column chromatography using ethyl acetate/hexanes (1:20) as eluent, gave compound **8a** (12 mg, 33%) as a yellow oil; ^{1}H NMR (300 MHz, acetone-d$_6$, 25 ^{o}C): δ 7.40 (s, 4H), 4.61 (dqd, 1H, *J* = 10.4, 3.2, 1.0 Hz), 4.56 (s, 1H), 4.43 (dqd, 1H, *J* = 10.2, 3.3, 1.1 Hz), 2.38 (ddd, 1H, *J* = 11.5, 8.8, 3.9 Hz), 2.06 (m, 1H), 1.92 (td, 1H, *J* = 11.8, 3.9 Hz), 1.77 (m, 1H), 1.67 (s, 3H), 1.28 (t, 3H, *J* = 3.2 Hz); ^{13}C{1H} NMR (75 MHz, acetone-d$_6$, 25 ^{o}C): δ 209.2, 138.3, 133.4, 128.9 (2C), 128.0 (2C), 109.2, 98.9, 90.7, 85.8, 74.7, 38.2, 37.7, 18.7, 14.3; IR (CHCl$_3$): ν 1003, 1955 cm^{-1}; HRMS (ESI) *m/z*: [M+H]$^+$ calcd for $C_{16}H_{18}ClO_2$: 277.0990; found 277.0983.

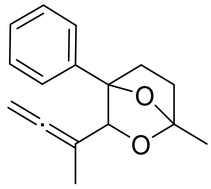

8b

Bridged ketal 8b. From 30 mg (0.12 mmol) of diol **3b**, and after column chromatography using ethyl acetate/hexanes (1:20) as eluent, gave compound **8b** (8.5 mg, 28%) as a yellow oil; ^{1}H NMR (300 MHz, acetone-d$_6$, 25 ^{o}C): δ 7.33 (m, 5H), 4.59 (m, 1H), 4.55 (s, 1H), 4.39 (dqd, 1H, *J* = 8.8, 3.2, 1.6 Hz), 2.37 (ddd, 1H, *J* = 11.5, 8.8, 3.8 Hz), 2.05 (m, 1H), 1.90 (td, 1H, *J* = 11.7, 3.8 Hz), 1.77 (m, 1H), 1.67 (s, 3H), 1.27 (t, 3H, *J* = 3.2 Hz); ^{13}C{1H} NMR (75 MHz, acetone-d$_6$, 25 ^{o}C): δ 209.3, 139.4, 128.7 (2C), 128.0, 126.2 (2C), 109.2, 99.2, 91.1, 86.0, 74.4, 38.2, 37.9, 18.8, 14.3; IR (CHCl$_3$): ν 1002, 1955 cm^{-1}; HRMS (ESI) *m/z*: [M+H]$^+$ calcd for $C_{16}H_{19}O_2$: 243.1380; found 243.1371.

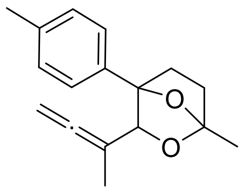

8c

Bridged ketal 8c. From 25 mg (0.11 mmol) of diol **3c**, and after column chromatography using ethyl acetate/hexanes (1:20) as eluent, gave compound **8c** (5.2 mg, 21%) as a yellow oil; ^{1}H NMR (300 MHz, CD_3CN, 25 °C): δ 7.22 (d, 2H, J = 8.5 Hz), 7.18 (d, 2H, J = 8.5 Hz), 4.61 (m, 1H), 4.49 (s, 1H), 4.43 (m, 1H), 2.33 (s, 3H), 2.28 (m, 1H), 2.03 (m, 1H), 1.86 (m, 1H), 1.73 (m, 1H), 1.66 (s, 3H), 1.25 (t, 3H, J = 3.2 Hz); ^{13}C{1H} NMR (75 MHz, CD_3CN, 25 °C): δ 209.2, 137.9, 136.4, 129.6 (2C), 126.2 (2C), 109.2, 99.3, 91.1, 86.0, 74.7, 38.2, 38.0, 21.2, 18.8, 14.5; IR ($CHCl_3$): ν 1003, 1954 cm^{-1}; HRMS (ESI) *m/z*: $[M+H]^+$ calcd for $C_{17}H_{21}O_2$: 257.1536; found 257.1530.

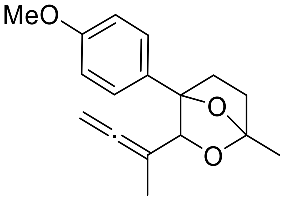

8d

Bridged ketal 8d. From 36 mg (0.13 mmol) of diol **3d**, and after column chromatography using ethyl acetate/hexanes (1:20) as eluent, gave compound **8d** (10.0 mg, 28%) as a yellow oil; ^{1}H NMR (300 MHz, acetone-d_6, 25 °C): δ 7.29 (d, 2H, J = 8.9 Hz), 6.91 (d, 2H, J = 8.9 Hz), 4.59 (dqd, 1H, J = 10.4, 3.2, 1.0 Hz), 4.49 (s, 1H), 4.42 (dqd, 1H, J = 10.0, 3.2, 1.2 Hz), 3.79 (s, 3H), 2.32 (ddd, 1H, J = 11.5, 8.8, 3.9 Hz), 2.05 (m, 1H), 1.89 (td, 1H, J = 11.8, 3.9 Hz), 1.76 (m, 1H), 1.65 (s, 3H), 1.27 (t, 3H, J = 3.2 Hz); ^{13}C{1H} NMR (75 MHz, acetone-d_6, 25 °C): δ 209.3, 159.9, 131.4, 127.4 (2C), 114.2 (2C), 108.9, 99.4, 90.9, 86.1, 74.6, 55.5, 38.3, 38.0, 18.9, 14.5; IR ($CHCl_3$): ν 1002, 1955 cm^{-1}; HRMS (ESI) *m/z*: $[M+H]^+$ calcd for $C_{17}H_{21}O_3$: 273.1485; found 273.1490.

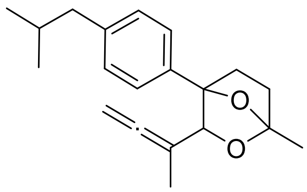

8f

Bridged ketal 8f. From 40 mg (0.13mmol) of diol **3f**, and after column chromatography using ethyl acetate/hexanes (1:20) as eluent, gave compound **8f** (12.4 mg, 36%) as a yellow oil; ^{1}H NMR (700 MHz, acetone-d$_6$, 25 ^{o}C): δ 7.28 (d, 2H, *J* = 8.2 Hz), 7.15 (d, 2H, *J* = 8.1 Hz), 4.57 (m, 1H), 4.49 (s, 1H), 4.37 (dtd, 1H, *J* = 9.9, 3.2, 1.9 Hz), 2.48 (dd, 2H, *J* = 7.3, 2.1 Hz), 2.35 (m, 1H), 2.05 (m,1H), 1.88 (m, 2H), 1.77 (td, 1H, *J* = 12.0, 4.9 Hz), 1.66 (s, 3H), 1.26 (t, 3H, *J* = 3.2 Hz), 0.88 (m, 6H); ^{13}C{1H} NMR (175 MHz, acetone-d$_6$, 25 ^{o}C): δ 209.2, 141.3, 136.7, 129.4 (2C), 126.0 (2C), 108.9, 99.3, 91.1, 86.0, 74.5, 45.6, 38.2, 37.7, 31.0, 22.6, 22.5, 18.8, 14.5; IR ($CHCl_3$): ν 1001, 1954 cm^{-1}; HRMS (ESI) *m/z*: [M+H]$^+$ calcd for $C_{20}H_{27}O_2$: 299.2006; found 299.2011.

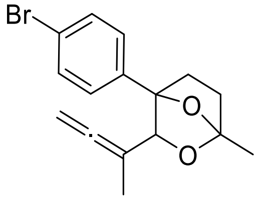

8g

Bridged ketal 8g. From 38 mg (0.12 mmol) of diol **3g**, and after column chromatography using ethyl acetate/hexanes (1:20) as eluent, gave compound **8g** (12.4 mg, 32%) as a yellow oil; ^{1}H NMR (300 MHz, acetone-d$_6$, 25 ^{o}C): δ 7.55 (d, 2H, *J* = 8.6 Hz), 7.34 (d, 2H, *J* = 8.7 Hz), 4.61 (dqd, 1H, *J* = 10.4, 3.2, 0.9 Hz), 4.56 (s, 1H), 4.43 (dqd, 1H, *J* =10.2, 3.2, 1.1 Hz), 2.38 (ddd, 1H, *J* = 11.5, 8.8, 3.9 Hz), 2.08 (m, 1H), 1.92 (td, 1H, *J* = 11.8, 3.9 Hz), 1.76 (td, 1H, *J* = 12.0, 4.9 Hz), 1.67 (s, 3H), 1.29 (t, 3H, *J* = 3.2 Hz); ^{13}C{1H} NMR (75 MHz, acetone-d$_6$, 25 ^{o}C): δ 209.2, 138.8, 131.8 (2C), 128.3 (2C), 121.5, 109.2, 98.9, 90.7, 85.8, 74.7, 38.2, 37.7, 18.7,14.3; IR (C HCl_3): ν 1004, 1956 cm^{-1}; HRMS (ESI) *m/z*: [M+H]$^+$ calcd for $C_{16}H_{18}BrO_2$: 321.0485; found 321.0492.

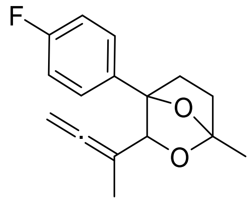

8i

Bridged ketal 8i. From 30 mg (0.12 mmol) of diol **3i**, and after column chromatography using ethyl acetate/hexanes (1:20) as eluent, gave compound **8i** (10 mg, 33%) as a yellow oil; ^{1}H NMR (700 MHz, acetone-d$_6$, 25 °C): δ 7.41 (dd, 2H, *J* = 8.8, 5.4 Hz), 7.13 (t, 2H, *J* = 9.0 Hz), 4.60 (dqd, 1H, *J* = 10.3, 3.3, 1.0 Hz), 4.54 (s, 1H), 4.42 (dqd, 1H, *J* = 9.9, 3.4, 1.0 Hz), 2.37 (ddd, 1H, *J* = 11.9, 8.9, 4.1 Hz), 2.07 (m, 1H), 1.91 (td, 1H, *J* = 12.0, 4.1 Hz), 1.77 (td, 1H, *J* = 12.0, 5.0 Hz), 1.67 (s, 3H), 1.28 (t, 3H, *J* = 3.2 Hz); ^{13}C{1H} NMR (175 MHz, acetone-d$_6$, 25 °C): δ 209.2, 162.8 (d, 1C, *J* = 243.5 Hz), 135.5, 128.2 (d, 2C, *J* = 7.6 Hz), 115.5 (d, 2C, *J* = 21.9 Hz), 109.1, 99.0, 90.7, 85.9, 74.6, 38.2, 37.8, 18.7, 14.4; IR (CHCl$_3$): ν 1003, 1953 cm^{-1}; HRMS (ESI) *m/z*: [M+H]$^+$ calcd for $C_{16}H_{18}FO_2$: 261.1285; found 261.1290.

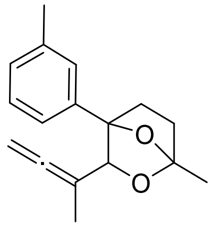

8j

Bridged ketal 8j. From 30 mg (0.12 mmol) of diol **3j**, and after column chromatography using ethyl acetate/hexanes (1:20) as eluent, gave compound **8j** (8.4 mg, 28%) as a yellow oil; ^{1}H NMR (300 MHz, acetone-d$_6$, 25 °C): δ 7.16 (m, 4H), 4.59 (dqd, 1H, *J* = 10.3, 3.2, 0.9 Hz), 4.55 (s, 1H), 4.40 (dqd, 1H, *J* = 10.1, 3.2, 1.1 Hz), 2.33 (m, 4H), 2.05 (m, 1H), 1.89 (td, 1H, *J* = 11.7, 3.9 Hz), 1.75 (tdd, 1H, *J* = 12.0, 4.8, 0.6 Hz), 1.66 (s, 3H), 1.28 (t, 3H, *J* = 3.2 Hz); ^{13}C{1H} NMR (75 MHz, acetone-d$_6$, 25 °C): δ 209.3, 139.4, 138.1, 128.6, 126.7, 123.2, 108.9, 99.2, 91.1, 86.0, 74.4, 38.2, 38.1, 21.5, 18.8, 14.3; IR (CHCl$_3$): ν 1002, 1952 cm^{-1}; HRMS (ESI) *m/z*: [M+H]$^+$ calcd for $C_{17}H_{21}O_2$: 257.1536; found 257.1544.

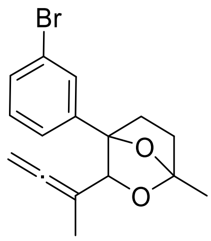

8k

Bridged ketal 8k. From 20 mg (0.06 mmol) of diol **3k**, and after column chromatography using ethyl acetate/hexanes (1:20) as eluent, gave compound **8k** (5 mg, 25%) as a yellow oil; ^{1}H NMR (300 MHz, acetone-d$_6$, 25 °C): δ 7.55 (m, 1H), 7.48 (dt, 1H, *J* = 7.5, 1.7 Hz), 7.36 (m, 2H), 4.62 (m, 2H), 4.44 (m, 1H), 2.41 (ddd, 1H, *J* = 11.5, 8.8, 3.8 Hz), 2.10 (m, 1H), 1.93 (td, 1H, *J* = 11.7, 3.8 Hz), 1.80 (m, 1H), 1.68 (s, 3H), 1.30 (t, 3H, *J* = 3.2 Hz); ^{13}C{1H} NMR (75 MHz, acetone-d$_6$, 25 °C): δ 209.3, 142.0, 131.1, 130.9, 129.2, 125.2, 122.4, 109.3, 98.8, 90.6, 85.7, 74.7, 38.1, 37.7, 18.7, 14.3; IR ($CHCl_3$): ν 1003, 1956 cm^{-1}; HRMS (ESI) *m/z*: [M+H]$^+$ calcd for $C_{16}H_{18}BrO_2$: 321.0485; found 321.0480.

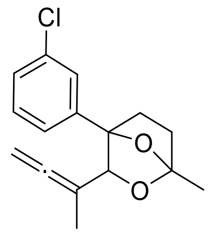

8l

Bridged ketal 8l. From 40 mg (0.14 mmol) of diol **3l**, and after column chromatography using ethyl acetate/hexanes (1:20) as eluent, gave compound **8l** (10.7 mg, 27%) as a yellow oil; ^{1}H NMR (300 MHz, acetone-d$_6$, 25 °C): δ 7.36 (m, 4H), 4.61 (m, 2H), 4.42 (dtt, 1H, *J* = 8.8, 3.2, 1.7 Hz), 2.41 (ddd, 1H, *J* = 11.5, 8.8, 3.8 Hz), 2.09 (m, 1H), 1.93 (td, 1H, *J* = 11.7, 3.8 Hz), 1.79 (m, 1H), 1.68 (s, 3H), 1.30 (t, 3H, *J* = 3.2 Hz); ^{13}C{1H} NMR (75 MHz, acetone-d$_6$, 25 °C): δ 209.3, 141.7, 134.2, 130.6, 128.1, 126.3, 124.8, 109.3, 98.9, 90.7, 85.7, 74.7, 38.1, 37.7, 18.7, 14.4; IR ($CHCl_3$): ν 1002, 1956 cm^{-1}; HRMS (ESI) *m/z*: [M+H]$^+$ calcd for $C_{16}H_{18}ClO_2$: 277.0990; found 277.0994.

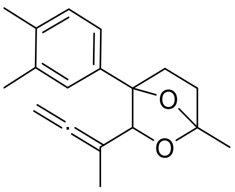

8m

Bridged ketal 8m. From 40 mg (0.15 mmol) of diol **3m**, and after column chromatography using ethyl acetate/hexanes (1:20) as eluent, gave compound **8m** (8.1 mg, 21%) as a yellow oil; ^{1}H NMR (300 MHz, acetone-d$_6$, 25 °C): δ 7.10 (m, 3H), 4.58 (m, 1H), 4.53 (s, 1H), 4.40 (dqd, 1H, *J* = 10.0, 3.2, 1.1 Hz), 2.31 (ddd, 1H, *J* = 8.6, 7.4, 4.3 Hz), 2.25 (s, 3H), 2.23 (s, 3H), 2.06 (m, 1H), 1.88 (td, 1H, *J* = 11.8, 3.9 Hz), 1.72 (td, 1H, *J* = 11.8, 5.0 Hz), 1.66 (s, 3H), 1.28 (t, 3H, *J* = 3.2 Hz); ^{13}C{1H} NMR (75 MHz, acetone-d$_6$, 25 °C): δ 209.4, 136.9, 136.6, 136.0, 129.9, 127.3, 123.6, 108.8, 99.3, 91.0, 86.0, 74.3, 38.3, 38.2, 19.8, 19.5, 18.8, 14.4; IR (CHCl$_3$): ν 1004, 1954 cm^{-1}; HRMS (ESI) *m/z*: [M+H]$^+$ calcd for $C_{18}H_{23}O_2$: 271.1693; found 271.1700.

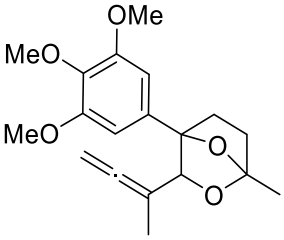

8n

Bridged ketal 8n. From 34.5 mg (0.10 mmol) of diol **3n**, and after column chromatography using ethyl acetate/hexanes (1:8) as eluent, gave compound **8n** (14.3 mg, 41%) as a yellow oil; ^{1}H NMR (300 MHz, acetone-d$_6$, 25 °C): δ 6.66 (s, 2H), 4.62 (dqd, 1H, *J* = 10.4, 3.2, 0.9 Hz), 4.54 (s, 1H), 4.45 (dqd, 1H, *J* = 10.1, 3.2, 1.1 Hz), 3.81 (s, 6H), 3.72 (s, 3H), 2.36 (ddd, 1H, *J* = 11.5, 8.8, 3.7 Hz), 2.06 (m, 1H), 1.90 (td, 1H, *J* = 11.6, 3.7), 1.78 (m, 1H), 1.66 (s, 3H), 1.33 (t, 3H, *J* = 3.2 Hz); ^{13}C{1H} NMR (75 MHz, acetone-d$_6$, 25 °C): δ 208.5, 153.2 (2C), 133.9, 108.1, 102.9 (2C), 98.4, 90.2, 85.0, 73.5, 59.6, 55.6 (2C), 37.2, 37.0, 17.9, 13.5; IR (CHCl$_3$): ν 1005, 1957 cm^{-1}; HRMS (ESI) *m/z*: [M+H]$^+$ calcd for $C_{19}H_{25}O_5$: 333.1697; found 333.1697.

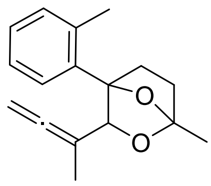

8o

Bridged ketal 8o. From 50 mg (0.2 mmol) of diol **3o**, and after column chromatography using ethyl acetate/hexanes (1:8) as eluent, gave compound **8o** (14 mg, 28%) as a yellow oil; ^{1}H NMR (300 MHz, acetone-d_6, 25 ^{o}C): δ 7.64 (d, 1H, *J* = 6.7 Hz), 7.17 (m, 3H), 4.82 (s, 1H), 4.61 (dqd, 1H, *J* = 10.2, 3.2, 0.7 Hz), 4.41 (m, 1H), 2.56 (m, 1H), 2.38 (s, 3H), 2.11 (m, 1H), 1.91 (td, 1H, *J* = 11.9, 4.1 Hz), 1.68 (m, 4H), 1.34 (t, 3H, *J* = 3.2 Hz); $^{13}C\{1H\}$ NMR (75 MHz, acetone-d_6, 25 ^{o}C): δ 207.8, 137.6, 134.3, 131.2, 127.7, 125.9, 125.7, 106.8, 98.4, 91.5, 83.7, 74.1, 37.7, 35.2, 21.2, 18.3, 13.3; IR ($CHCl_3$): ν 1004, 1956 cm^{-1}; HRMS (ESI) *m/z*: $[M+H]^+$ calcd for $C_{17}H_{21}O_2$: 257.1536; found 257.1532.

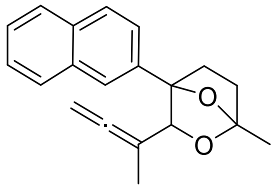

8p

Bridged ketal 8p. From 31 mg (0.11 mmol) of diol **3p**, and after column chromatography using ethyl acetate/hexanes (1:20) as eluent, gave compound **8p** (12 mg, 40%) as a yellow oil; ^{1}H NMR (300 MHz, acetone-d_6, 25 ^{o}C): δ 7.92 (m, 4H), 7.51 (m, 3H), 4.69 (s, 1H), 4.60 (dq, 1H, *J* = 9.7, 3.1 Hz), 4.36 (m, 1H), 2.47 (ddd, 1H, *J* = 12.0, 8.9, 4.0 Hz), 2.11 (m, 1H), 1.96 (td, 1H, *J* = 12.0, 4.0 Hz), 1.85 (td, 1H, *J* = 11.9, 4.9 Hz), 1.72 (s, 3H), 1.29 (t, 3H, *J* = 3.1 Hz); $^{13}C\{1H\}$ NMR (75 MHz, acetone-d_6, 25 ^{o}C): δ 209.2, 137.0, 134.0, 133.6, 128.8, 128.5, 128.3, 127.1, 126.7, 124.7, 124.5, 109.1, 99.1, 91.3, 85.9, 74.6, 38.2, 38.0, 18.8, 14.3; IR ($CHCl_3$): ν 1004, 1955 cm^{-1}; HRMS (ESI) *m/z*: $[M+H]^+$ calcd for $C_{20}H_{21}O_2$: 293.1536; found 293.1540.

2i

R_1=3.59%

Crystal Data and Experimental

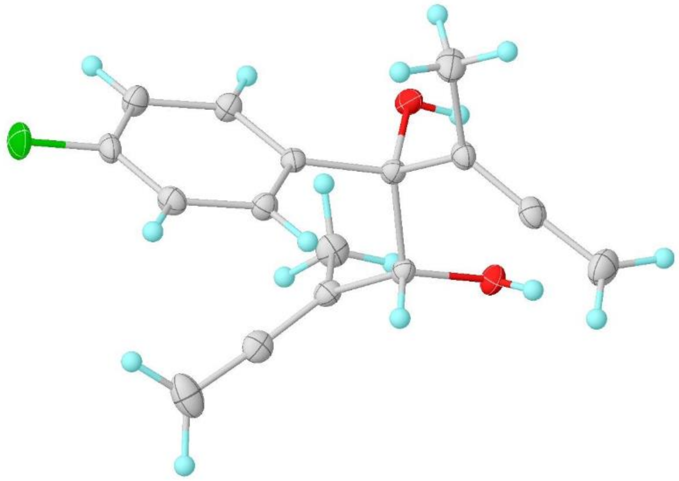

ORTEP drawing of **2i** showing thermal ellipsoids at the 50% probability level.

**Compound**	**2i**
Formula	$C_{16}H_{17}FO_2$
$D_{calc.}$/ g cm^{-3}	1.280
μ/mm^{-1}	0.757
Formula Weight	260.29
Colour	colourless
Shape	block
Size/mm^3	0.18×0.12×0.09
T/K	100.1(5)
Crystal System	monoclinic
Space Group	$P2_1/c$
a/Å	13.59295(12)
b/Å	5.72295(5)
c/Å	18.30481(15)
α/°	90
β/°	108.5081(9)
γ/°	90
V/Å^3	1350.31(2)
Z	4
Z'	1
Wavelength/Å	1.54184
Radiation type	Cu K$_\alpha$
Θ_{min}/°	3.429
Θ_{max}/°	78.044
Measured Refl.	30324
Independent Refl.	2882
Reflections with I > 2(I)	2812
R_{int}	0.0342
Parameters	198
Restraints	0
Largest Peak	0.247
Deepest Hole	-0.270
GooF	1.057
wR_2 (all data)	0.0902
wR_2	0.0898
R_1 (all data)	0.0364
R_1	0.0359

Experimental. Single colourless block-shaped crystals of **2i** were obtained from slow evaporation of dichloromethane-hexane (1:10) mixture at room temperature. A suitable crystal 0.18×0.12×0.09 mm^3 was selected and mounted on a MiTeGEN Dual Thickness MicroLoops in perfluoropolyether oil on an XtaLAB Synergy R, HyPix diffractometer. The crystal was kept at a steady T = 100.1(5) K during data collection. The structure was solved with the ShelXT (Sheldrick, 2015) structure solution program using the Intrinsic Phasing solution method and by using **Olex2** (Dolomanov et al., 2009) as the graphical interface. The model was refined with version 2018/3 of ShelXL 2018/3 (Sheldrick, 2015) using Least Squares minimisation.

Crystal Data. $C_{16}H_{17}FO_2$, M_r = 260.29, monoclinic, $P2_1/c$ (No. 14), a = 13.59295(12) Å, b = 5.72295(5) Å, c = 18.30481(15) Å, β = 108.5081(9)°, α = γ = 90°, V = 1350.31(2) Å^3, T = 100.1(5) K, Z = 4, Z' = 1, μ(Cu K$_\alpha$) = 0.757, 30324 reflections measured, 2882 unique (R_{int} = 0.0342) which were used in all calculations. The final wR_2 was 0.0902 (all data) and R_1 was 0.0359 (I > 2(I)).

Structure Quality Indicators

**Reflections:**	d min (Cu\a) 2Θ=156.7° 0.79	I/σ(I) 71.5	Rint 3.42%	Full 135.4° 99% to 156.7° 100
**Refinement:**	Shift -0.001	Max Peak 0.2	Min Peak -0.3	GooF 1.057

Experimental Extended. A colourless block-shaped crystal with dimensions 0.18×0.12×0.09 mm^3 was mounted on a MiTeGEN Dual Thickness MicroLoops in perfluoropolyether oil. Data were collected using an XtaLAB Synergy R, HyPix diffractometer operating at T = 100.1(5) K.

Data were measured using ω scans of 0.5° per frame for 0.1/0.3 s using Cu K$_\alpha$ radiation. The diffraction pattern was indexed and the total number of runs and images was based on the strategy calculation from the program CrysAlisPro (Rigaku, V1.171.42.84a, 2023) The maximum resolution that was achieved was Θ = 78.044° (0.79 Å).

The diffraction pattern was indexed The diffraction pattern was indexed and the total number of runs and images was based on the strategy calculation from the program CrysAlisPro (Rigaku, V1.171.42.84a, 2023) and the unit cell was refined using CrysAlisPro (Rigaku, V1.171.42.84a, 2023) on 23599 reflections, 78% of the observed reflections.

Data reduction, scaling and absorption corrections were performed using CrysAlisPro (Rigaku, V1.171.42.84a, 2023). The final completeness is 100.00 % out to 78.044° in Θ. A multi-scan absorption correction was performed using CrysAlisPro 1.171.42.84a (Rigaku Oxford Diffraction, 2023) using spherical harmonics,implemented in SCALE3 ABSPACK scaling algorithm. The absorption coefficient μ of this material is 0.757 mm^{-1} at this wavelength (λ = 1.542Å) and the minimum and maximum transmissions are 0.427 and 1.000.

The structure was solved and the space group $P2_1/c$ (# 14) determined by the ShelXT (Sheldrick, 2015) structure solution program using Intrinsic Phasing and refined by Least Squares using version 2018/3 of ShelXL 2018/3 (Sheldrick, 2015). All non-hydrogen atoms were refined anisotropically. Hydrogen atom positions were calculated geometrically and refined using the riding model. Most hydrogen atom positions were calculated geometrically and refined using the riding model, but some hydrogen atoms were refined freely.

_exptl_absorpt_process_details: CrysAlisPro 1.171.42.84a (Rigaku Oxford Diffraction, 2023) using spherical harmonics,implemented in SCALE3 ABSPACK scaling algorithm.

Table S4: Fractional Atomic Coordinates (×10[4]) and Equivalent Isotropic Displacement Parameters (Å[2]×10[3]) for **2i**. U_{eq} is defined as 1/3 of the trace of the orthogonalised U_{ij}.

Atom	x	y	z	U_{eq}
C1	8015.3(7)	1004.9(17)	8941.5(6)	14.4(2)
C2	8451.0(7)	2750.8(17)	9620.9(5)	14.5(2)
C3	8314.6(7)	1689.2(19)	8227.1(6)	16.6(2)
C4	8985.4(8)	3331(2)	8232.9(6)	19.4(2)
C5	9679.3(9)	4905(2)	8238.5(7)	27.0(3)
C6	7815.1(8)	266(2)	7507.5(6)	24.6(2)
C7	8064.6(8)	2258.3(18)	10299.5(6)	16.1(2)
C8	7321.8(8)	3571(2)	10384.1(6)	20.1(2)
C9	6545.5(10)	4819(3)	10426.1(7)	30.3(3)
C10	8512.5(9)	247(2)	10837.6(6)	22.4(2)
C11	6837.0(7)	911.5(17)	8738.8(5)	14.2(2)
C12	6231.9(8)	2775.6(18)	8351.6(6)	16.6(2)
C13	5159.1(8)	2779.6(19)	8199.2(6)	18.1(2)
C14	4716.8(8)	894.1(19)	8444.2(6)	17.3(2)
C15	5285.7(8)	-987.3(19)	8823.9(6)	19.2(2)
C16	6356.4(8)	-966.6(18)	8970.3(6)	17.2(2)
F1	3667.6(5)	855.0(12)	8291.5(4)	24.38(17)
O1	8406.6(6)	-1286.6(12)	9170.5(4)	17.05(17)
O2	9566.6(5)	2630.3(13)	9895.6(4)	17.21(17)

Table S5: Anisotropic Displacement Parameters (×10[4]) **2i.** The anisotropic displacement factor exponent takes the form: $-2\pi^2[h^2a^{*2} \times U_{11} + \ldots + 2hka^* \times b^* \times U_{12}]$

Atom	U_{11}	U_{22}	U_{33}	U_{23}	U_{13}	U_{12}
C1	13.7(4)	12.7(4)	16.5(4)	0.5(4)	4.1(4)	0.3(3)
C2	12.1(4)	14.1(4)	16.3(4)	0.3(4)	3.2(4)	-0.3(3)
C3	12.7(4)	20.3(5)	16.7(5)	-1.0(4)	4.5(4)	1.2(4)
C4	18.3(5)	25.4(5)	15.6(4)	-0.2(4)	6.7(4)	1.5(4)
C5	26.4(6)	30.7(6)	26.5(6)	0.0(5)	11.9(5)	-7.8(5)
C6	21.9(5)	34.1(6)	18.5(5)	-6.5(5)	7.4(4)	-4.8(5)
C7	15.5(4)	16.9(5)	14.6(4)	-0.7(4)	2.8(4)	-2.0(4)
C8	22.6(5)	23.6(5)	14.3(4)	2.1(4)	6.0(4)	-1.8(4)
C9	30.3(6)	39.0(7)	26.2(6)	9.3(5)	15.2(5)	11.8(5)
C10	24.8(5)	22.8(5)	19.7(5)	4.0(4)	7.1(4)	1.3(4)
C11	13.9(4)	15.7(5)	12.7(4)	-1.6(4)	3.7(3)	-0.6(4)
C12	17.0(5)	15.7(5)	17.2(4)	1.3(4)	5.5(4)	-0.7(4)
C13	17.1(5)	17.8(5)	18.3(5)	1.1(4)	4.1(4)	3.9(4)
C14	11.4(4)	23.0(5)	17.8(5)	-3.1(4)	5.0(4)	0.1(4)
C15	18.0(5)	19.2(5)	21.5(5)	1.7(4)	7.6(4)	-2.8(4)
C16	16.4(5)	16.3(5)	17.9(5)	2.3(4)	4.1(4)	0.3(4)
F1	11.5(3)	29.7(4)	32.0(4)	0.4(3)	6.9(2)	0.8(2)
O1	12.9(3)	13.4(3)	22.2(4)	-0.2(3)	1.9(3)	1.1(3)
O2	11.9(3)	19.6(4)	18.4(3)	0.9(3)	2.3(3)	-2.1(3)

Table S6: Bond Lengths in Å for **2i**.

Atom	Atom	Length/Å
C1	C2	1.5583(13)
C1	C3	1.5383(13)
C1	C11	1.5260(13)
C1	O1	1.4272(12)
C2	C7	1.5206(13)
C2	O2	1.4399(11)
C3	C4	1.3070(15)
C3	C6	1.5136(14)
C4	C5	1.3021(16)
C7	C8	1.3067(15)

Atom	Atom	Length/Å
C7	C10	1.5116(14)
C8	C9	1.2966(17)
C11	C12	1.3952(14)
C11	C16	1.3919(14)
C12	C13	1.3950(14)
C13	C14	1.3777(15)
C14	C15	1.3781(15)
C14	F1	1.3636(11)
C15	C16	1.3938(14)

Table S7: Bond Angles in ° for **2i**.

Atom	Atom	Atom	Angle/°
C3	C1	C2	112.28(8)
C11	C1	C2	108.67(8)
C11	C1	C3	110.03(8)
O1	C1	C2	110.06(8)
O1	C1	C3	108.01(8)
O1	C1	C11	107.68(8)
C7	C2	C1	113.01(8)
O2	C2	C1	109.83(8)
O2	C2	C7	107.72(7)
C4	C3	C1	123.24(9)
C4	C3	C6	120.86(9)
C6	C3	C1	115.86(9)
C5	C4	C3	177.82(12)
C8	C7	C2	118.06(9)
C8	C7	C10	121.81(9)
C10	C7	C2	120.09(9)
C9	C8	C7	176.27(12)
C12	C11	C1	120.05(9)
C16	C11	C1	120.72(9)
C16	C11	C12	119.15(9)
C13	C12	C11	120.87(9)
C14	C13	C12	118.04(9)
C13	C14	C15	122.90(9)
F1	C14	C13	118.82(9)
F1	C14	C15	118.26(9)
C14	C15	C16	118.34(9)
C11	C16	C15	120.69(9)

Table S8: Hydrogen Fractional Atomic Coordinates (×10^4) and Equivalent Isotropic Displacement Parameters (Å^2×10^3) for **2i**. U_{eq} is defined as 1/3 of the trace of the orthogonalised U_{ij}.

Atom	x	y	z	U_{eq}
H2A	8243.23	4337.84	9433.91	17
H5A	9460(12)	6560(30)	8096(9)	39(4)
H5B	10431(13)	4460(30)	8387(9)	42(4)
H6A	7815.26	-1354.98	7641.96	37
H6B	8199.36	467.78	7153.24	37
H6C	7113.99	784.17	7270.94	37
H9A	5809(13)	4270(30)	10139(10)	42(4)
H9B	6643(12)	6290(30)	10741(9)	41(4)
H10A	8317.58	-1202.84	10566.47	34
H10B	8249.17	294.36	11265.91	34
H10C	9255.02	372.56	11022.19	34
H12	6547.91	4032.02	8193.11	20
H13	4754.34	4018.47	7939.84	22
H15	4963.15	-2239.62	8978.68	23
H16	6753.86	-2220.87	9225.25	21
H1	9048(14)	-1180(30)	9362(10)	38(4)
H2	9786(13)	3410(30)	9592(10)	43(5)

Citations

O.V. Dolomanov and L.J. Bourhis and R.J. Gildea and J.A.K. Howard and H. Puschmann, Olex2: A complete structure solution, refinement and analysis program, *J. Appl. Cryst.*, (2009), **42**, 339-341.

Sheldrick, G.M. (2015). Acta Cryst. A71, 3-8.

Sheldrick, G.M. (2015). Acta Cryst. C71, 3-8.

3a-NO_2

R_1=4.69%

Crystal Data and Experimental

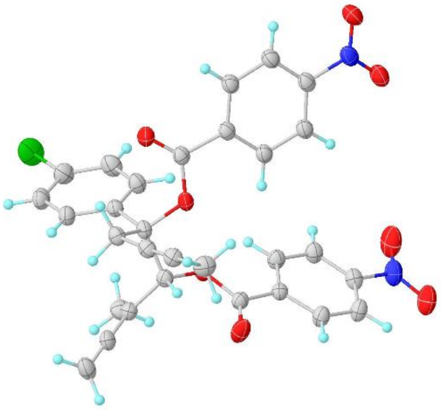

Experimental. Single colourless block-shaped crystals of **3a-NO_2** were obtained from slow evaporation of dichloromethane-hexane (1:10) mixture at room temperature. A suitable crystal 0.14×0.10×0.08 mm^3 was selected and mounted on a MiTeGEN Dual Thickness MicroLoops in perfluoropolyether oil on an XtaLAB Synergy R, HyPix diffractometer. The crystal was kept at a steady T = 100.0(4) K during data collection. The structure was solved with the ShelXT (Sheldrick, 2015) structure solution program using the Intrinsic Phasing solution method and by using **Olex2** (Dolomanov et al., 2009) as the graphical interface. The model was refined with version 2018/3 of ShelXL 2018/3 (Sheldrick, 2015) using Least Squares minimisation.

Crystal Data. $C_{30}H_{23}ClN_2O_8$, M_r = 574.95, triclinic, P-1 (No. 2), a = 8.38786(11) Å, b = 18.5874(3) Å, c = 18.6958(3) Å, α = 71.6639(17)°, β = 87.4407(12)°, γ = 86.9463(12)°, V = 2761.72(8) Å^3, T = 100.0(4) K, Z = 4, Z' = 2, μ(Cu K$_\alpha$) = 1.699, 60933 reflections measured, 11505 unique (R_{int} = 0.0506) which were used in all calculations. The final wR_2 was 0.1285 (all data) and R_1 was 0.0469 (I > 2(I)).

ORTEP drawing of **3a-NO_2** showing thermal ellipsoids at the 50% probability level.

**Compound**	**3a-NO_2**
Formula	$C_{30}H_{23}ClN_2O_8$
$D_{calc.}$/ g cm^{-3}	1.383
μ/mm^{-1}	1.699
Formula Weight	574.95
Colour	colourless
Shape	block
Size/mm^3	0.14×0.10×0.08
T/K	100.0(4)
Crystal System	triclinic
Space Group	P-1
a/Å	8.38786(11)
b/Å	18.5874(3)
c/Å	18.6958(3)
α/°	71.6639(17)
β/°	87.4407(12)
γ/°	86.9463(12)
V/Å^3	2761.72(8)
Z	4
Z'	2
Wavelength/Å	1.54184
Radiation type	Cu K$_\alpha$
Θ_{min}/°	2.491
Θ_{max}/°	78.337
Measured Refl.	60933
Independent Refl.	11505
Reflections with I > 2(I)	9570
R_{int}	0.0506
Parameters	759
Restraints	612
Largest Peak	0.336
Deepest Hole	-0.435
GooF	1.035
wR_2 (all data)	0.1285
wR_2	0.1198
R_1 (all data)	0.0569
R_1	0.0469

Structure Quality Indicators

**Reflections:**	d min (Cu\a) 2Θ=156.7° 0.79	I/σ(I) 24.9	Rint 5.06%	Full 135.4° 97% to 156.7° 99.7
**Refinement:**	Shift -0.001	Max Peak 0.3	Min Peak -0.4	GooF 1.035

Experimental Extended. A colourless block-shaped crystal with dimensions 0.14×0.10×0.08 mm³ was mounted on a MiTeGEN Dual Thickness MicroLoops in perfluoropolyether oil. Data were collected using an XtaLAB Synergy R, HyPix diffractometer operating at T = 100.0(4) K.

Data were measured using ω scans of 0.5° per frame for 0.1 s using Cu K_α radiation. The diffraction pattern was indexed and the total number of runs and images was based on the strategy calculation from the program CrysAlisPro (Rigaku, V1.171.42.84a, 2023) The maximum resolution that was achieved was Θ = 78.337° (0.79 Å).

The diffraction pattern was indexed The diffraction pattern was indexed and the total number of runs and images was based on the strategy calculation from the program CrysAlisPro (Rigaku, V1.171.42.84a, 2023) and the unit cell was refined using CrysAlisPro (Rigaku, V1.171.42.84a, 2023) on 32195 reflections, 53% of the observed reflections.

Data reduction, scaling and absorption corrections were performed using CrysAlisPro (Rigaku, V1.171.42.84a, 2023). The final completeness is 99.70 % out to 78.337° in Θ. A multi-scan absorption correction was performed using CrysAlisPro 1.171.42.84a (Rigaku Oxford Diffraction, 2023) using spherical harmonics, implemented in SCALE3 ABSPACK scaling algorithm. The absorption coefficient μ of this material is 1.699 mm^{-1} at this wavelength (λ = 1.542Å) and the minimum and maximum transmissions are 0.880 and 1.000.

The structure was solved and the space group P-1 (# 2) determined by the ShelXT (Sheldrick, 2015) structure solution program using Intrinsic Phasing and refined by Least Squares using version 2018/3 of ShelXL 2018/3 (Sheldrick, 2015). All non-hydrogen atoms were refined anisotropically. Hydrogen atom positions were calculated geometrically and refined using the riding model. Most hydrogen atom positions were calculated geometrically and refined using the riding model, but some hydrogen atoms were refined freely.

_exptl_absorpt_process_details: CrysAlisPro 1.171.42.84a (Rigaku Oxford Diffraction, 2023) using spherical harmonics, implemented in SCALE3 ABSPACK scaling algorithm.

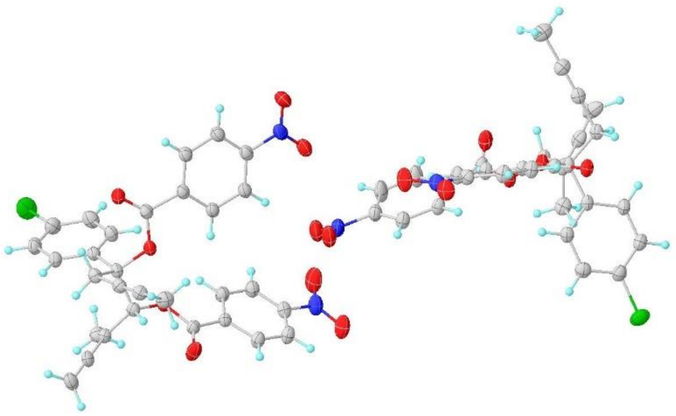

ORTEP diagram of the asymmetric unit showing two molecules of **3a-NO$_2$** and 50% probability ellipsoids.

Table S9: Fractional Atomic Coordinates (×10[4]) and Equivalent Isotropic Displacement Parameters (Å[2]×10[3]) for **3a-NO2**. U_{eq} is defined as 1/3 of the trace of the orthogonalised U_{ij}.

**Atom**	**x**	**y**	**z**	U_{eq}
C1	5177(2)	2877.6(11)	9115.4(10)	31.5(3)
C2	4398(3)	2865.6(12)	8475.6(12)	47.0(5)
C3	4030(3)	3534.2(12)	7917.4(12)	46.7(5)
C4	4500(3)	4201.9(11)	7999.1(11)	36.6(4)
C5	5314(2)	4232.7(11)	8613.2(11)	35.0(4)
C6	5620(2)	3560.3(11)	9187.2(11)	32.2(3)
C7	5602(2)	2131.5(10)	9676.5(10)	30.7(3)
C9	6702(2)	1466.9(10)	10837.0(10)	27.6(3)
C10	7816(2)	1618.2(9)	11410.9(10)	26.3(2)
C11	8858(2)	884.9(10)	11730.4(10)	28.4(3)
C12	9929(2)	719.2(10)	11156.4(11)	30.3(3)
C13	10797(2)	610.0(11)	10675.1(11)	34.4(4)
C14	11899(3)	496.9(14)	10084.7(13)	44.8(5)
C16	9942(2)	2486.4(10)	11260.6(10)	26.6(3)
C18	5329(2)	966.1(10)	11214.5(10)	29.6(3)
C19	5514(2)	244.2(11)	11292.9(11)	34.7(4)
C20	5728(3)	-468.4(13)	11341.4(15)	49.9(6)
C21	3806(2)	1322.7(12)	11439.5(12)	36.5(4)
C26	10655(2)	3177.1(10)	10714.2(10)	26.2(3)
C27	10011(2)	3534.0(10)	10012.2(10)	30.6(3)
C28	10644(2)	4197.5(11)	9538.8(11)	33.1(4)
C29	11906(2)	4489.6(10)	9794.9(11)	30.3(3)
C30	12584(2)	4142.9(11)	10485.1(11)	33.3(4)
C31	11949(2)	3475.6(10)	10950.3(11)	31.3(3)
C35	6918(2)	1874.4(9)	12018.8(10)	25.9(3)
C36	6575(2)	1361.2(10)	12724.4(10)	30.8(3)
C37	5650(2)	1583.9(11)	13260.0(11)	35.3(4)
C38	5077(2)	2326.7(11)	13084.8(11)	34.1(4)
C39	5421(2)	2855.7(11)	12392.2(12)	35.4(4)
C40	6335(2)	2624.4(10)	11861.7(11)	30.0(3)
Cl2	3907.5(7)	2605.8(3)	13758.2(3)	51.33(15)
N23	4157(2)	4913.5(10)	7391.6(10)	41.8(4)
N32	12554(2)	5206.8(9)	9307.1(10)	35.2(3)
O8	6104.7(15)	2186.9(7)	10334.2(7)	27.9(2)
O15	8790.1(15)	2229.5(7)	10938.8(7)	27.2(2)
O17	10352.3(15)	2214.3(7)	11904.0(7)	30.7(3)
O22	5527.5(19)	1536.5(8)	9551.3(8)	41.4(3)
O24	4981(2)	5455.8(9)	7335.4(9)	51.6(4)
O25	3071(2)	4923.7(9)	6967.9(9)	51.3(4)
O33	12121(2)	5453.1(9)	8654.3(9)	49.6(4)
O34	13497.3(18)	5524.4(8)	9578.5(9)	43.9(3)
C42	756(2)	7285.0(10)	5833.2(10)	27.0(3)
C43	593(2)	7199.9(11)	6599.7(11)	33.2(4)
C44	53(2)	6531.9(11)	7099.8(11)	34.6(4)
C45	-301(2)	5958.5(10)	6816.0(11)	32.5(3)
C46	-109(2)	6017.7(11)	6062.2(11)	33.1(4)
C47	413(2)	6691.6(10)	5563.7(11)	31.4(3)
C48	1300(2)	8025.0(10)	5323.1(10)	29.0(3)
C50	2153(2)	8728.9(10)	4096.6(10)	27.8(2)
C51	3419(2)	8540.3(9)	3544.7(10)	26.9(2)
C52	4430(2)	9247.7(10)	3222.3(10)	29.1(3)
C53	5342(2)	9406.3(10)	3802.8(11)	29.1(3)
C54	6118(2)	9508.8(10)	4279.8(11)	31.2(4)
C55	7122(2)	9620.5(12)	4855.0(12)	39.9(4)
C57	5590(2)	7588.2(10)	3743.9(10)	28.1(3)
C59	759(2)	9230.8(10)	3700.4(10)	30.4(3)
C60	840(2)	9959.6(11)	3564.9(11)	32.6(4)
C61	965(3)	10685.5(12)	3435.7(13)	40.9(5)

Atom	x	y	z	U_{eq}
C62	-657(2)	8861.8(12)	3506.0(13)	39.0(4)
C67	6051(2)	6833.7(10)	4296.1(10)	28.0(3)
C68	7353(2)	6423.1(10)	4114.1(11)	32.0(3)
C69	7724(2)	5693.4(11)	4577.5(11)	34.0(3)
C70	6772(2)	5397.6(10)	5219.1(11)	31.0(3)
C71	5484(2)	5796.9(10)	5418.7(11)	31.9(3)
C72	5124(2)	6524.1(10)	4948.2(10)	30.7(3)
C76	2689(2)	8277.1(10)	2942.6(10)	26.4(3)
C77	2285(2)	8792.9(10)	2248.5(10)	30.5(3)
C78	1525(2)	8561.4(11)	1719.2(11)	36.4(4)
C79	1191(3)	7805.3(12)	1886.0(12)	38.5(4)
C80	1578(3)	7278.5(11)	2567.7(13)	39.5(4)
C81	2320(2)	7521.3(10)	3095.5(11)	32.5(4)
Cl1	257.2(8)	7514.1(4)	1212.3(4)	60.33(18)
N64	-910(2)	5249.4(10)	7340.3(10)	40.6(4)
N73	7138(2)	4621.8(9)	5711.8(10)	34.4(3)
O49	1567.4(15)	8024.4(7)	4607.4(7)	28.5(2)
O56	4371.3(15)	7919.8(7)	4036.1(7)	28.0(2)
O58	6192.8(16)	7840.0(8)	3123.4(7)	34.4(3)
O63	1468(2)	8567.0(8)	5526.8(8)	46.0(4)
O65	-1057(3)	4717.8(9)	7099.2(10)	60.6(5)
O66	-1258(2)	5232.6(9)	7991.4(9)	50.3(4)
O74	8358.9(19)	4289.8(8)	5564.4(9)	43.3(3)
O75	6211.7(18)	4340.1(8)	6240.9(8)	41.3(3)

Table S10: Anisotropic Displacement Parameters (×10[4]) **3a-NO₂**. The anisotropic displacement factor exponent takes the form: $-2\pi^2[h^2a^{*2} \times U_{11} + \ldots + 2hka^* \times b^* \times U_{12}]$

Atom	U_{11}	U_{22}	U_{33}	U_{23}	U_{13}	U_{12}
C1	37.7(9)	33.1(5)	22.3(6)	-5.0(5)	-5.9(5)	-9.0(5)
C2	70.8(14)	36.6(6)	33.2(8)	-6.5(5)	-23.1(8)	-9.7(7)
C3	69.0(13)	40.0(7)	29.6(7)	-5.5(6)	-20.7(8)	-6.5(7)
C4	45.5(9)	35.8(6)	26.0(7)	-5.9(5)	-3.5(6)	-0.8(6)
C5	43.8(10)	32.6(6)	27.7(7)	-6.9(5)	-2.9(6)	-7.0(6)
C6	38.4(9)	33.4(6)	25.0(7)	-7.9(5)	-3.6(6)	-7.7(6)
C7	36.4(9)	33.5(6)	22.9(6)	-7.9(5)	-5.4(5)	-10.4(6)
C9	32.6(5)	27.5(6)	23.1(5)	-6.9(4)	-3.3(4)	-8.9(4)
C10	31.1(5)	24.2(6)	21.9(5)	-3.6(5)	-3.4(4)	-7.7(4)
C11	33.0(7)	25.3(5)	26.5(7)	-6.0(4)	-3.8(4)	-6.6(4)
C12	30.4(8)	29.4(8)	31.7(9)	-8.9(7)	-5.1(6)	-6.0(5)
C13	30.6(9)	40.9(10)	34.6(9)	-14.8(8)	-2.1(6)	-7.4(7)
C14	37.7(10)	58.7(13)	44.3(11)	-24.4(10)	2.0(8)	-8.6(9)
C16	29.8(7)	26.5(6)	24.6(7)	-8.6(5)	-1.9(6)	-7.0(5)
C18	35.7(8)	31.5(6)	21.9(8)	-6.3(5)	-3.9(6)	-12.0(5)
C19	44.1(9)	33.6(6)	28.3(9)	-10.7(7)	7.0(7)	-17.3(6)
C20	69.1(15)	37.0(7)	49.9(14)	-23.0(10)	26.1(12)	-23.2(9)
C21	33.6(7)	40.0(8)	36.9(10)	-11.9(8)	0.2(6)	-12.0(6)
C26	29.1(7)	25.9(6)	24.2(6)	-7.8(4)	-0.7(5)	-7.0(5)
C27	36.1(8)	29.6(7)	26.0(7)	-6.7(5)	-3.5(5)	-11.0(6)
C28	38.4(8)	30.8(7)	27.7(7)	-3.9(5)	-3.3(6)	-11.1(6)
C29	32.9(8)	26.1(6)	31.1(7)	-6.8(5)	2.0(6)	-8.1(5)
C30	35.4(8)	32.5(7)	32.3(7)	-8.3(6)	-2.5(6)	-13.0(6)
C31	33.7(8)	32.0(7)	27.7(6)	-6.7(5)	-3.4(5)	-11.2(5)
C35	29.4(7)	25.4(6)	22.7(5)	-5.8(5)	-4.5(4)	-7.8(5)
C36	36.5(9)	30.4(6)	23.1(6)	-4.6(5)	-1.7(6)	-3.8(6)
C37	39.0(9)	40.2(7)	25.2(6)	-7.5(6)	-0.1(6)	-4.8(7)
C38	39.5(9)	37.2(8)	28.1(7)	-13.0(6)	-2.8(6)	-3.9(7)
C39	41.0(9)	31.5(6)	36.5(9)	-13.9(5)	-2.2(7)	-4.7(6)
C40	36.2(9)	26.8(6)	27.0(7)	-7.2(5)	-4.0(6)	-5.9(5)
Cl2	59.8(3)	56.9(3)	43.2(3)	-25.9(3)	8.5(2)	-0.3(3)
N23	54.8(10)	39.0(7)	27.9(7)	-5.6(6)	-3.4(6)	1.2(7)

Atom	U_{11}	U_{22}	U_{33}	U_{23}	U_{13}	U_{12}
N32	36.8(8)	29.2(7)	37.4(8)	-6.6(5)	1.7(6)	-9.8(5)
O8	34.1(6)	27.7(5)	22.0(5)	-6.0(4)	-5.3(4)	-7.8(4)
O15	32.4(5)	26.6(4)	21.7(5)	-4.8(4)	-3.6(3)	-9.3(4)
O17	35.3(6)	32.7(6)	23.2(5)	-5.4(4)	-5.5(4)	-9.8(5)
O22	66.1(9)	32.3(5)	27.7(6)	-9.1(5)	-12.5(6)	-12.0(5)
O24	78.3(10)	36.5(6)	34.3(8)	-1.9(5)	-9.6(7)	-3.0(6)
O25	62.6(9)	47.1(8)	35.9(7)	-0.5(6)	-12.3(6)	3.4(6)
O33	57.0(9)	41.4(7)	40.5(6)	4.8(5)	-5.4(6)	-21.7(7)
O34	48.7(8)	33.4(6)	47.6(7)	-7.1(6)	-1.4(6)	-18.3(6)
C42	29.6(8)	26.3(5)	24.4(6)	-6.7(5)	-1.8(5)	0.2(5)
C43	42.3(10)	31.8(6)	25.9(6)	-8.7(5)	-1.3(6)	-6.9(6)
C44	43.3(10)	35.3(7)	24.4(5)	-6.8(5)	-1.8(6)	-9.4(7)
C45	32.3(8)	30.6(5)	31.3(7)	-4.1(5)	-5.4(6)	-4.6(5)
C46	40.6(9)	29.5(6)	29.5(6)	-8.5(5)	-5.0(6)	-5.1(6)
C47	37.1(9)	29.8(6)	28.1(6)	-9.7(5)	-3.0(6)	-3.5(6)
C48	34.8(8)	28.2(6)	24.6(6)	-9.1(5)	-1.9(5)	-1.4(6)
C50	32.2(5)	24.7(6)	26.5(5)	-7.6(4)	-1.4(4)	-2.7(4)
C51	29.9(5)	23.9(6)	24.4(6)	-3.7(5)	-3.1(4)	-2.2(4)
C52	30.2(7)	24.9(5)	29.9(7)	-5.0(5)	-2.5(5)	-2.7(5)
C53	28.4(8)	25.6(7)	32.2(8)	-7.1(7)	-1.2(6)	-2.8(6)
C54	30.6(9)	29.8(9)	33.8(9)	-10.6(7)	-1.4(7)	-3.2(7)
C55	35.7(10)	46.6(11)	42.7(10)	-21.0(9)	-5.4(8)	-3.0(8)
C57	29.9(7)	27.8(6)	27.2(8)	-8.7(5)	-6.9(6)	-0.7(5)
C59	33.8(7)	31.3(6)	24.5(8)	-7.1(5)	-0.8(6)	1.3(5)
C60	35.7(8)	33.3(6)	25.3(9)	-4.4(6)	-6.9(7)	3.9(5)
C61	55.3(13)	31.0(7)	35.7(11)	-8.4(8)	-15.7(10)	4.6(7)
C62	32.1(7)	43.0(9)	43.8(11)	-16.4(9)	-5.3(7)	1.8(6)
C67	30.7(7)	27.5(5)	26.6(7)	-9.1(4)	-7.4(5)	0.2(4)
C68	33.9(8)	32.0(7)	29.5(7)	-8.7(5)	-7.0(5)	3.2(5)
C69	36.8(8)	32.1(6)	33.3(8)	-10.5(5)	-7.5(6)	5.5(5)
C70	36.4(8)	27.6(6)	30.1(8)	-9.7(4)	-10.2(6)	1.6(5)
C71	38.3(8)	28.1(6)	28.6(7)	-7.6(5)	-6.3(5)	1.4(5)
C72	36.3(8)	28.3(6)	27.1(7)	-8.0(5)	-6.3(5)	2.1(5)
C76	27.4(7)	26.1(7)	24.9(5)	-6.7(5)	-2.4(5)	-0.3(5)
C77	34.6(8)	29.8(6)	25.7(7)	-6.0(5)	-4.7(6)	-1.4(6)
C78	41.2(10)	38.7(8)	28.4(6)	-8.3(6)	-9.2(6)	1.0(7)
C79	42.2(10)	41.9(10)	36.5(8)	-18.4(8)	-11.1(7)	-0.1(7)
C80	45.5(10)	30.2(7)	45.3(10)	-13.9(6)	-11.2(8)	-2.6(6)
C81	37.6(9)	26.9(6)	32.4(7)	-7.6(5)	-7.7(6)	-1.9(5)
Cl1	76.1(4)	59.6(4)	57.5(4)	-32.5(3)	-32.0(3)	1.7(3)
N64	48.4(9)	37.0(7)	32.6(7)	-2.8(6)	-7.1(6)	-13.5(6)
N73	41.0(8)	29.5(6)	33.8(8)	-10.6(5)	-11.2(6)	3.3(5)
O49	35.4(6)	25.5(5)	24.9(5)	-7.6(3)	-0.1(4)	-4.7(4)
O56	32.1(5)	25.5(4)	24.5(5)	-4.9(4)	-4.3(3)	-0.3(3)
O58	35.6(6)	35.9(6)	28.1(5)	-5.3(4)	-1.3(4)	2.5(5)
O63	80.7(11)	30.6(5)	29.3(6)	-12.4(5)	7.2(7)	-14.1(6)
O65	96.2(14)	38.7(6)	43.2(8)	-3.5(6)	-3.3(8)	-27.2(7)
O66	61.7(9)	49.3(8)	34.0(6)	-1.3(5)	-3.3(6)	-23.1(7)
O74	48.6(7)	33.1(6)	44.4(8)	-7.8(5)	-6.5(5)	9.5(5)
O75	48.1(7)	30.8(6)	39.7(7)	-3.9(5)	-5.8(5)	3.2(5)

Table S11: Bond Lengths in Å for **3a-NO$_2$**.

Atom	Atom	Length/Å
C1	C2	1.395(3)
C1	C6	1.389(3)
C1	C7	1.489(3)
C2	C3	1.379(3)
C3	C4	1.377(3)
C4	C5	1.380(3)
C4	N23	1.472(3)

Atom	Atom	Length/Å
C5	C6	1.388(3)
C7	O8	1.353(2)
C7	O22	1.206(2)
C9	C10	1.553(2)
C9	C18	1.523(2)
C9	O8	1.451(2)
C10	C11	1.545(2)

Atom	Atom	Length/Å
C10	C35	1.520(2)
C10	O15	1.463(2)
C11	C12	1.465(3)
C12	C13	1.193(3)
C13	C14	1.466(3)
C16	C26	1.500(2)
C16	O15	1.344(2)
C16	O17	1.207(2)
C18	C19	1.305(3)
C18	C21	1.511(3)
C19	C20	1.302(3)
C26	C27	1.390(3)
C26	C31	1.393(2)
C27	C28	1.388(2)
C28	C29	1.384(3)
C29	C30	1.381(3)
C29	N32	1.474(2)
C30	C31	1.389(3)
C35	C36	1.392(2)
C35	C40	1.397(2)
C36	C37	1.391(3)
C37	C38	1.380(3)
C38	C39	1.387(3)
C38	Cl2	1.750(2)
C39	C40	1.386(3)
N23	O24	1.227(2)
N23	O25	1.229(2)
N32	O33	1.225(2)
N32	O34	1.227(2)
C42	C43	1.393(3)
C42	C47	1.396(2)
C42	C48	1.487(2)
C43	C44	1.383(3)
C44	C45	1.382(3)
C45	C46	1.381(3)
C45	N64	1.474(2)

Atom	Atom	Length/Å
C46	C47	1.385(3)
C48	O49	1.347(2)
C48	O63	1.201(2)
C50	C51	1.551(2)
C50	C59	1.521(2)
C50	O49	1.452(2)
C51	C52	1.544(2)
C51	C76	1.524(2)
C51	O56	1.453(2)
C52	C53	1.466(2)
C53	C54	1.194(3)
C54	C55	1.466(3)
C57	C67	1.500(2)
C57	O56	1.349(2)
C57	O58	1.205(2)
C59	C60	1.302(3)
C59	C62	1.516(3)
C60	C61	1.303(3)
C67	C68	1.390(3)
C67	C72	1.394(3)
C68	C69	1.388(3)
C69	C70	1.387(3)
C70	C71	1.382(3)
C70	N73	1.471(2)
C71	C72	1.387(3)
C76	C77	1.393(2)
C76	C81	1.392(2)
C77	C78	1.387(3)
C78	C79	1.382(3)
C79	C80	1.381(3)
C79	Cl1	1.7480(19)
C80	C81	1.389(3)
N64	O65	1.222(2)
N64	O66	1.230(2)
N73	O74	1.233(2)
N73	O75	1.227(2)

Table S12: Bond Angles in ° for **3a-NO$_2$**.

Atom	Atom	Atom	Angle/°
C2	C1	C7	117.04(17)
C6	C1	C2	120.34(18)
C6	C1	C7	122.48(16)
C3	C2	C1	120.15(19)
C4	C3	C2	118.25(19)
C3	C4	C5	123.12(19)
C3	C4	N23	118.33(18)
C5	C4	N23	118.53(18)
C4	C5	C6	118.20(18)
C5	C6	C1	119.85(17)
O8	C7	C1	113.56(15)
O22	C7	C1	123.10(16)
O22	C7	O8	123.32(17)
C18	C9	C10	112.96(14)
O8	C9	C10	109.06(13)
O8	C9	C18	110.81(14)
C11	C10	C9	106.45(14)
C35	C10	C9	113.31(14)
C35	C10	C11	113.20(14)
O15	C10	C9	103.05(13)

Atom	Atom	Atom	Angle/°
O15	C10	C11	110.44(14)
O15	C10	C35	109.90(13)
C12	C11	C10	112.57(15)
C13	C12	C11	177.8(2)
C12	C13	C14	178.0(2)
O15	C16	C26	110.89(14)
O17	C16	C26	123.51(15)
O17	C16	O15	125.59(16)
C19	C18	C9	117.06(18)
C19	C18	C21	123.30(17)
C21	C18	C9	119.56(16)
C20	C19	C18	177.5(2)
C27	C26	C16	121.83(15)
C27	C26	C31	120.62(16)
C31	C26	C16	117.47(16)
C28	C27	C26	120.26(16)
C29	C28	C27	117.84(17)
C28	C29	N32	118.29(17)
C30	C29	C28	123.20(17)
C30	C29	N32	118.51(16)

Atom	Atom	Atom	Angle/°
C29	C30	C31	118.36(17)
C30	C31	C26	119.69(17)
C36	C35	C10	121.01(16)
C36	C35	C40	118.64(17)
C40	C35	C10	120.24(16)
C37	C36	C35	120.93(18)
C38	C37	C36	119.04(18)
C37	C38	C39	121.48(18)
C37	C38	Cl2	119.00(15)
C39	C38	Cl2	119.51(15)
C40	C39	C38	118.85(18)
C39	C40	C35	121.04(18)
O24	N23	C4	118.04(18)
O24	N23	O25	124.20(18)
O25	N23	C4	117.75(18)
O33	N32	C29	118.29(15)
O33	N32	O34	123.65(16)
O34	N32	C29	118.06(16)
C7	O8	C9	112.79(13)
C16	O15	C10	118.78(13)
C43	C42	C47	120.44(17)
C43	C42	C48	117.55(16)
C47	C42	C48	122.01(16)
C44	C43	C42	120.24(17)
C45	C44	C43	118.08(18)
C44	C45	N64	118.65(17)
C46	C45	C44	123.04(18)
C46	C45	N64	118.31(17)
C45	C46	C47	118.53(17)
C46	C47	C42	119.63(17)
O49	C48	C42	112.85(14)
O63	C48	C42	123.53(17)
O63	C48	O49	123.62(17)
C59	C50	C51	113.28(15)
O49	C50	C51	108.79(13)
O49	C50	C59	109.73(14)
C52	C51	C50	106.77(14)
C76	C51	C50	113.00(14)
C76	C51	C52	113.76(15)
O56	C51	C50	102.92(13)
O56	C51	C52	110.27(14)
O56	C51	C76	109.54(13)
C53	C52	C51	112.39(15)
C54	C53	C52	177.43(19)
C53	C54	C55	177.8(2)
O56	C57	C67	109.79(15)
O58	C57	C67	124.38(17)
O58	C57	O56	125.78(16)
C60	C59	C50	117.42(17)
C60	C59	C62	123.65(17)
C62	C59	C50	118.90(16)
C59	C60	C61	178.3(2)
C68	C67	C57	118.36(17)
C68	C67	C72	120.61(17)
C72	C67	C57	120.85(16)
C69	C68	C67	119.95(19)
C70	C69	C68	118.18(18)
C69	C70	N73	118.86(17)
C71	C70	C69	123.08(17)
C71	C70	N73	118.06(17)
C70	C71	C72	118.08(18)
C71	C72	C67	120.10(18)

Atom	Atom	Atom	Angle/°
C77	C76	C51	120.89(15)
C81	C76	C51	120.31(16)
C81	C76	C77	118.69(16)
C78	C77	C76	120.88(17)
C79	C78	C77	118.95(18)
C78	C79	Cl1	118.78(16)
C80	C79	C78	121.69(18)
C80	C79	Cl1	119.53(16)
C79	C80	C81	118.65(18)
C80	C81	C76	121.14(18)
O65	N64	C45	118.02(17)
O65	N64	O66	124.03(17)
O66	N64	C45	117.95(16)
O74	N73	C70	117.82(17)
O75	N73	C70	118.45(16)
O75	N73	O74	123.73(16)
C48	O49	C50	114.54(13)
C57	O56	C51	119.96(14)

Table S13: Hydrogen Fractional Atomic Coordinates (×10[4]) and Equivalent Isotropic Displacement Parameters (Å[2]×10[3]) for **3a-NO2**. U_{eq} is defined as 1/3 of the trace of the orthogonalised U_{ij}.

Atom	x	y	z	U_{eq}
H2	4120.21	2395.71	8424.48	56
H3	3466.2	3534.27	7487.75	56
H5	5656.56	4701.44	8642.38	42
H6	6131.75	3567.02	9628.09	39
H9	7354.68	1201.18	10528.08	33
H11A	9502.34	935.91	12141.38	34
H11B	8153.91	453.83	11948.29	34
H14A	11312.45	570.79	9623.46	67
H14B	12376.48	-19.23	10252.73	67
H14C	12743.18	863.62	9983.28	67
H20A	5320(40)	-625(17)	10921(17)	67(8)
H20B	6280(40)	-862(18)	11797(18)	72(9)
H21A	4053.6	1607.12	11779.15	55
H21B	3068.71	924.48	11697.27	55
H21C	3308.57	1667.86	10988	55
H27	9133.91	3322.97	9855.63	37
H28	10223.81	4443.13	9054.87	40
H30	13462.89	4355.6	10637.94	40
H31	12394.17	3223.93	11427.36	38
H36	6979.83	851.41	12841.95	37
H37	5415.11	1229.74	13739.47	42
H39	5036.4	3367.81	12283.04	43
H40	6569.48	2981.64	11383.91	36
H43	852.97	7602.28	6779.72	40
H44	-70.85	6469	7623.28	42
H46	-330.03	5605.11	5889.74	40
H47	536.61	6750.01	5041.08	38
H50	2685.46	8999.3	4400.2	33
H52A	5177.94	9169.6	2826.14	35
H52B	3714.81	9692.55	2985.01	35
H55A	7936.67	9984.58	4611.2	60
H55B	7642.24	9135.17	5135.69	60
H55C	6457.55	9816.41	5202.66	60
H61A	470(30)	10914(14)	3797(15)	47(7)
H61B	1520(30)	11010(16)	2974(16)	60(8)
H62A	-281.32	8511.78	3231.29	58
H62B	-1379.14	9253.92	3190.25	58
H62C	-1227.8	8581.02	3970.74	58
H68	7988.18	6641.64	3672.79	38
H69	8605.63	5404.56	4458.65	41
H71	4863.39	5579.57	5865.22	38
H72	4243.65	6811.65	5070.49	37
H77	2533	9310.28	2136.05	37
H78	1239.45	8917.06	1249.45	44
H80	1341.09	6760.47	2673.65	47
H81	2579.89	7165.25	3568.99	39

Citations

O.V. Dolomanov and L.J. Bourhis and R.J. Gildea and J.A.K. Howard and H. Puschmann, Olex2: A complete structure solution, refinement and analysis program, *J. Appl. Cryst.*, (2009), **42**, 339-341.

Sheldrick, G.M. (2015). Acta Cryst. A71, 3-8.

^{1}H NMR (300 MHz, $CDCl_3$, rt)

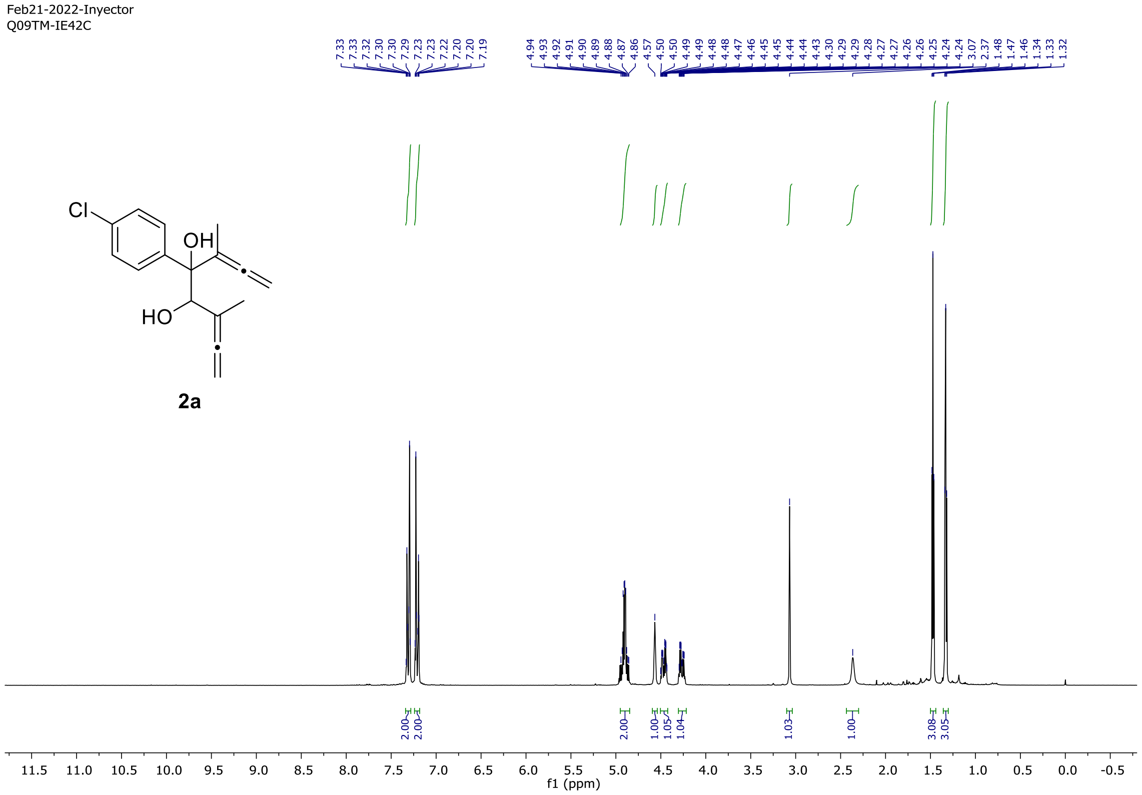

^{13}C NMR (75 MHz, $CDCl_3$, rt)

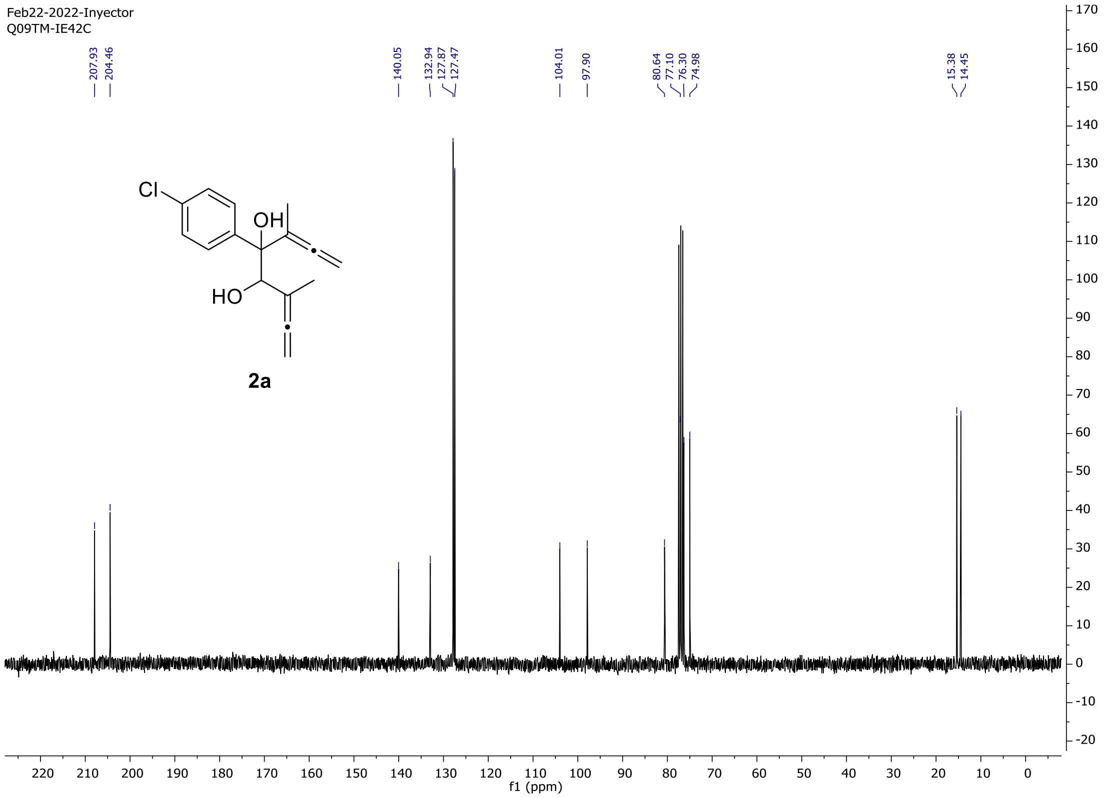

^{1}H NMR (300 MHz, $CDCl_3$, rt)

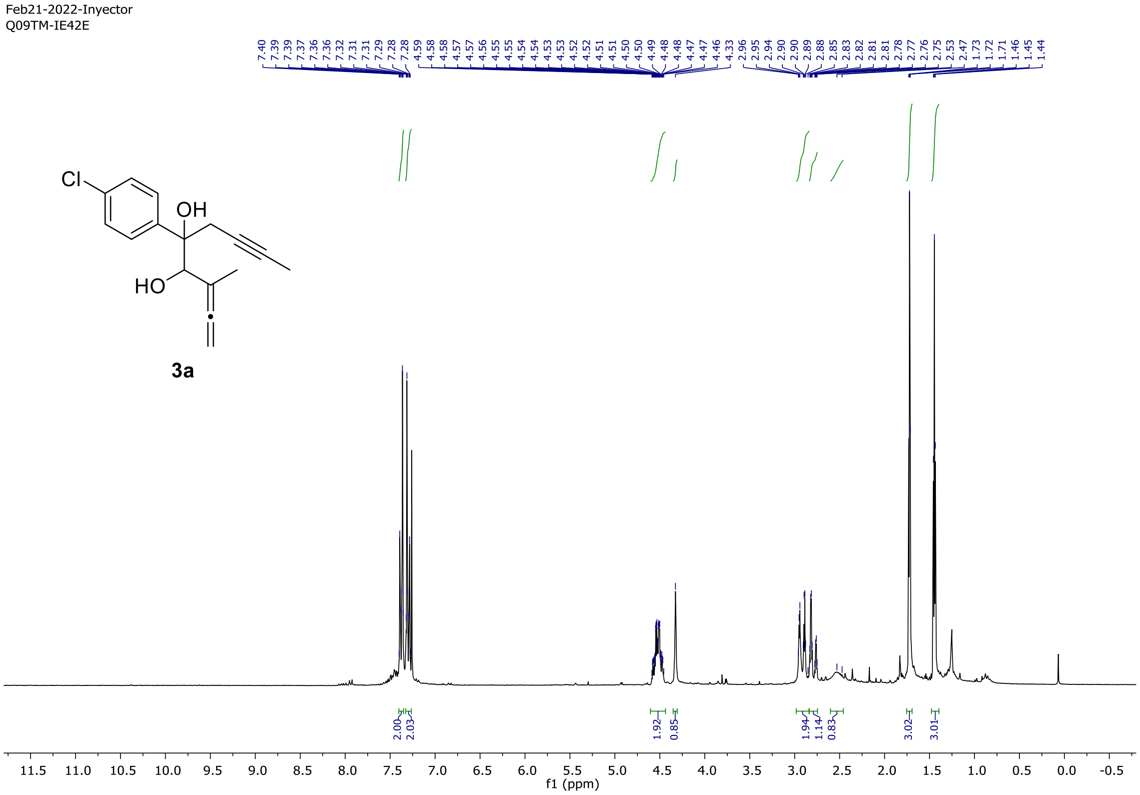

^{13}C NMR (75 MHz, $CDCl_3$, rt)

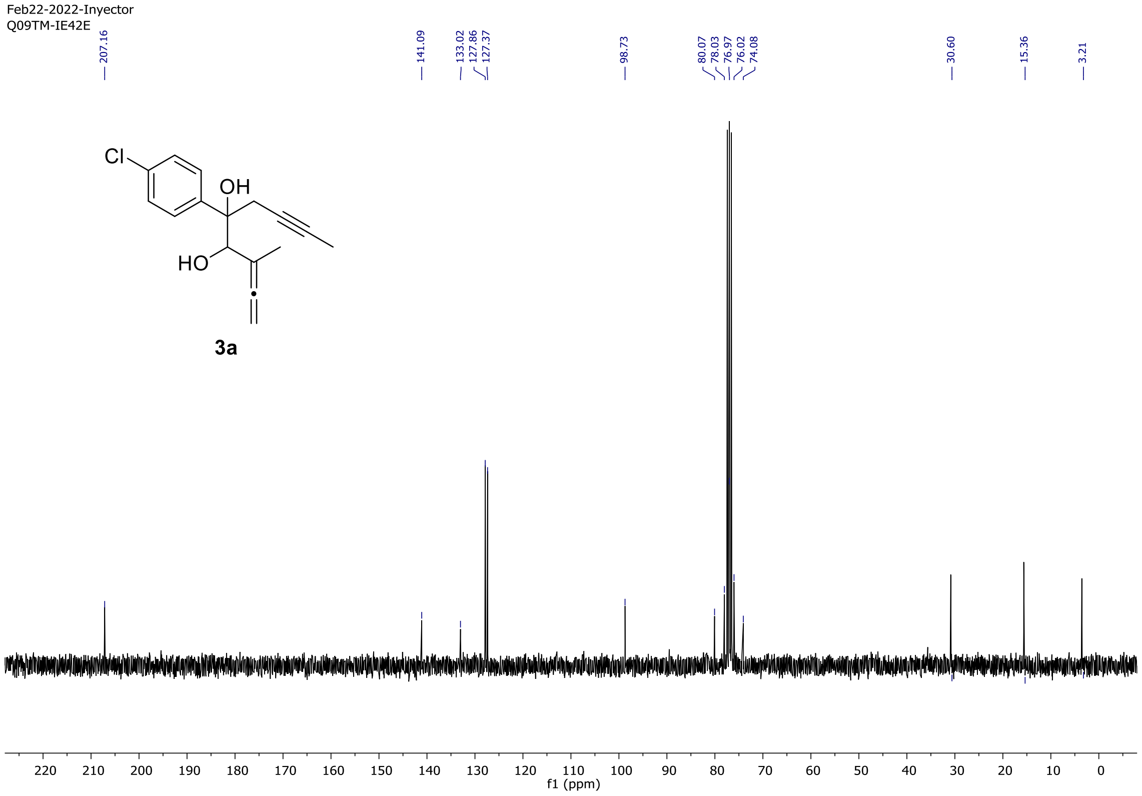

^{1}H NMR (300 MHz, $CDCl_3$, rt)

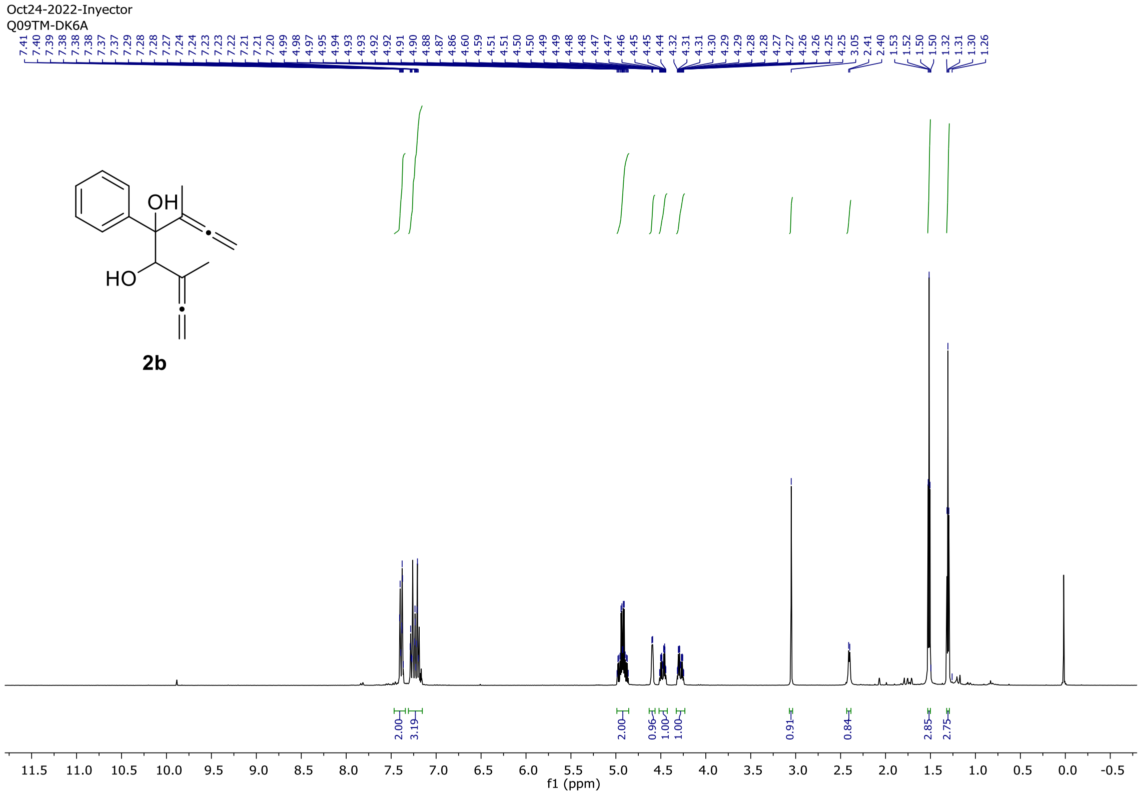

^{13}C NMR (75 MHz, $CDCl_3$, rt)

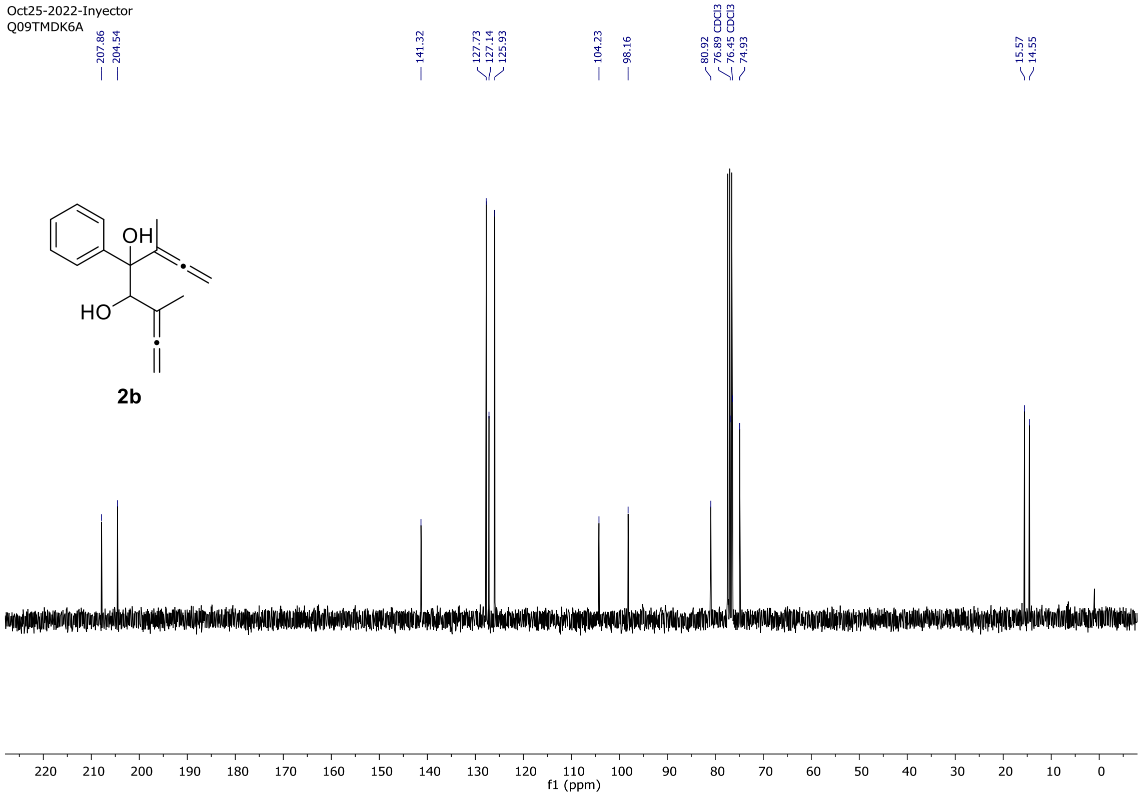

^{1}H NMR (300 MHz, $CDCl_3$, rt)

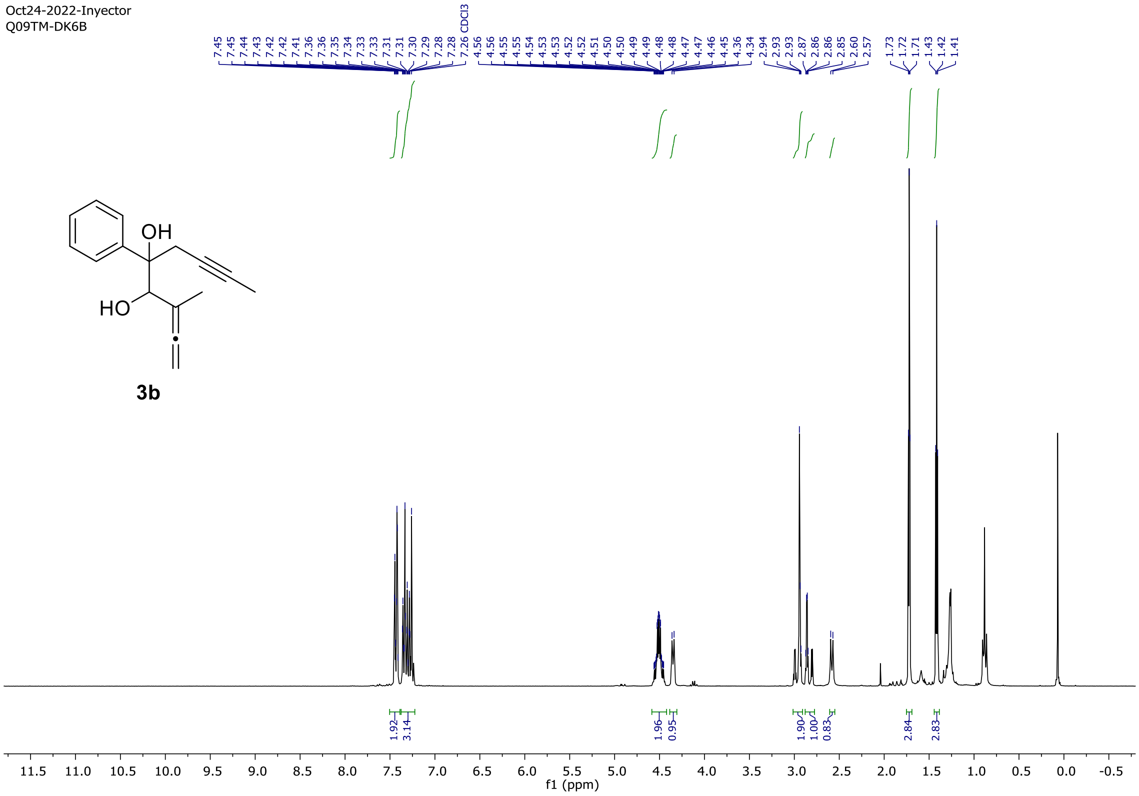

^{13}C NMR (75 MHz, $CDCl_3$, rt)

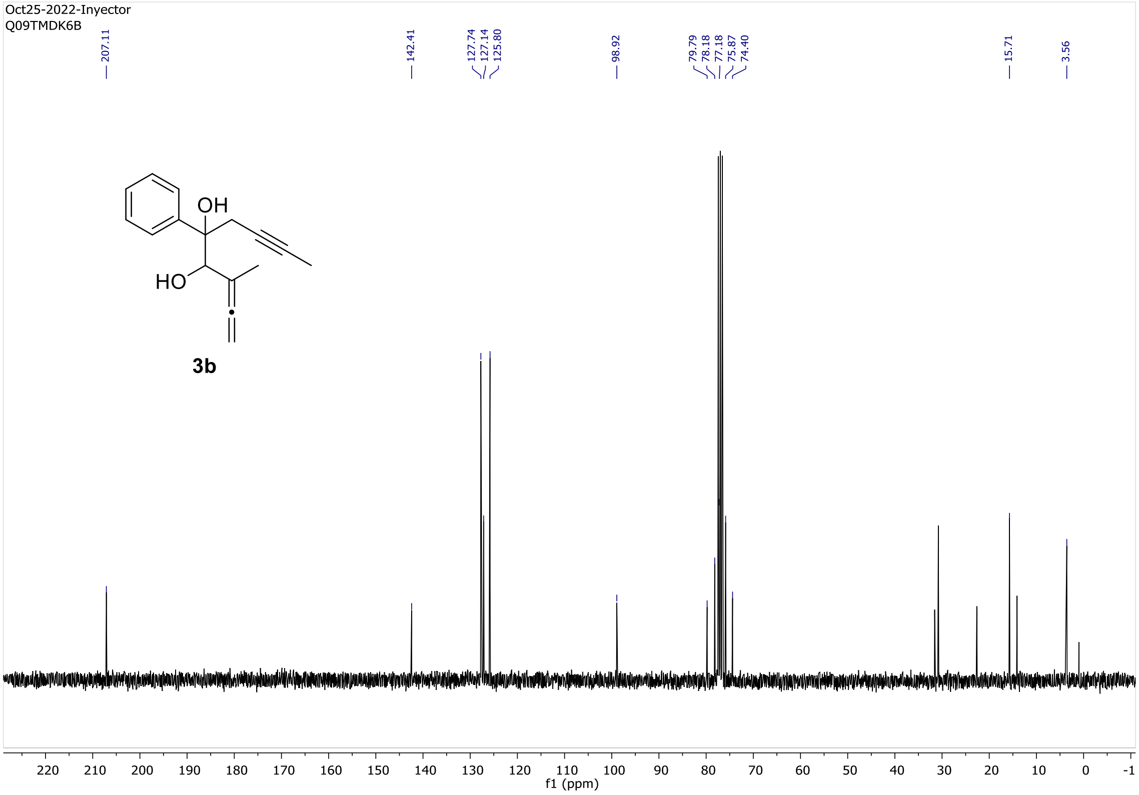

^{1}H NMR (300 MHz, $CDCl_3$, rt)

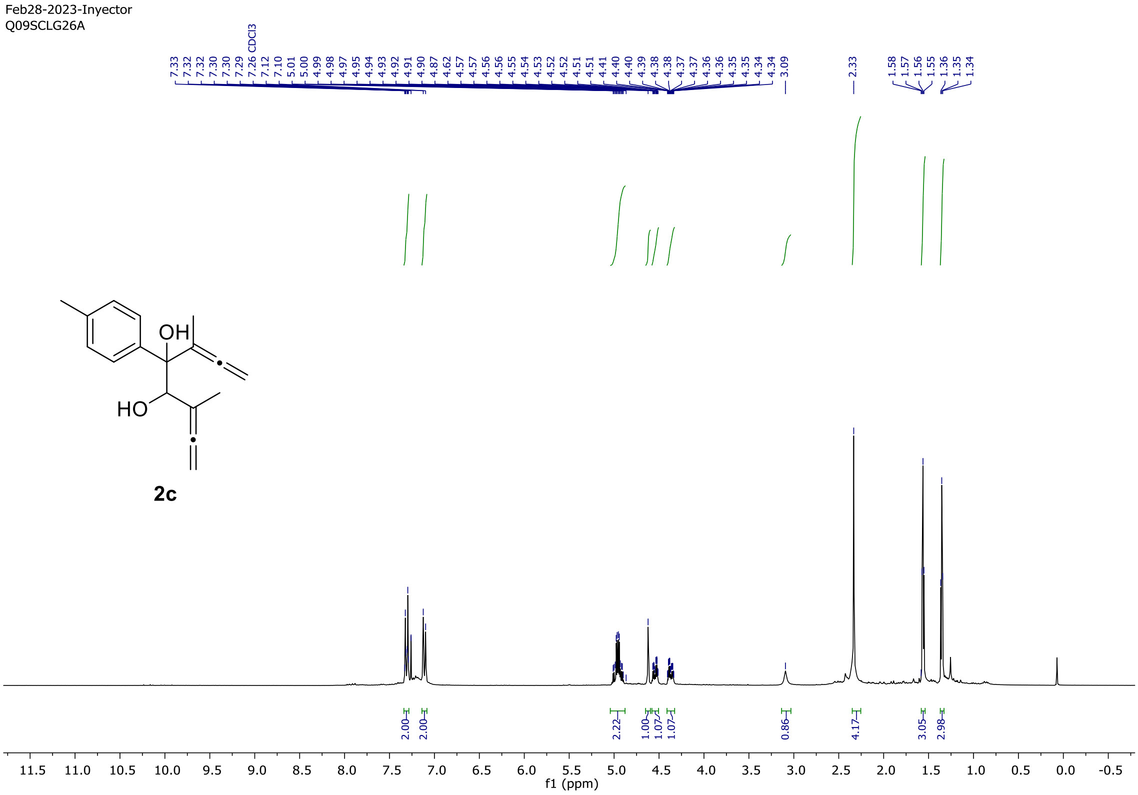

^{13}C NMR (75 MHz, $CDCl_3$, rt)

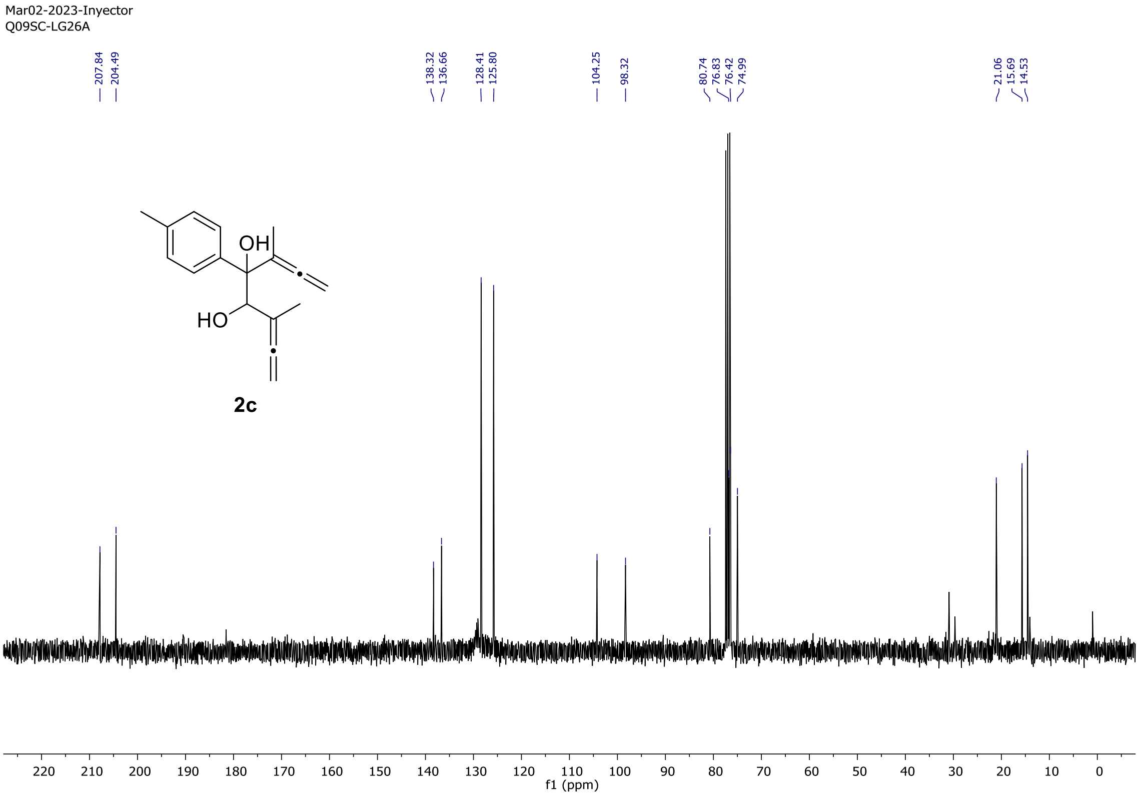

^{1}H NMR (300 MHz, $CDCl_3$, rt)

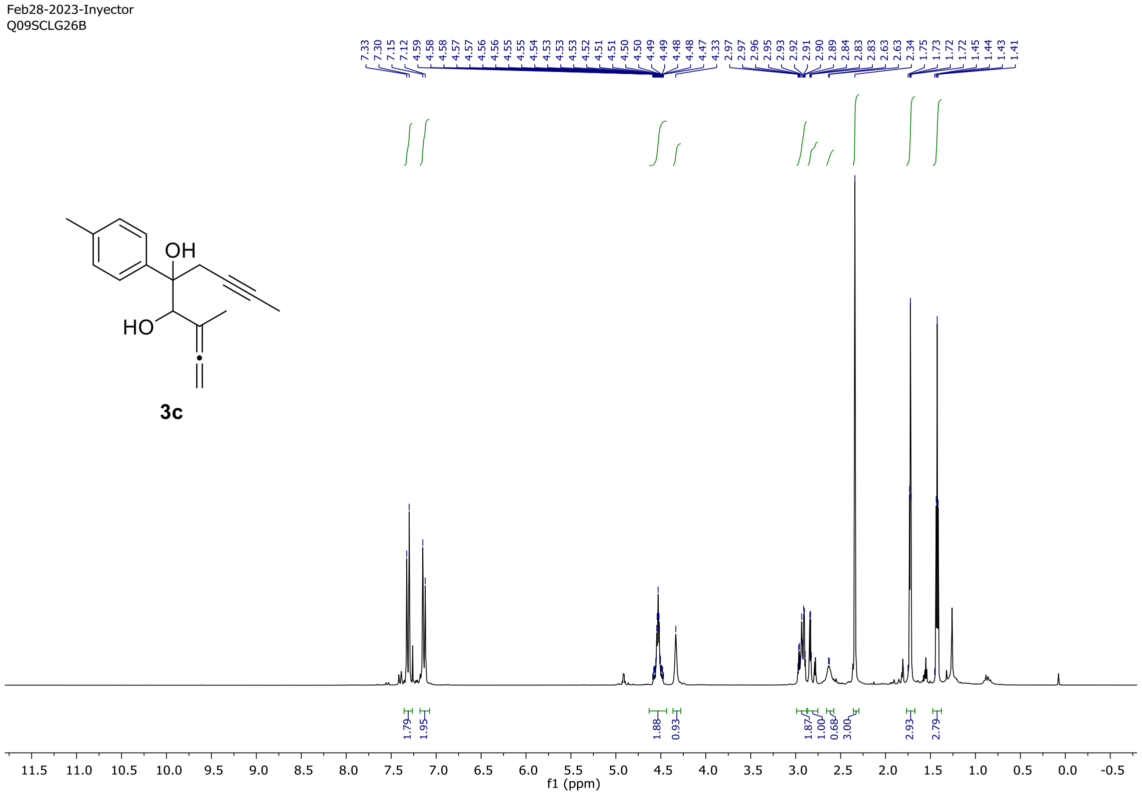

^{13}C NMR (75 MHz, $CDCl_3$, rt)

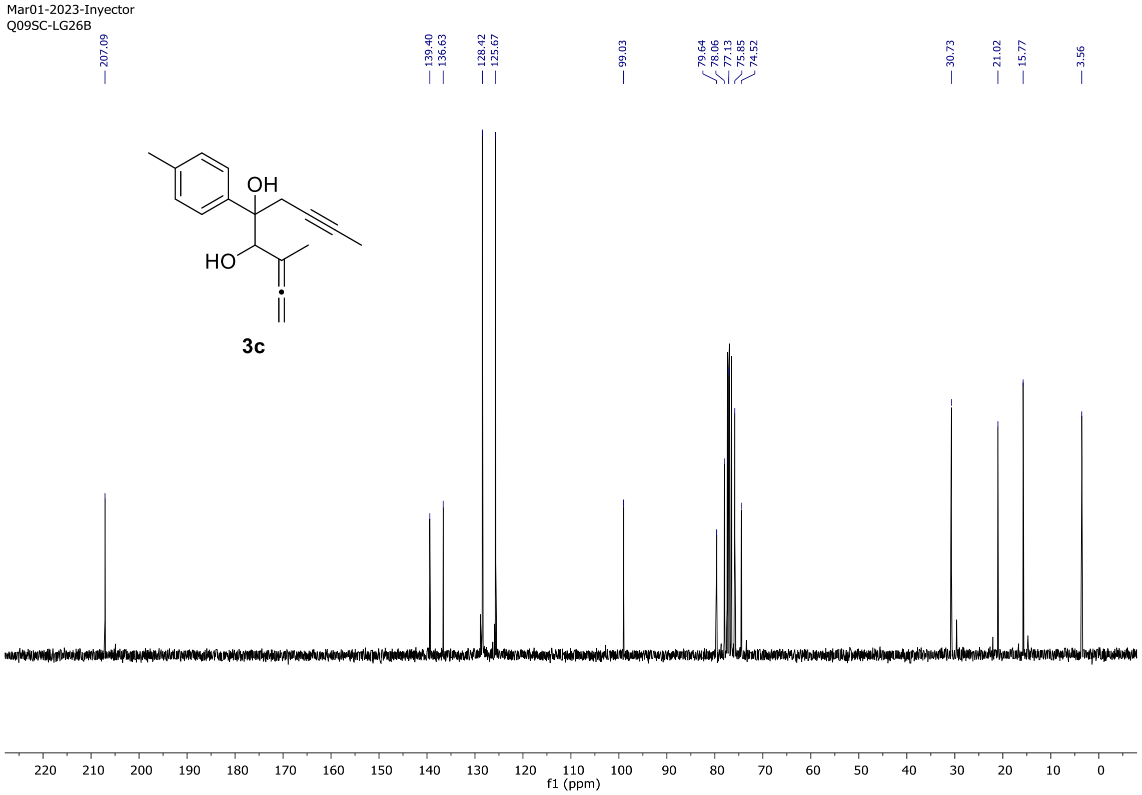

^{1}H NMR (300 MHz, $CDCl_3$, rt)

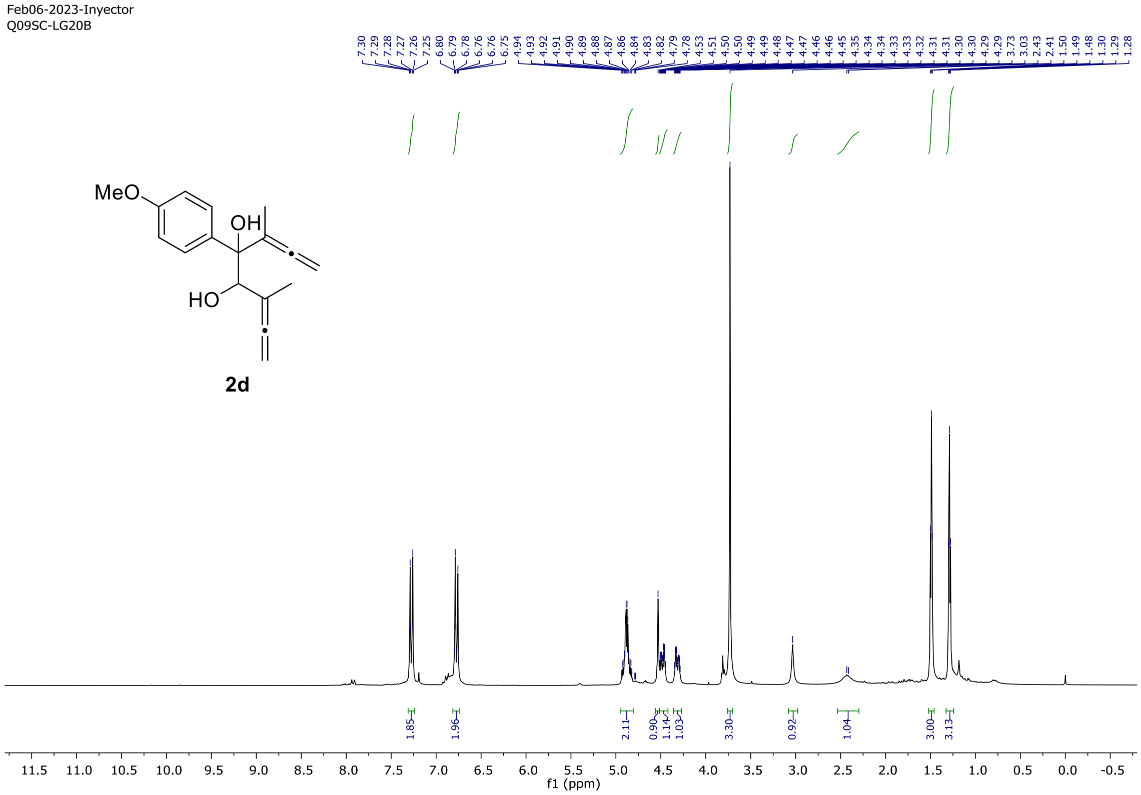

^{13}C NMR (75 MHz, $CDCl_3$, rt)

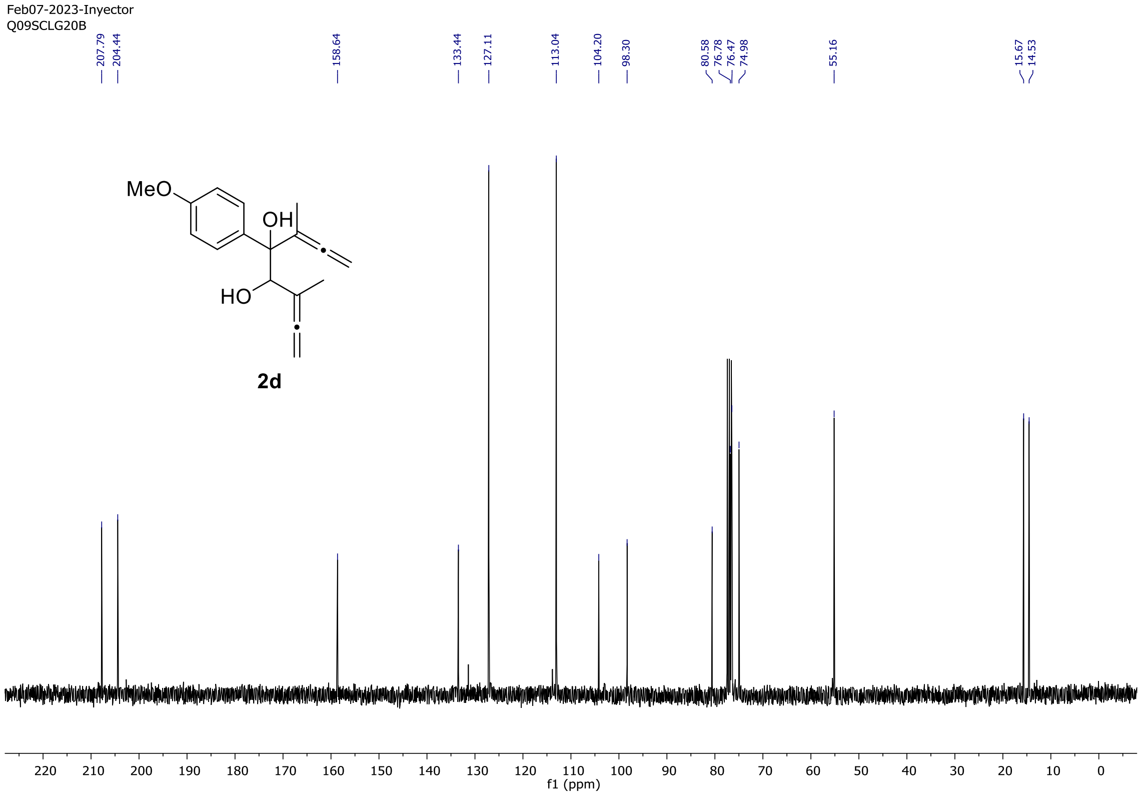

^{1}H NMR (300 MHz, $CDCl_3$, rt)

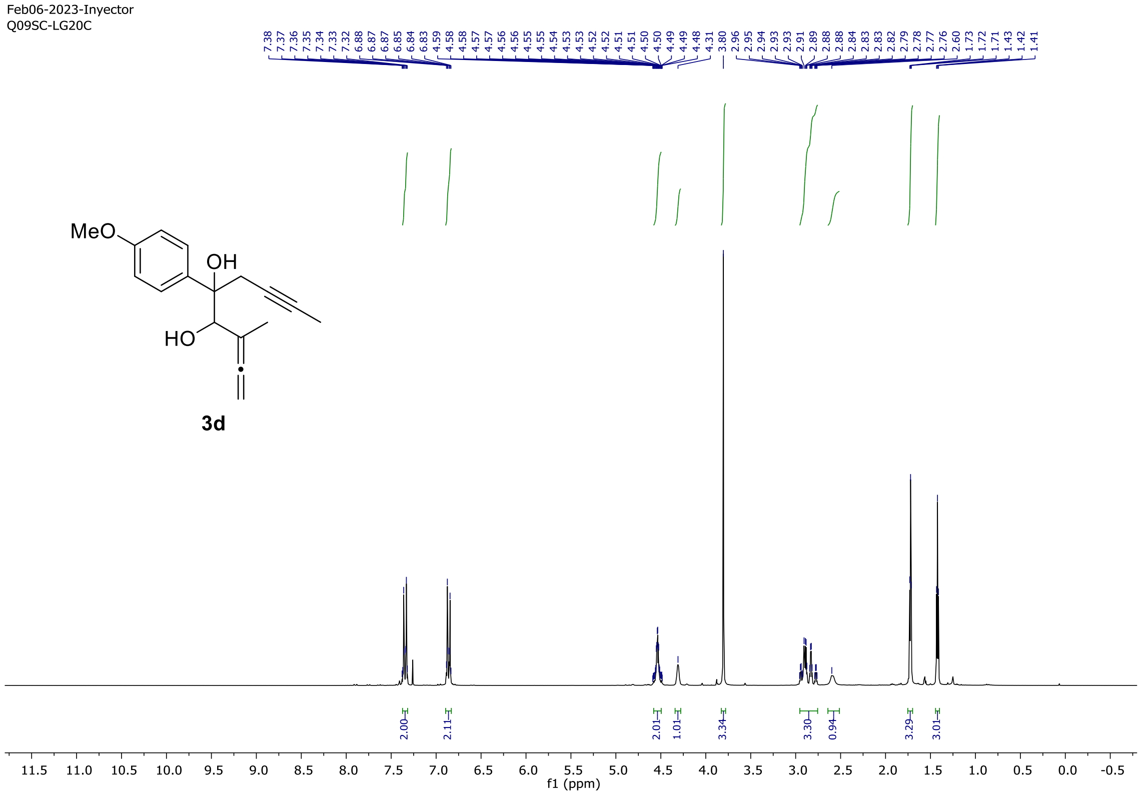

^{13}C NMR (75 MHz, $CDCl_3$, rt)

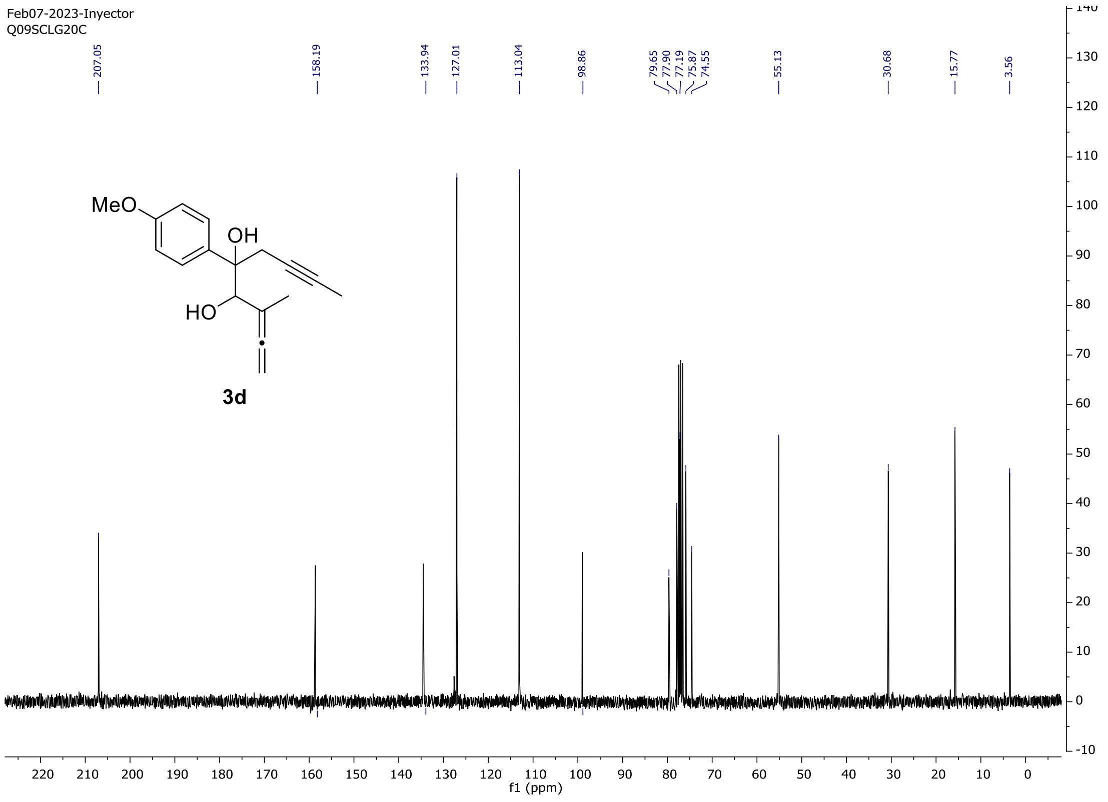

^1H NMR (300 MHz, acetone-d$_6$, rt)

Jan09-2024-Inyector.230.fid
Q09DS150FA

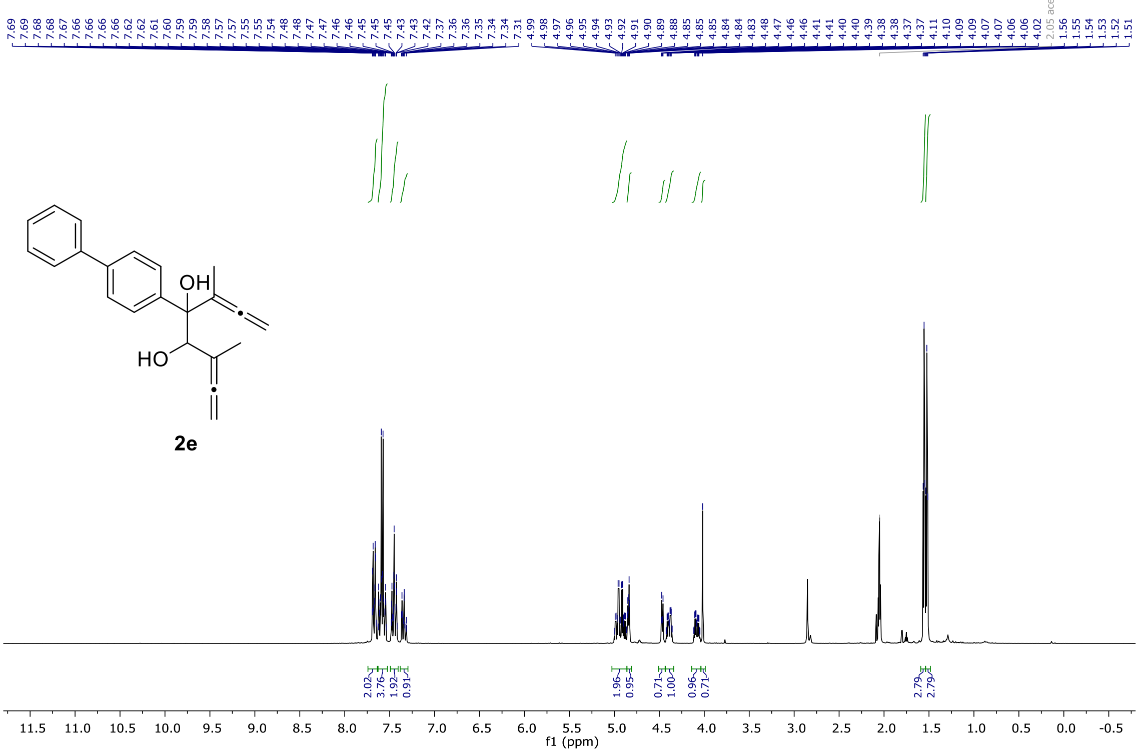

^{13}C NMR (75 MHz, acetone-d$_6$, rt)

Jan10-2024-Inyector.10.1.1r
Q09DS150FA

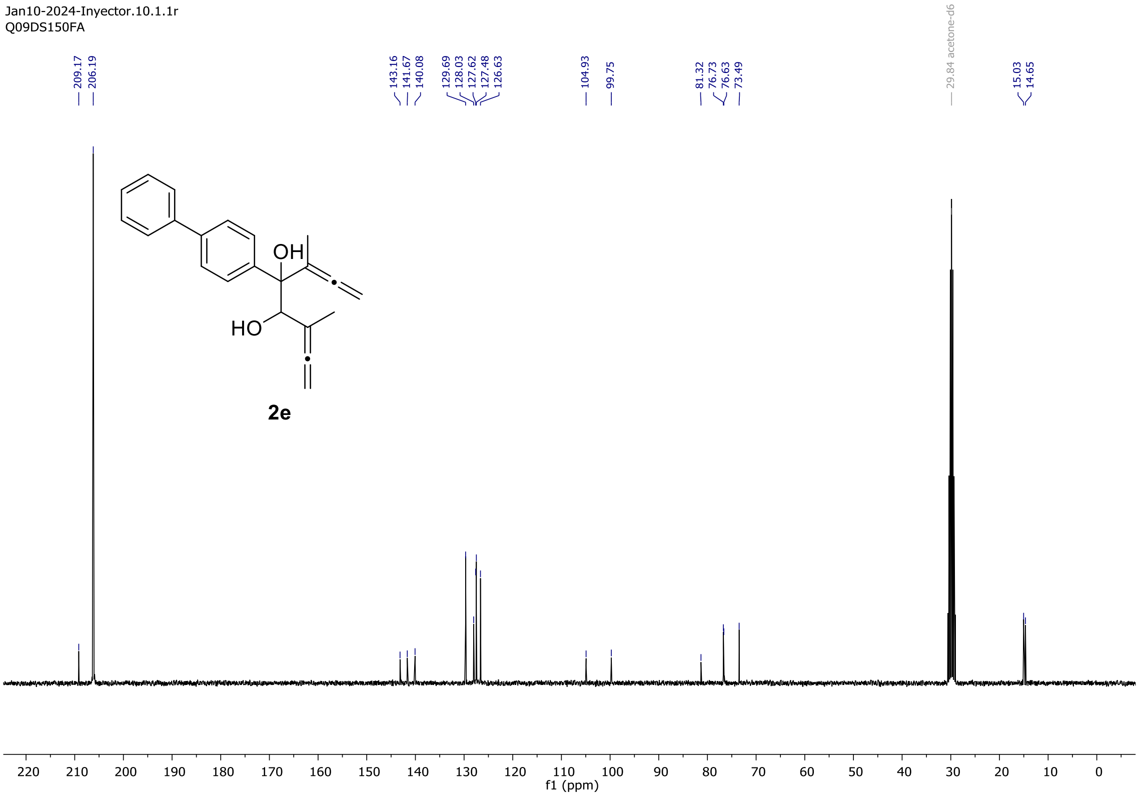

^{1}H NMR (300 MHz, acetone-d$_6$, rt)

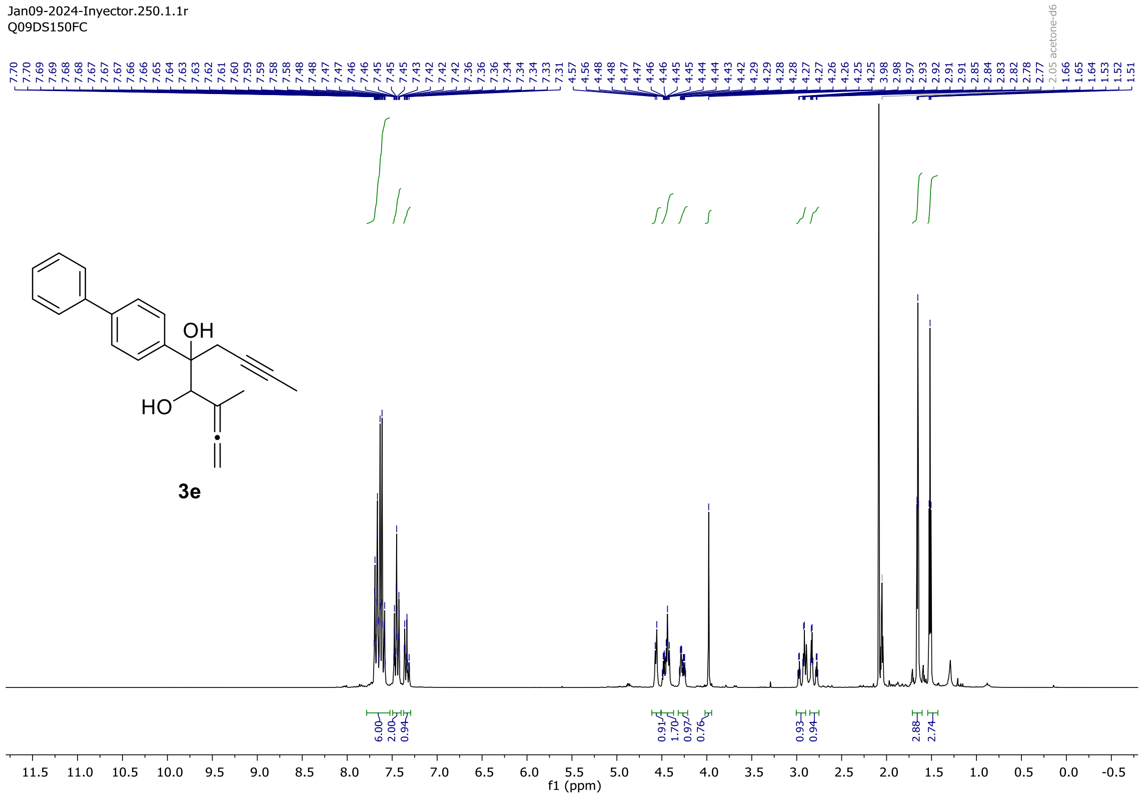

^{13}C NMR (75 MHz, acetone-d$_6$, rt)

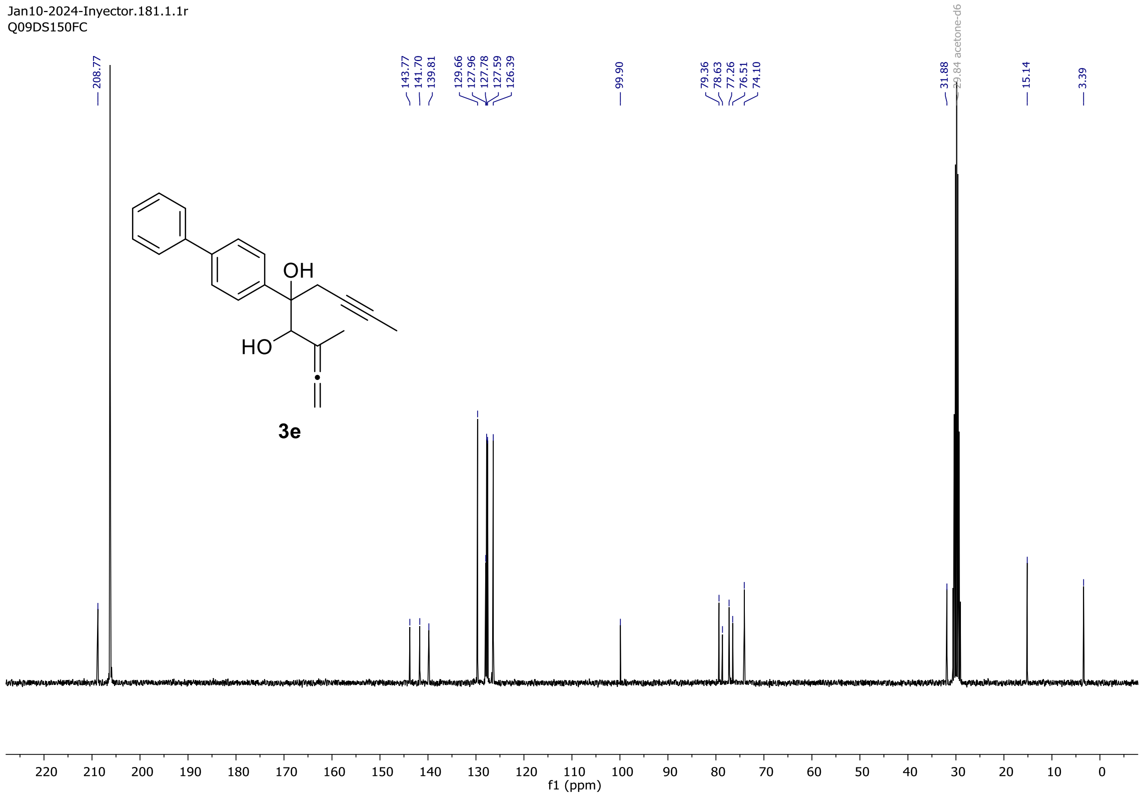

^1H NMR (300 MHz, acetone-d$_6$, rt)

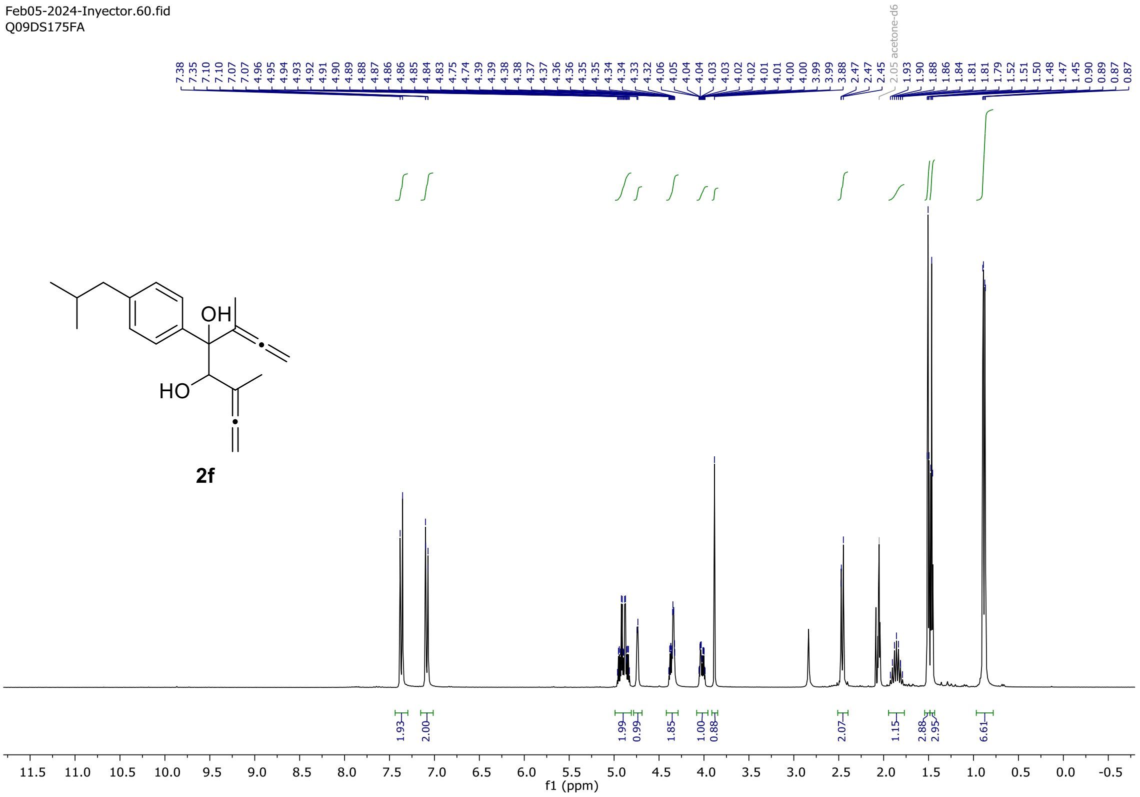

^{13}C NMR (75 MHz, acetone-d$_6$, rt)

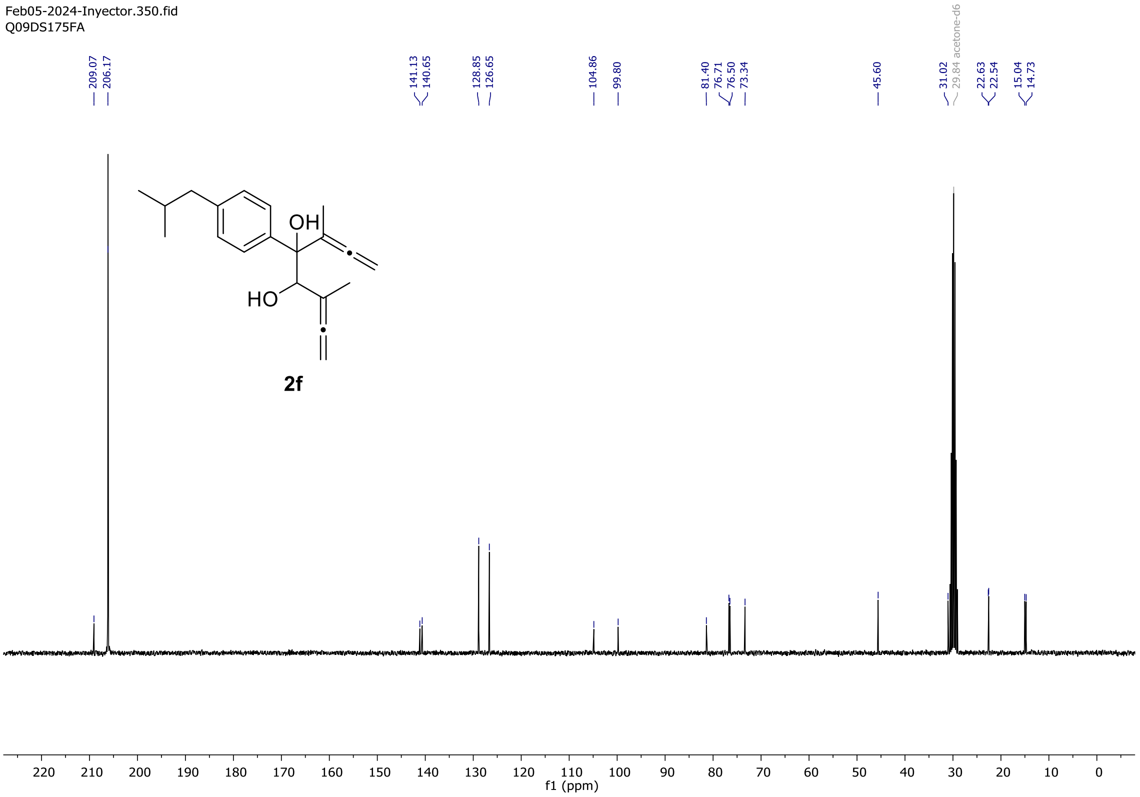

^{1}H NMR (300 MHz, acetone-d$_6$, rt)

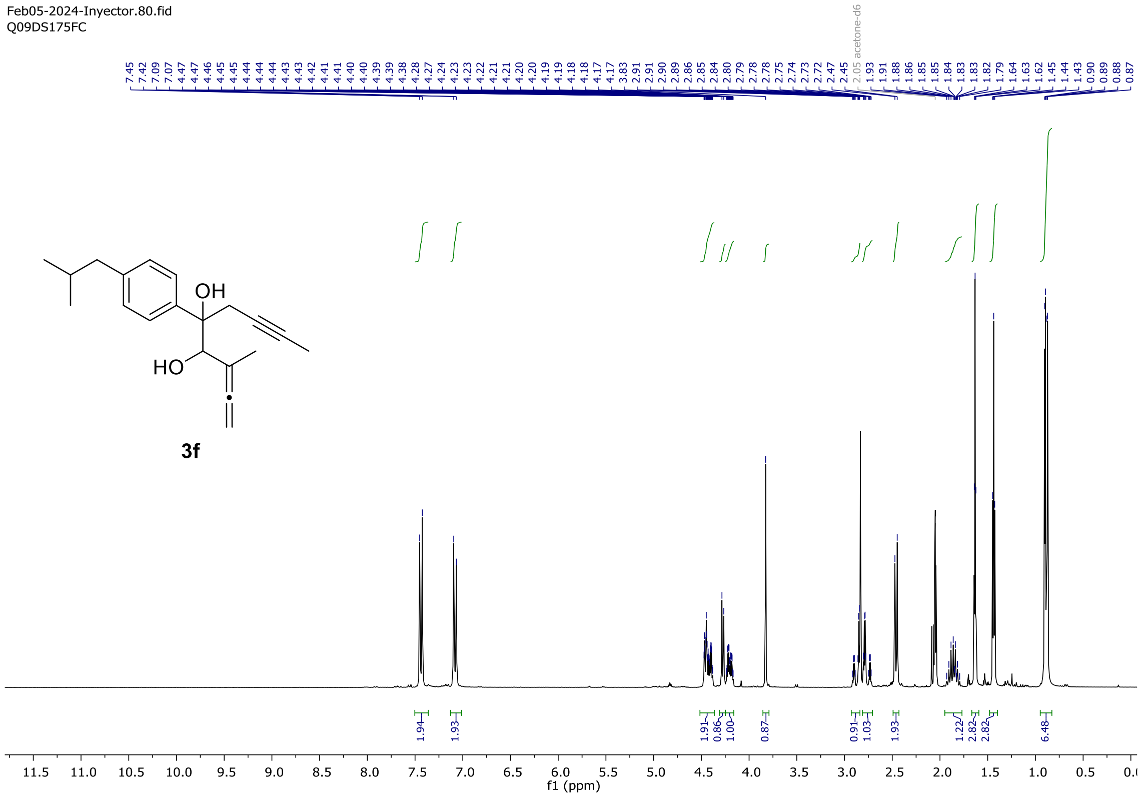

^{13}C NMR (75 MHz, acetone-d$_6$, rt)

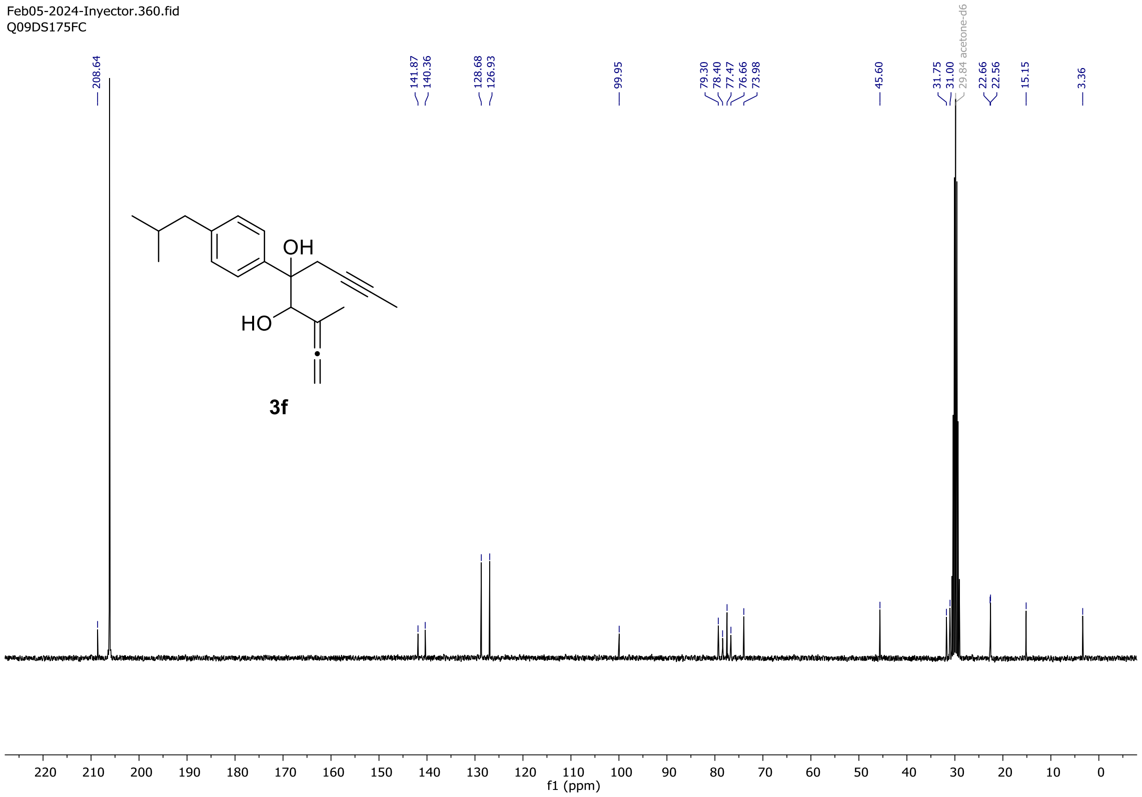

^{1}H NMR (300 MHz, acetone-d$_6$, rt)

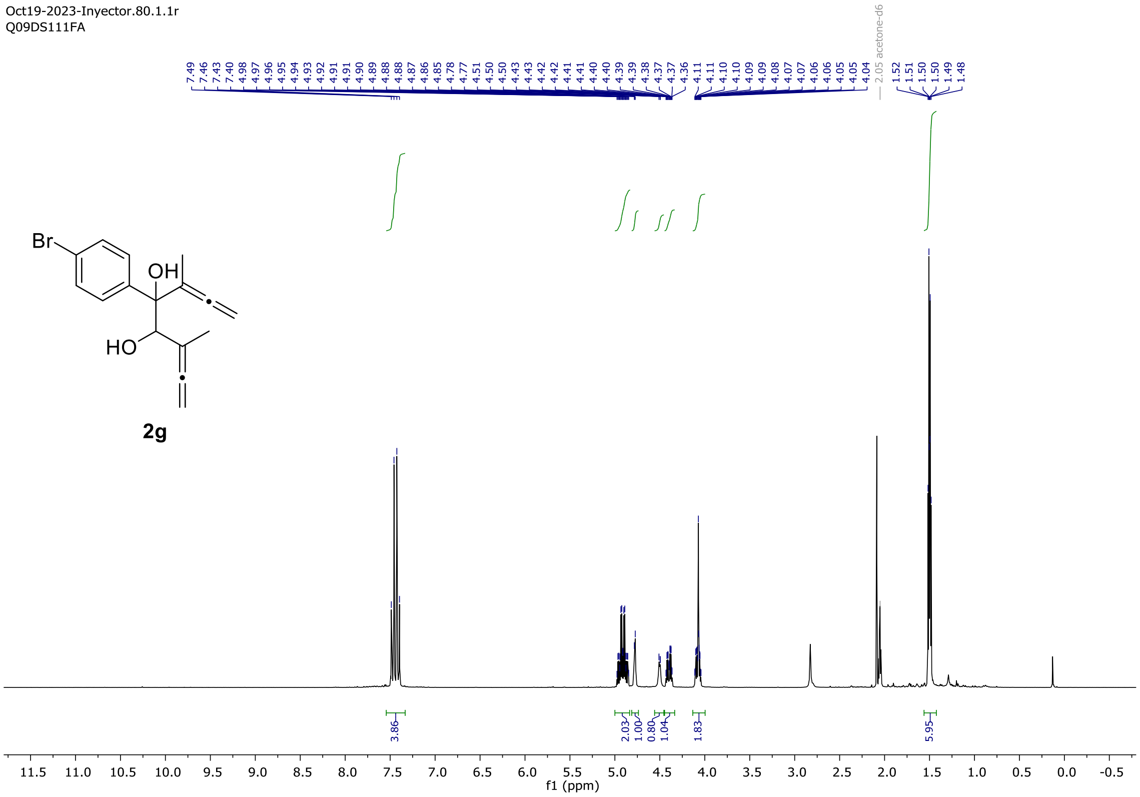

^{13}C NMR (75 MHz, acetone-d$_6$, rt)

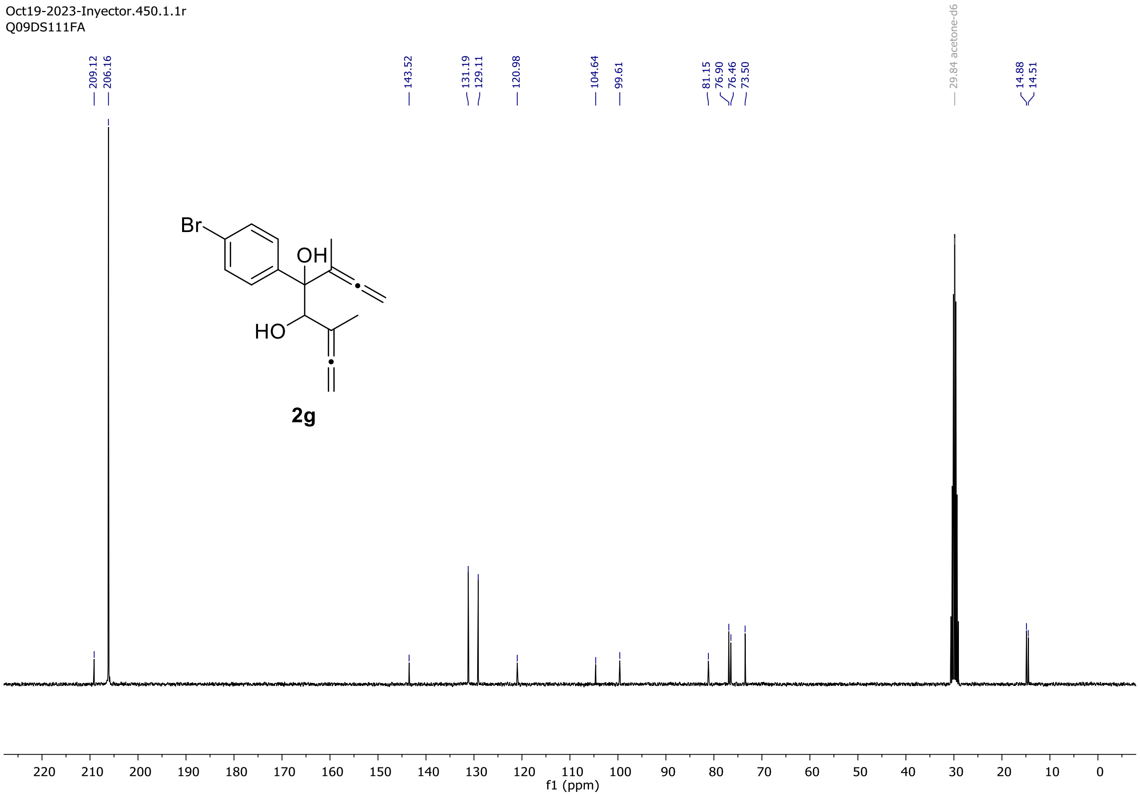

^{1}H NMR (300 MHz, acetone-d$_6$, rt)

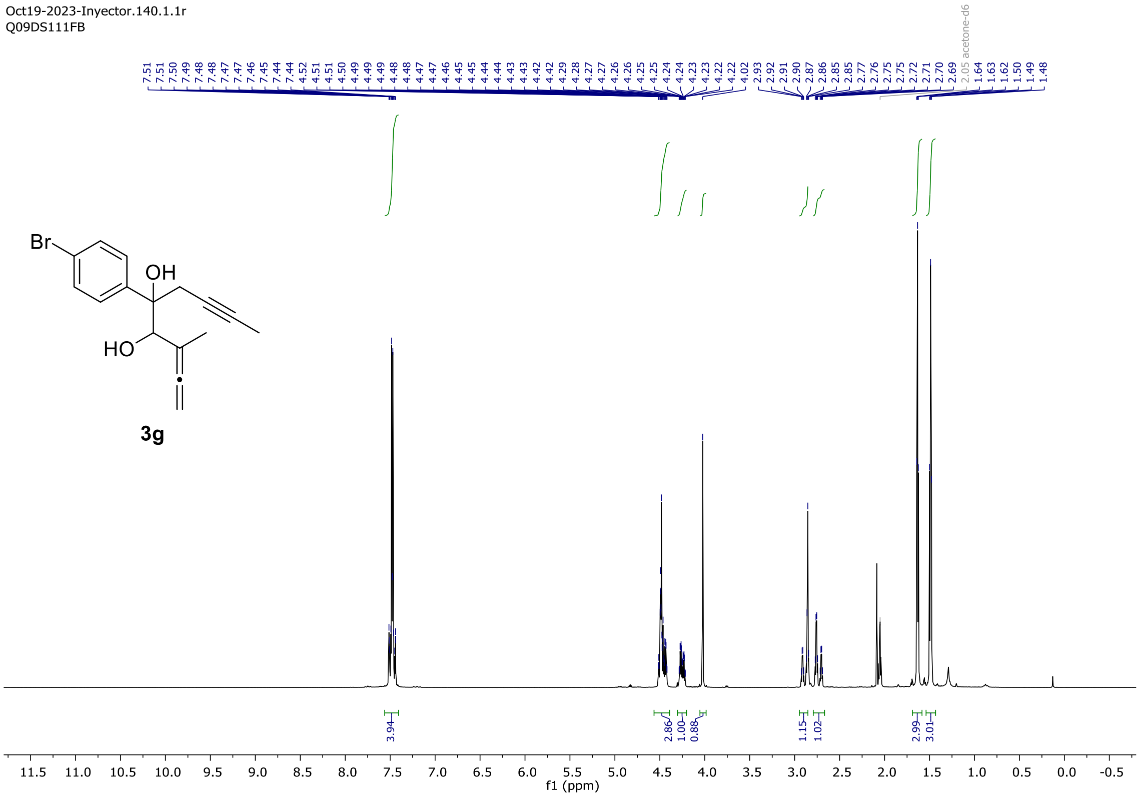

^{13}C NMR (75 MHz, acetone-d$_6$, rt)

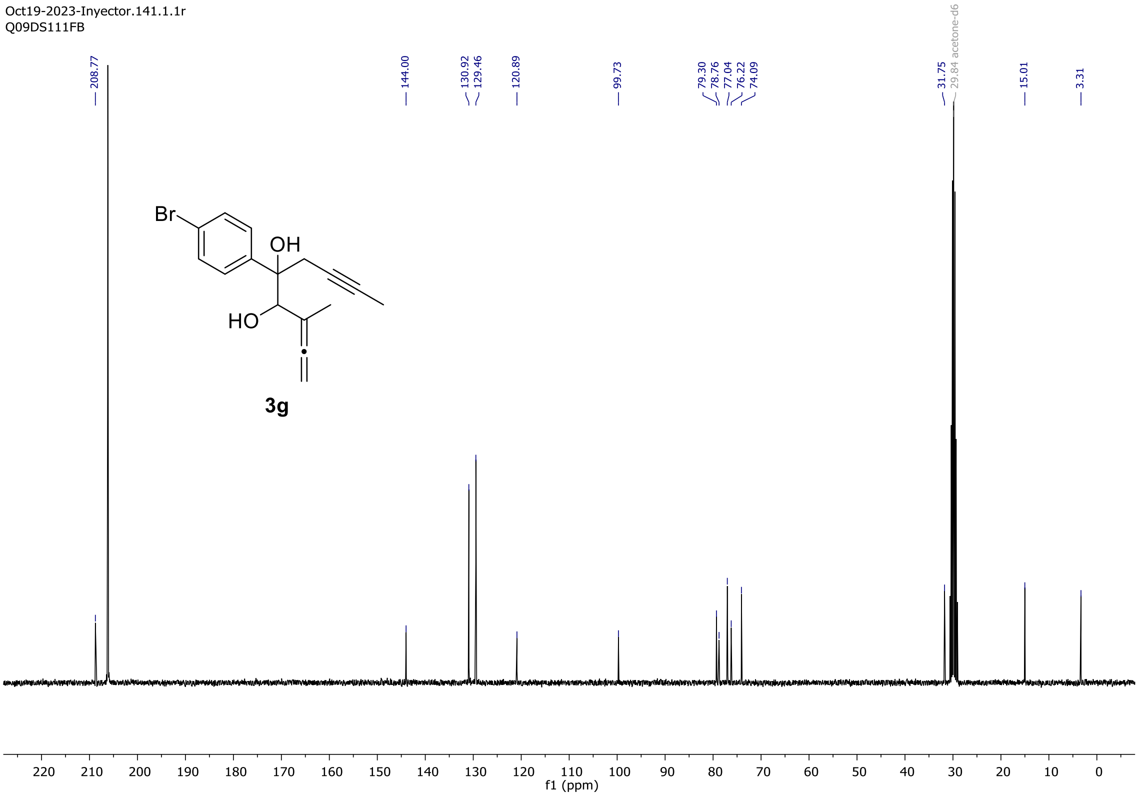

^{1}H NMR (300 MHz, acetone-d_6, rt)

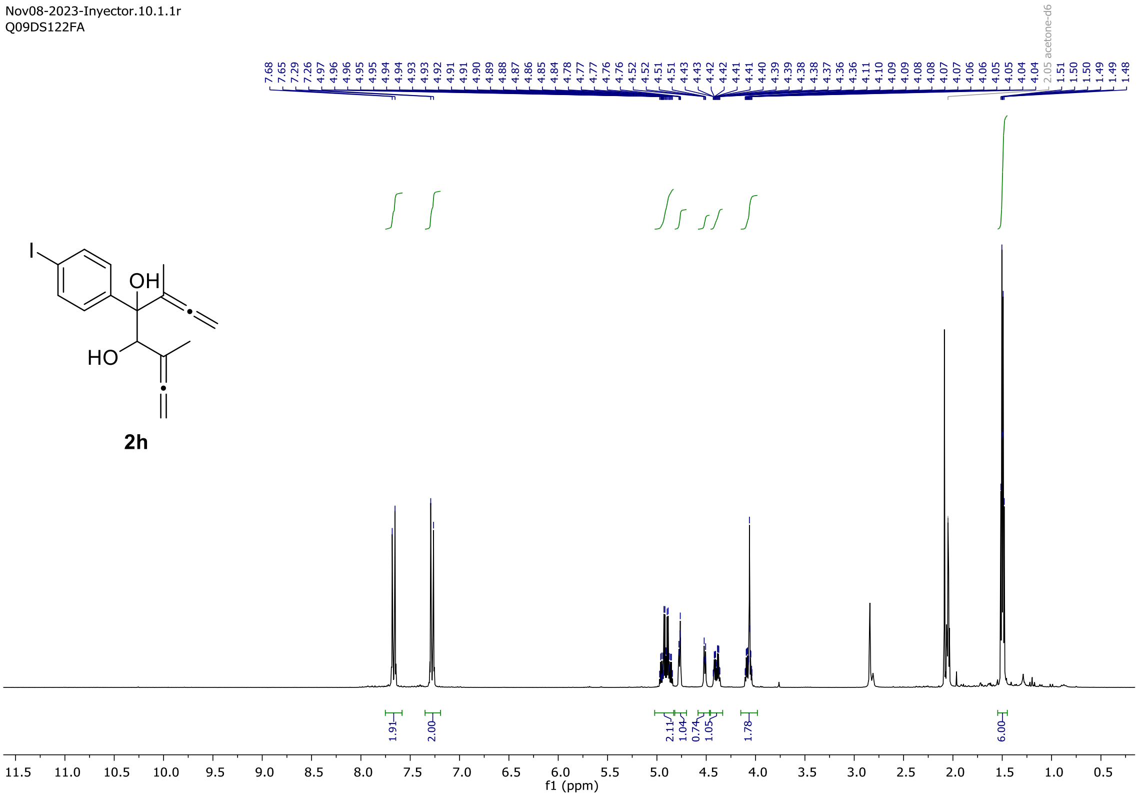

^{13}C NMR (75 MHz, acetone-d_6, rt)

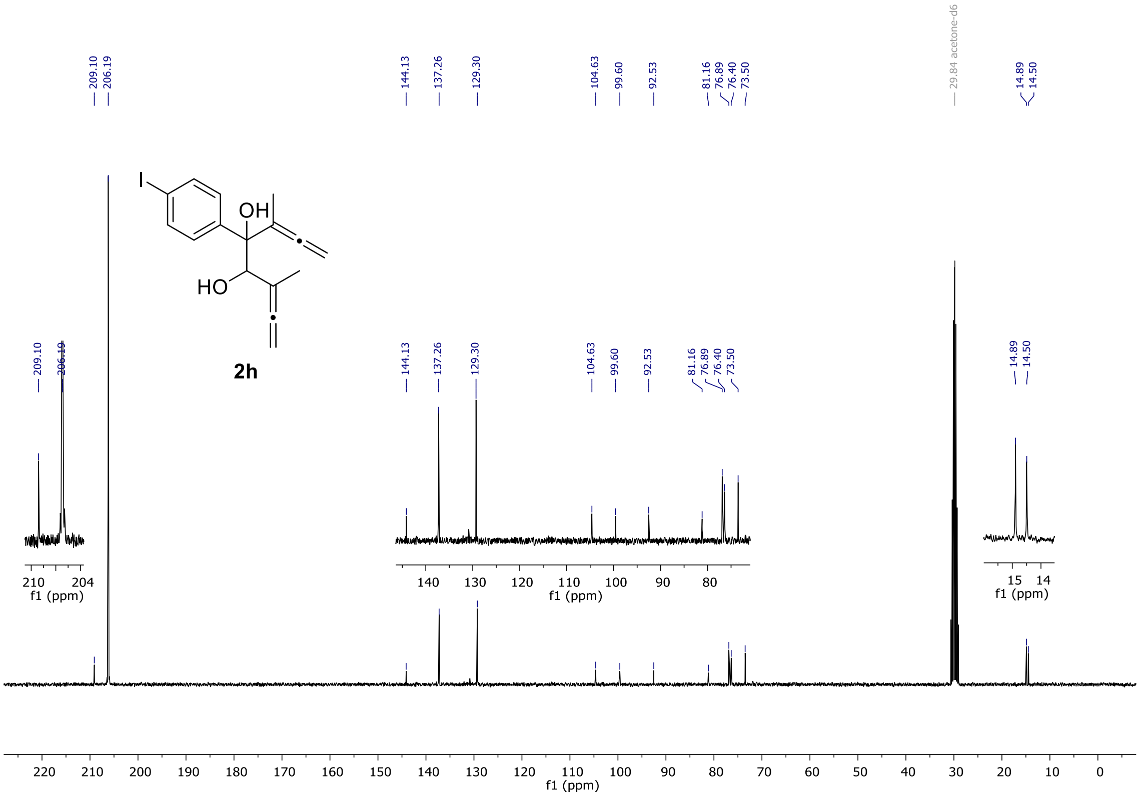

^{1}H NMR (300 MHz, acetone-d$_6$, rt)

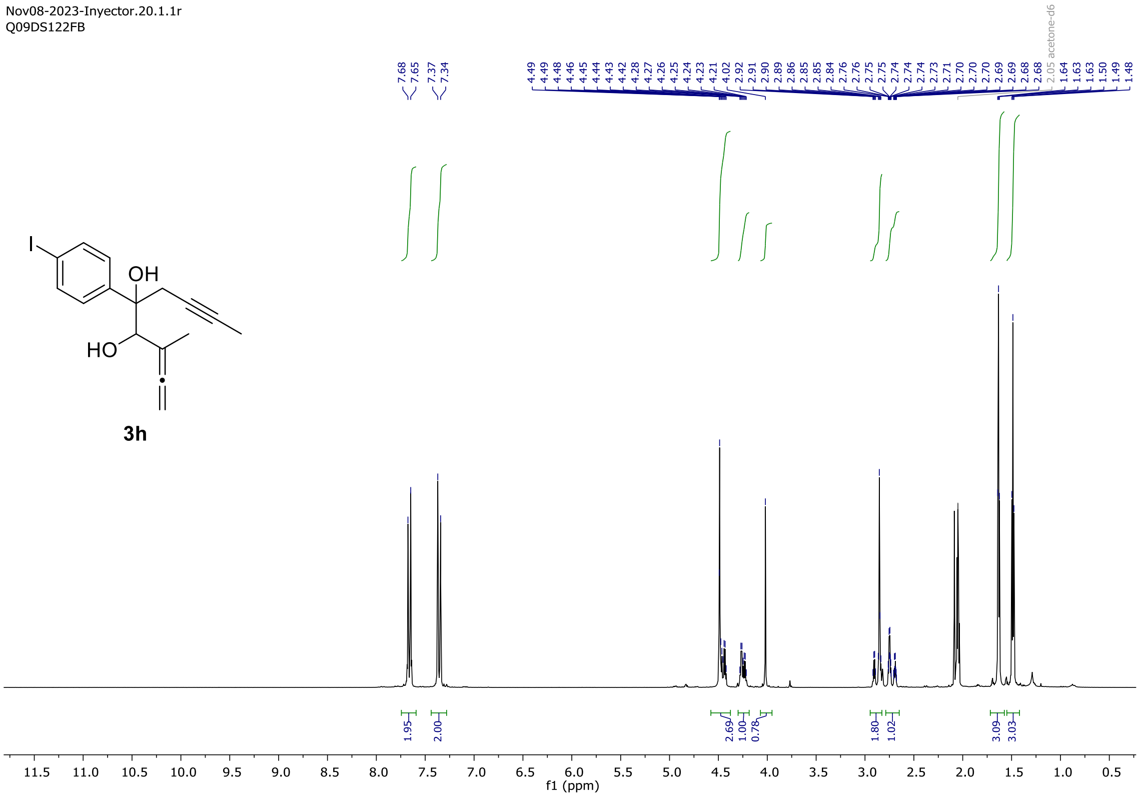

^{13}C NMR (75 MHz, acetone-d$_6$, rt)

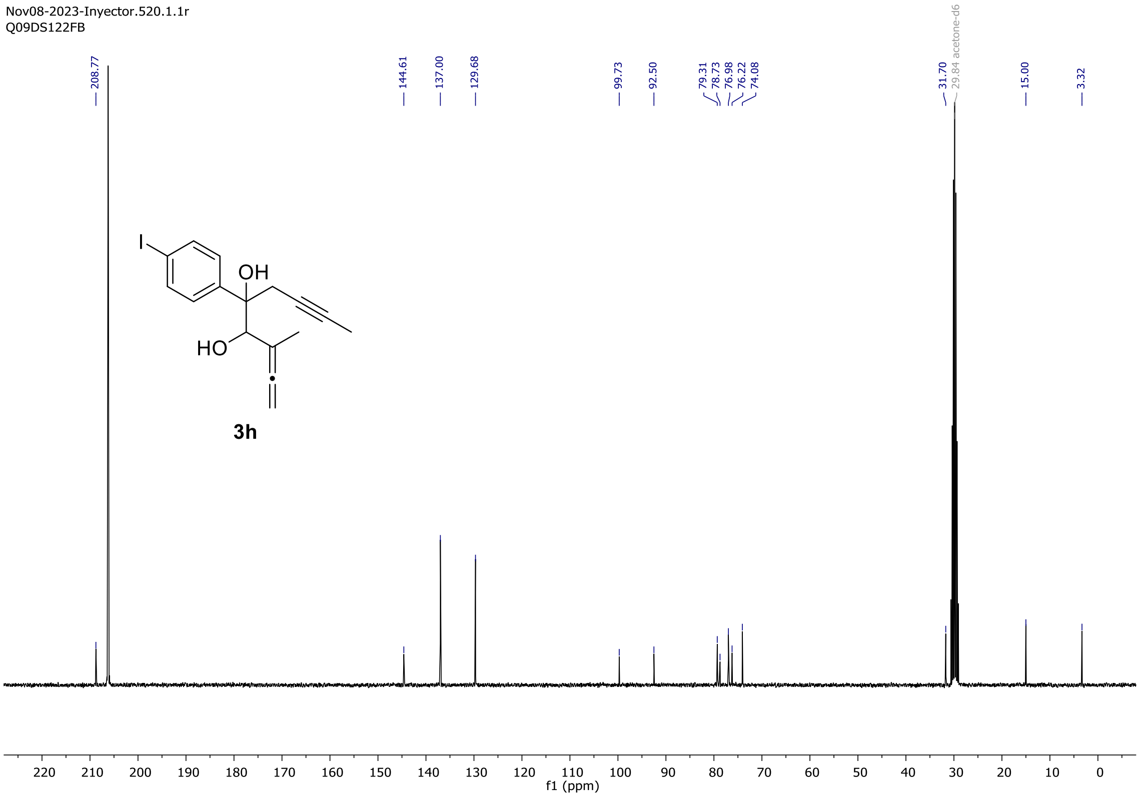

^{1}H NMR (300 MHz, CD_3CN, rt)

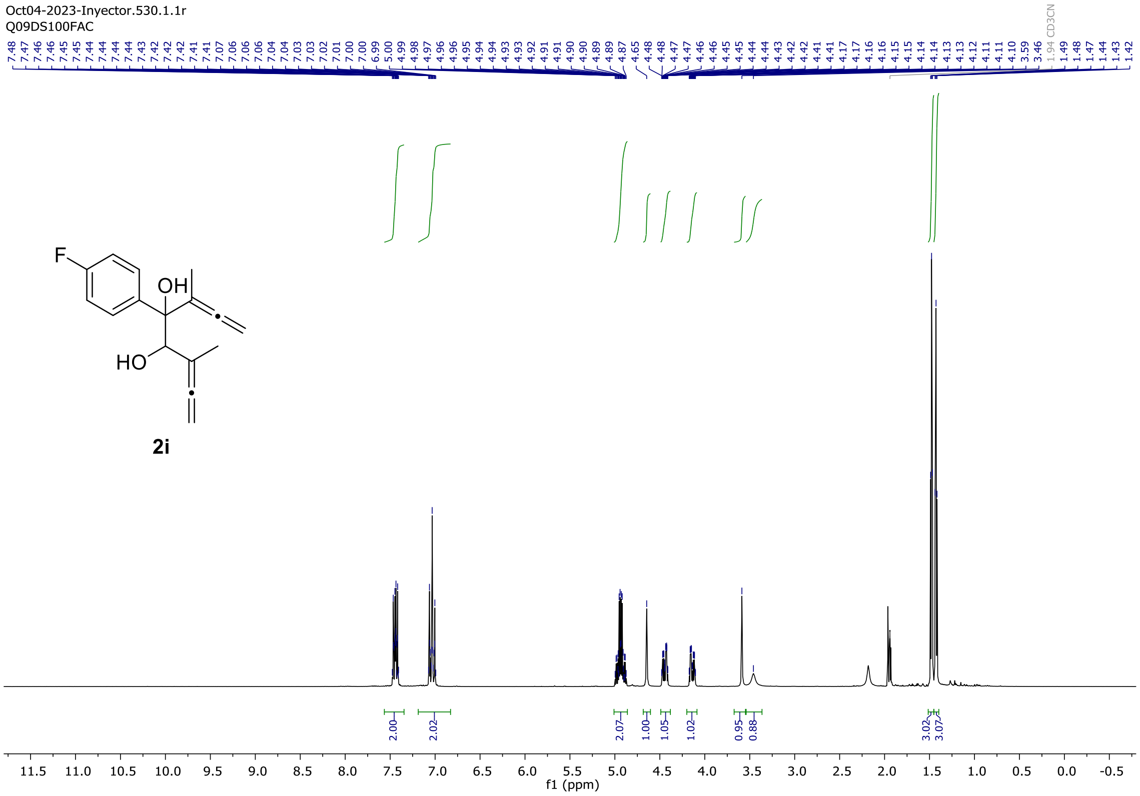

^{13}C NMR (75 MHz, CD_3CN, rt)

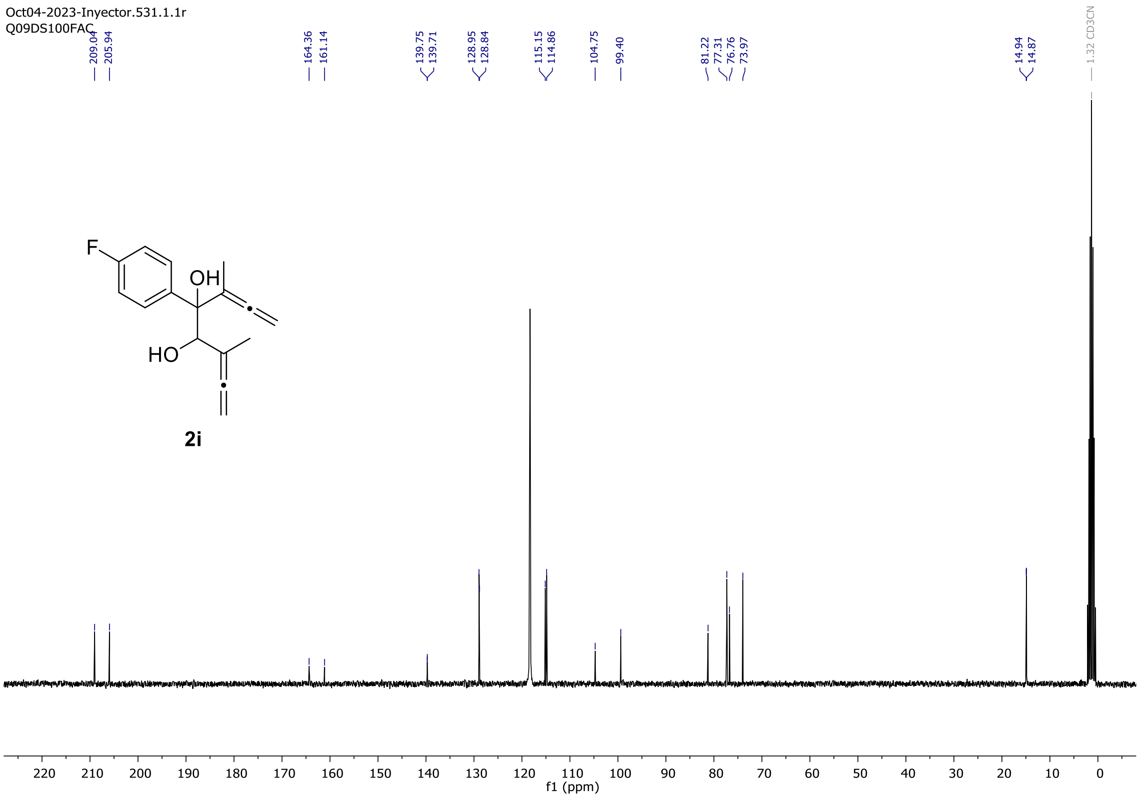

^{1}H NMR (300 MHz, CD_3CN, rt)

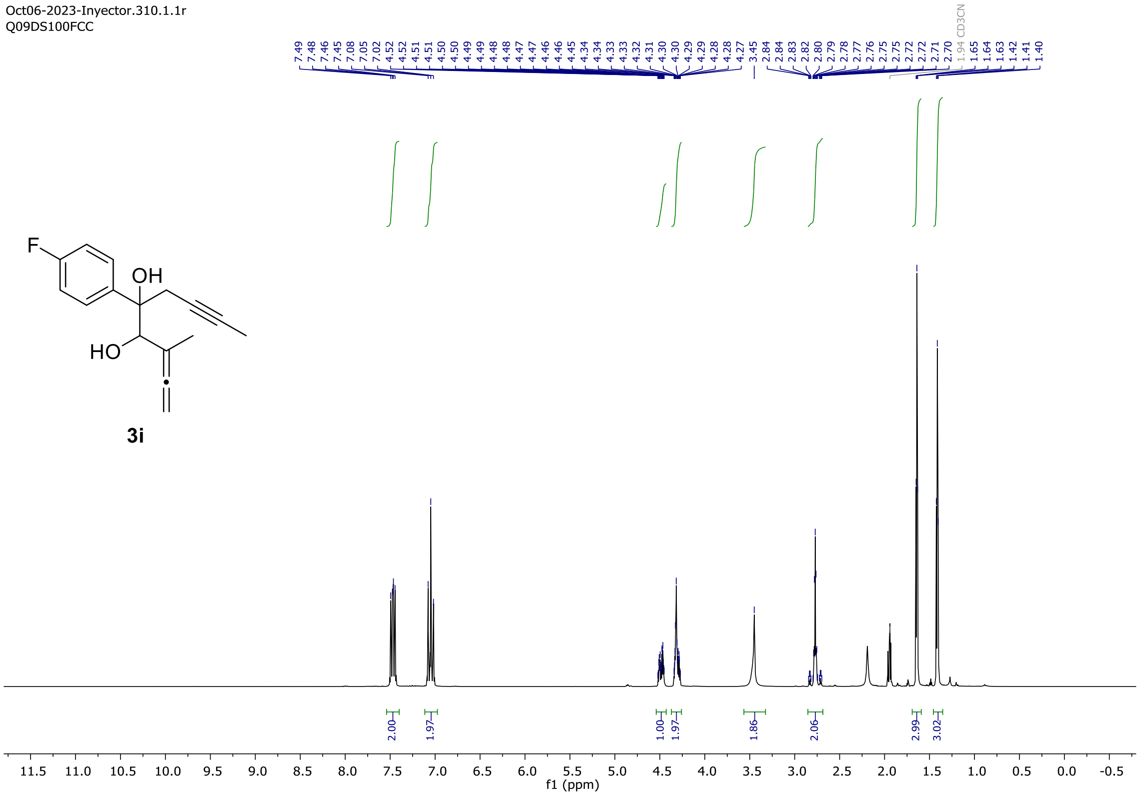

^{13}C NMR (75 MHz, CD_3CN, rt)

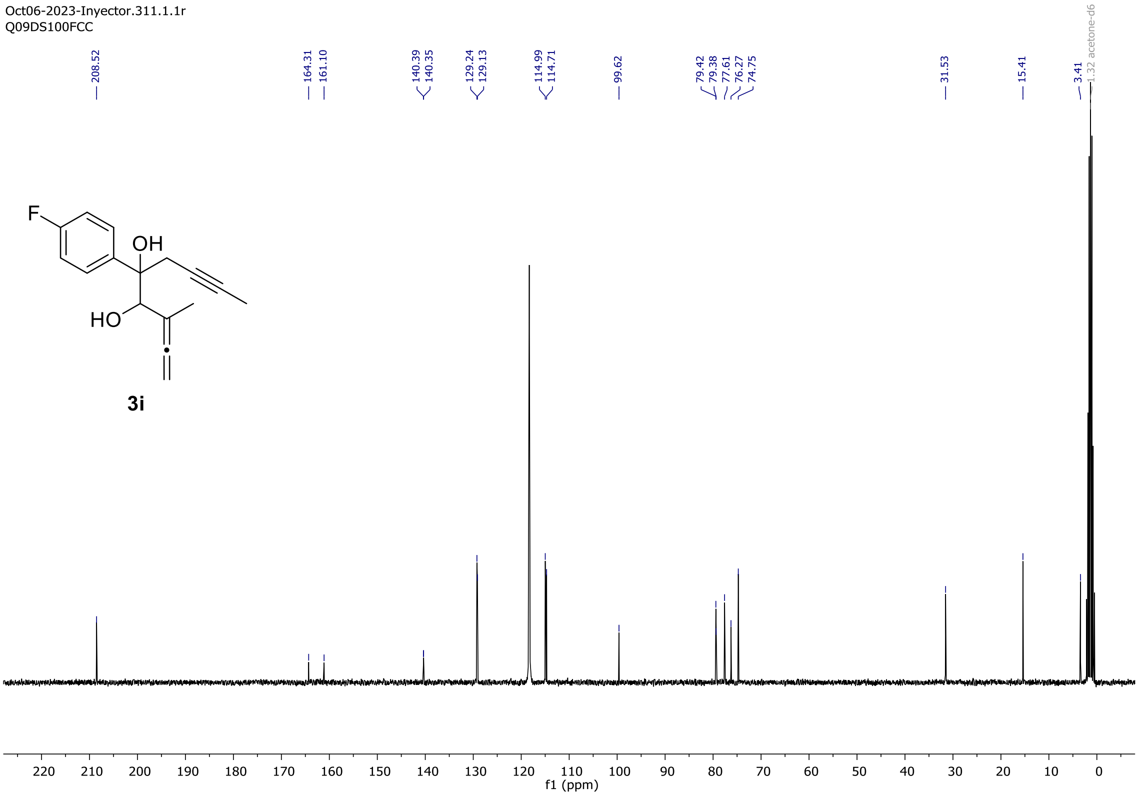

^{1}H NMR (300 MHz, acetone-d_6, rt)

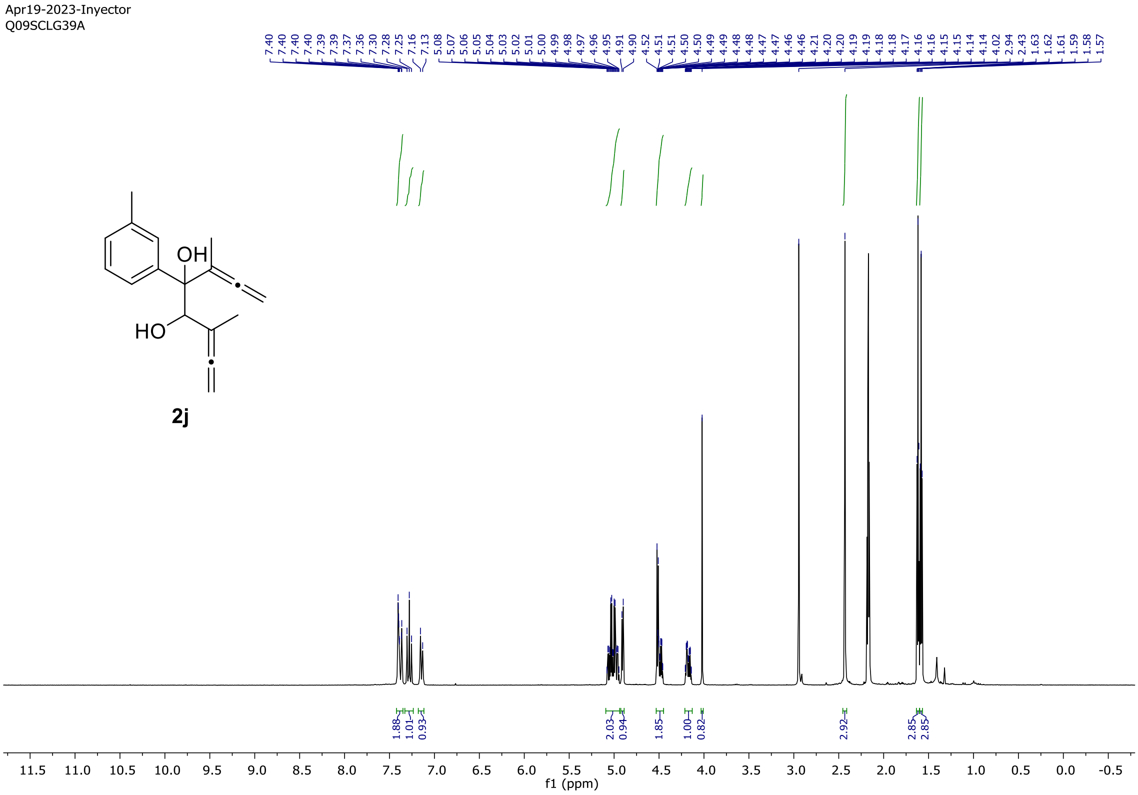

^{13}C NMR (75 MHz, acetone-d_6, rt)

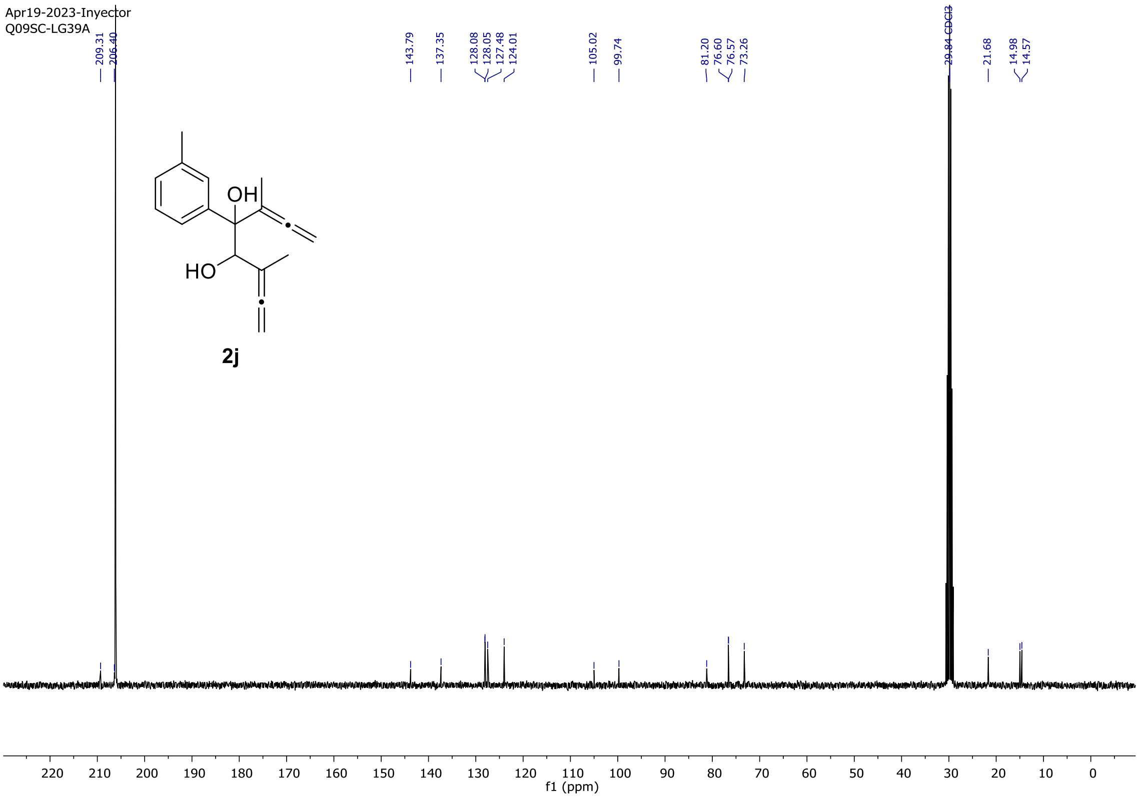

^{1}H NMR (300 MHz, acetone-d$_6$, rt)

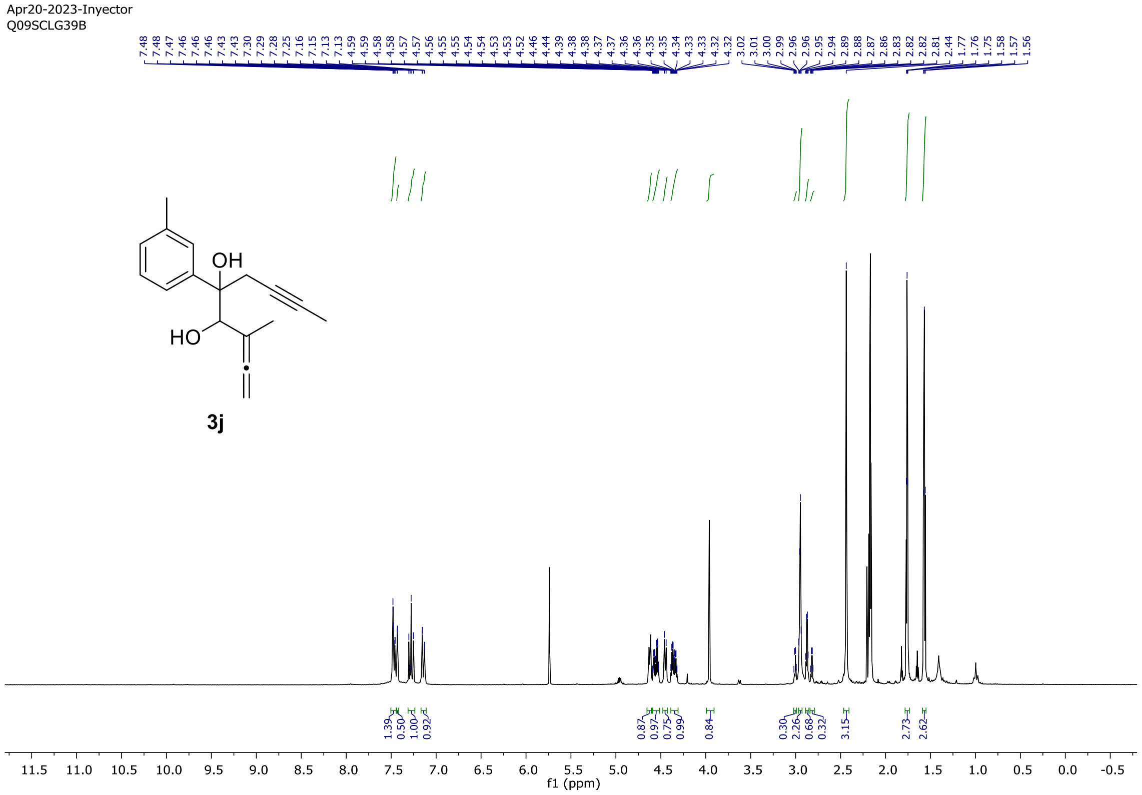

^{13}C NMR (75 MHz, acetone-d$_6$, rt)

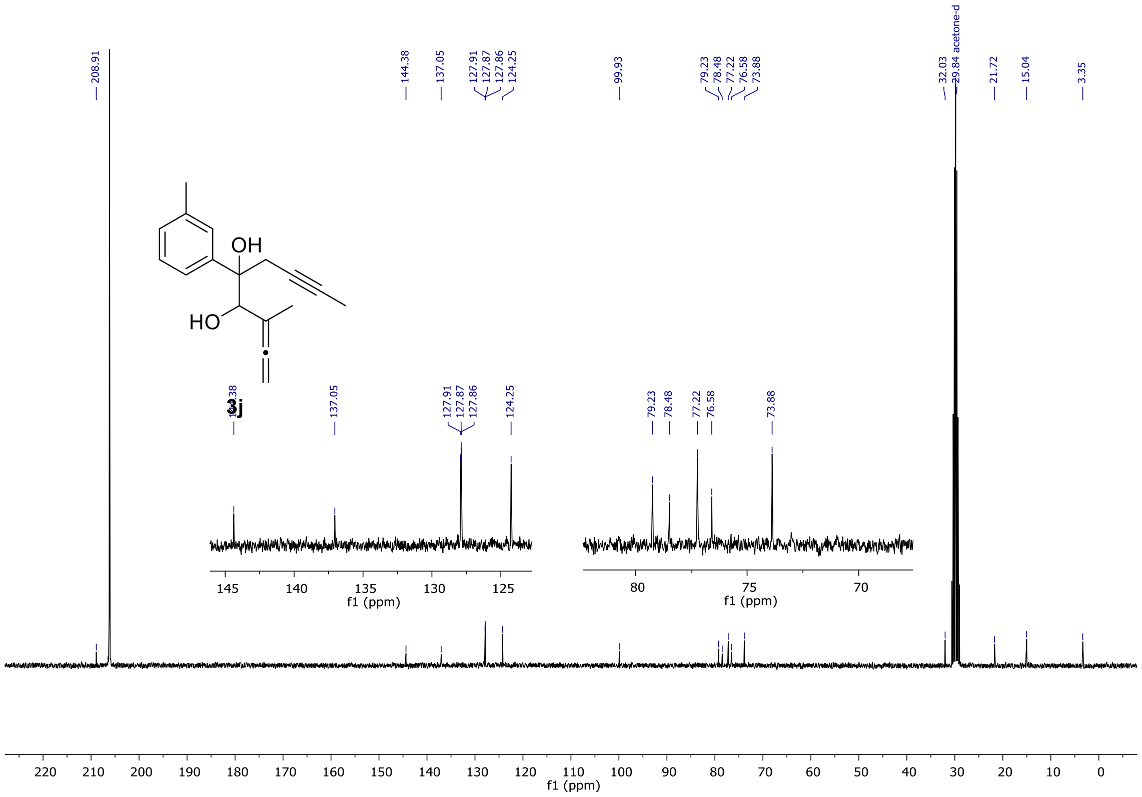

^{1}H NMR (300 MHz, acetone-d$_6$, rt)

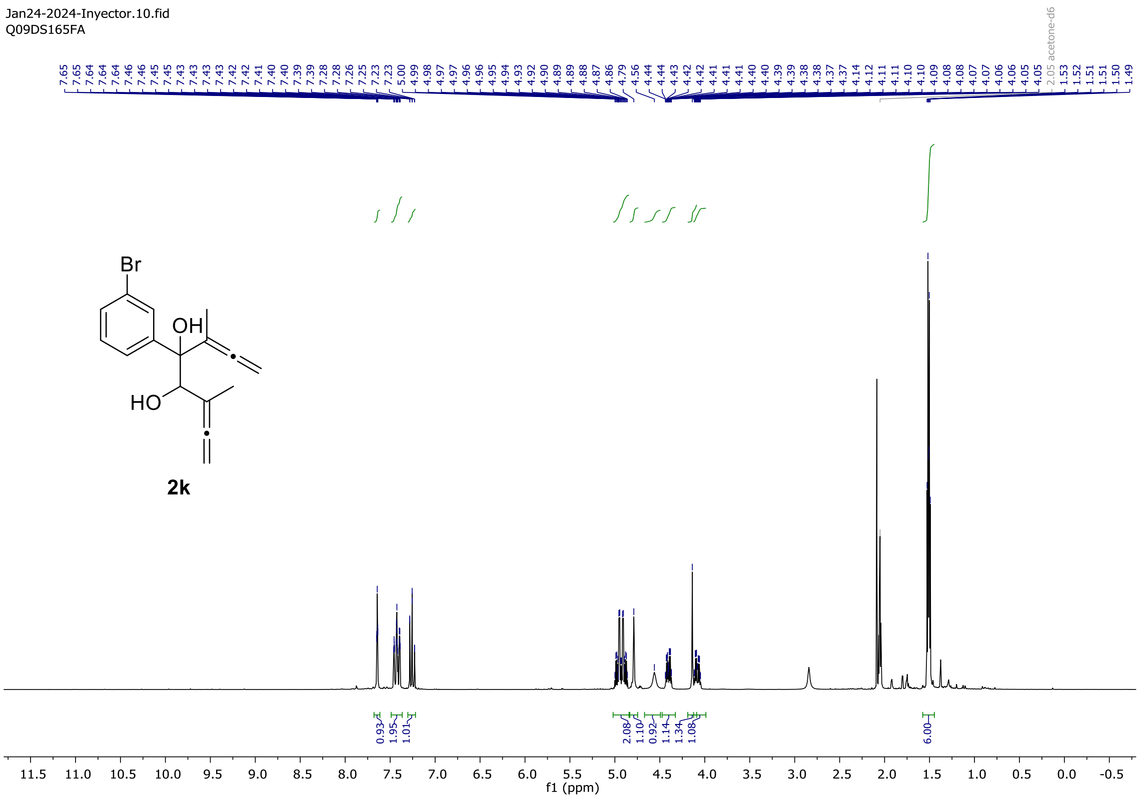

^{13}C NMR (75 MHz, acetone-d$_6$, rt)

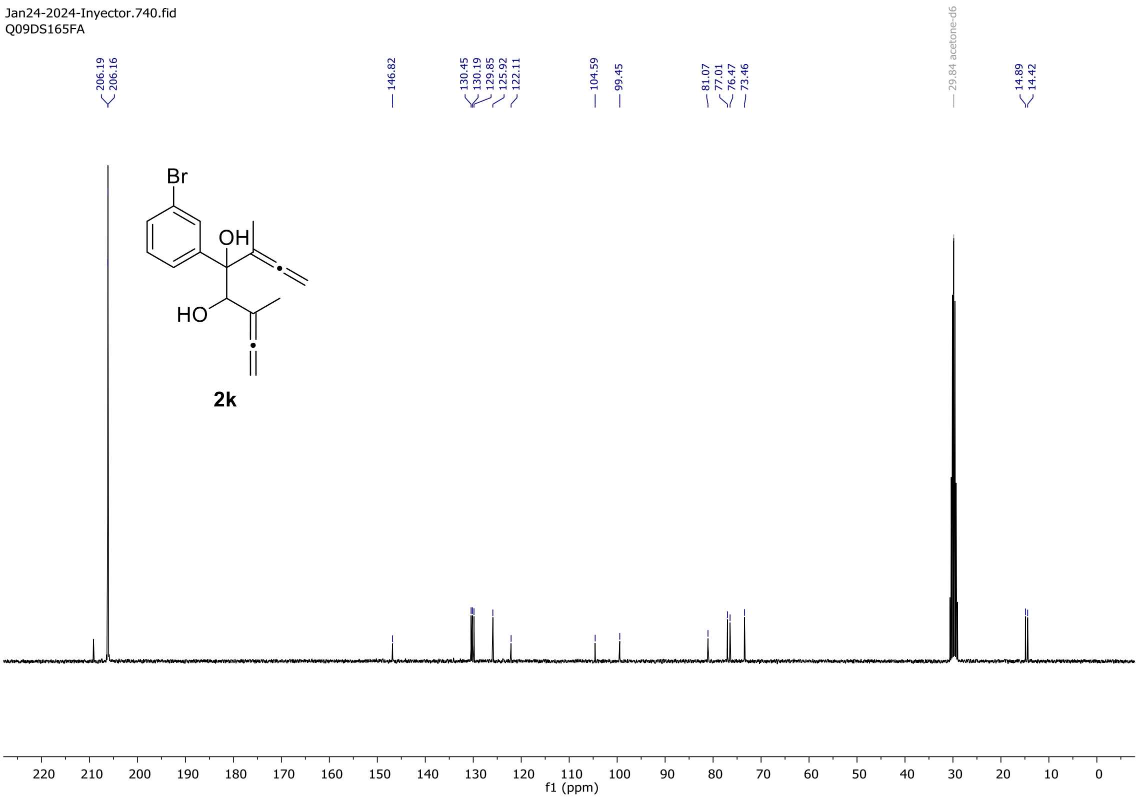

^{1}H NMR (300 MHz, acetone-d$_6$, rt)

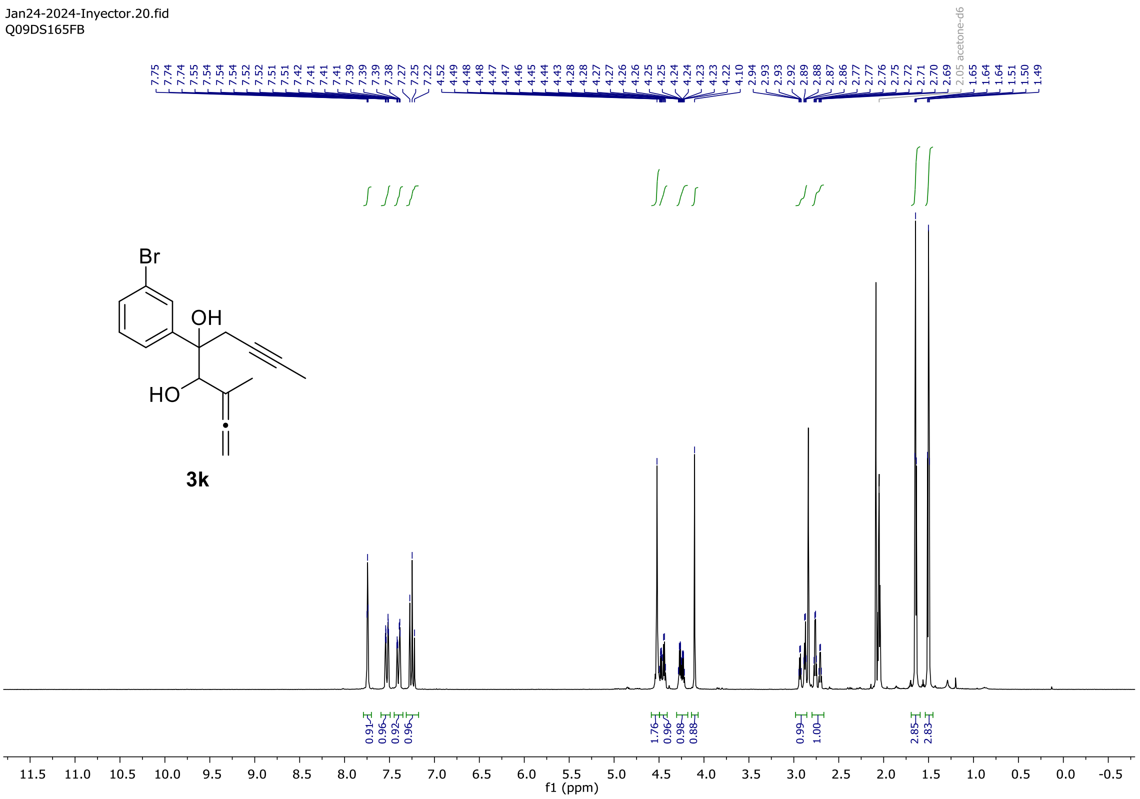

^{13}C NMR (75 MHz, acetone-d$_6$, rt)

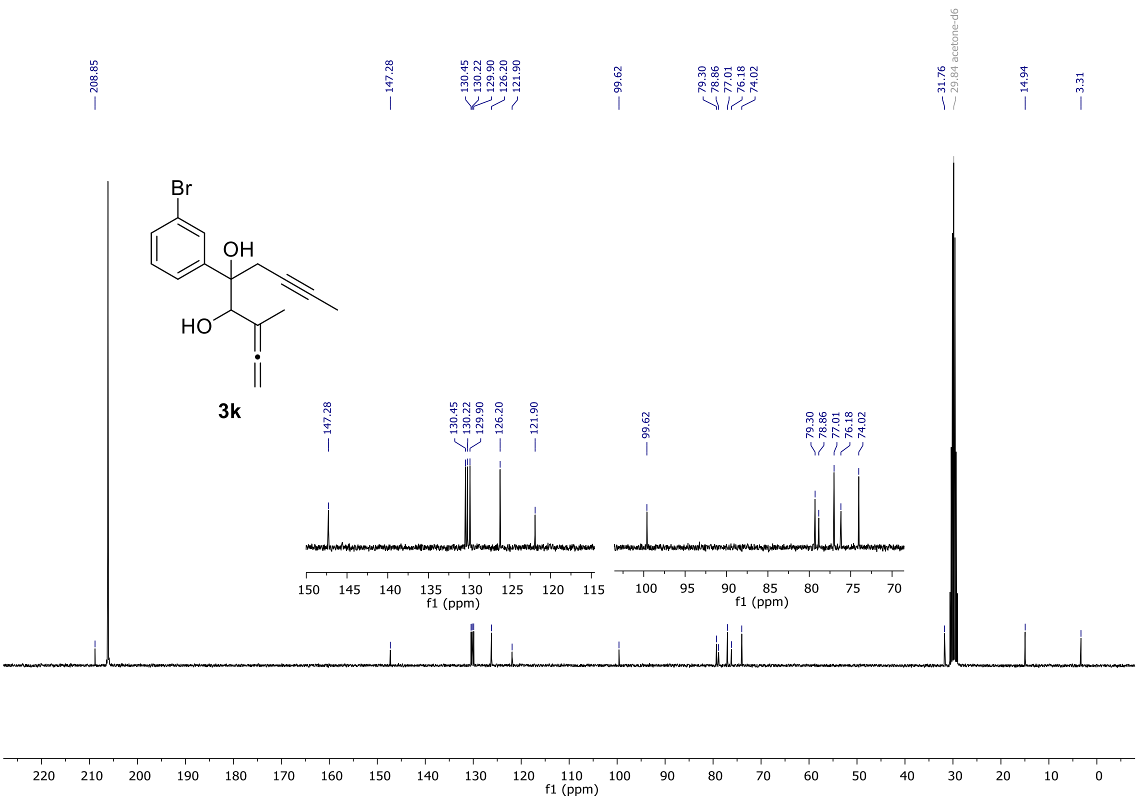

^{1}H NMR (300 MHz, acetone-d$_6$, rt)

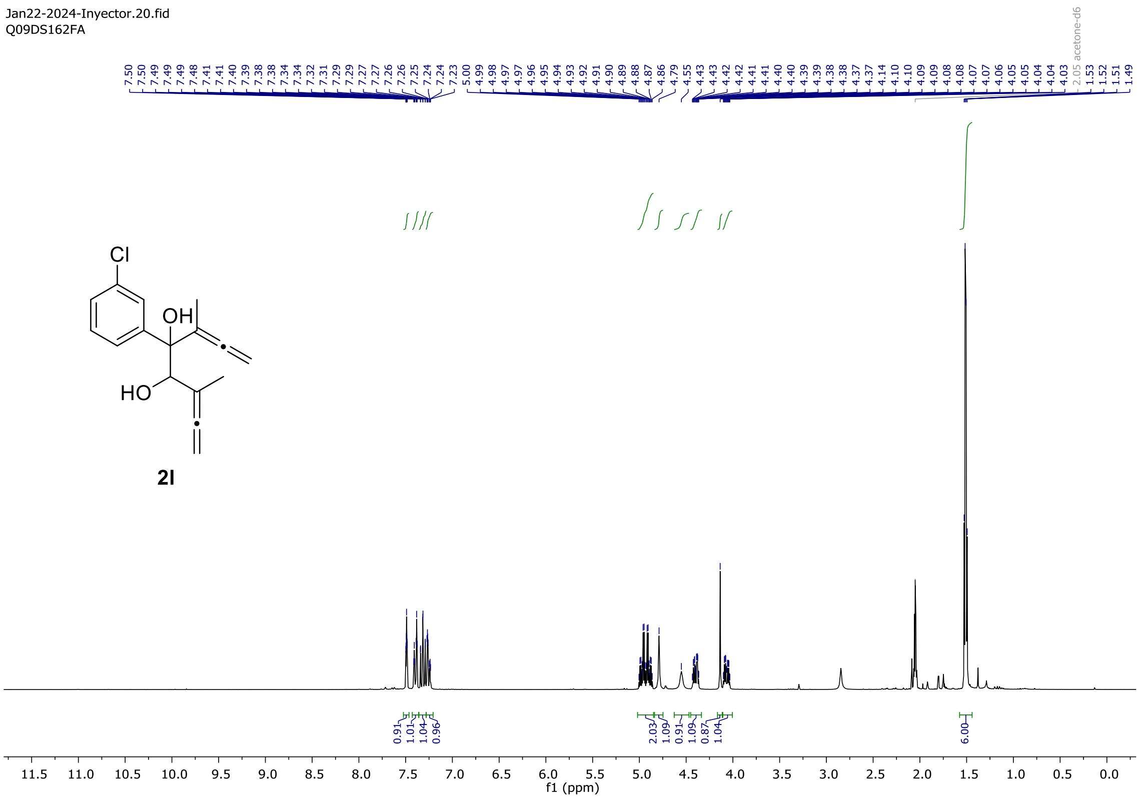

^{13}C NMR (75 MHz, acetone-d$_6$, rt)

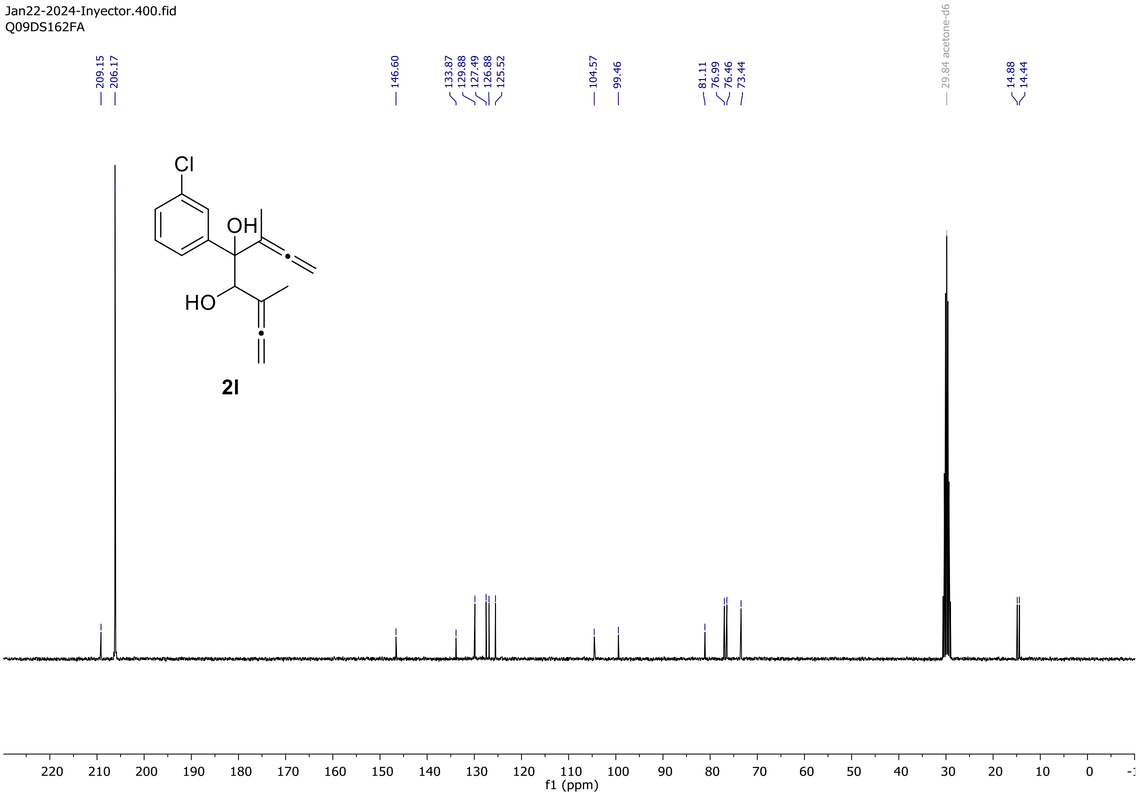

^{1}H NMR (300 MHz, acetone-d$_6$, rt)

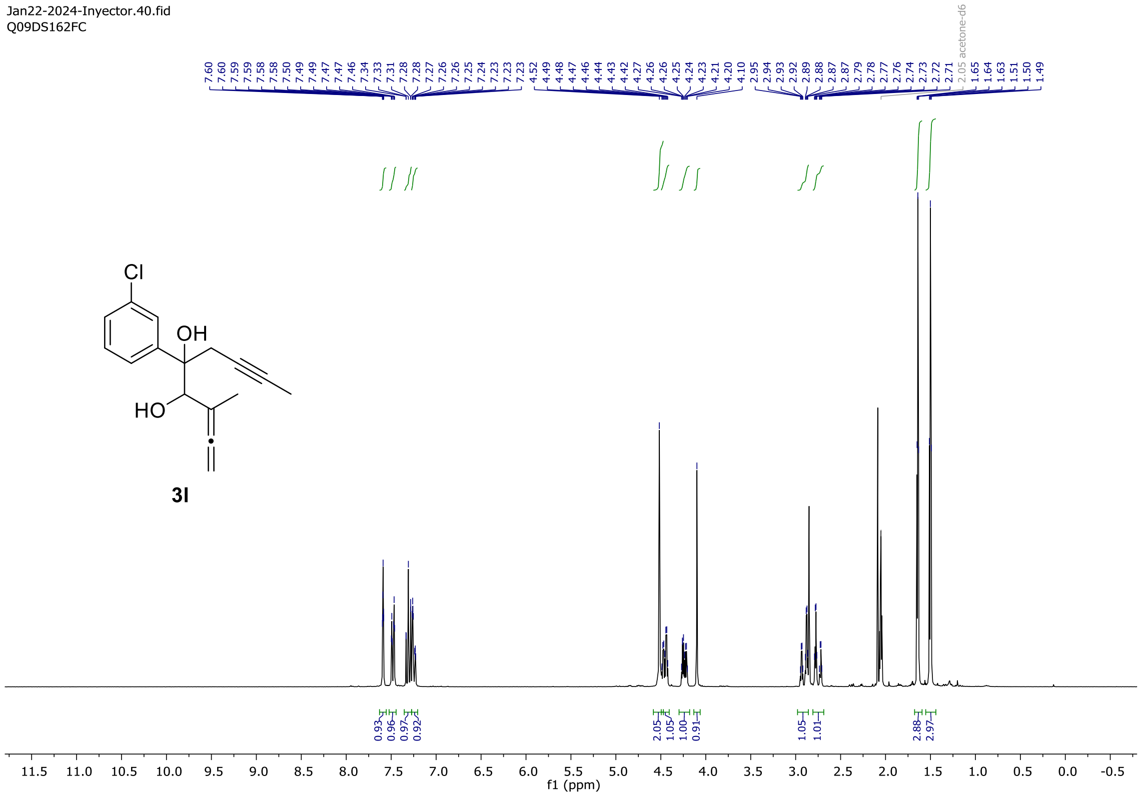

^{13}C NMR (75 MHz, acetone-d$_6$, rt)

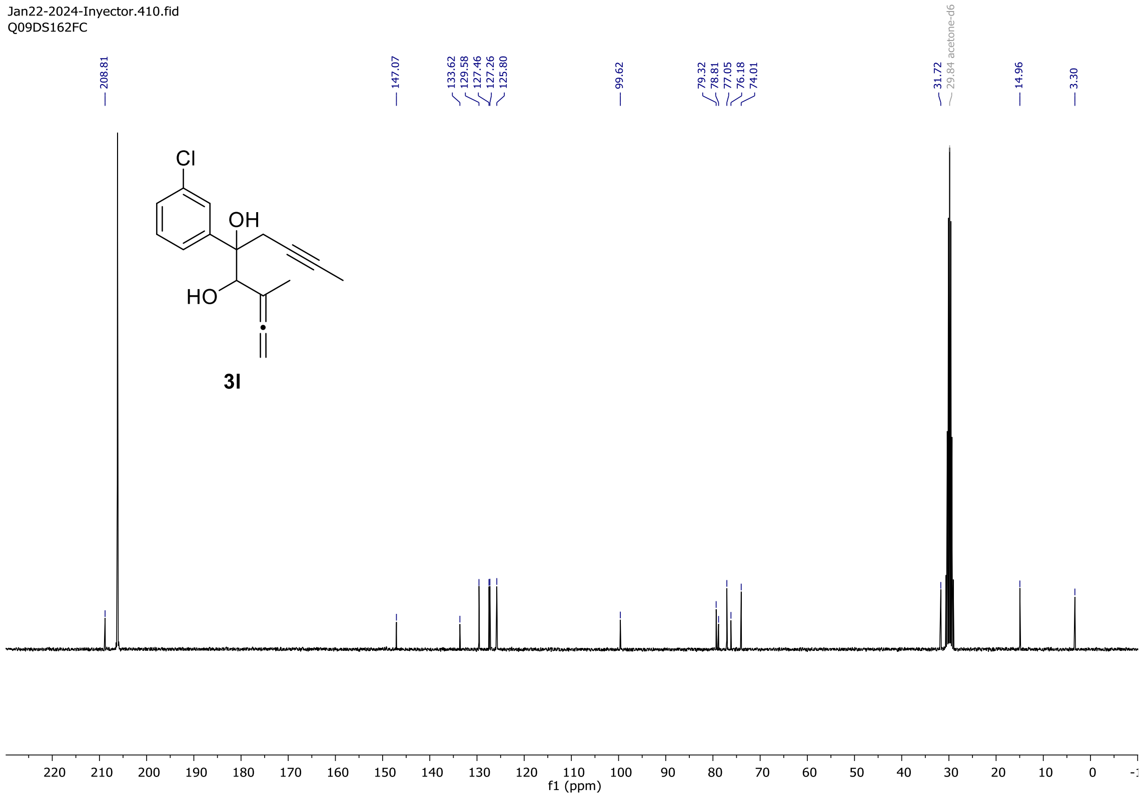

^{1}H NMR (300 MHz, acetone-d$_6$, rt)

Jan17-2024-Inyector.490.fid
Q09DS152FA

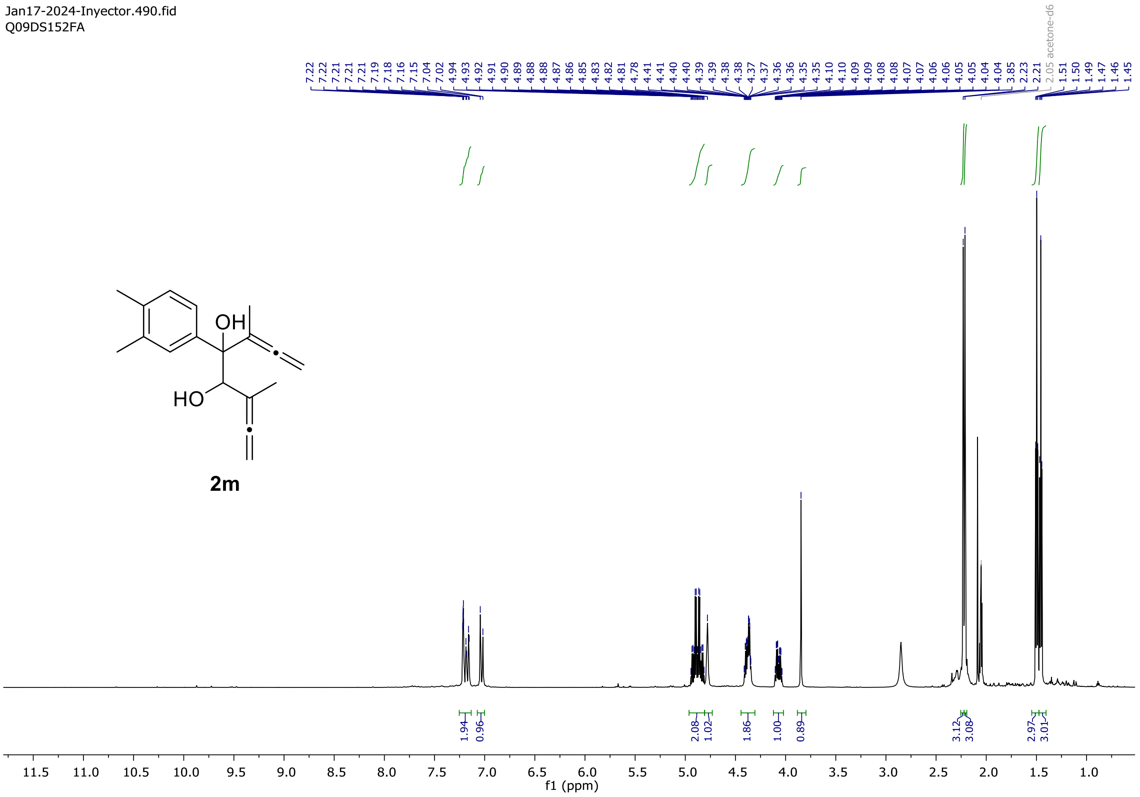

^{13}C NMR (75 MHz, acetone-d$_6$, rt)

Jan17-2024-Inyector.491.fid
Q09DS152FA

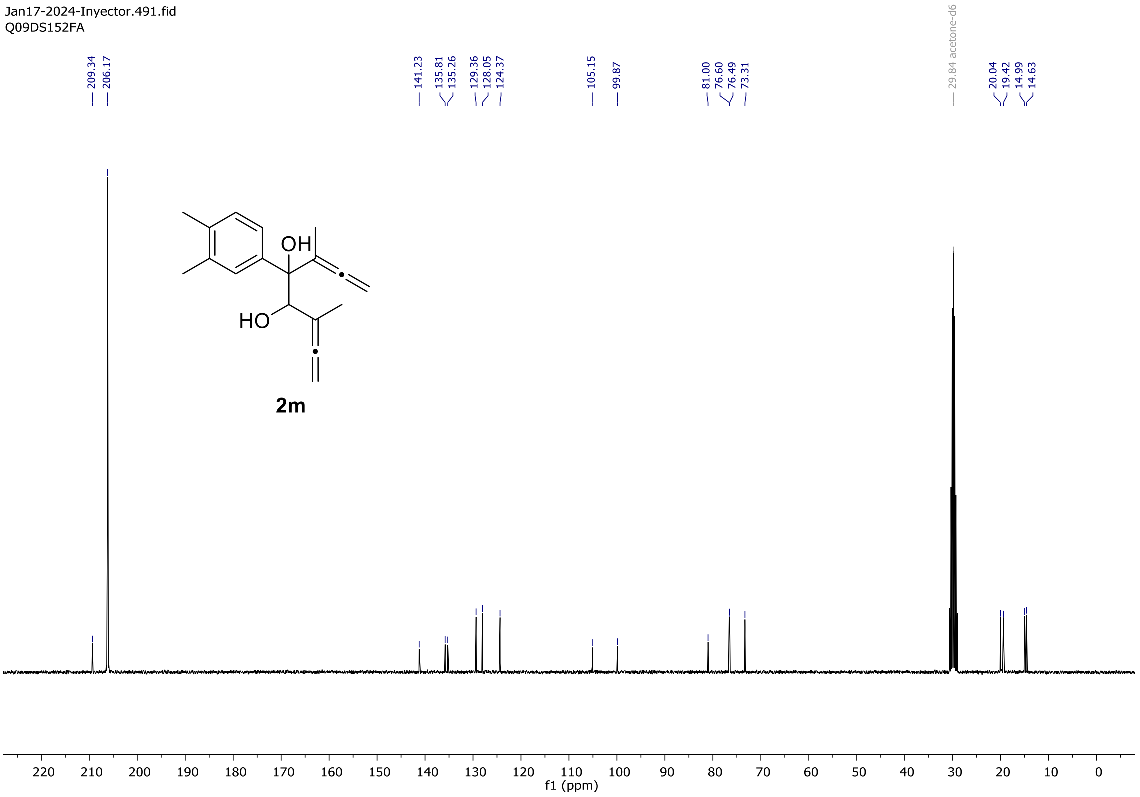

^{1}H NMR (300 MHz, acetone-d$_6$, rt)

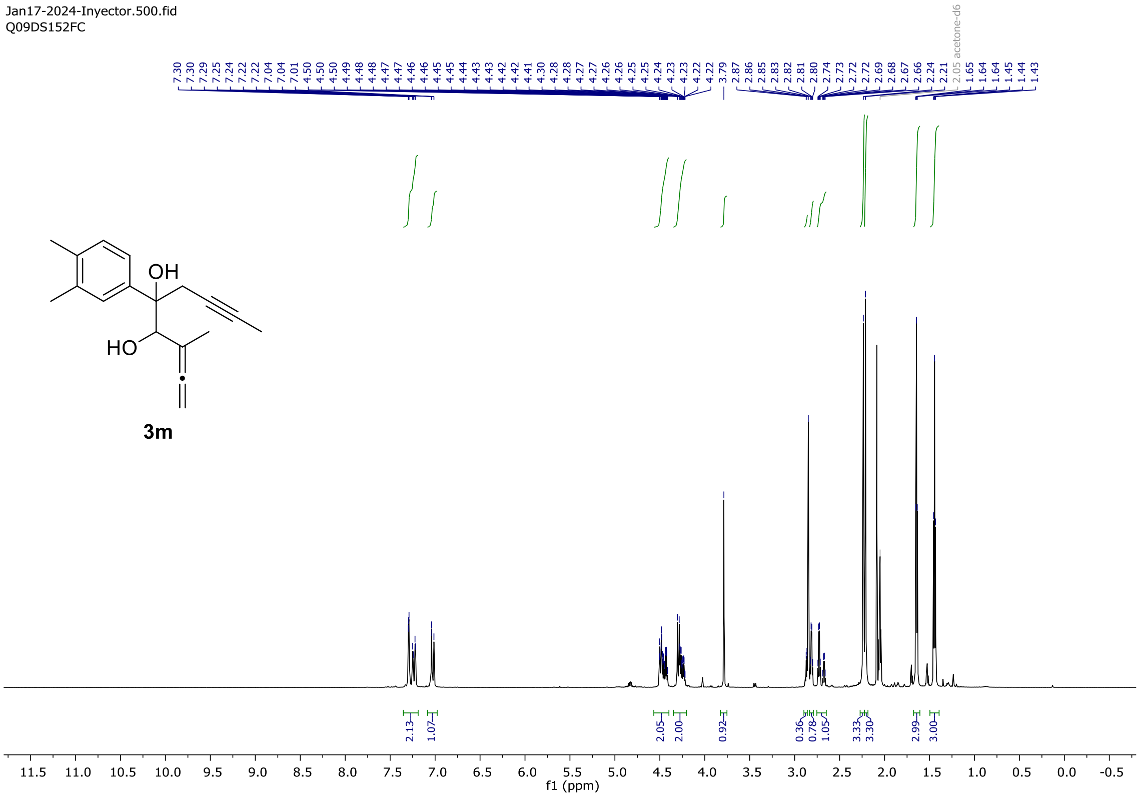

^{13}C NMR (75 MHz, acetone-d$_6$, rt)

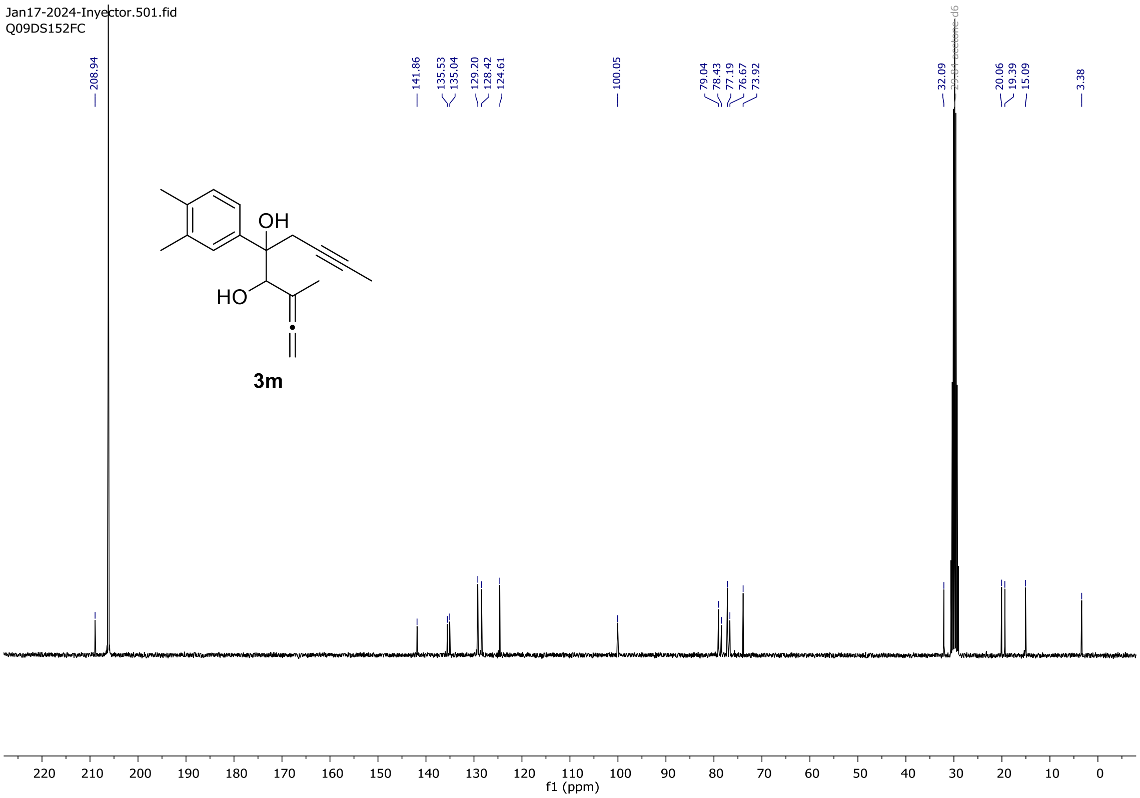

^{1}H NMR (300 MHz, acetone-d_6, rt)

Jan31-2024-Inyector.550.fid
Q09DS170FA

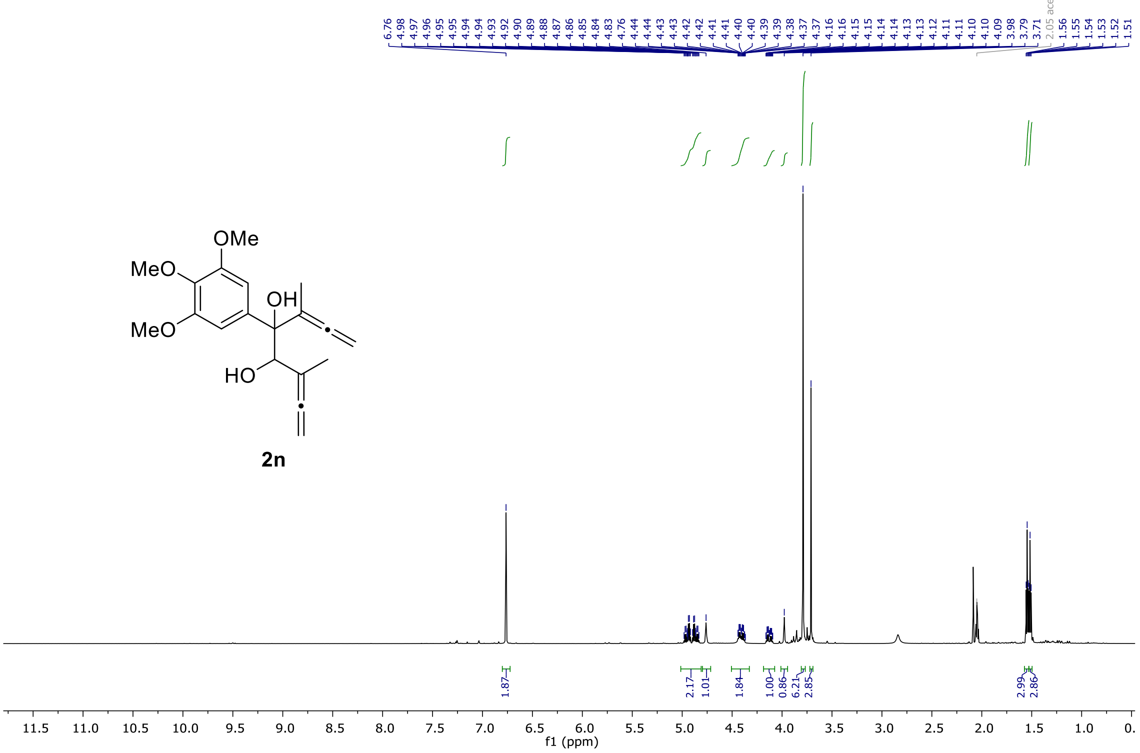

^{13}C NMR (75 MHz, acetone-d_6, rt)

Jan31-2024-Inyector.551.fid
Q09DS170FA

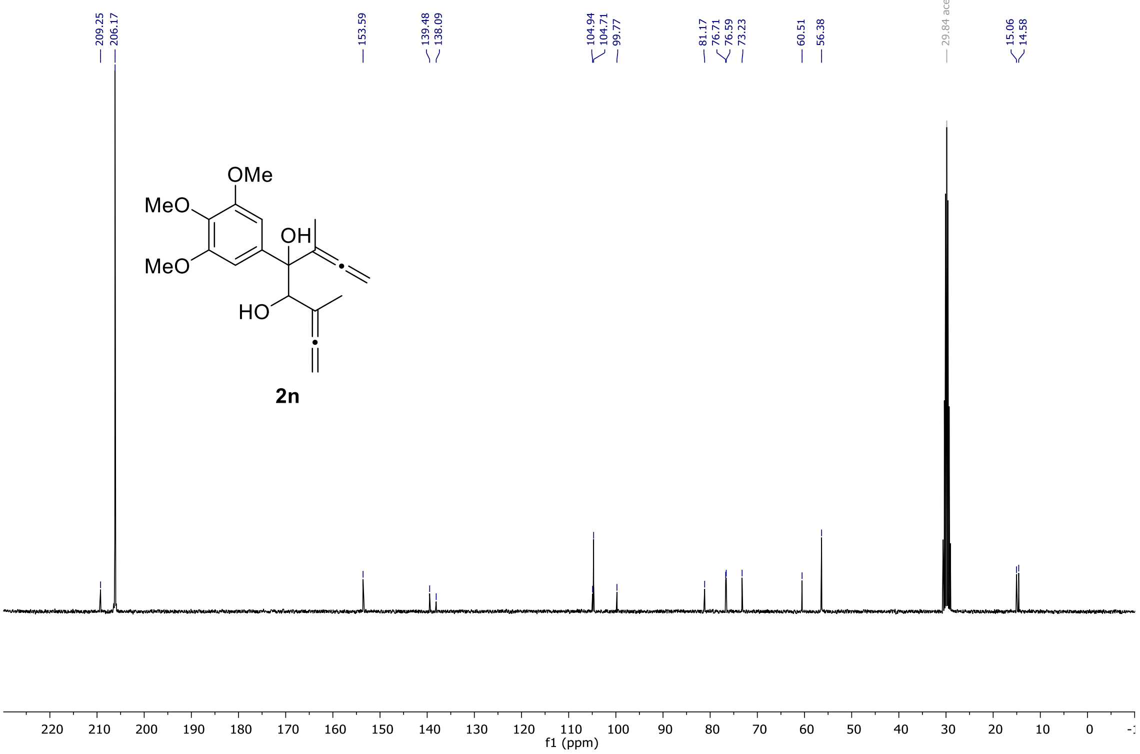

^1H NMR (300 MHz, acetone-d$_6$, rt)

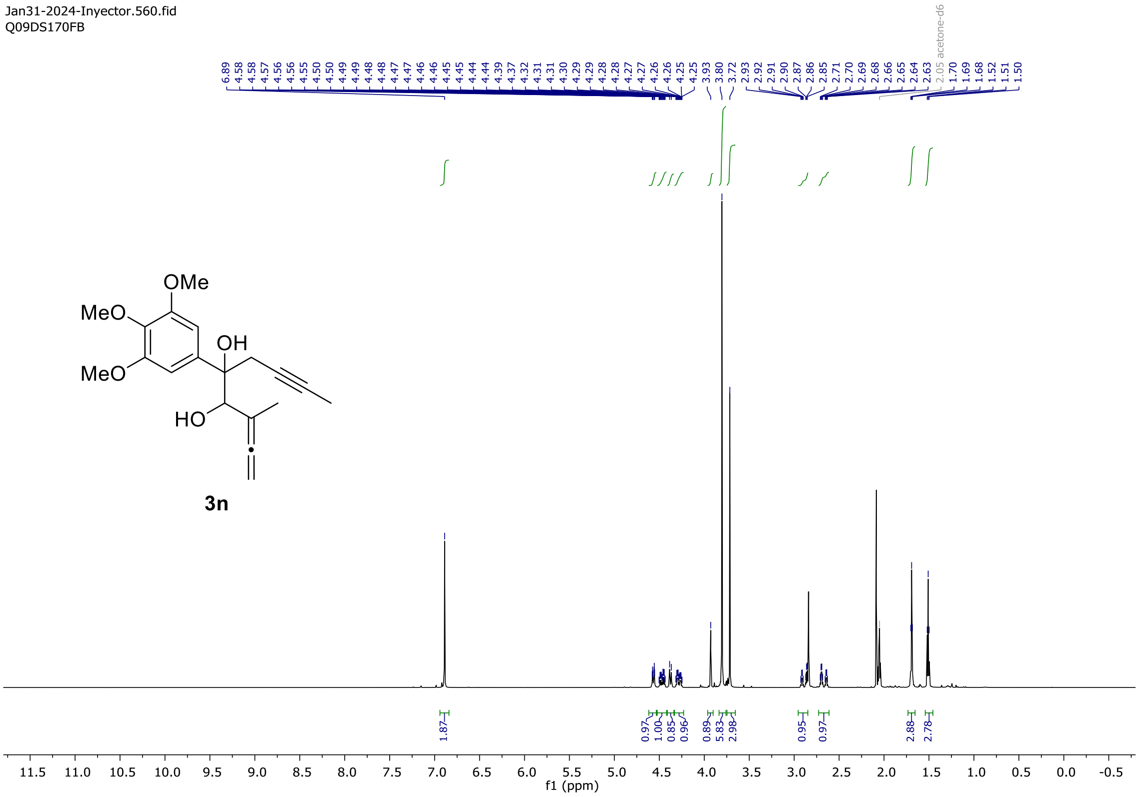

^{13}C NMR (75 MHz, acetone-d$_6$, rt)

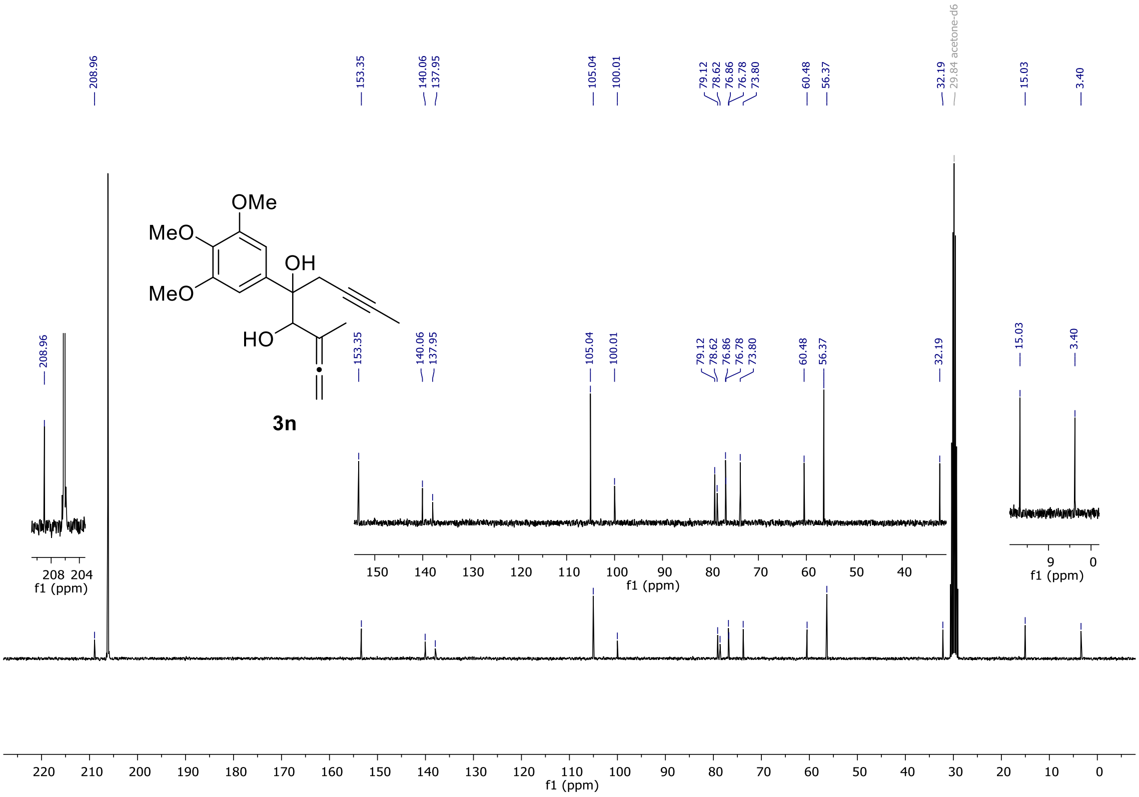

^{1}H NMR (300 MHz, acetone-d$_6$, rt)

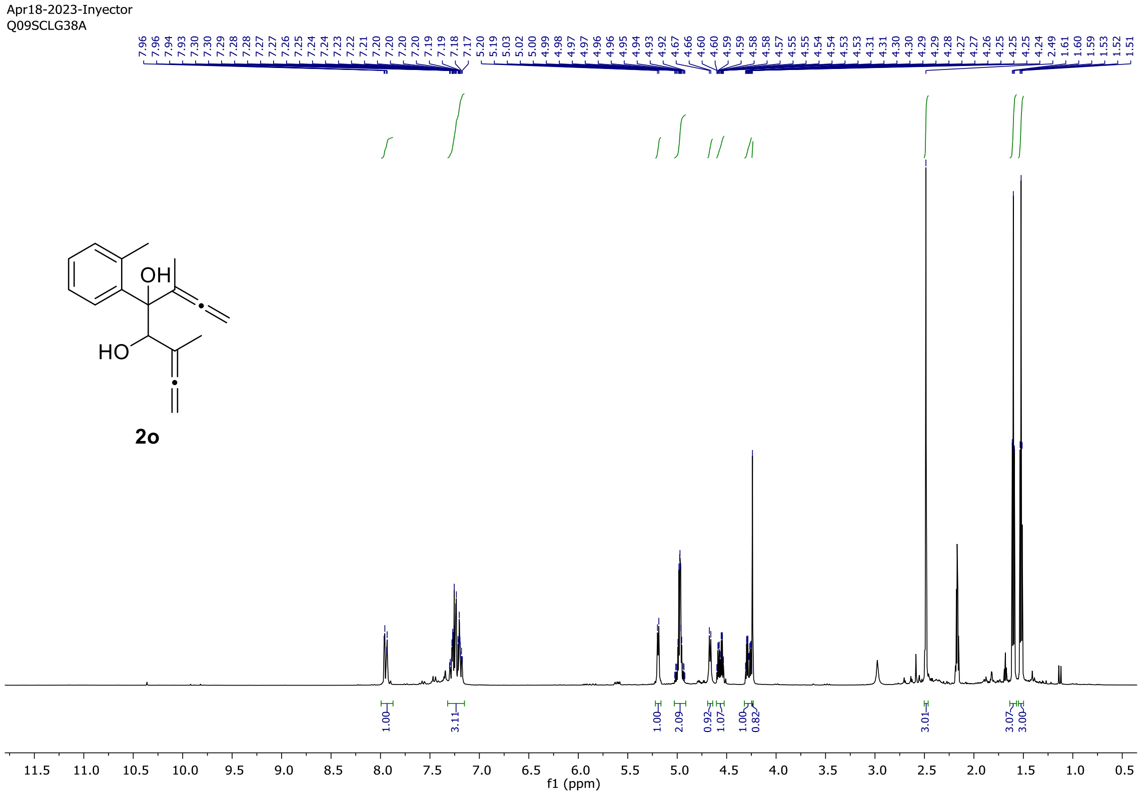

^{13}C NMR (75 MHz, acetone-d$_6$, rt)

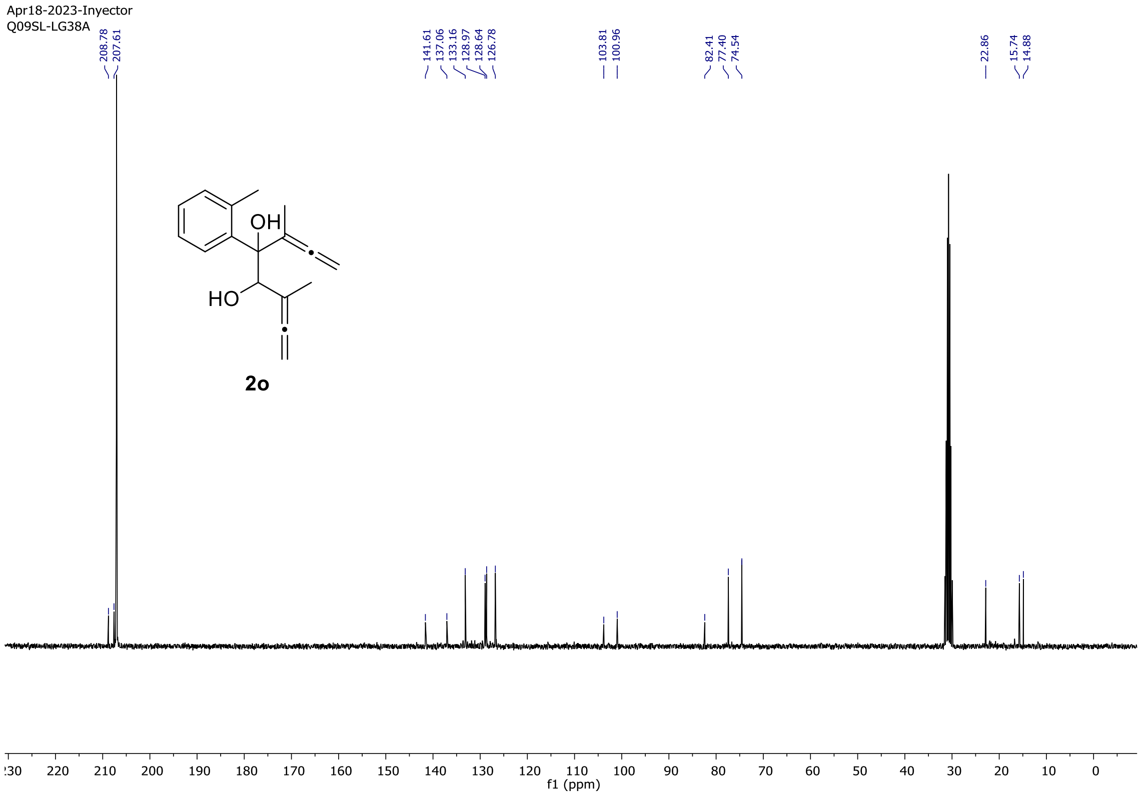

^{1}H NMR (300 MHz, acetone-d6, rt)

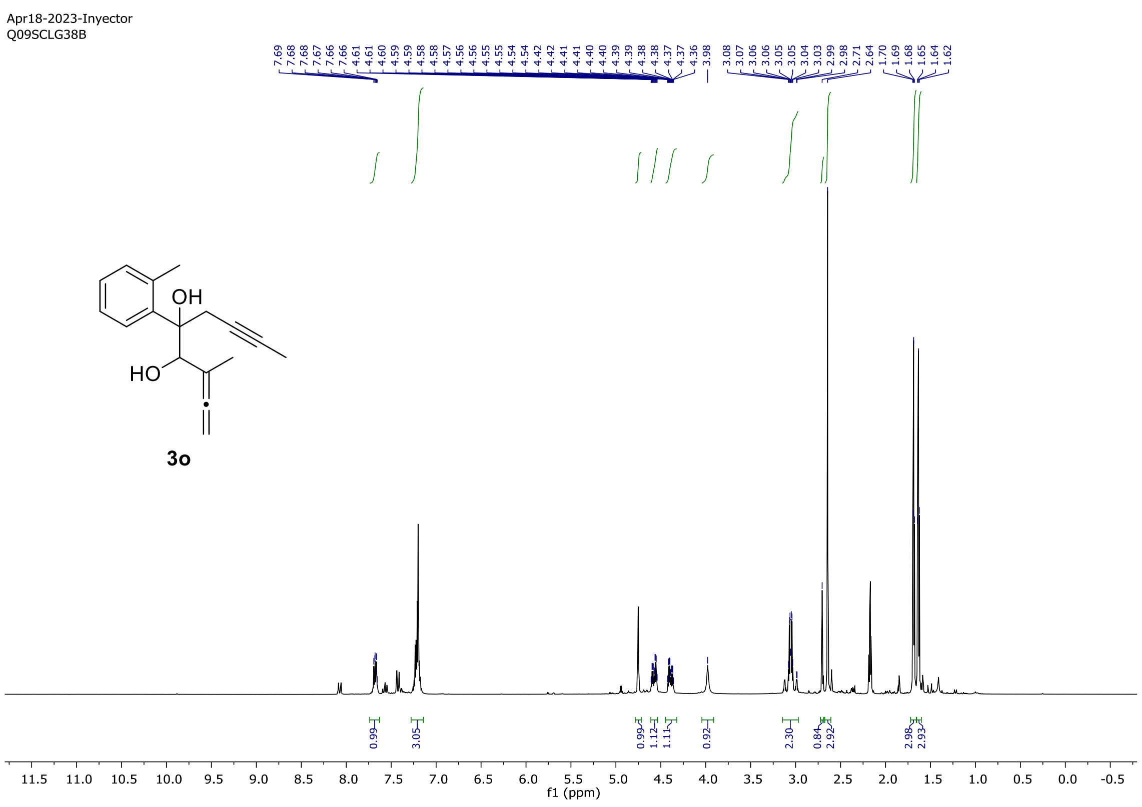

^{13}C NMR (75 MHz, acetone-d6, rt)

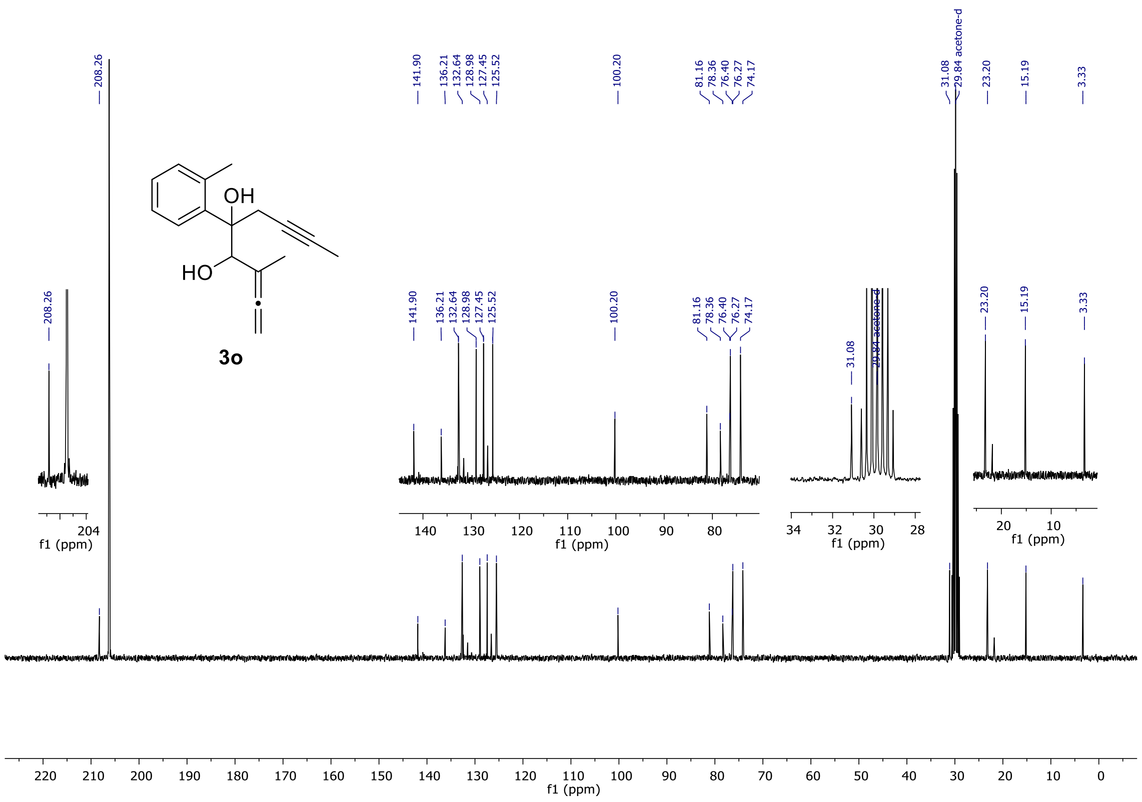

^{1}H NMR (300 MHz, acetone-d$_6$, rt)

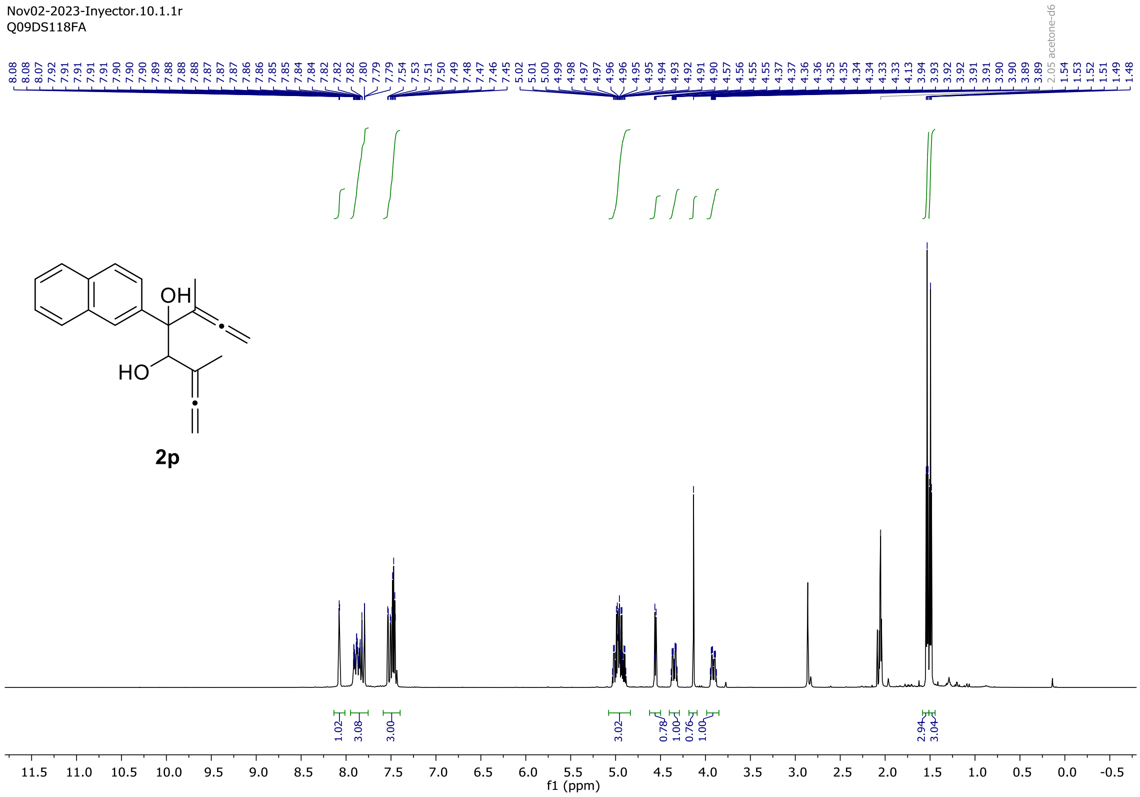

^{13}C NMR (75 MHz, acetone-d$_6$, rt)

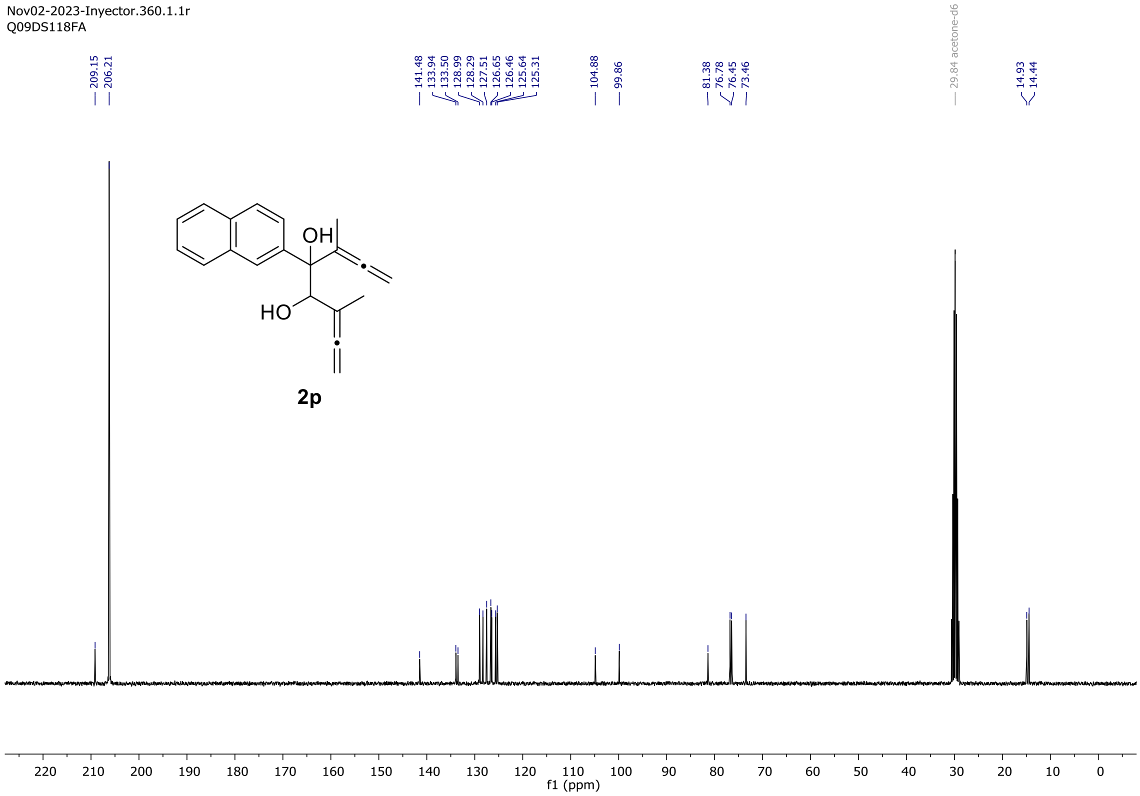

^{1}H NMR (300 MHz, acetone-d$_6$, rt)

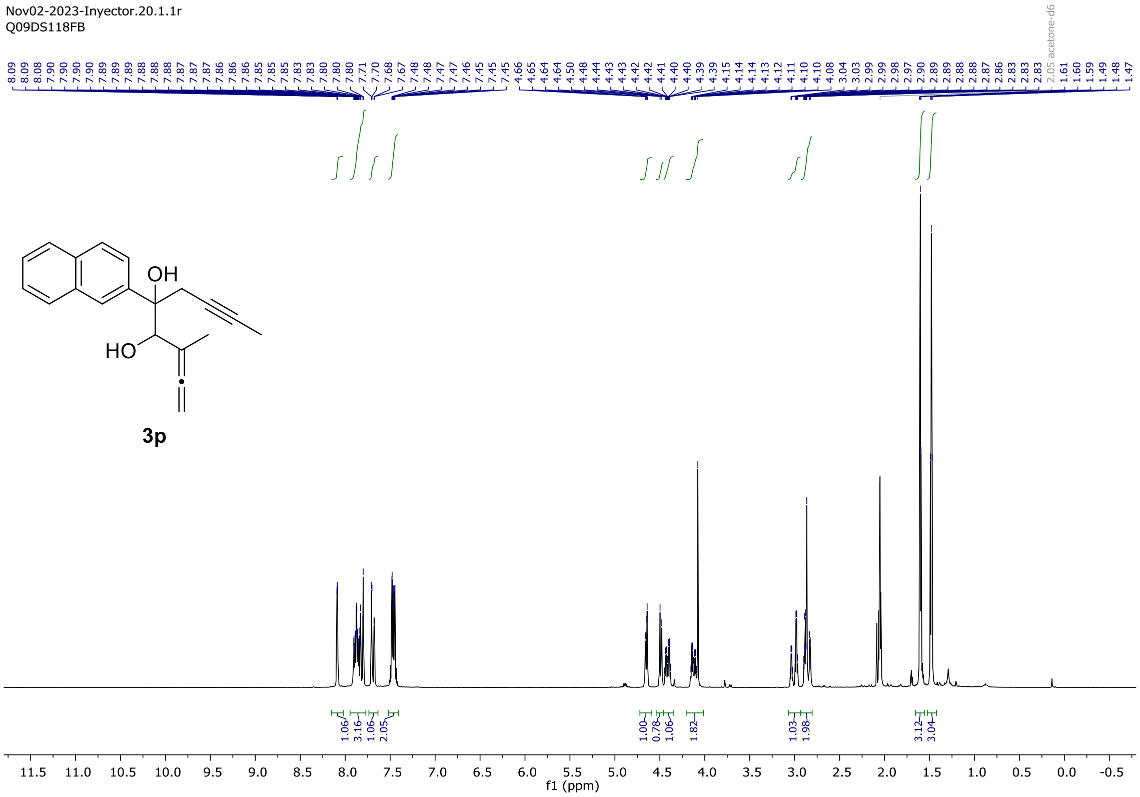

^{13}C NMR (75 MHz, acetone-d$_6$, rt)

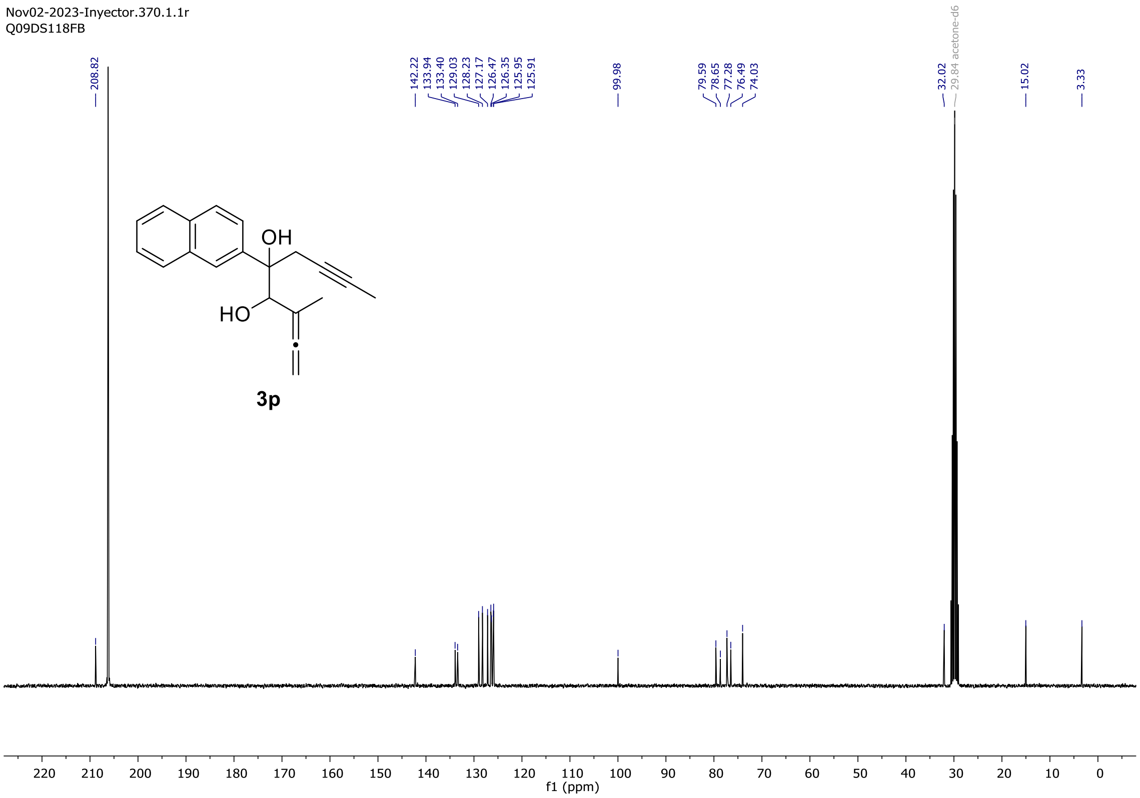

^{1}H NMR (300 MHz, $CDCl_3$, rt)

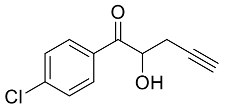

Ia

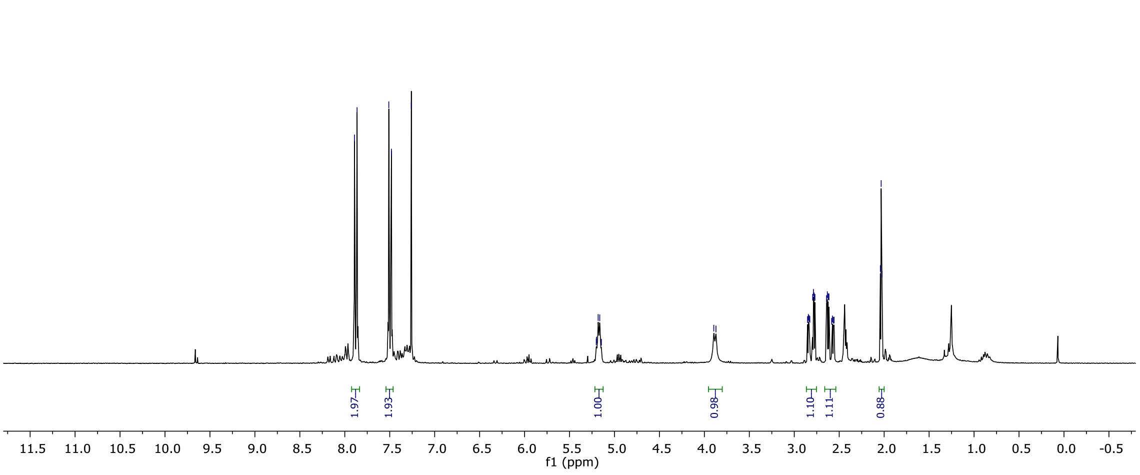

^{13}C NMR (75 MHz, $CDCl_3$, rt)

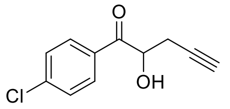

Ia

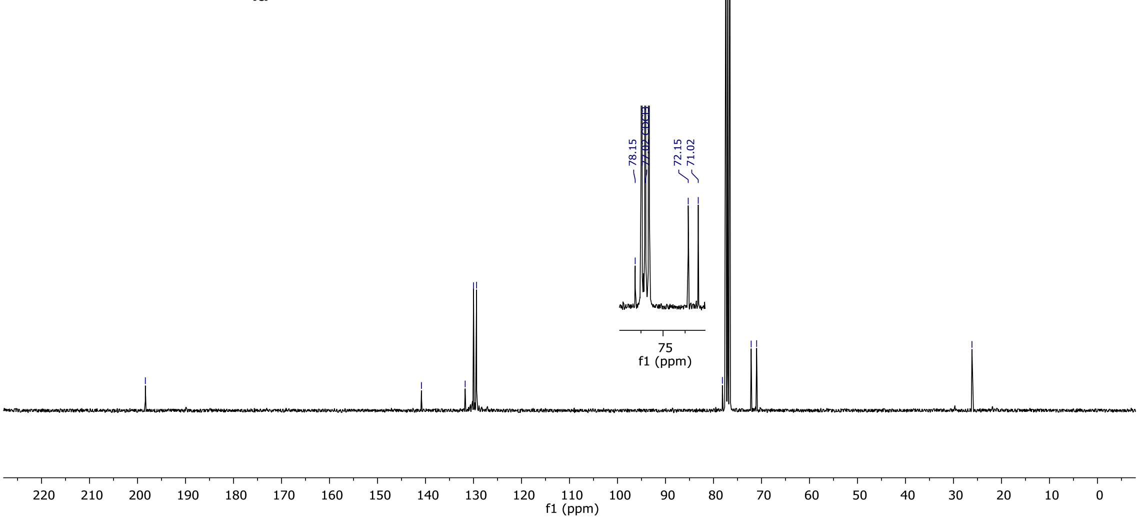

^{1}H NMR (300 MHz, acetone-d_6, rt)

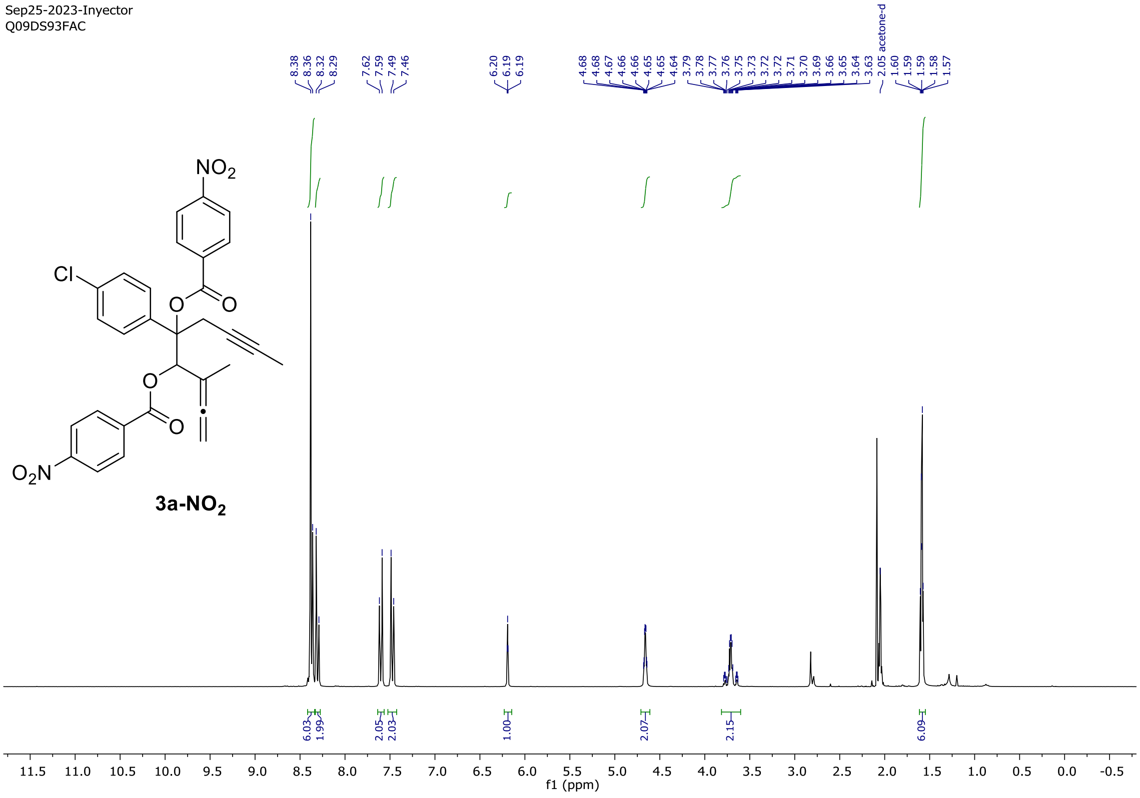

^{13}C NMR (75 MHz, acetone-d_6, rt)

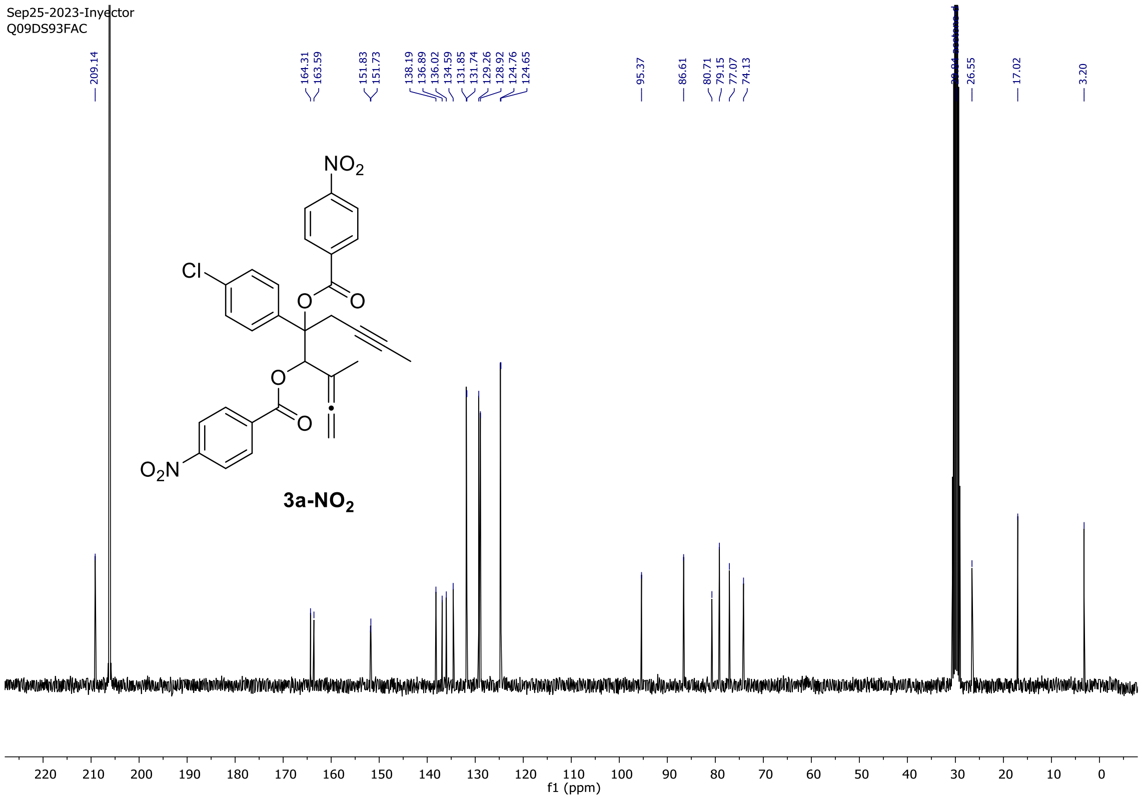

^{1}H NMR (300 MHz, $CDCl_3$, rt)

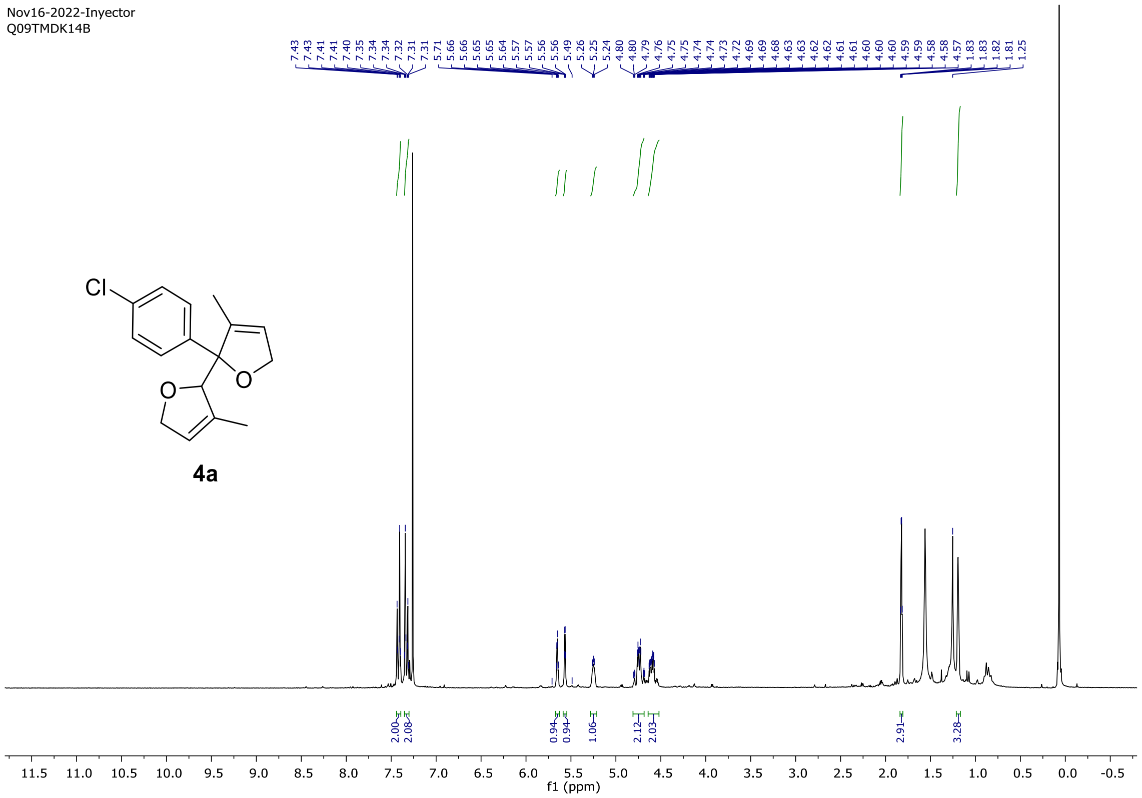

^{13}C NMR (75 MHz, $CDCl_3$, rt)

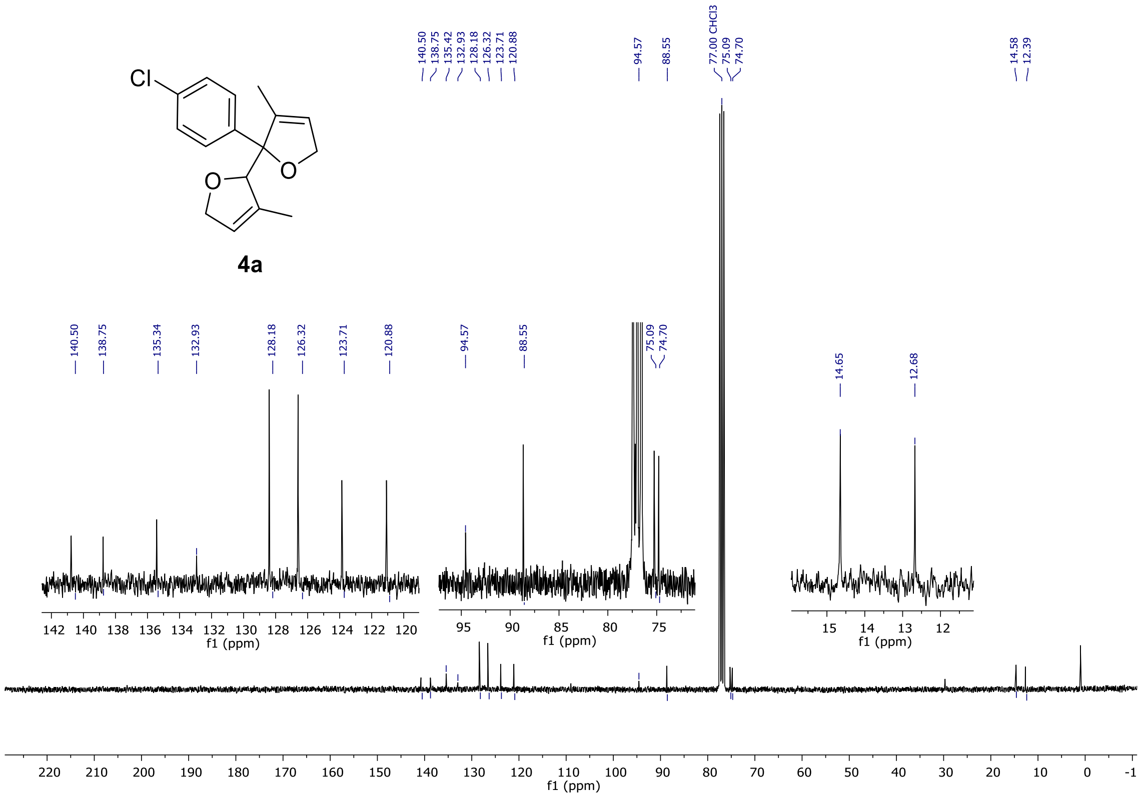

^{1}H NMR (300 MHz, $CDCl_3$, rt)

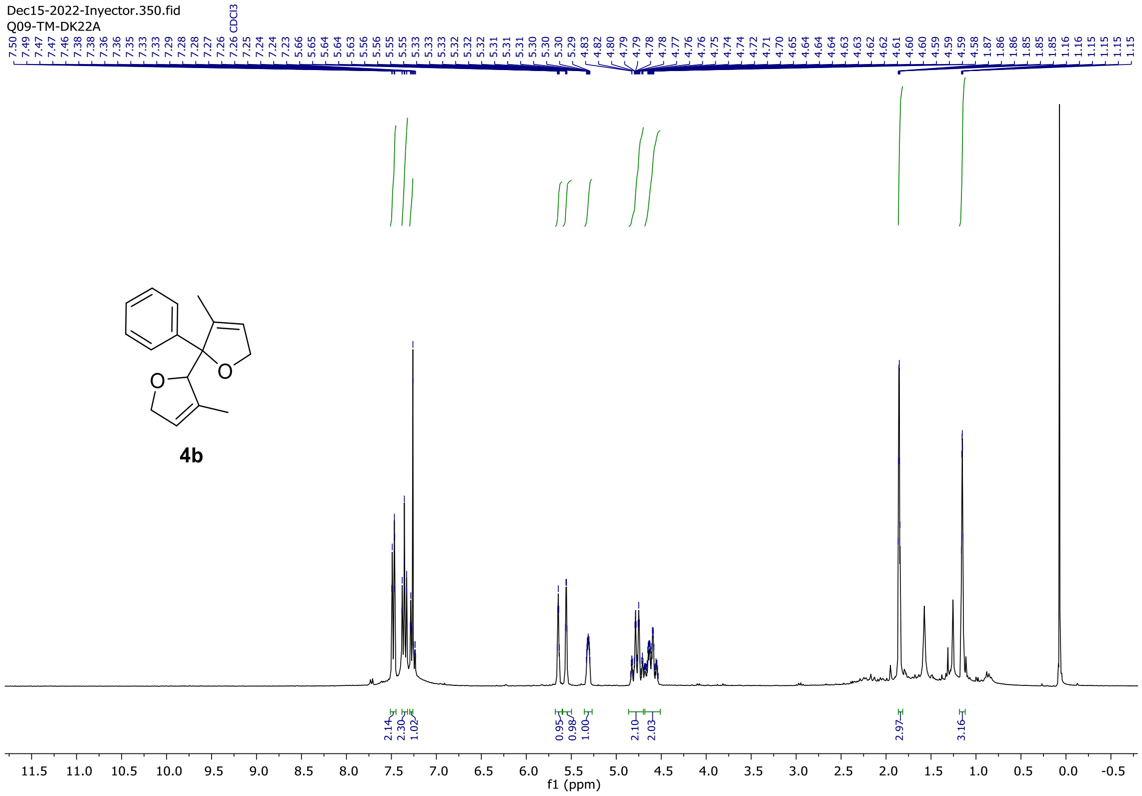

^{13}C NMR (75 MHz, $CDCl_3$, rt)

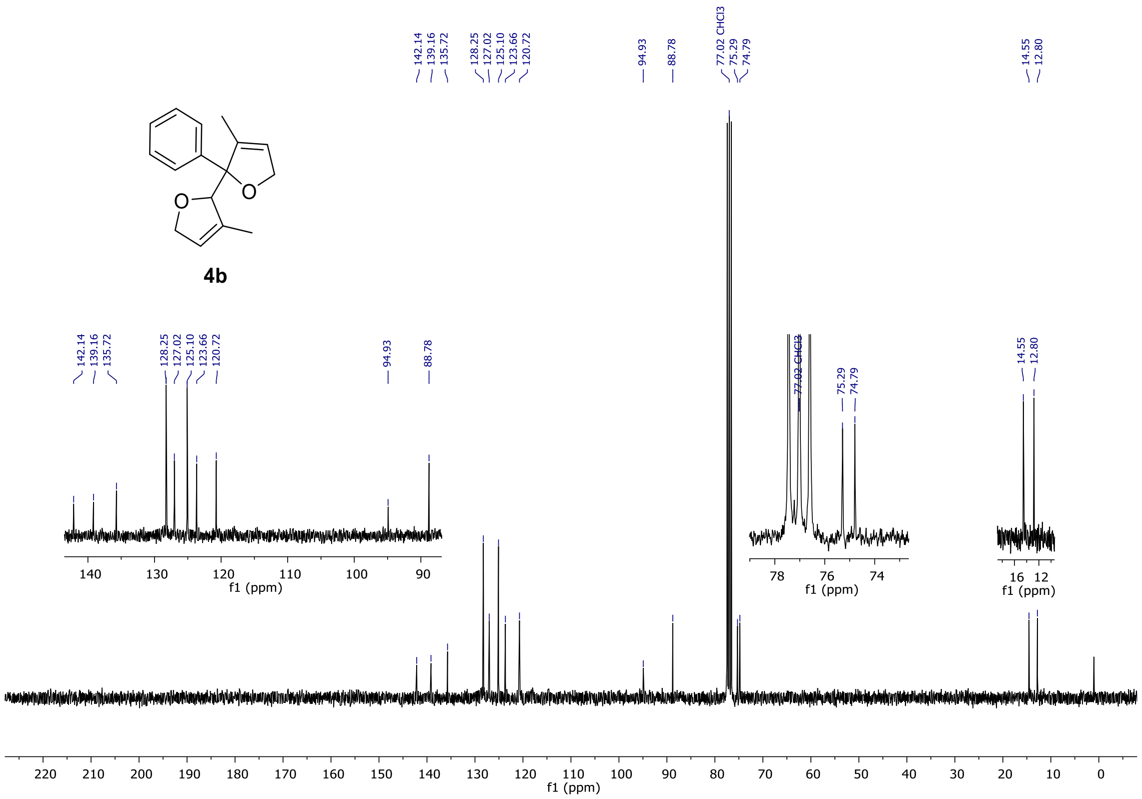

^{1}H NMR (300 MHz, $CDCl_3$, rt)

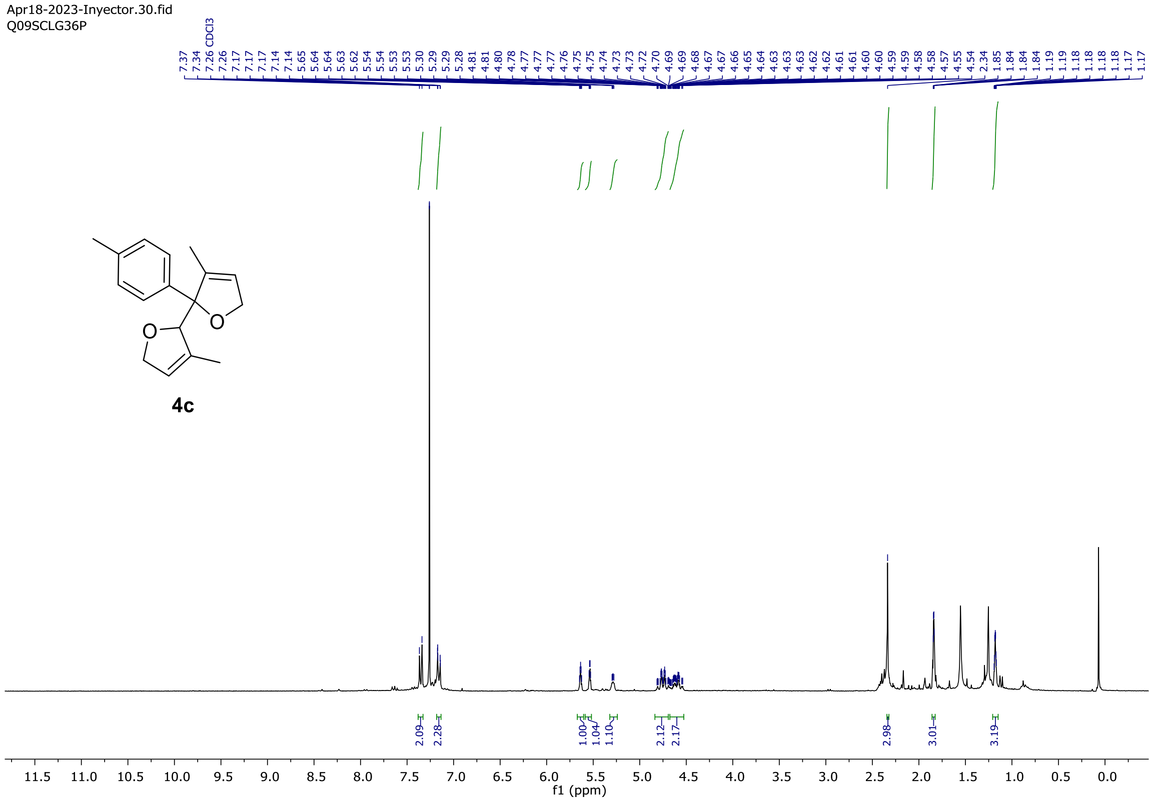

^{13}C NMR (175 MHz, $CDCl_3$, rt)

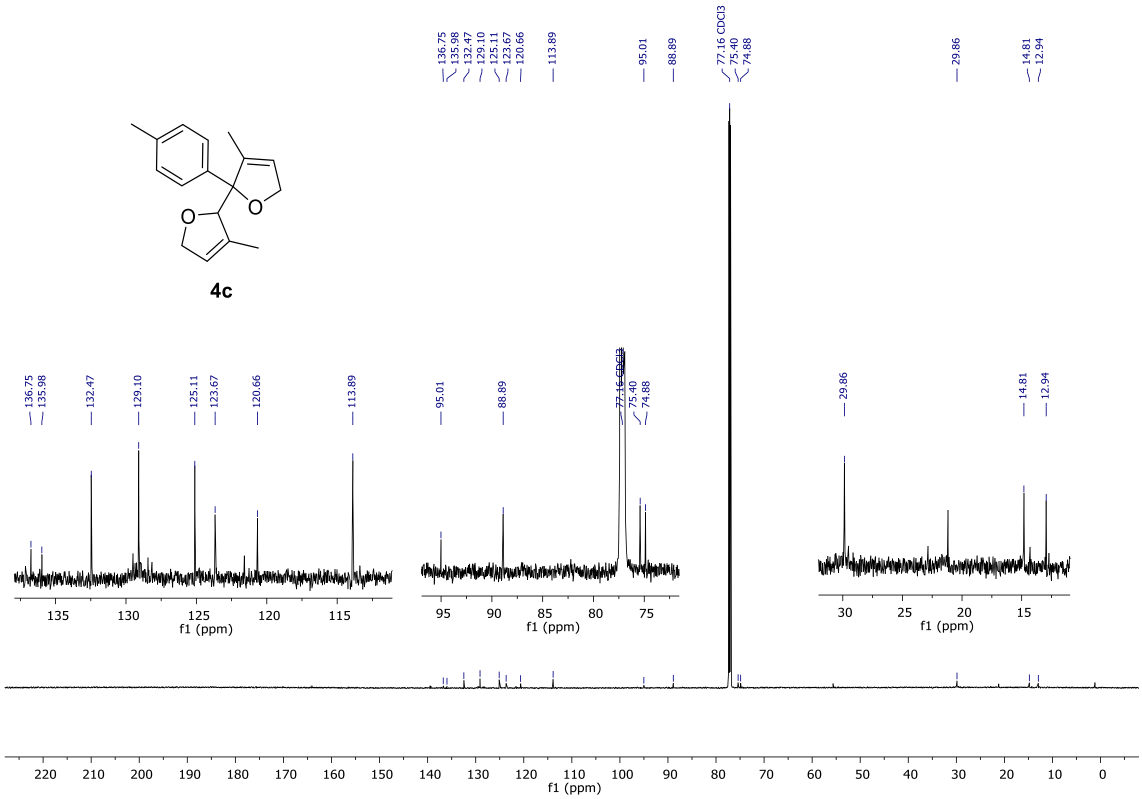

^{1}H NMR (300 MHz, $CDCl_3$, rt)

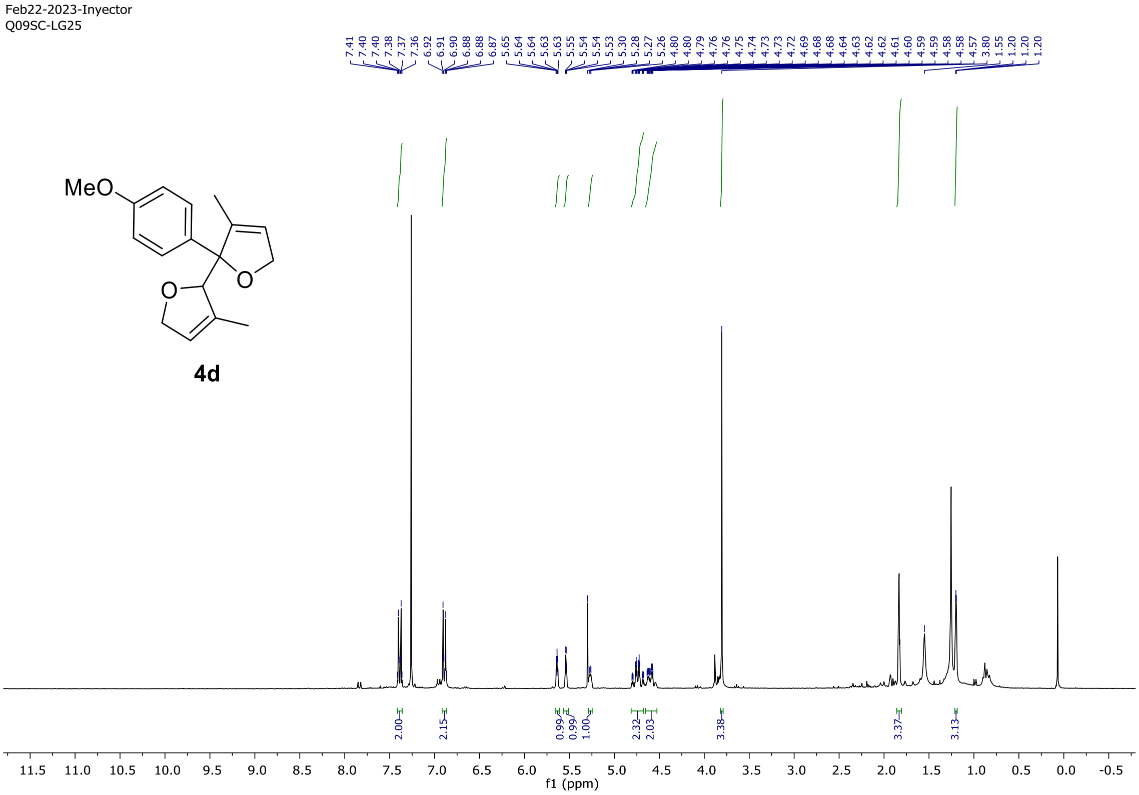

^{13}C NMR (175 MHz, $CDCl_3$, rt)

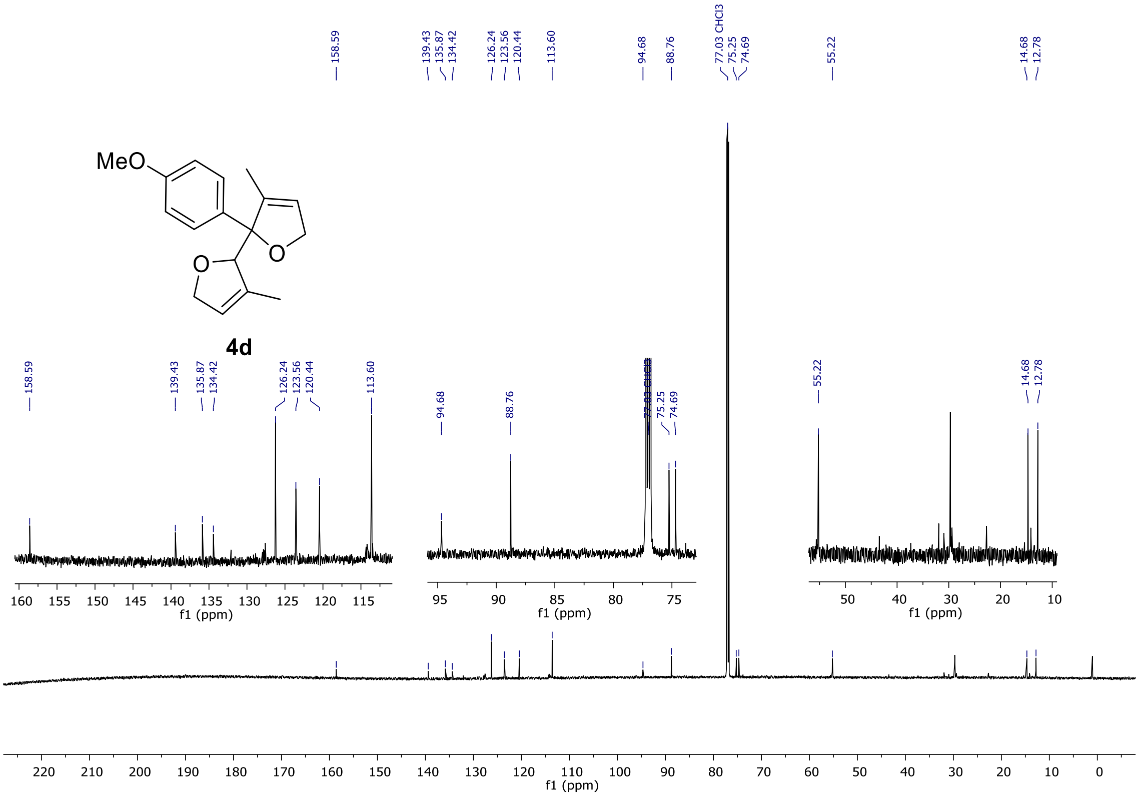

^{1}H NMR (300 MHz, acetone-d$_6$, rt)

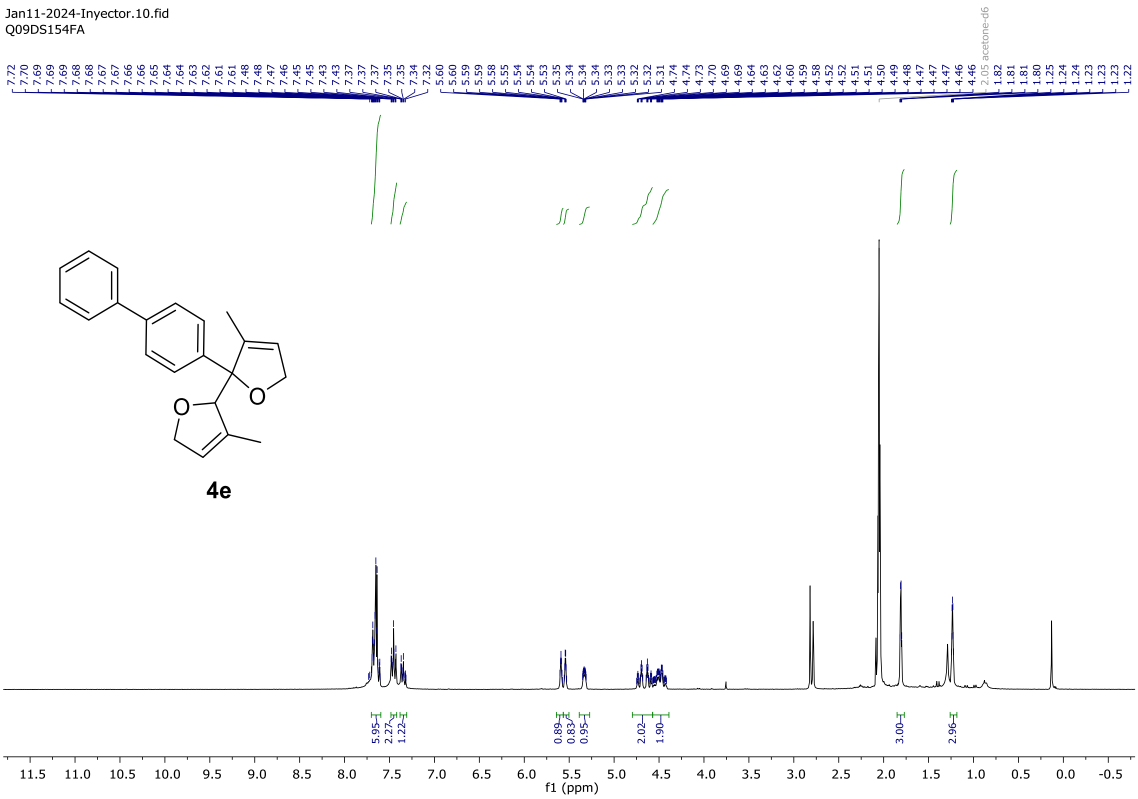

^{13}C NMR (75 MHz, acetone-d$_6$, rt)

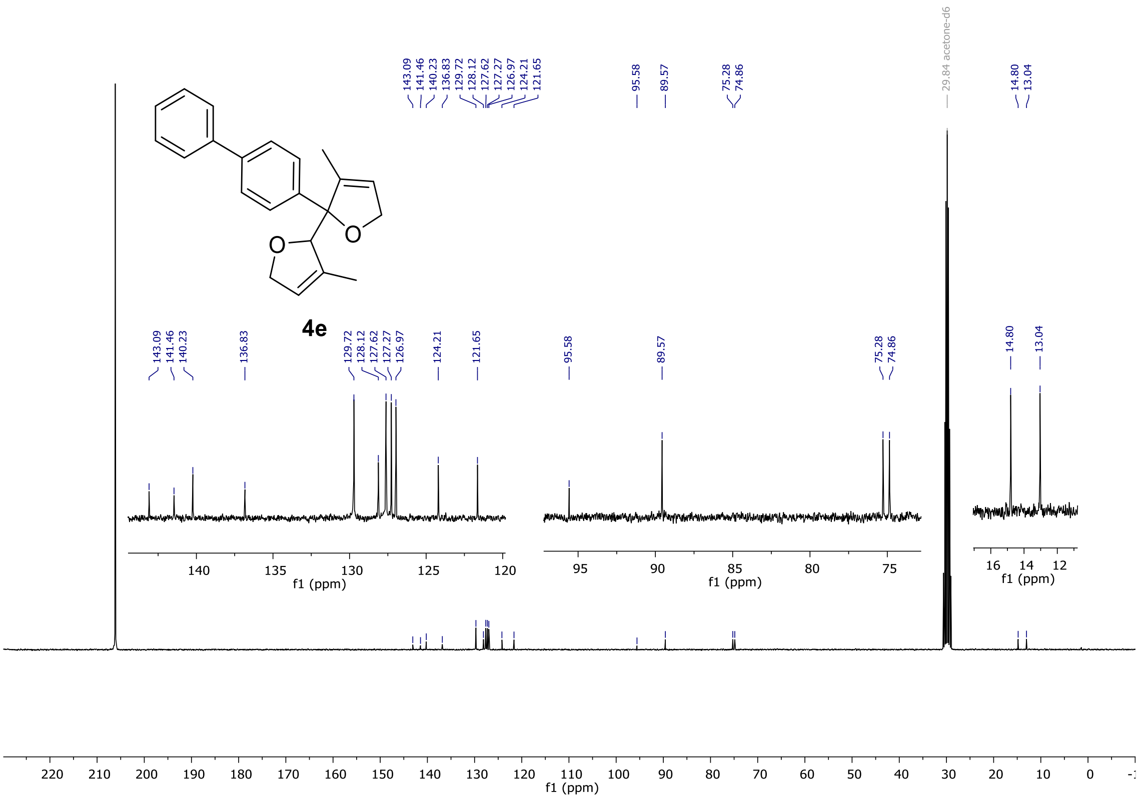

^1H NMR (300 MHz, acetone-d$_6$, rt)

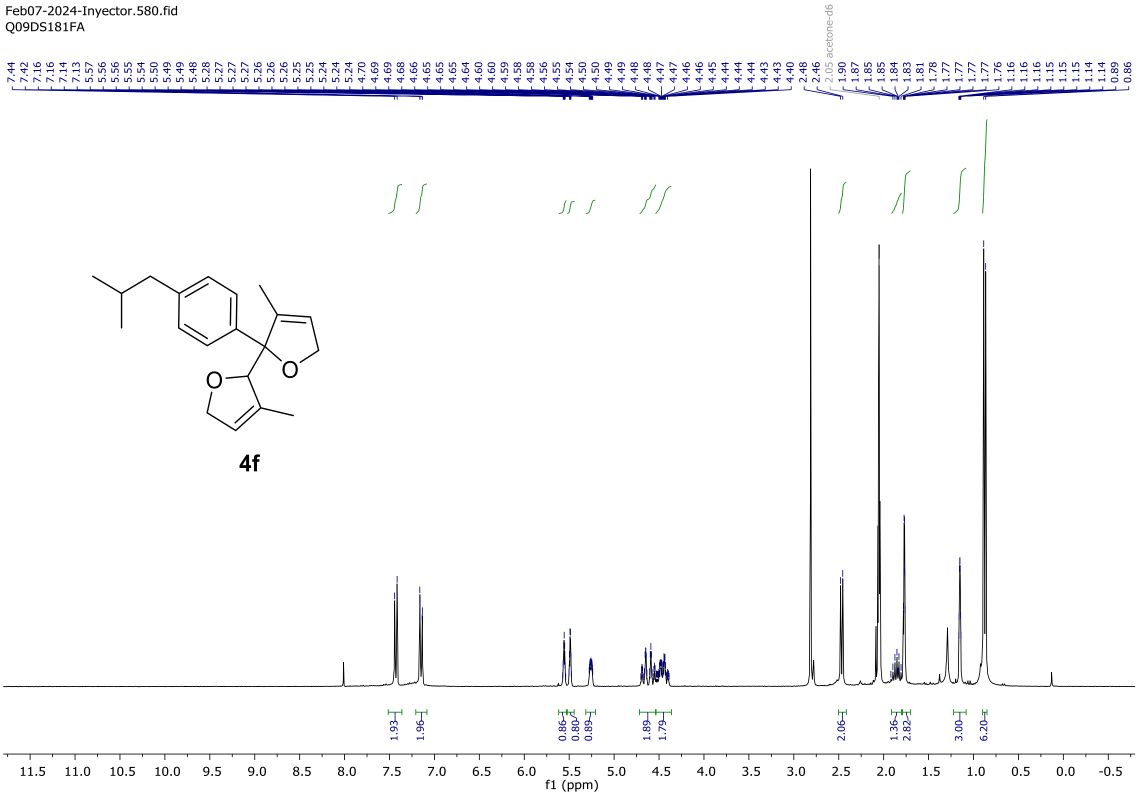

^{13}C NMR (75 MHz, acetone-d$_6$, rt)

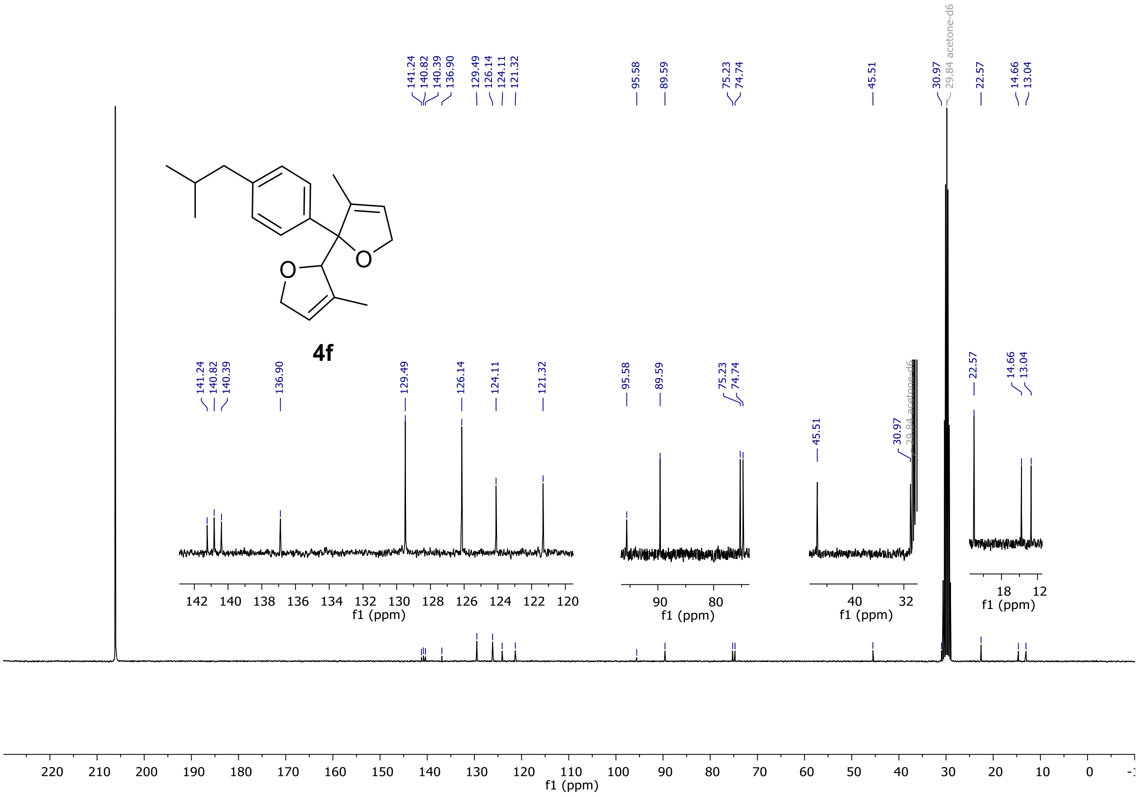

^{1}H NMR (300 MHz, acetone-d_6, rt)

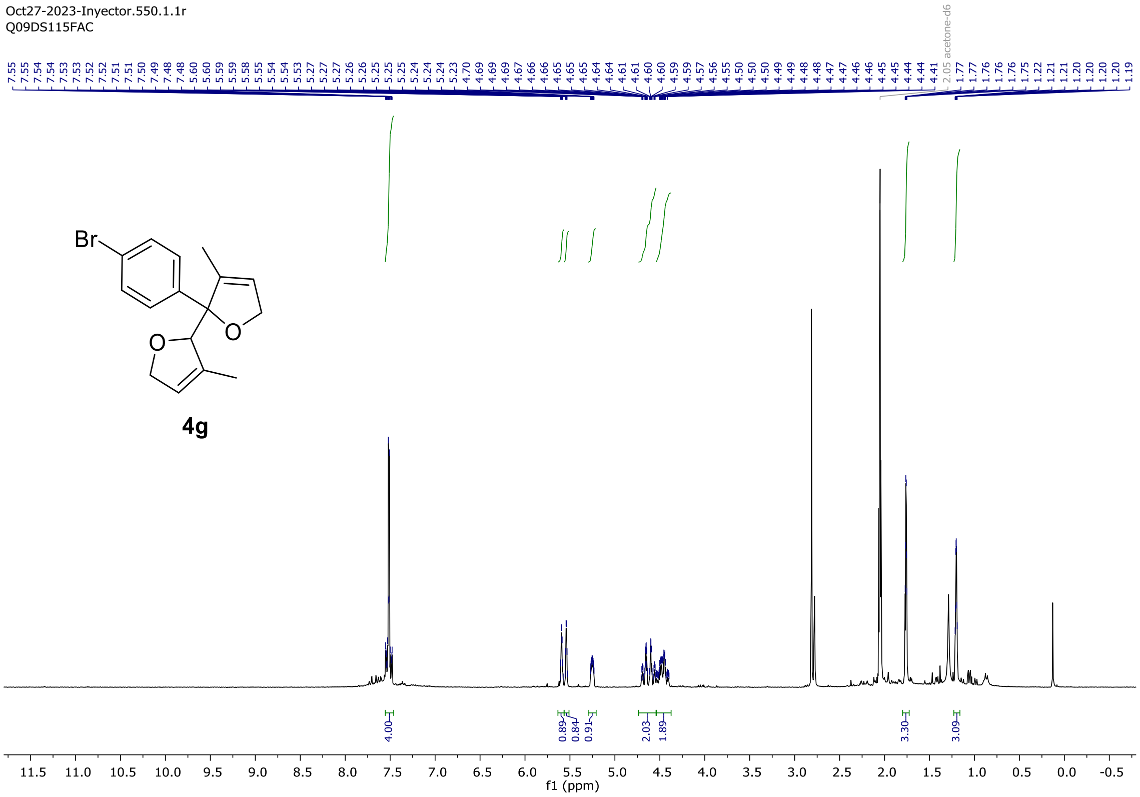

^{13}C NMR (75 MHz, acetone-d_6, rt)

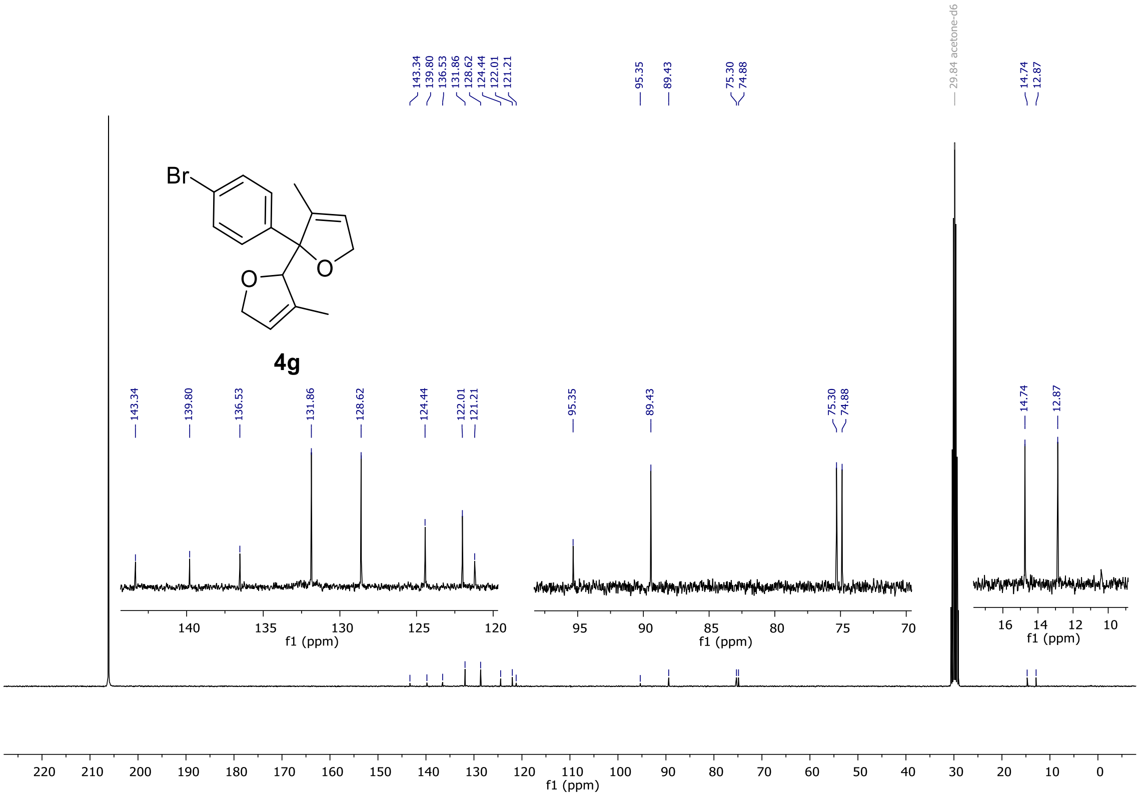

^{1}H NMR (300 MHz, acetone-d$_6$, rt)

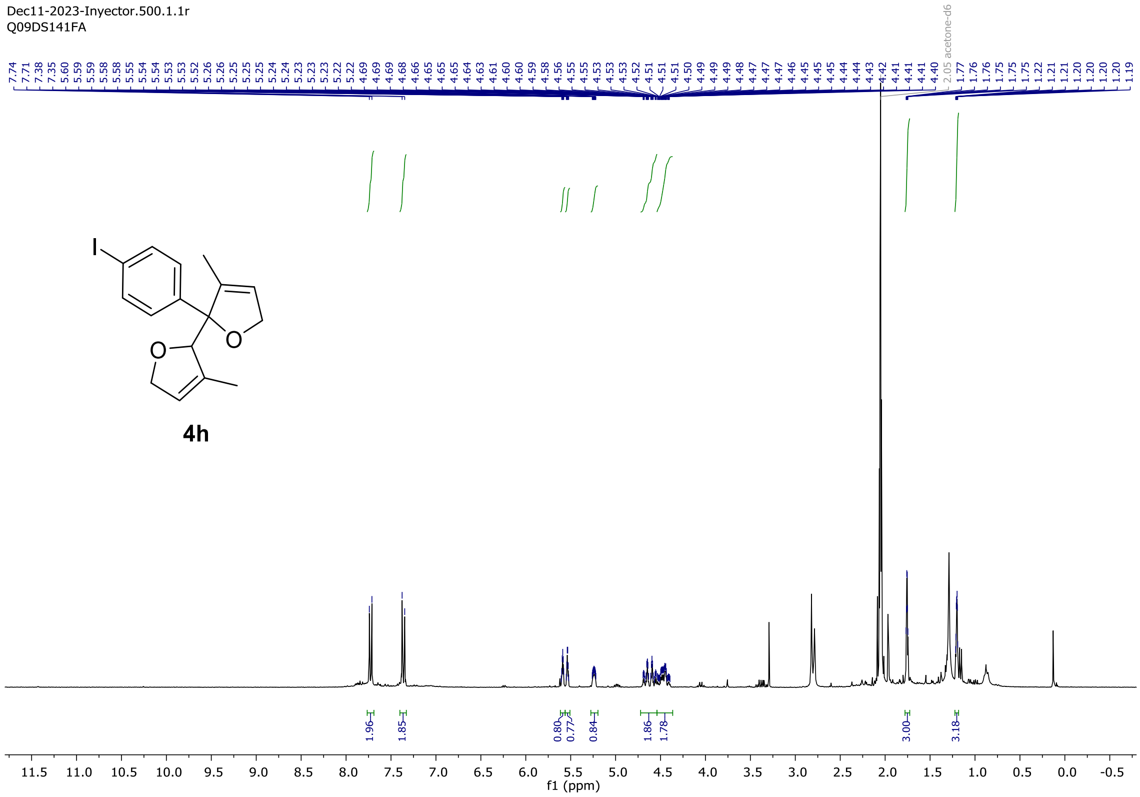

^{1}H NMR (300 MHz, acetone-d$_6$, rt)

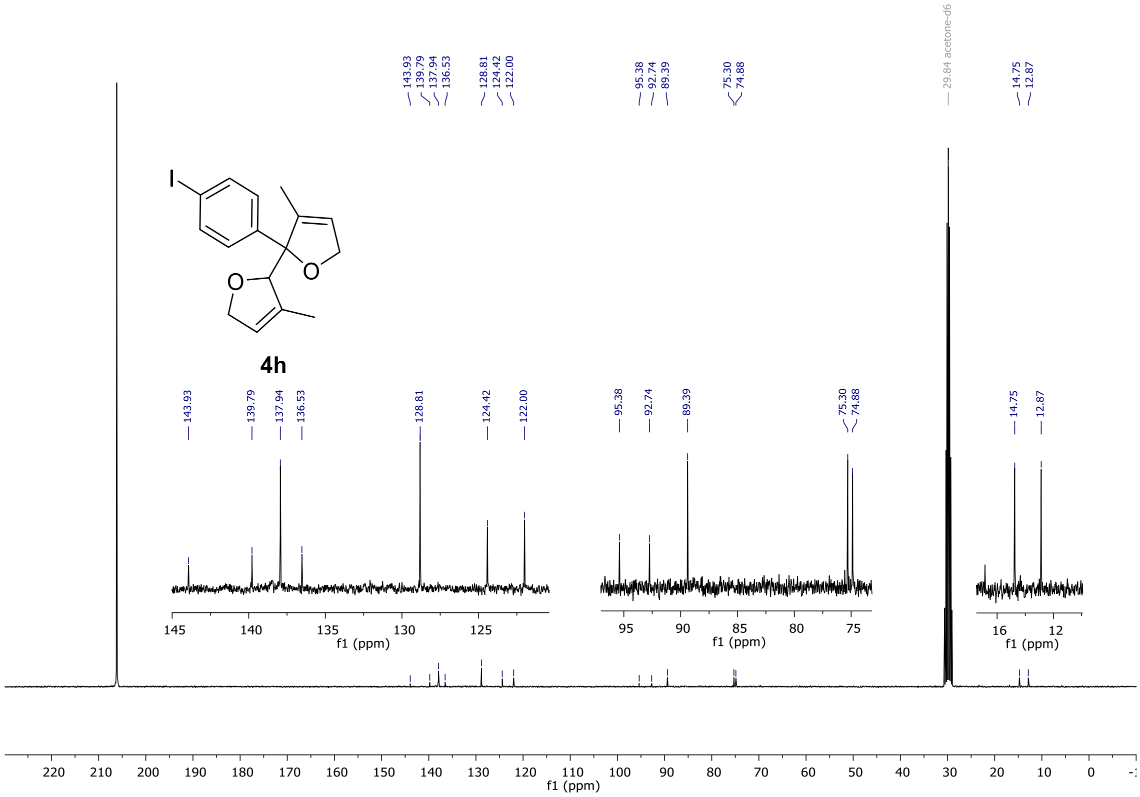

^{1}H NMR (300 MHz, CD_3CN, rt)

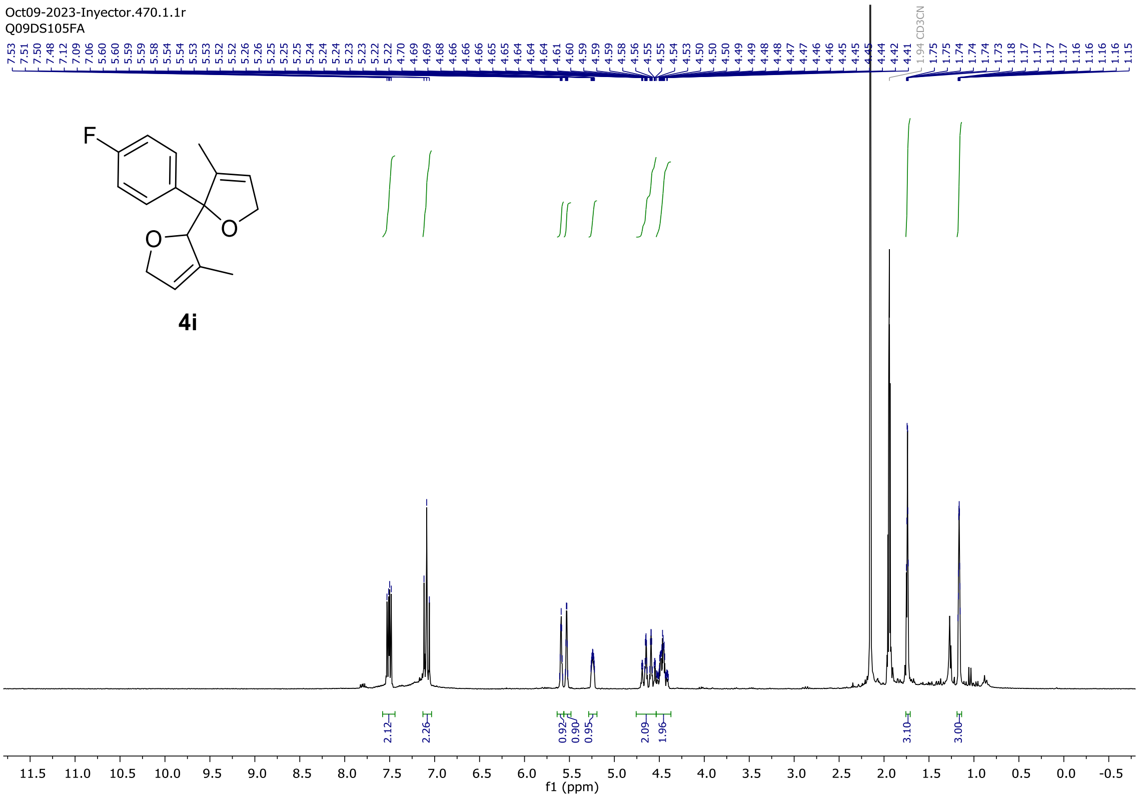

^{13}C NMR (75 MHz, CD_3CN, rt)

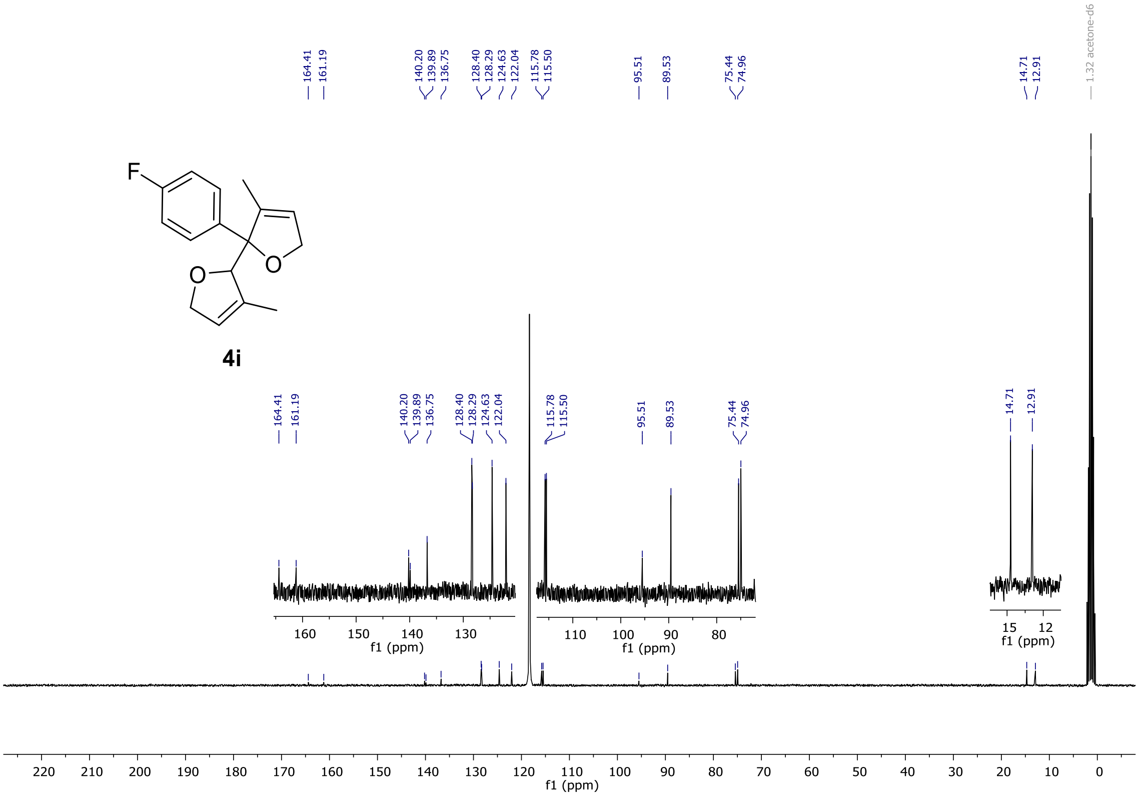

^{1}H NMR (300 MHz, $CDCl_3$, rt)

May03-2023-Inyector
Q09SCLG42P

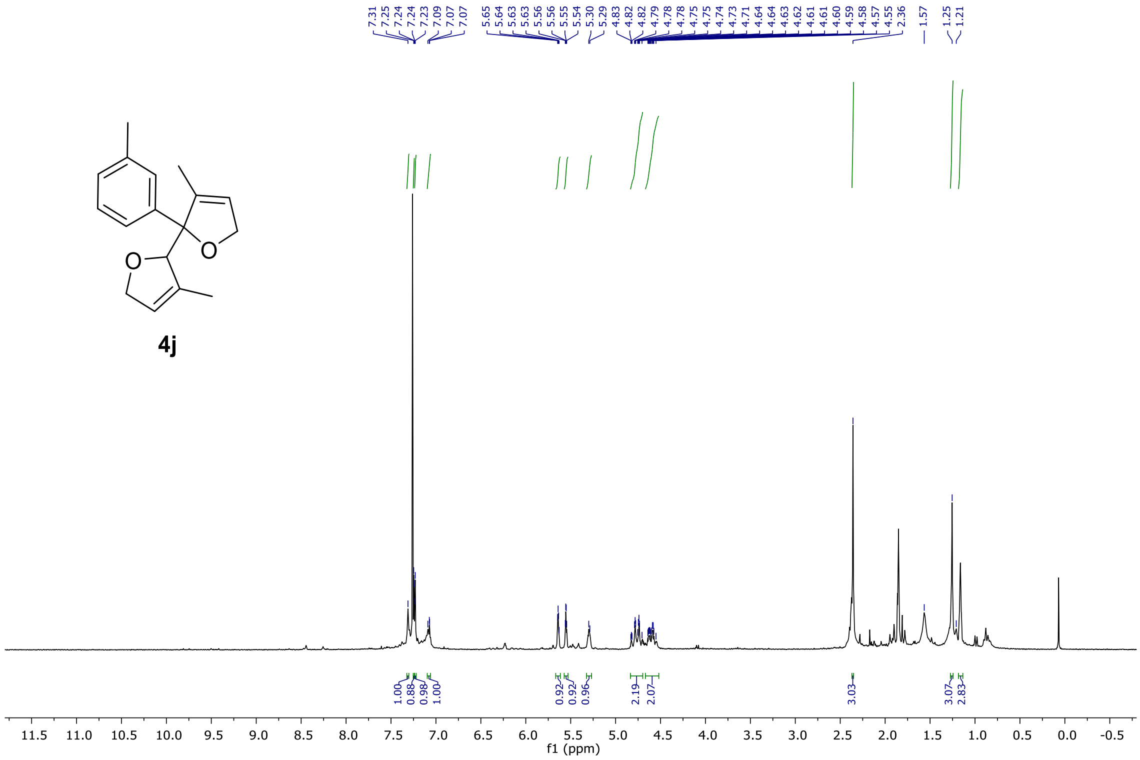

^{13}C NMR (175 MHz, $CDCl_3$, rt)

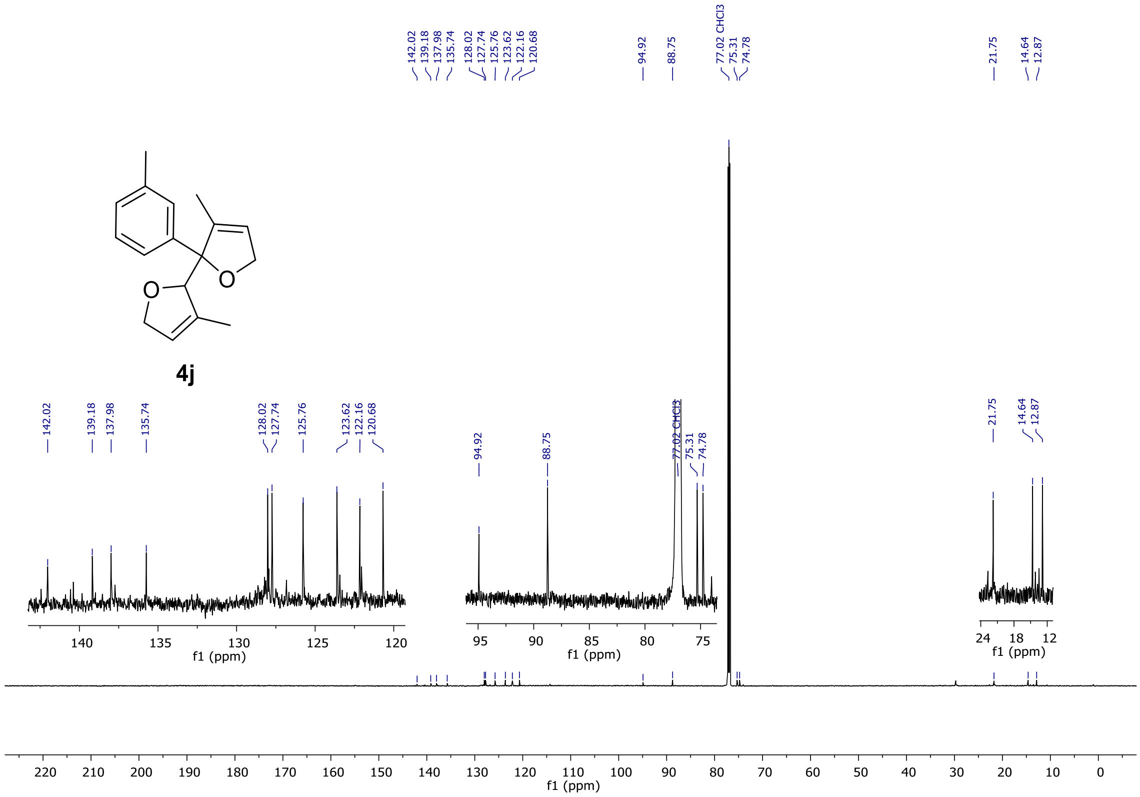

^{1}H NMR (300 MHz, acetone-d$_6$, rt)

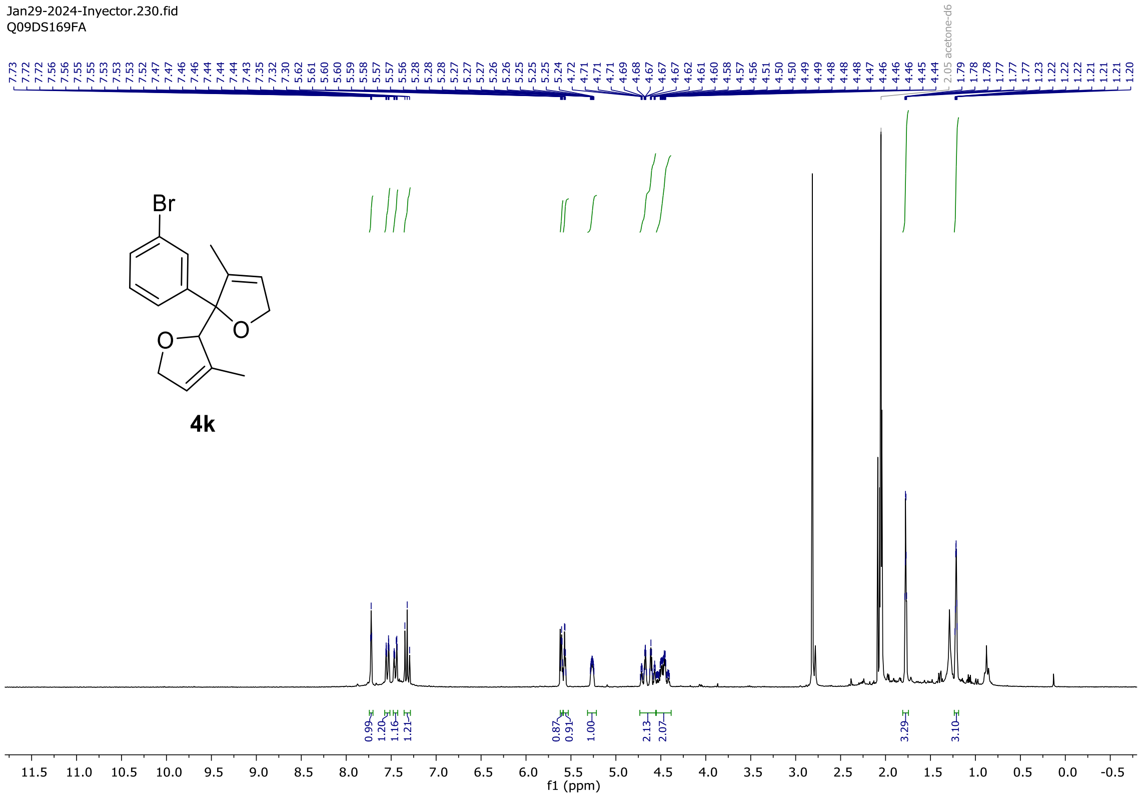

^{13}C NMR (75 MHz, acetone-d$_6$, rt)

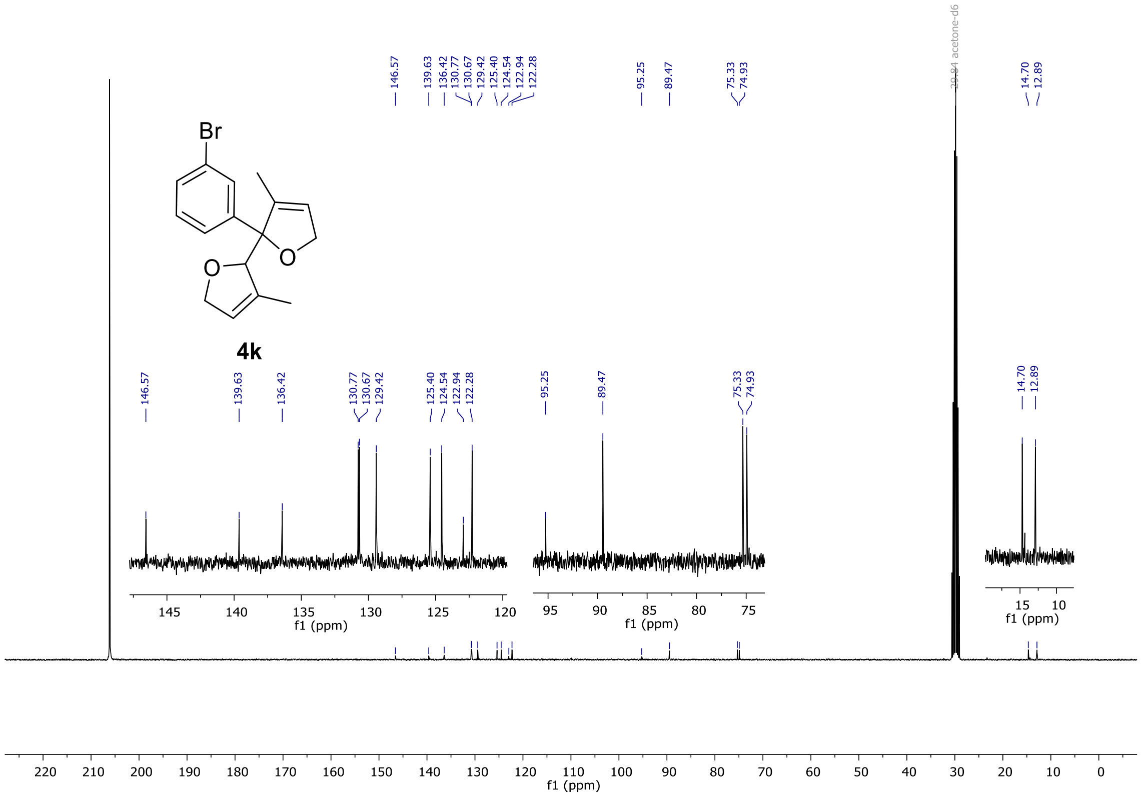

^{1}H NMR (300 MHz, acetone-d$_6$, rt)

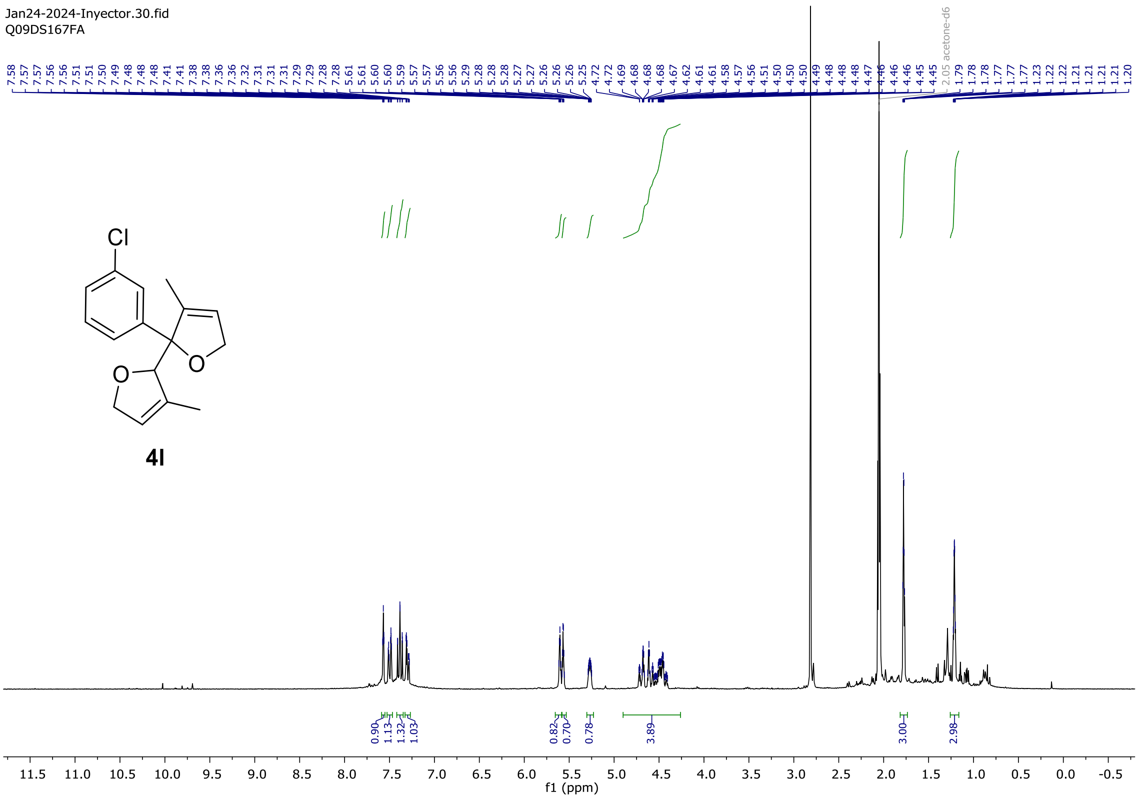

^{13}C NMR (75 MHz, acetone-d$_6$, rt)

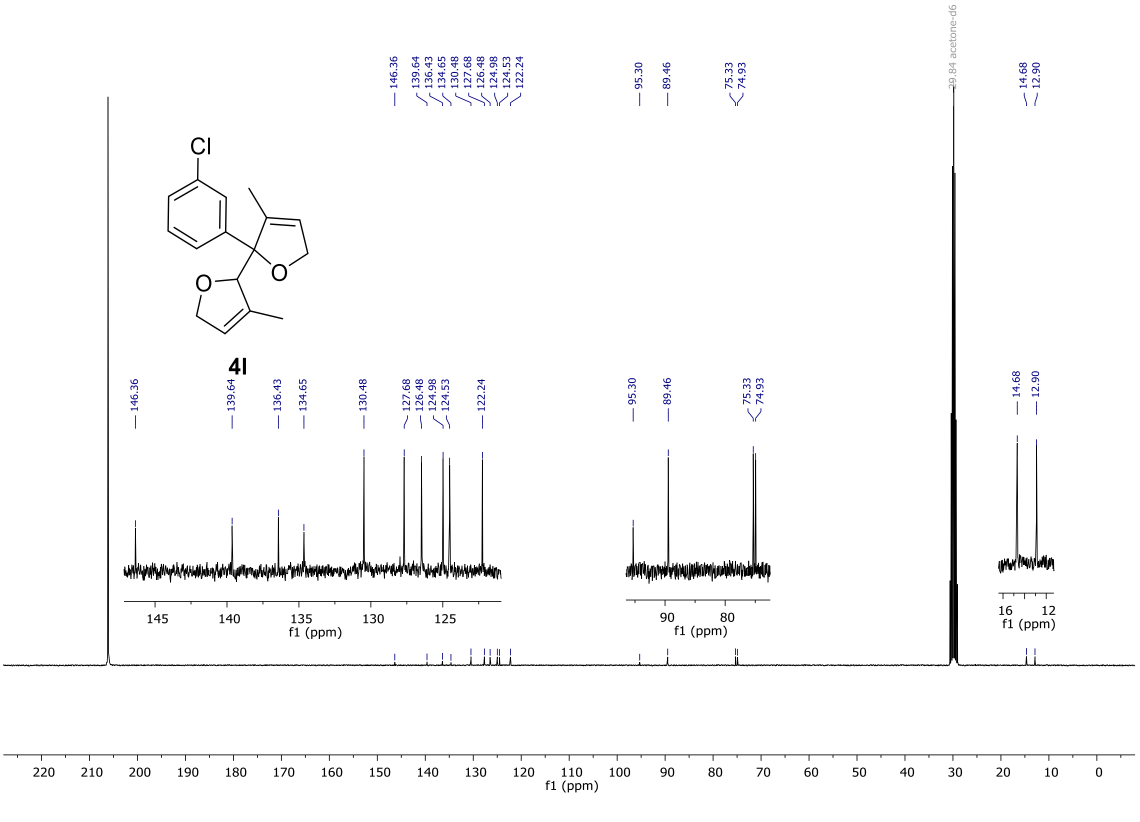

^{1}H NMR (300 MHz, acetone-d6, rt)

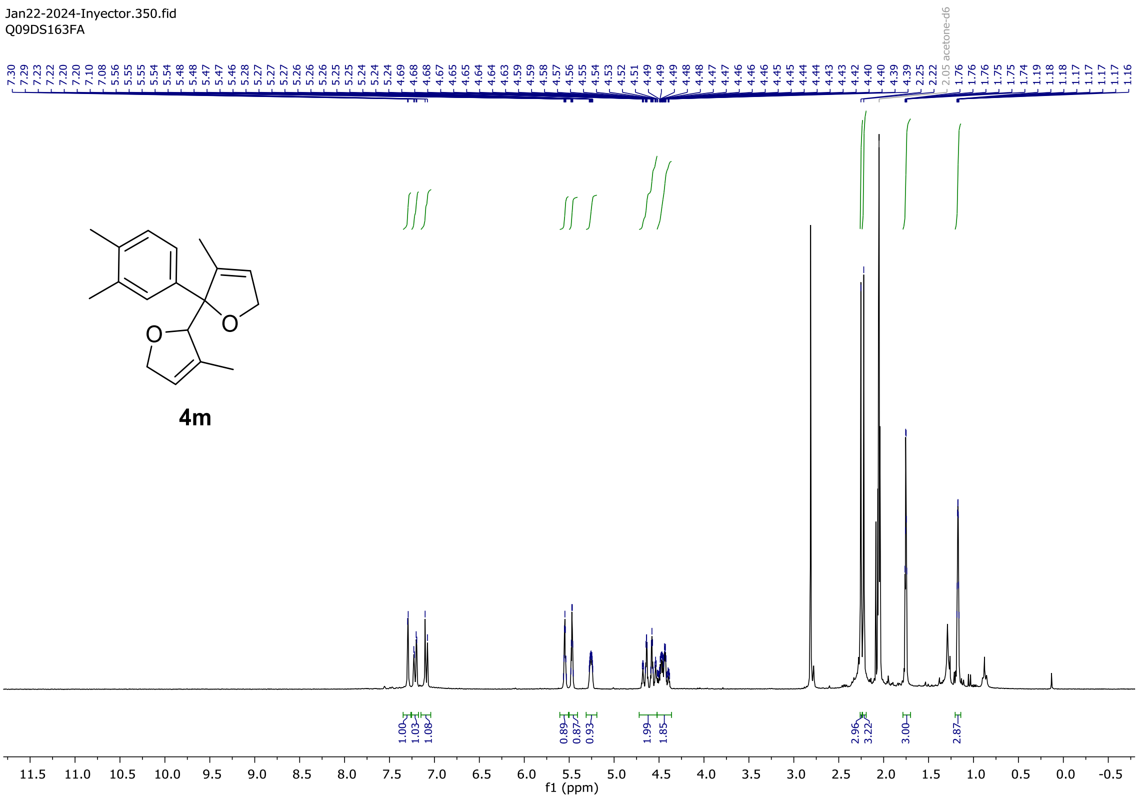

^{13}C NMR (75 MHz, acetone-d6, rt)

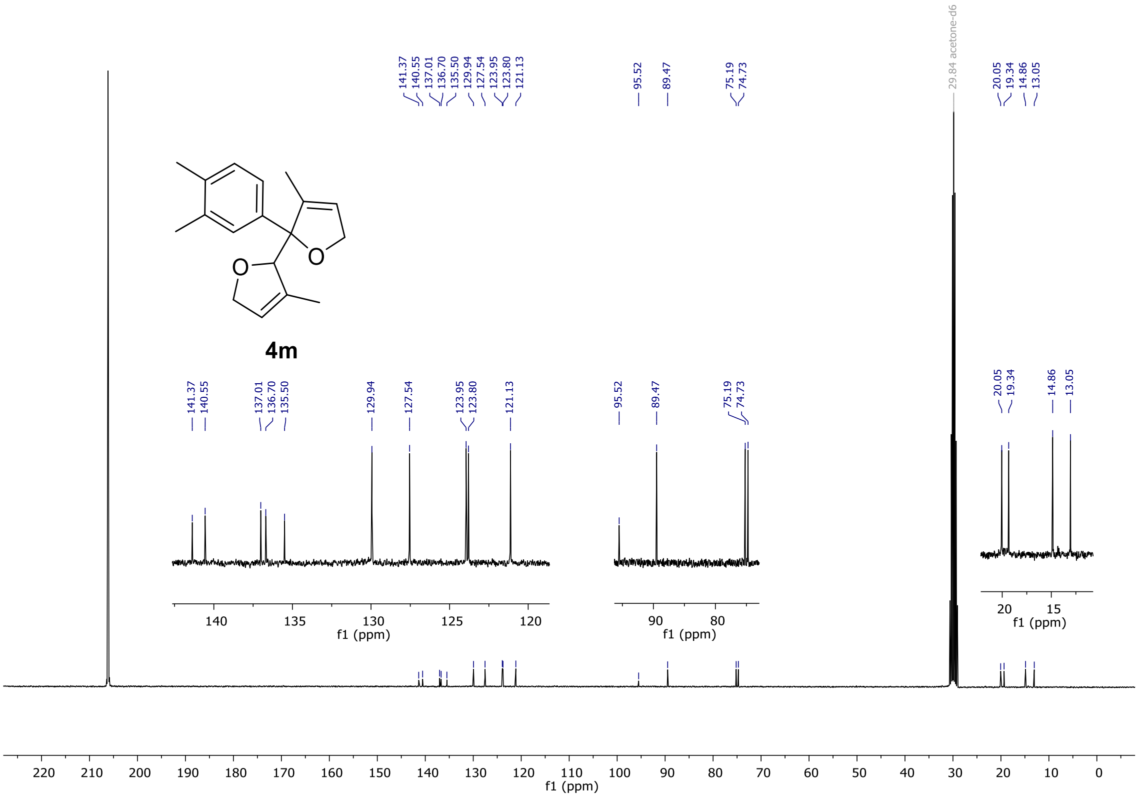

^{1}H NMR (300 MHz, acetone-d_6, rt)

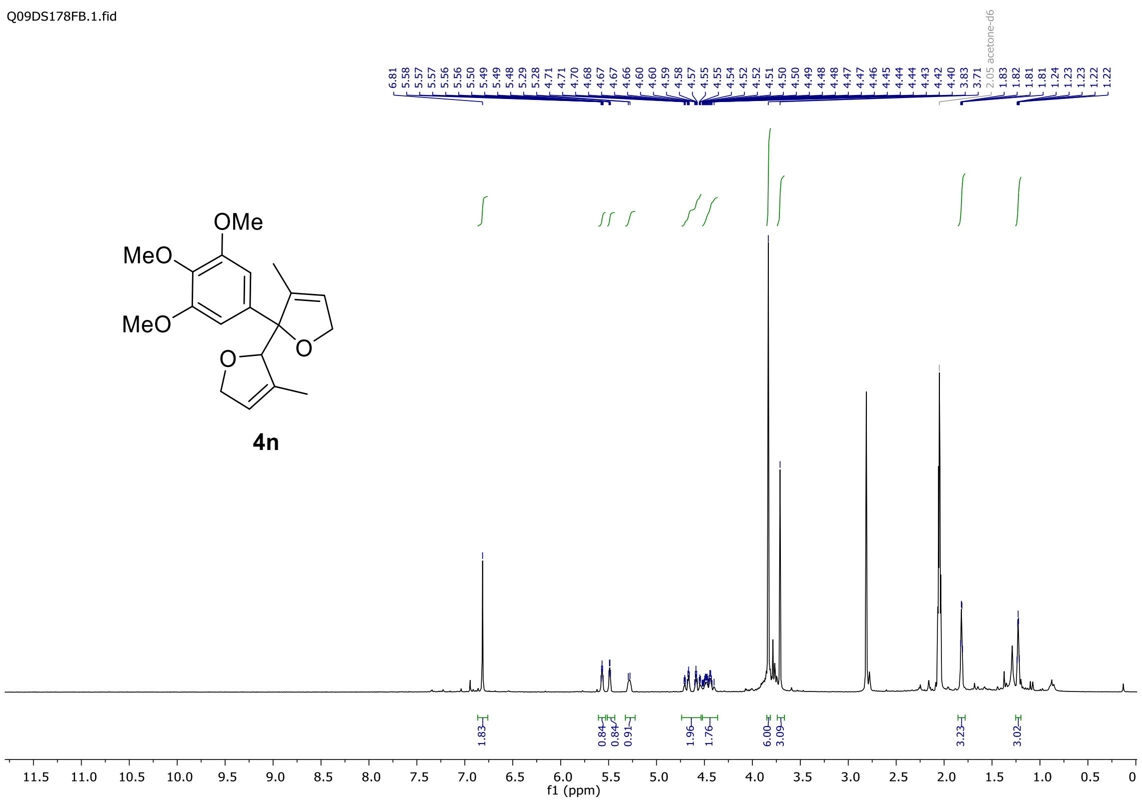

^{13}C NMR (75 MHz, acetone-d_6, rt)

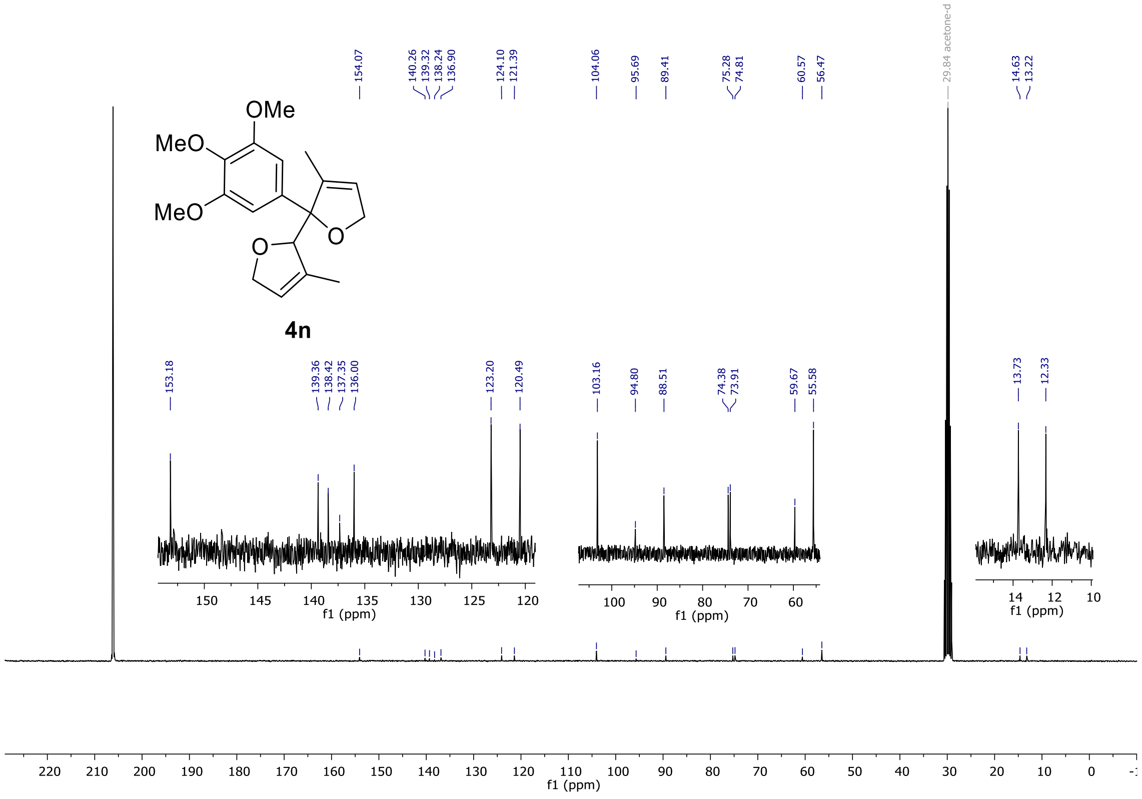

^{1}H NMR (300 MHz, acetone-d$_6$, rt)

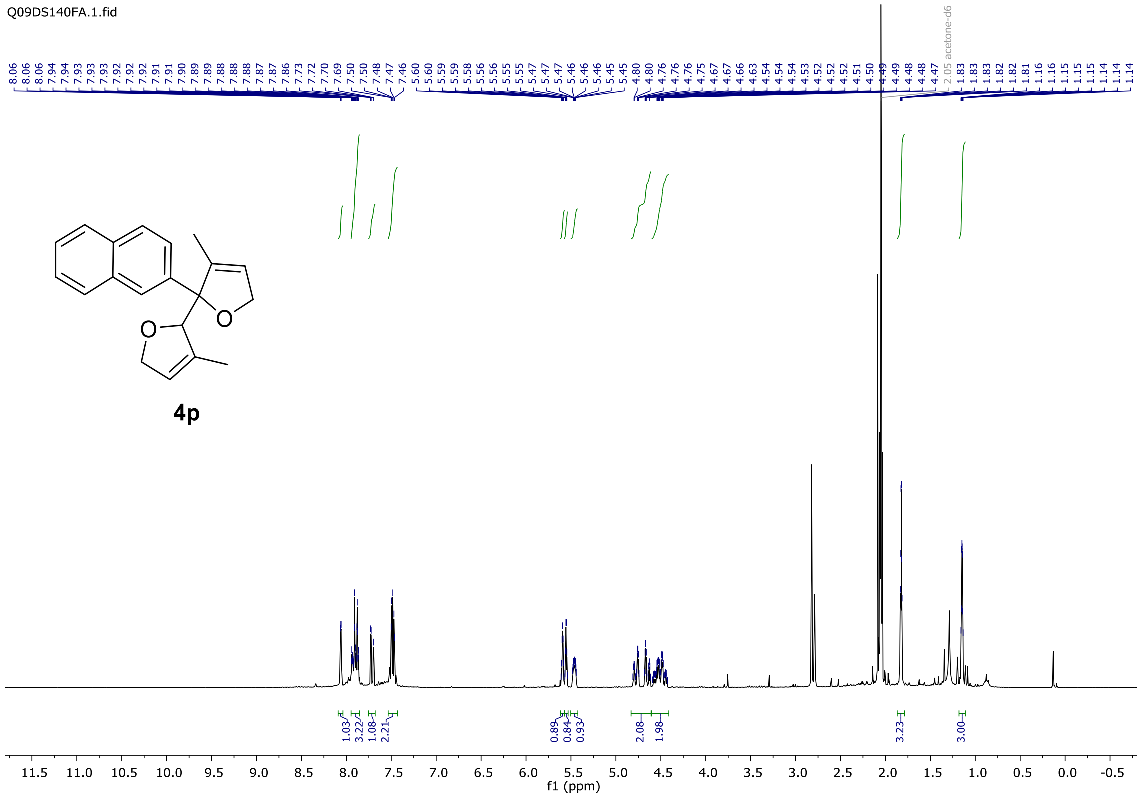

^{13}C NMR (75 MHz, acetone-d$_6$, rt)

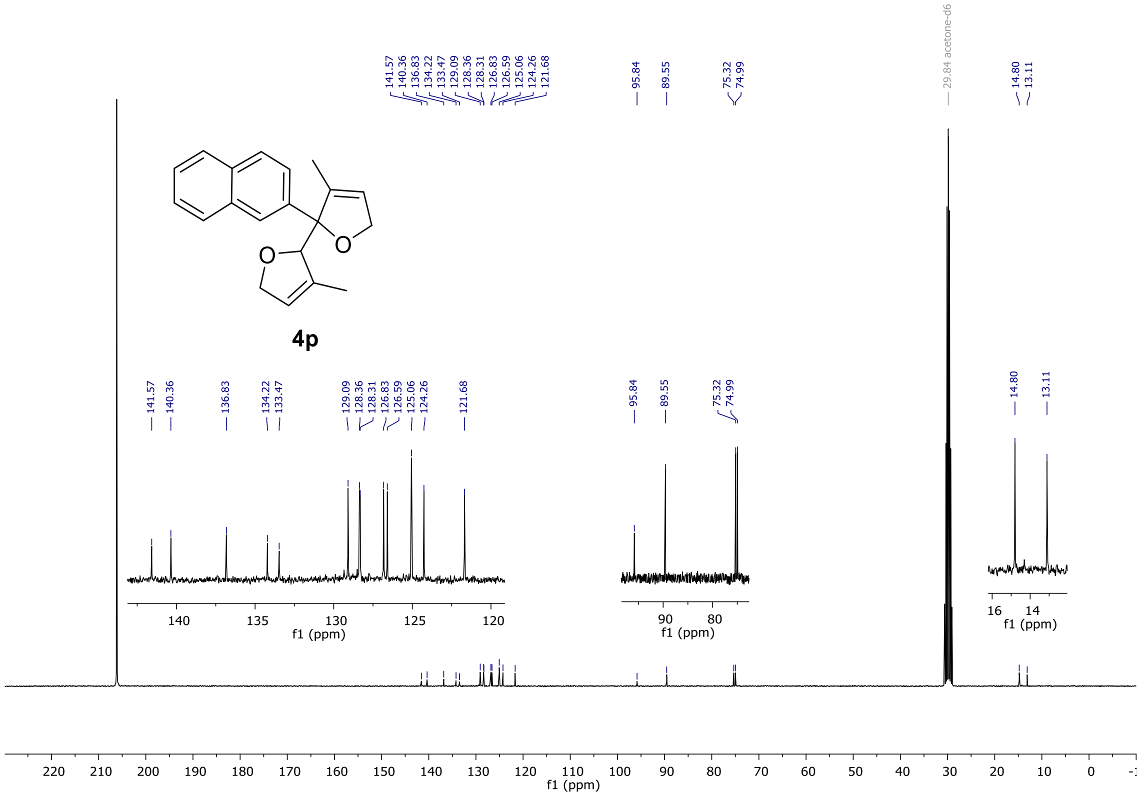

^{1}H NMR (300 MHz, acetone-d$_6$, rt)

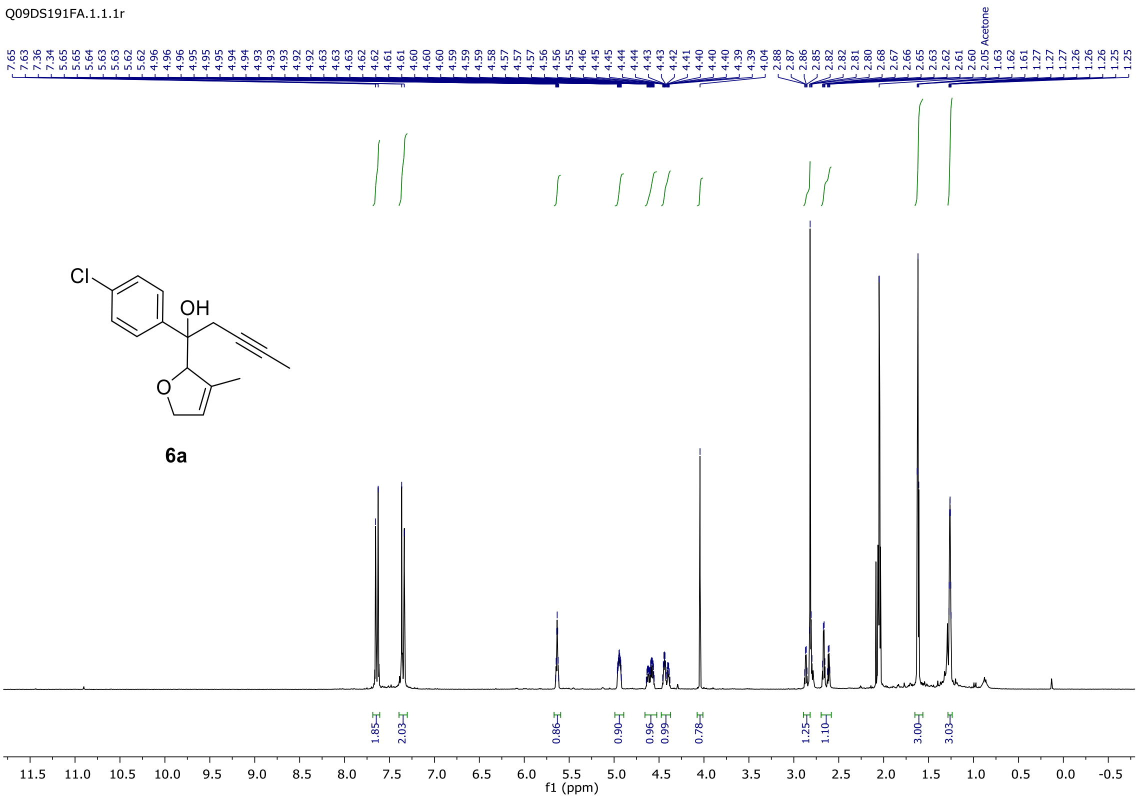

^{13}C NMR (75 MHz, acetone-d$_6$, rt)

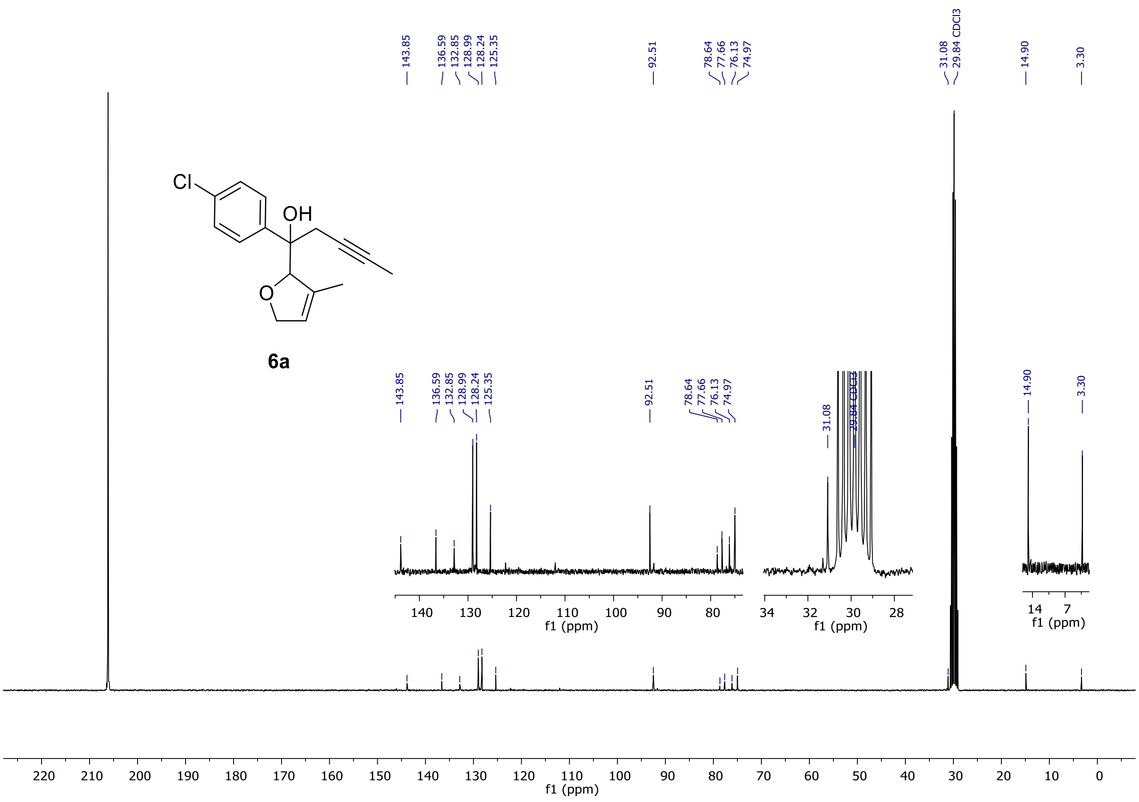

^{1}H NMR (300 MHz, CD_3CN, rt)

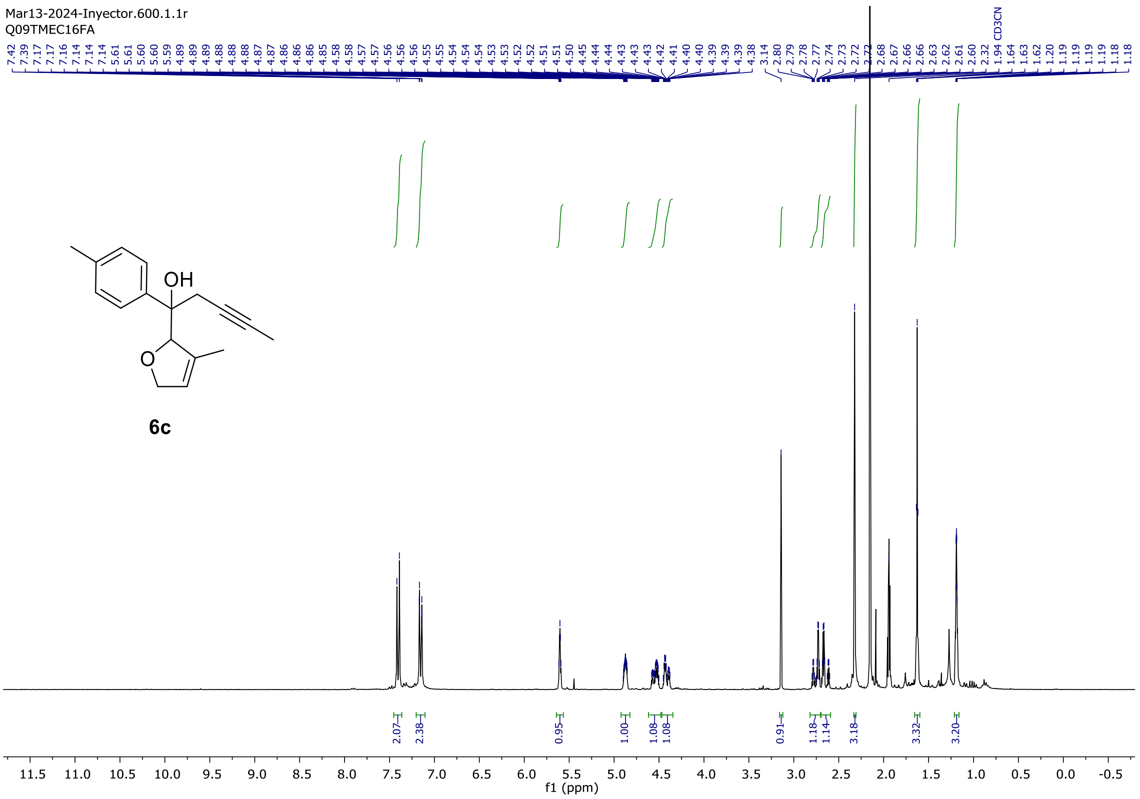

^{13}C NMR (75 MHz, CD_3CN, rt)

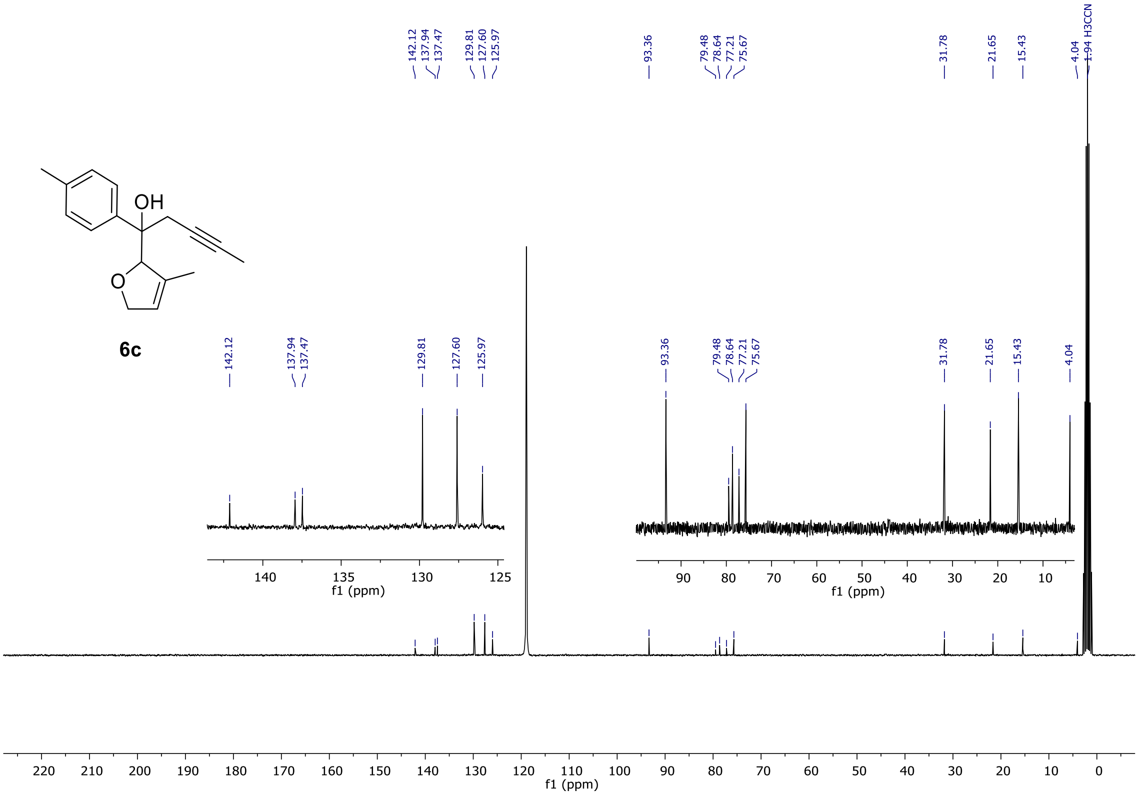

^{1}H NMR (300 MHz, acetone-d$_6$, rt)

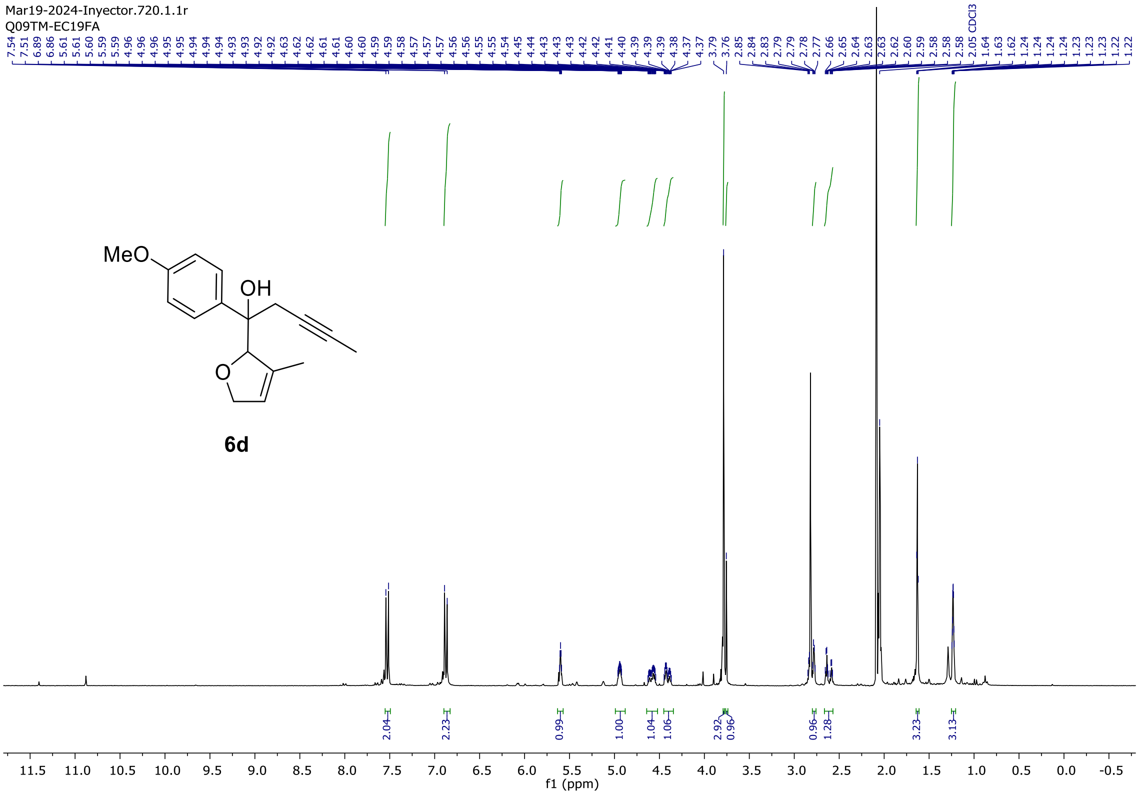

^{13}C NMR (75 MHz, acetone-d$_6$, rt)

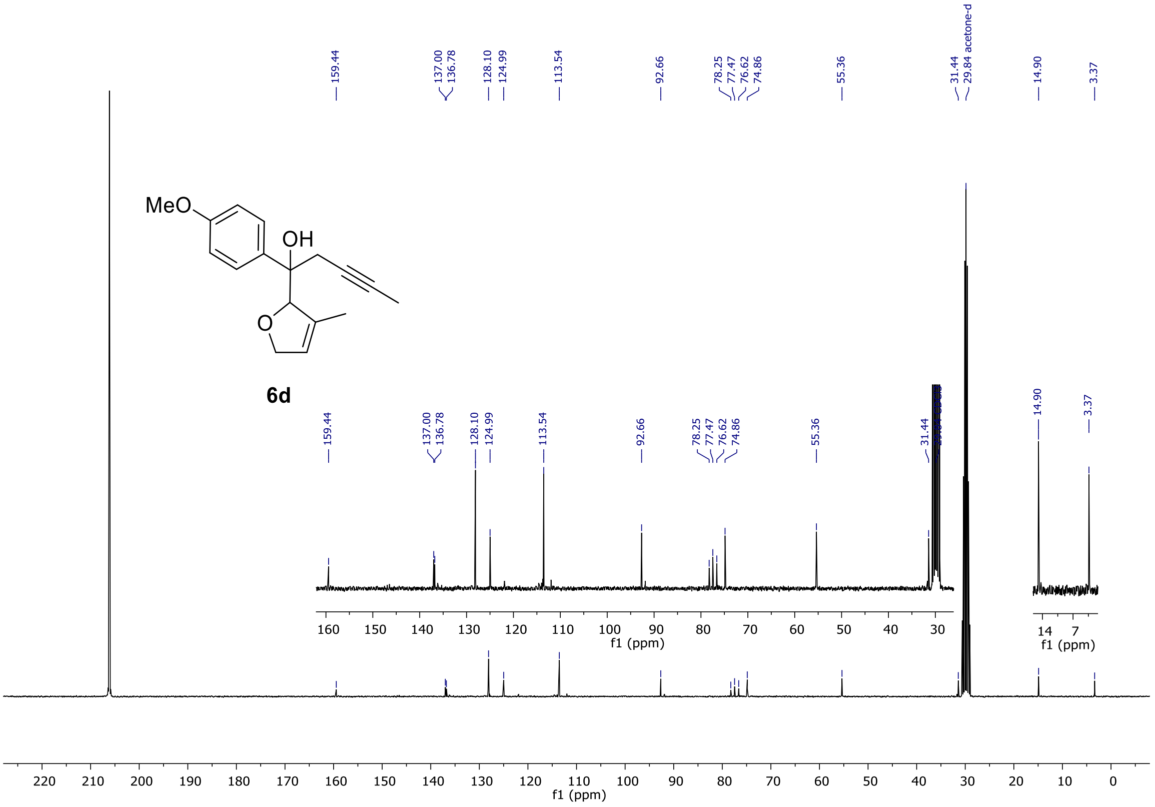

^{1}H NMR (300 MHz, CD_3CN, rt)

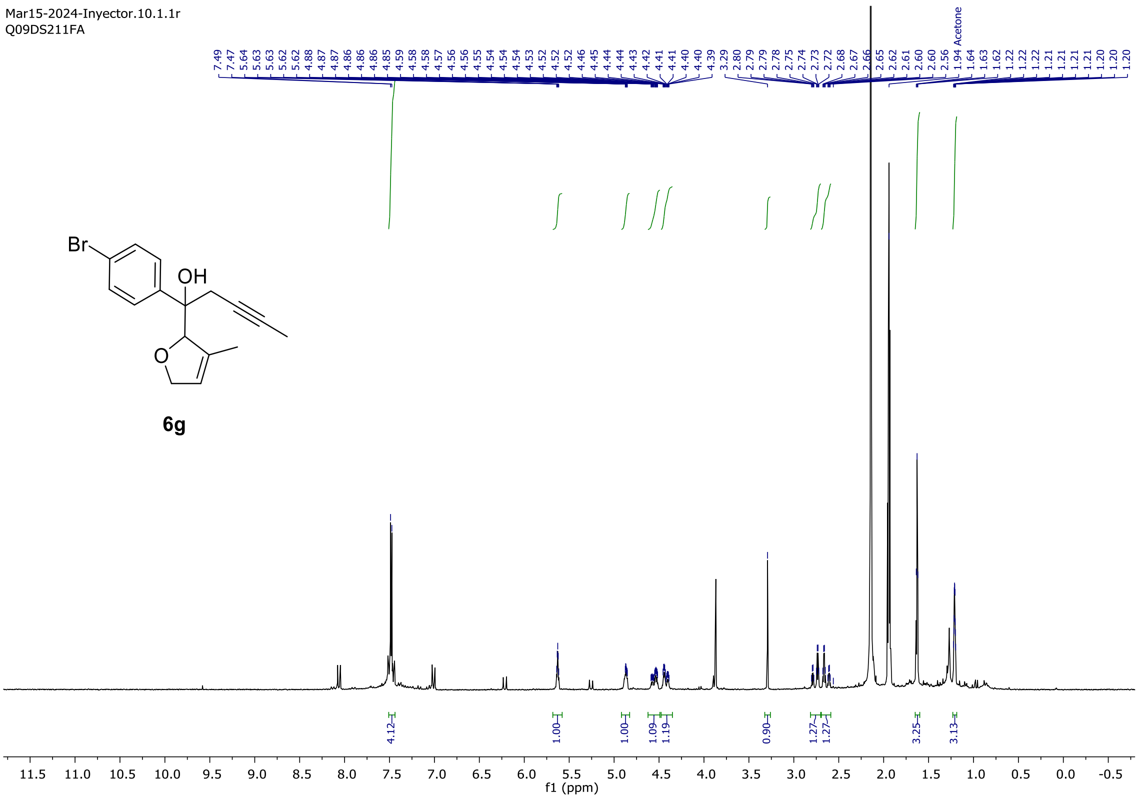

^{13}C NMR (75 MHz, CD_3CN, rt)

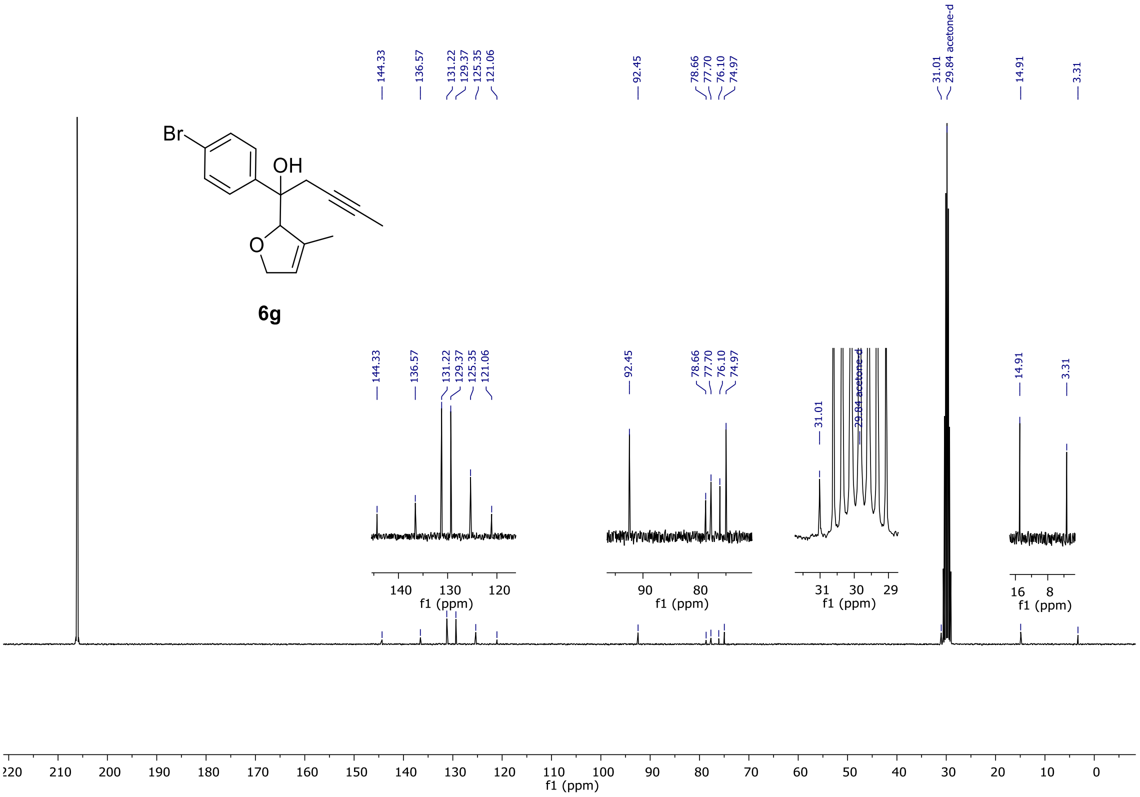

^{1}H NMR (300 MHz, acetone-d$_6$, rt)

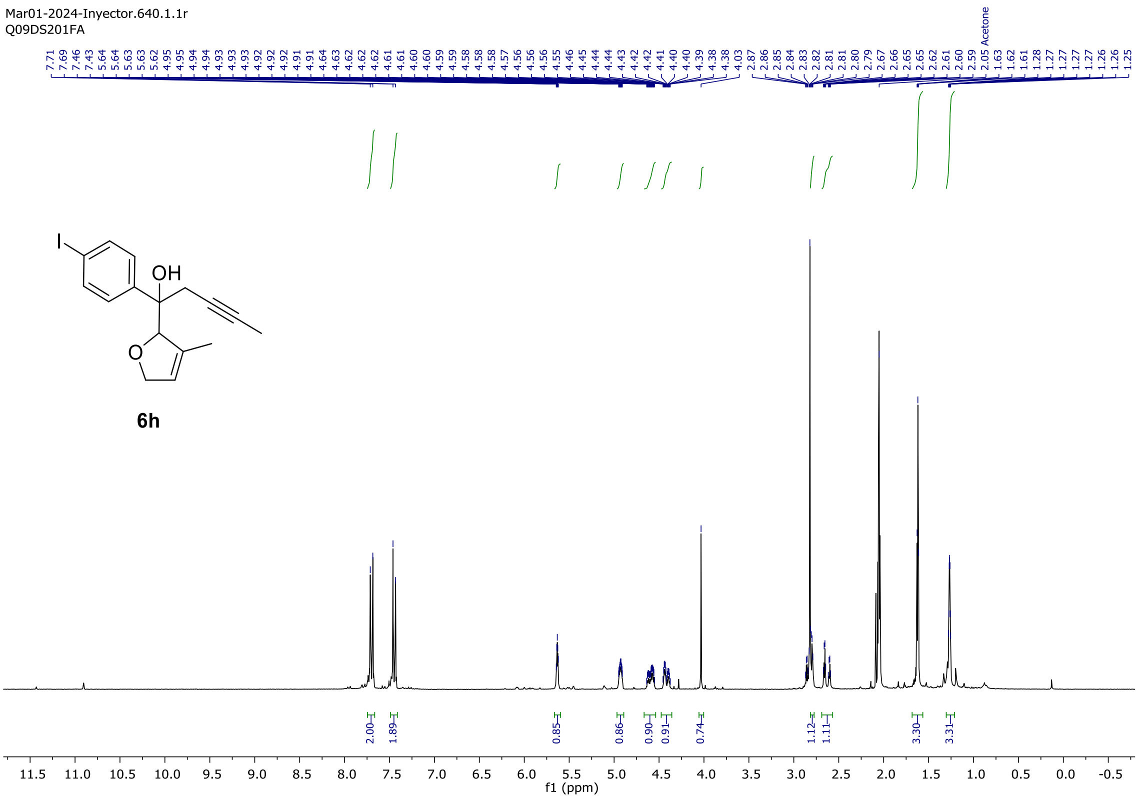

^{13}C NMR (75 MHz, acetone-d$_6$, rt)

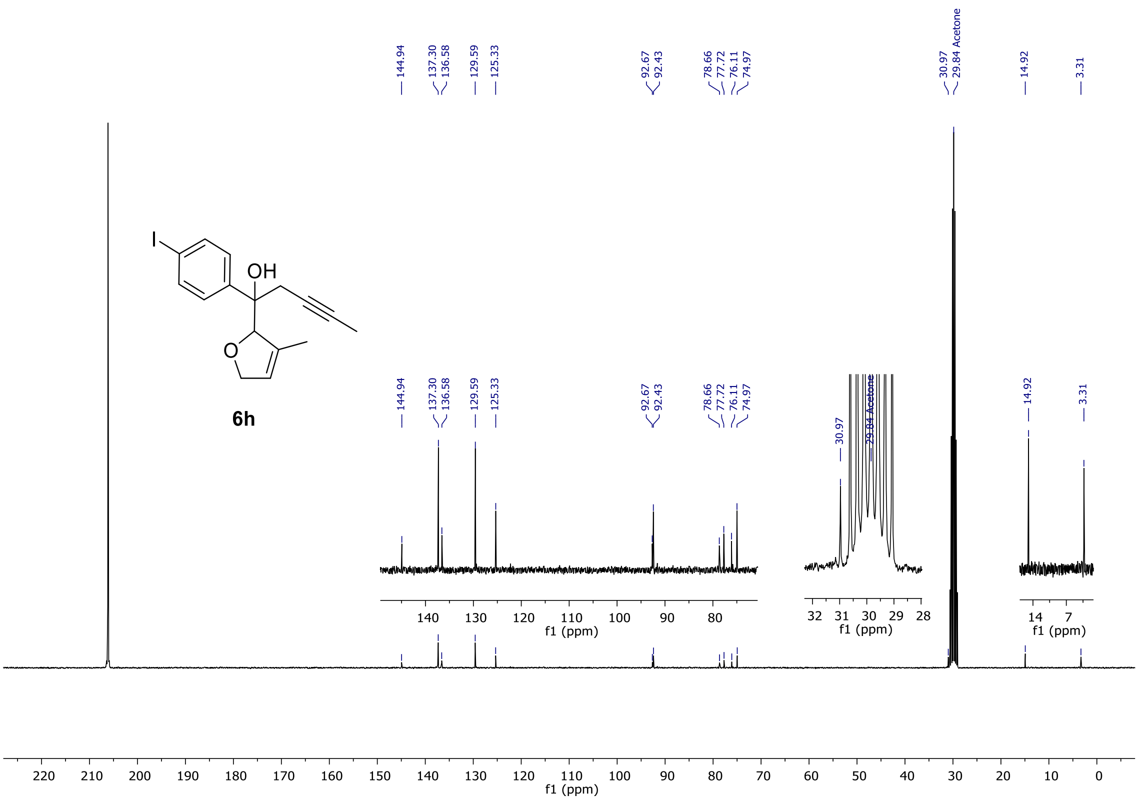

^{1}H NMR (300 MHz, acetone-d$_6$, rt)

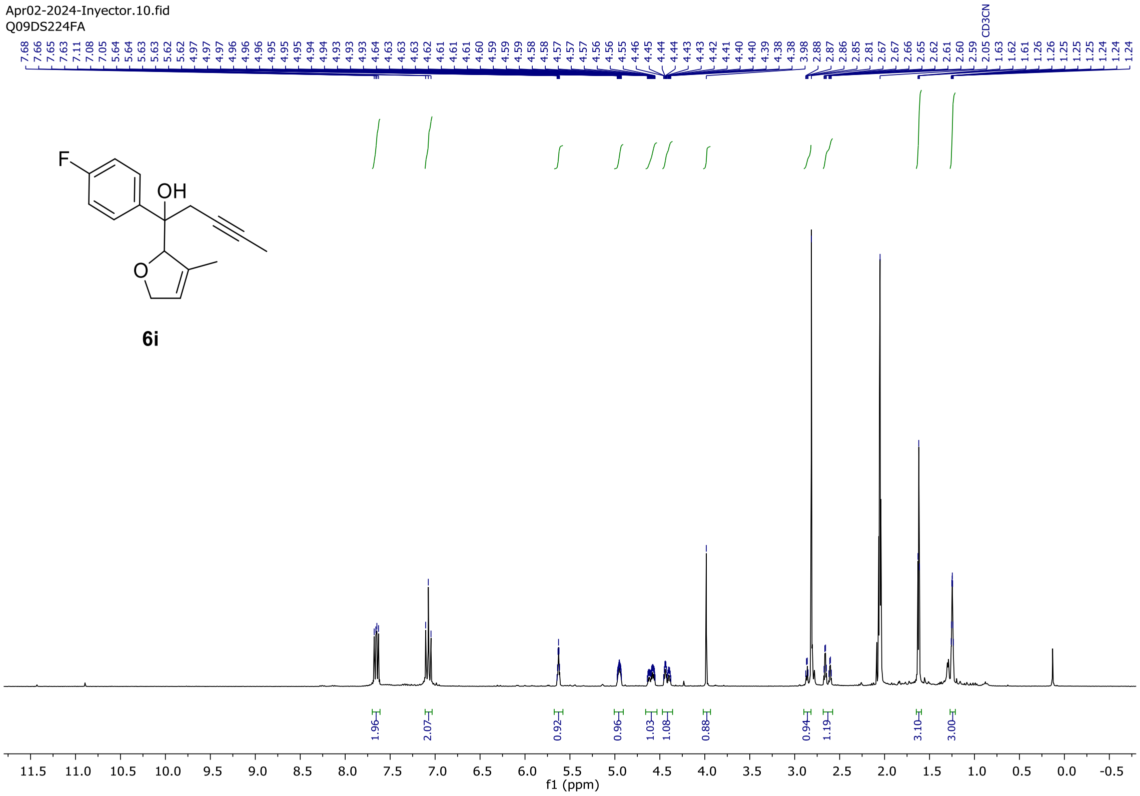

^{13}C NMR (75 MHz, acetone-d$_6$, rt)

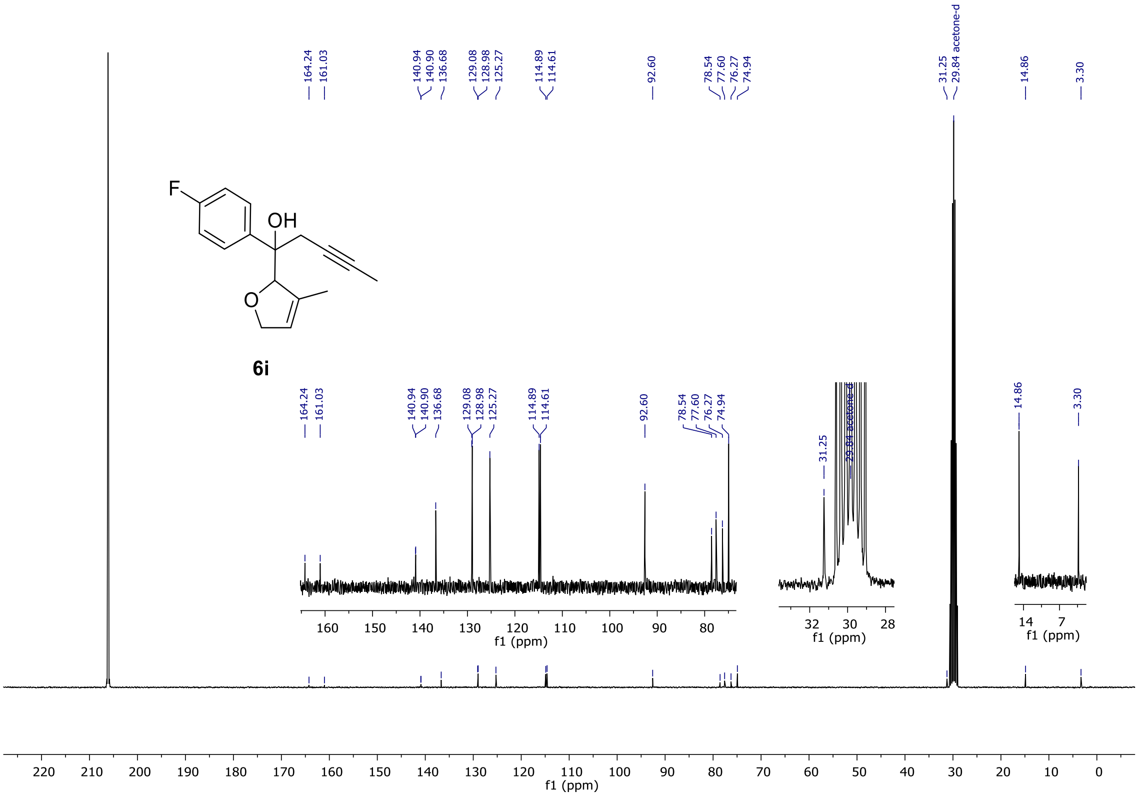

^{1}H NMR (300 MHz, CD_3CN, rt)

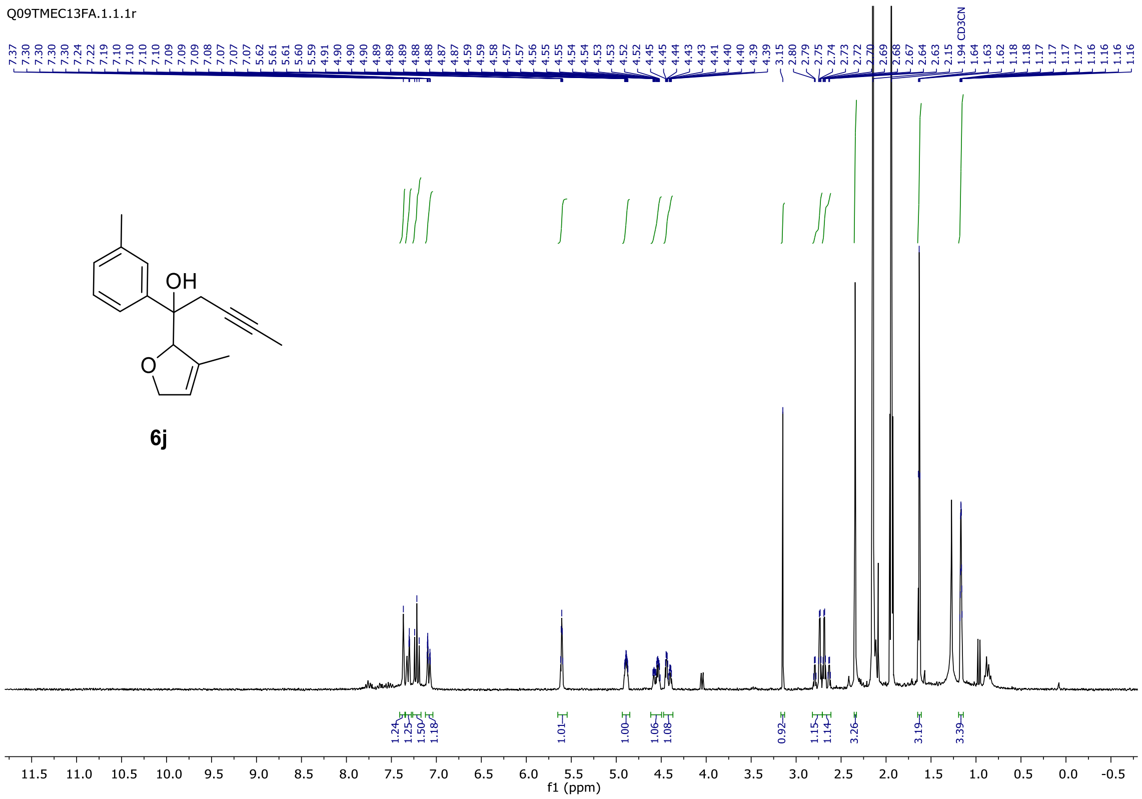

^{13}C NMR (75 MHz, acetone-d_6, rt)

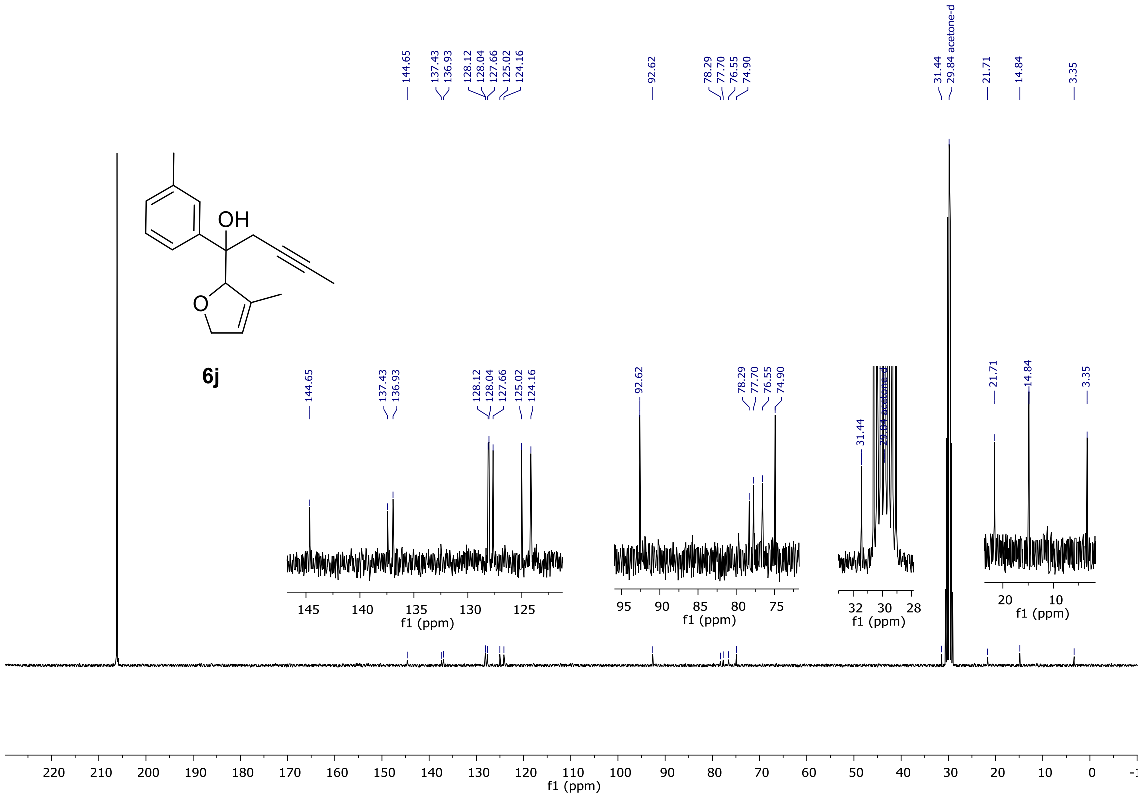

^{1}H NMR (300 MHz, acetone-d$_6$, rt)

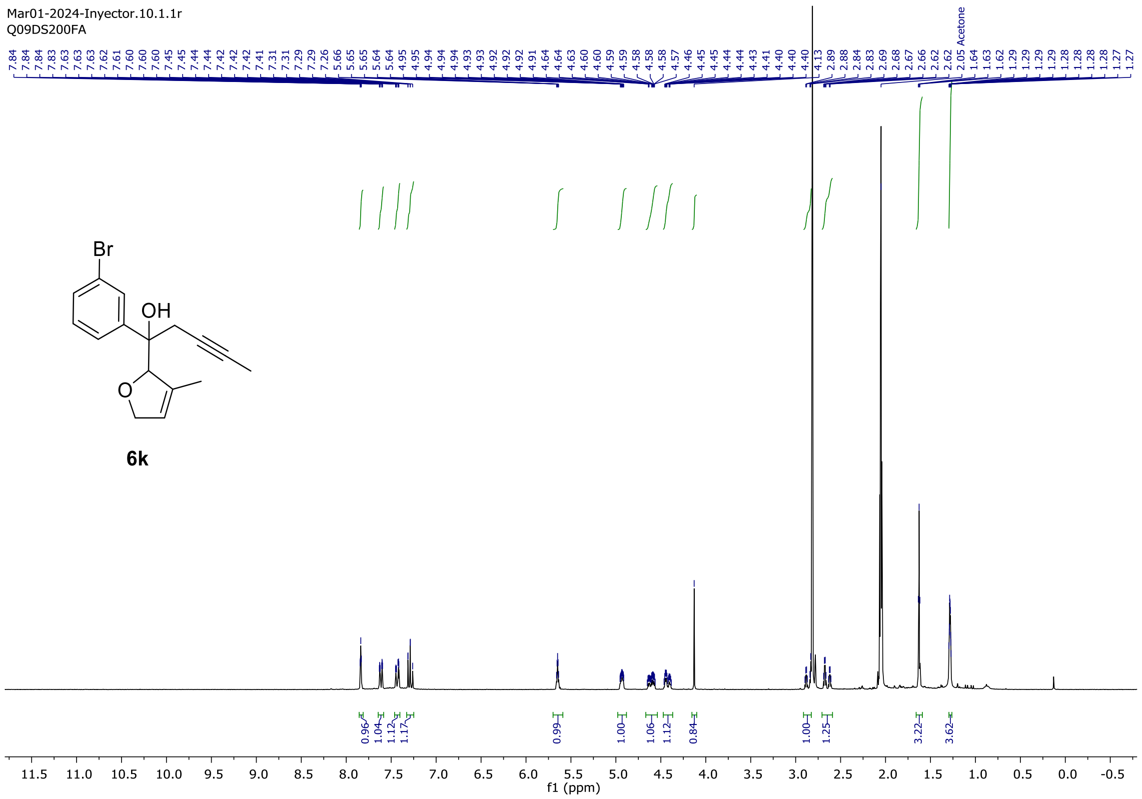

^{13}C NMR (75 MHz, acetone-d$_6$, rt)

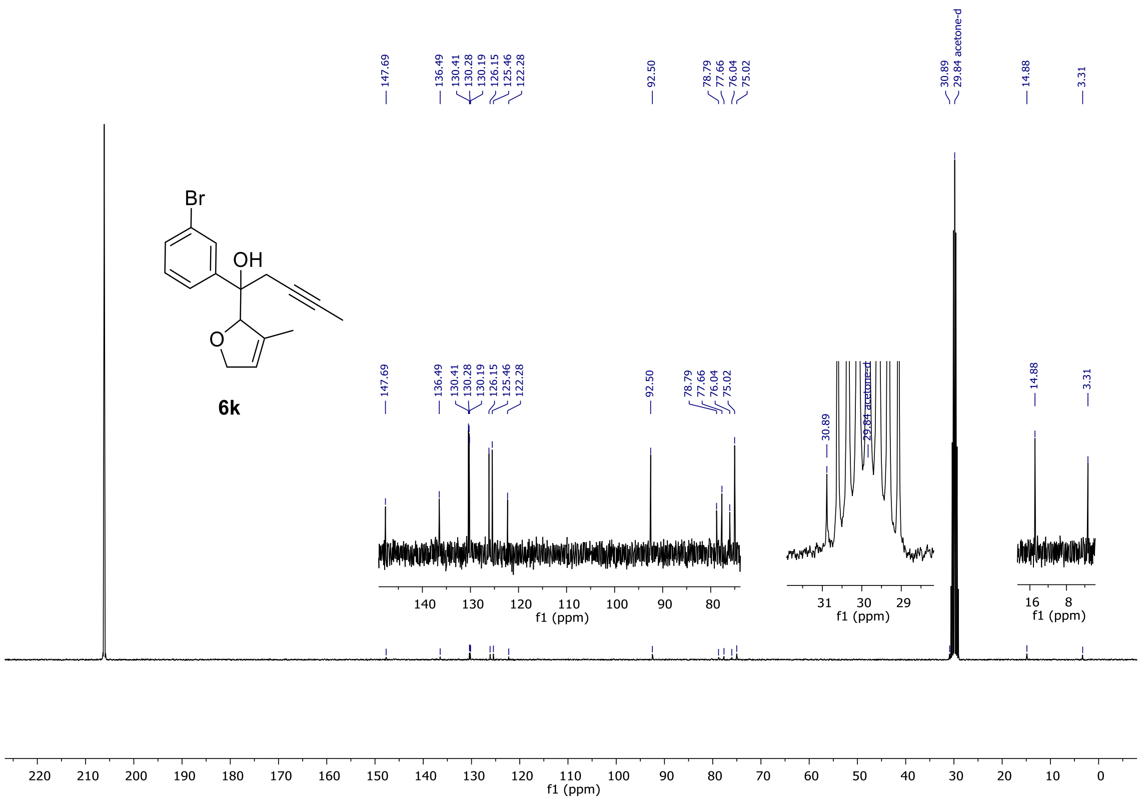

^{1}H NMR (300 MHz, acetone-d$_6$, rt)

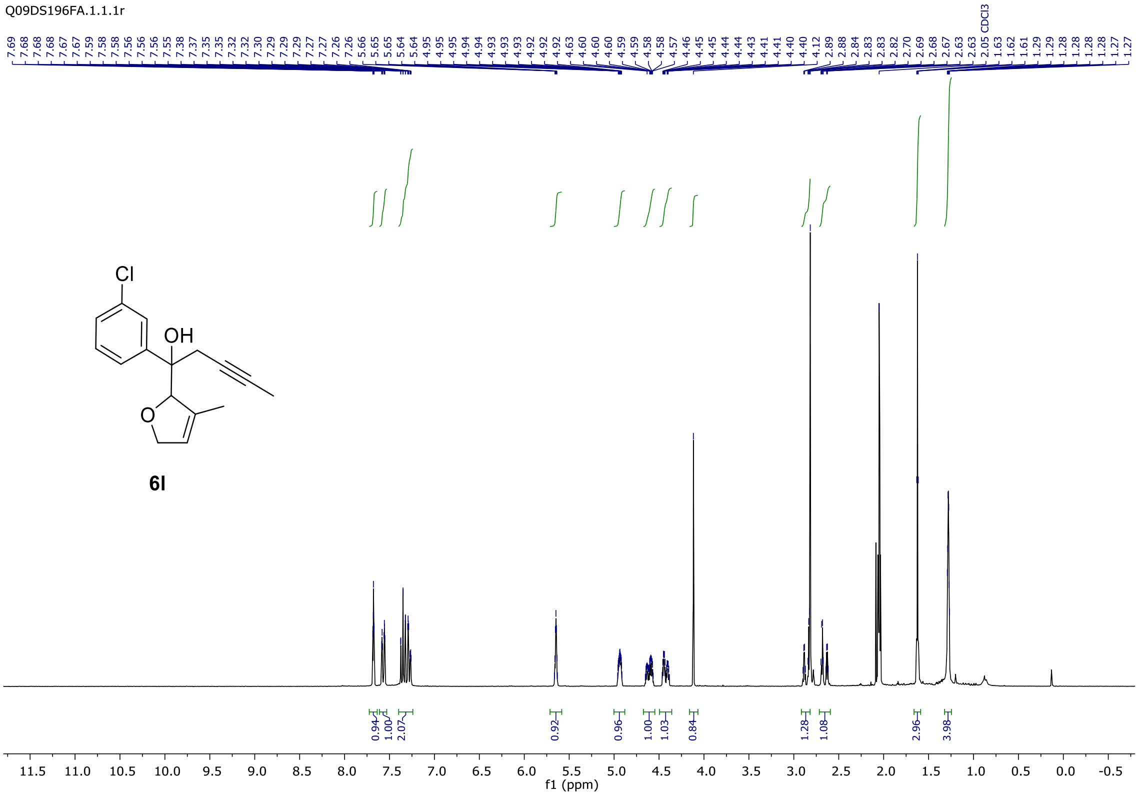

^{13}C NMR (75 MHz, acetone-d$_6$, rt)

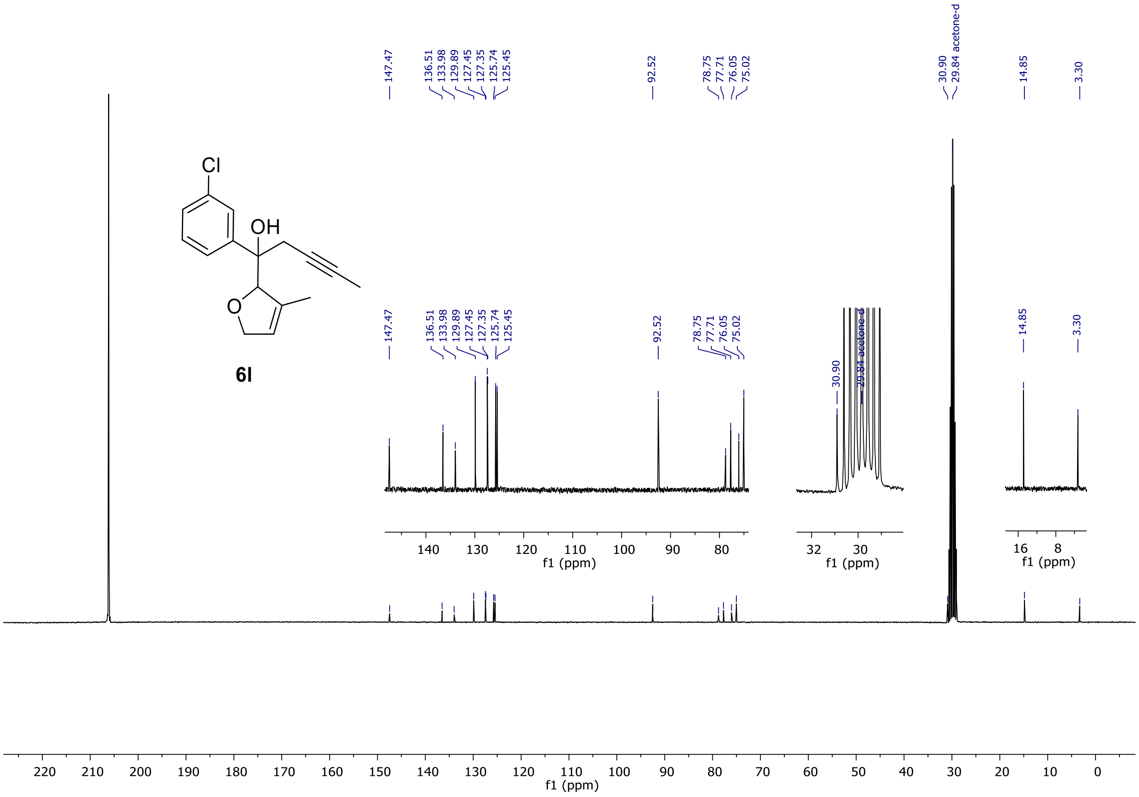

^{1}H NMR (300 MHz, acetone-d$_6$, rt)

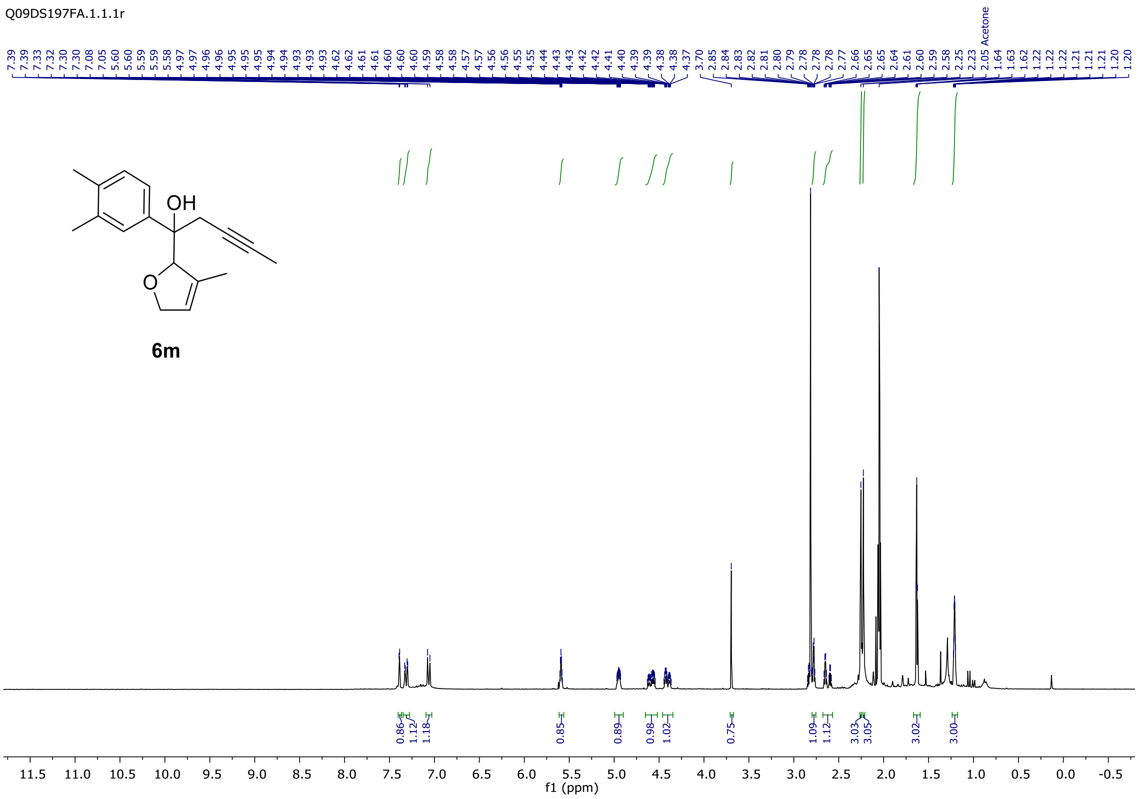

^{13}C NMR (75 MHz, acetone-d$_6$, rt)

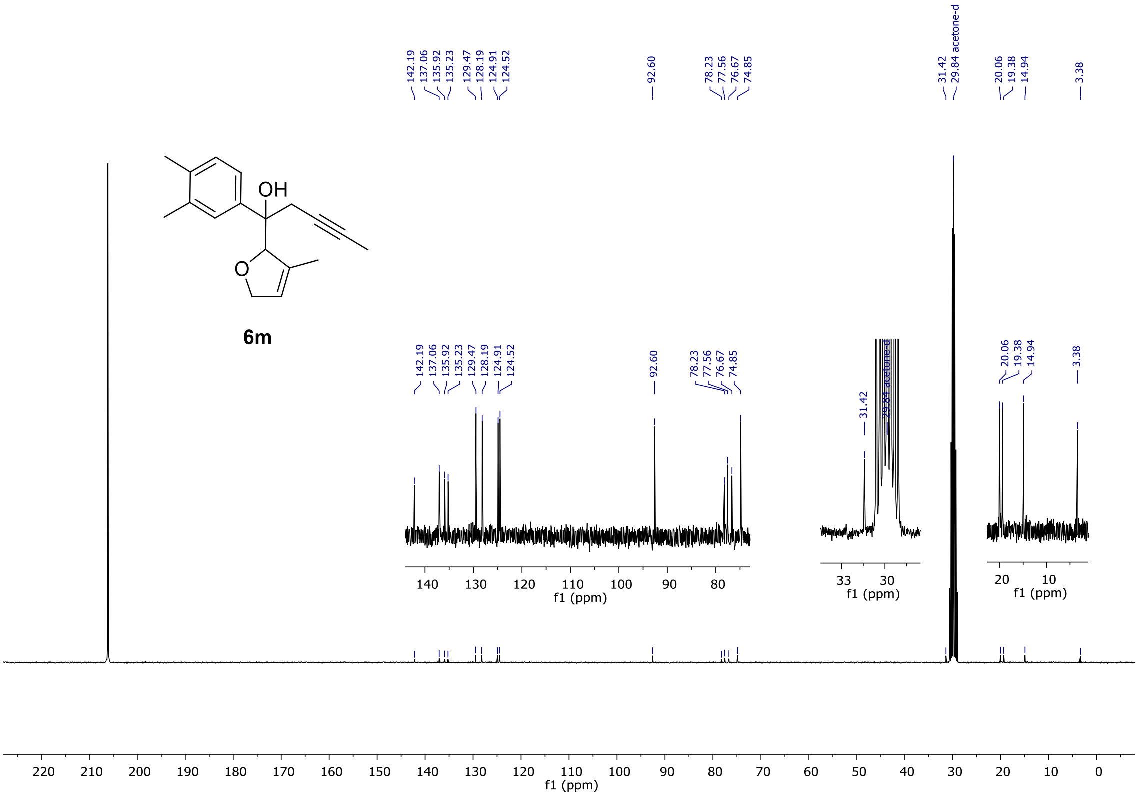

^{1}H NMR (300 MHz, acetone-d$_6$, rt)

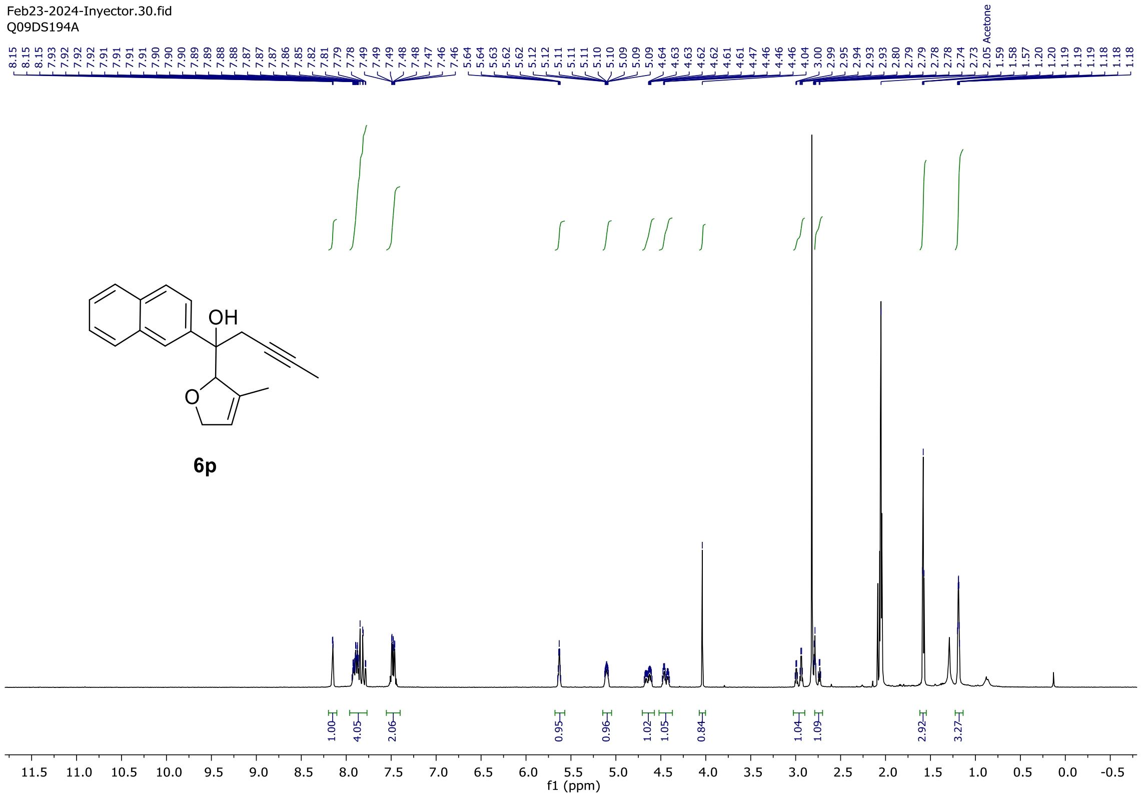

^{13}C NMR (75 MHz, acetone-d$_6$, rt)

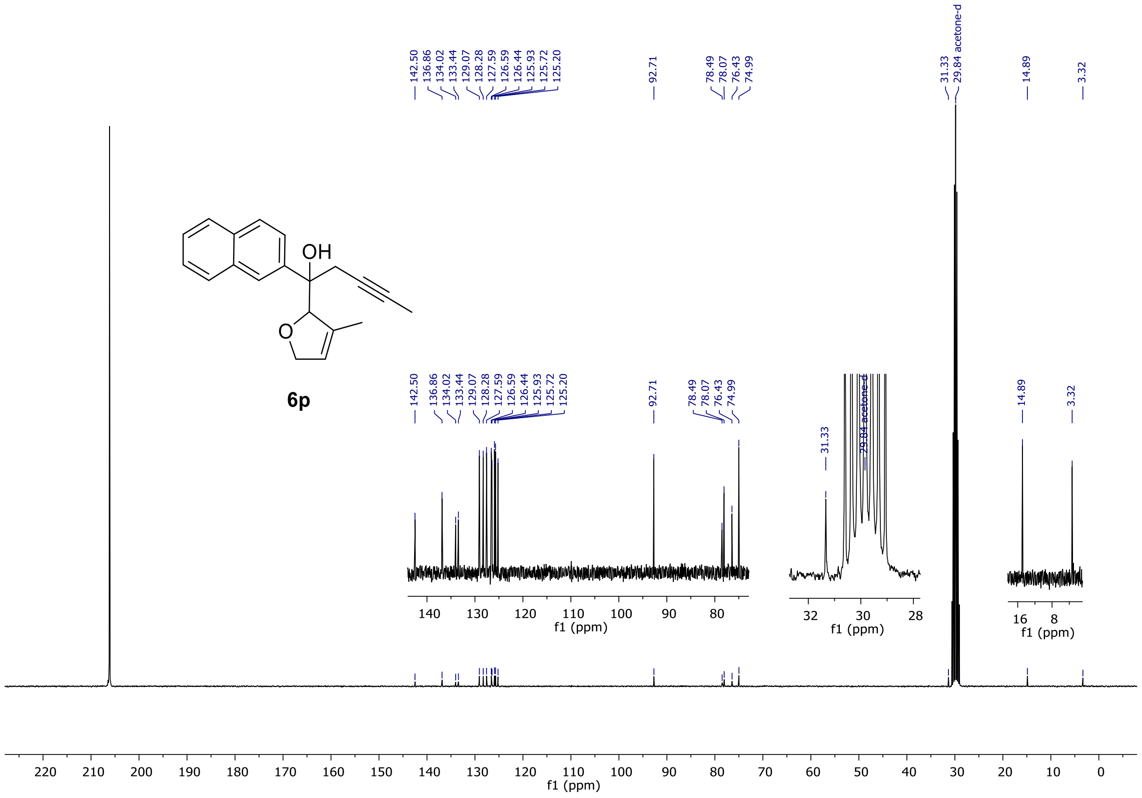

^1H NMR (300 MHz, acetone-d_6, rt)

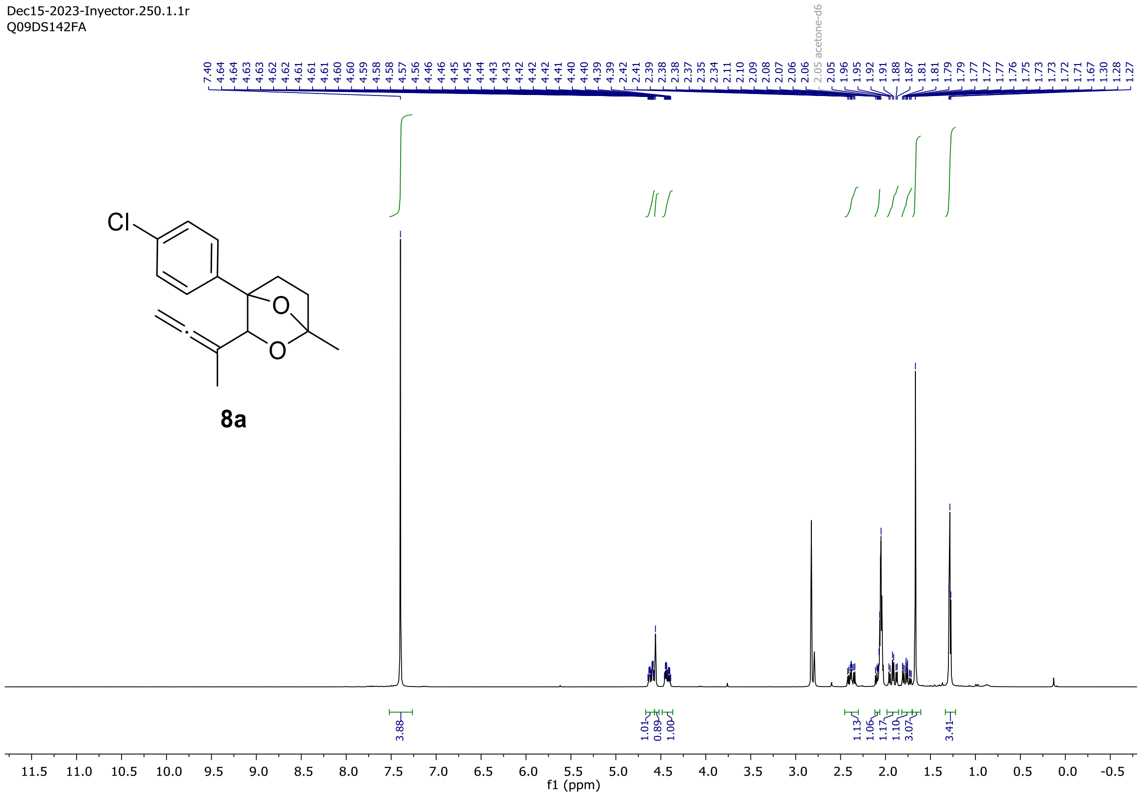

^{13}C NMR (75 MHz, acetone-d_6, rt)

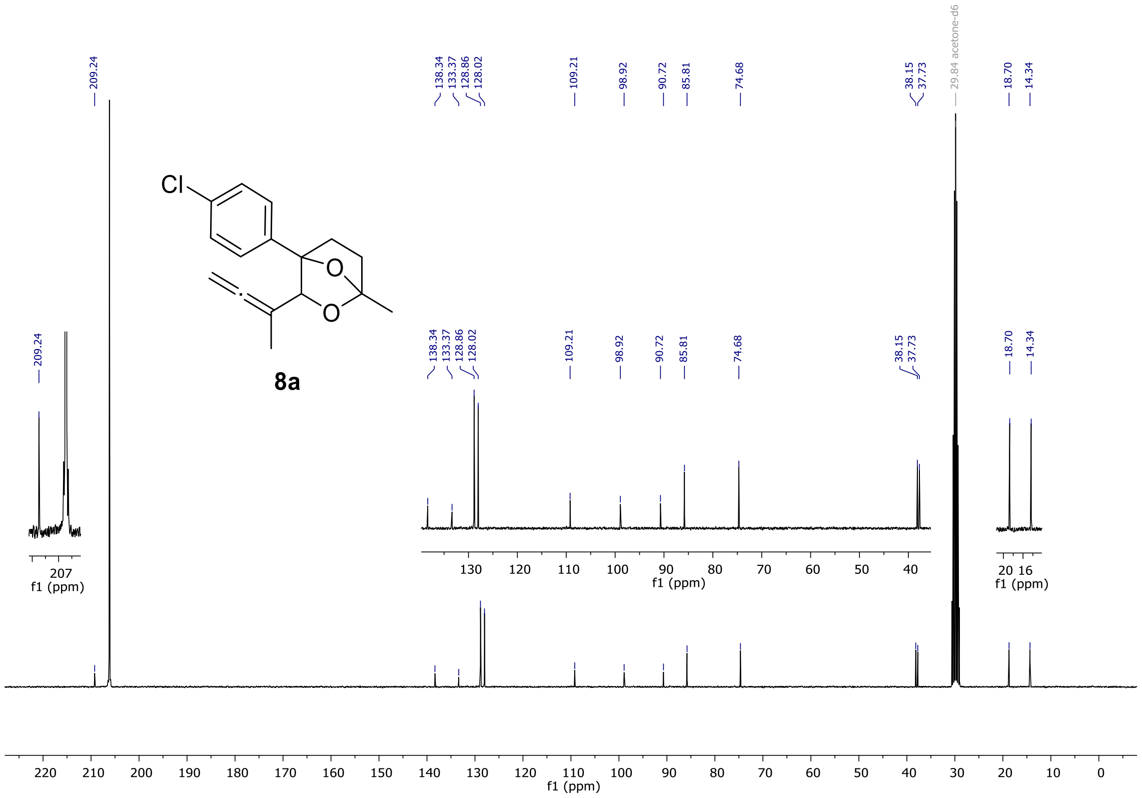

^{1}H NMR (300 MHz, acetone-d_6, rt)

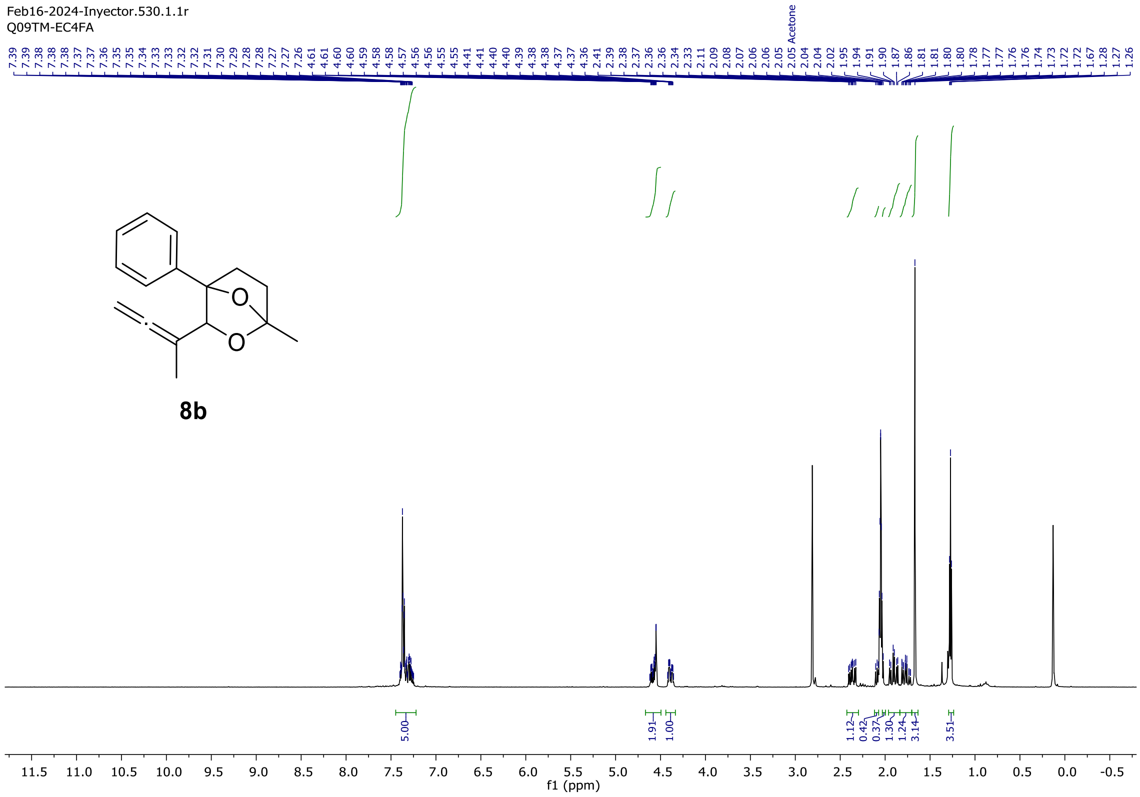

^{13}C NMR (75 MHz, acetone-d_6, rt)

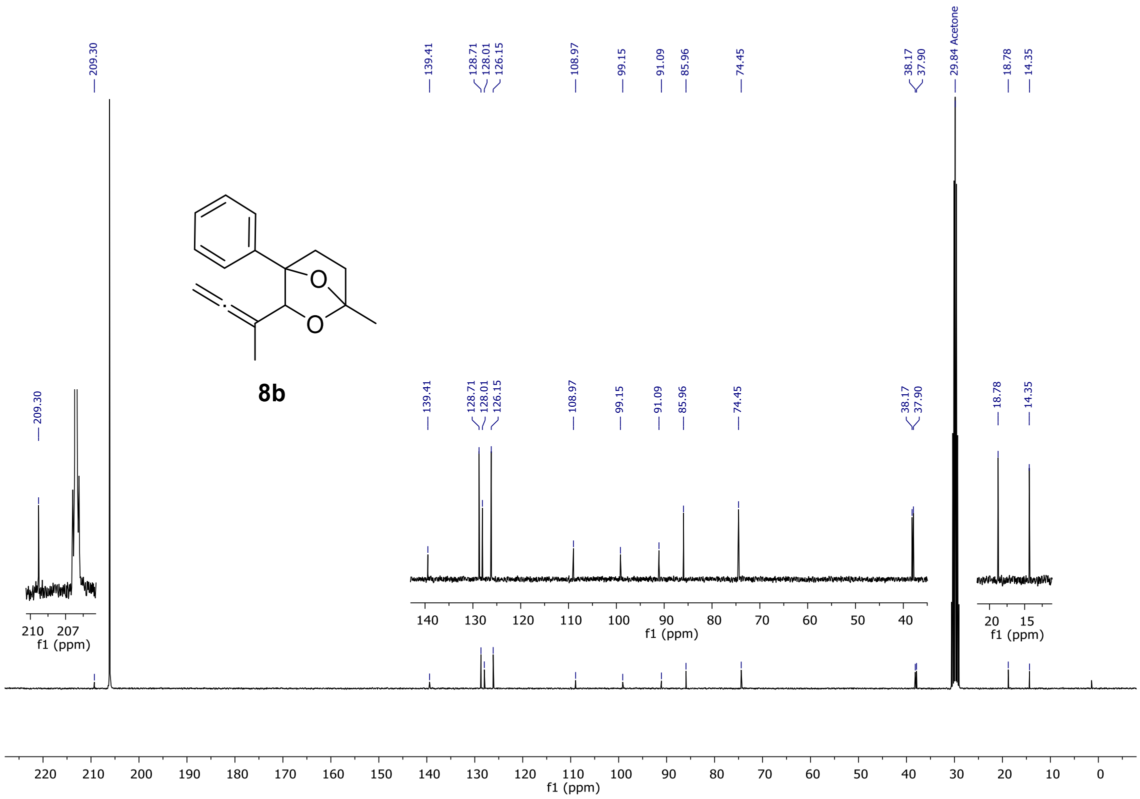

^{1}H NMR (300 MHz, CD_3CN, rt)

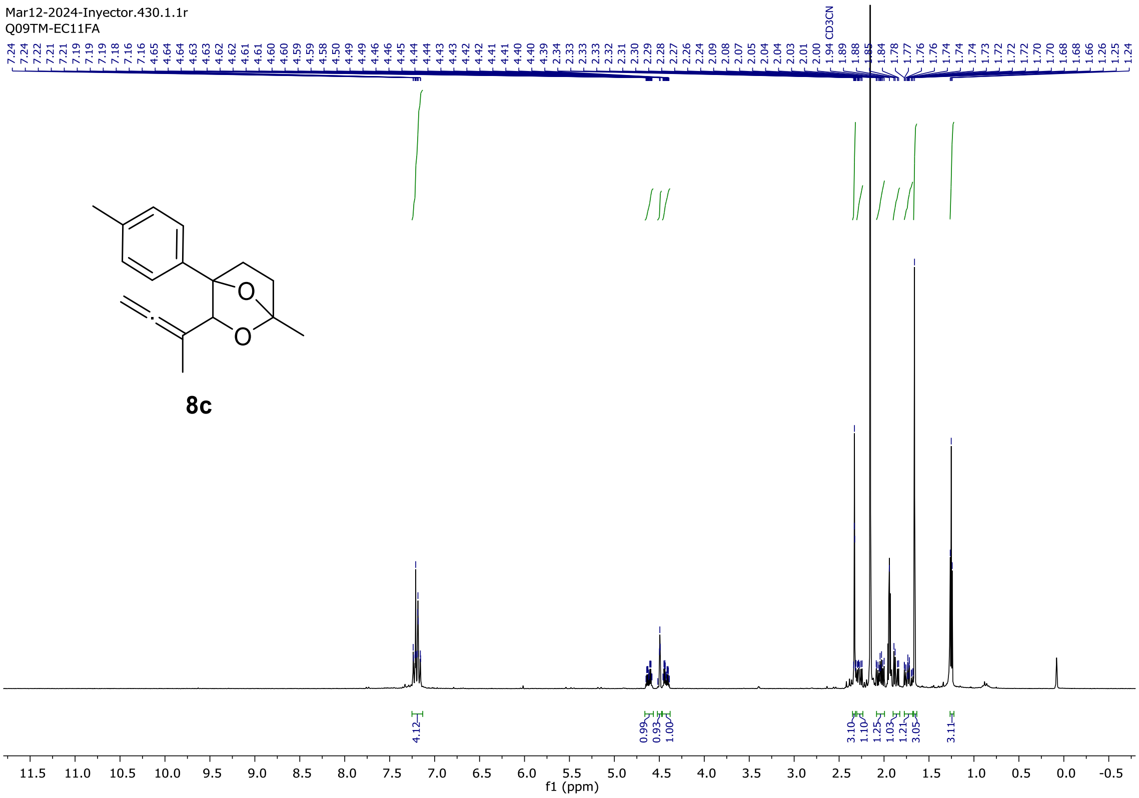

^{13}C NMR (75 MHz, CD_3CN, rt)

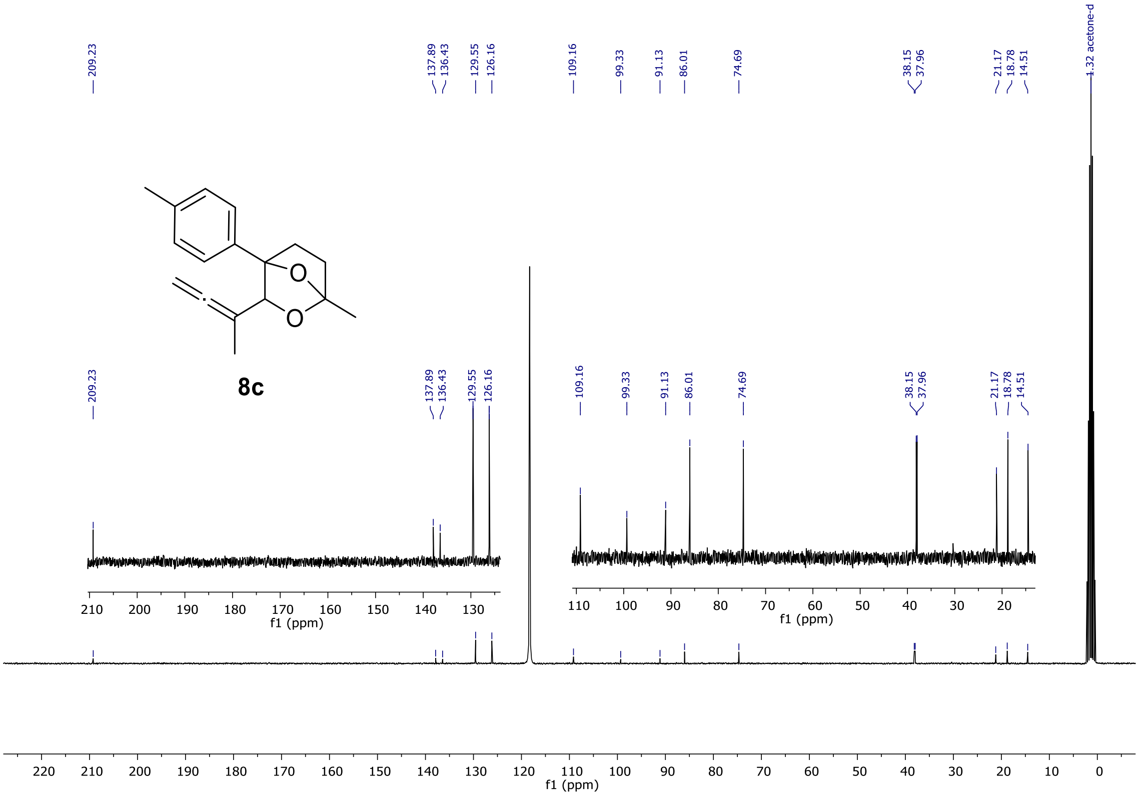

^{1}H NMR (300 MHz, acetone-d_6, rt)

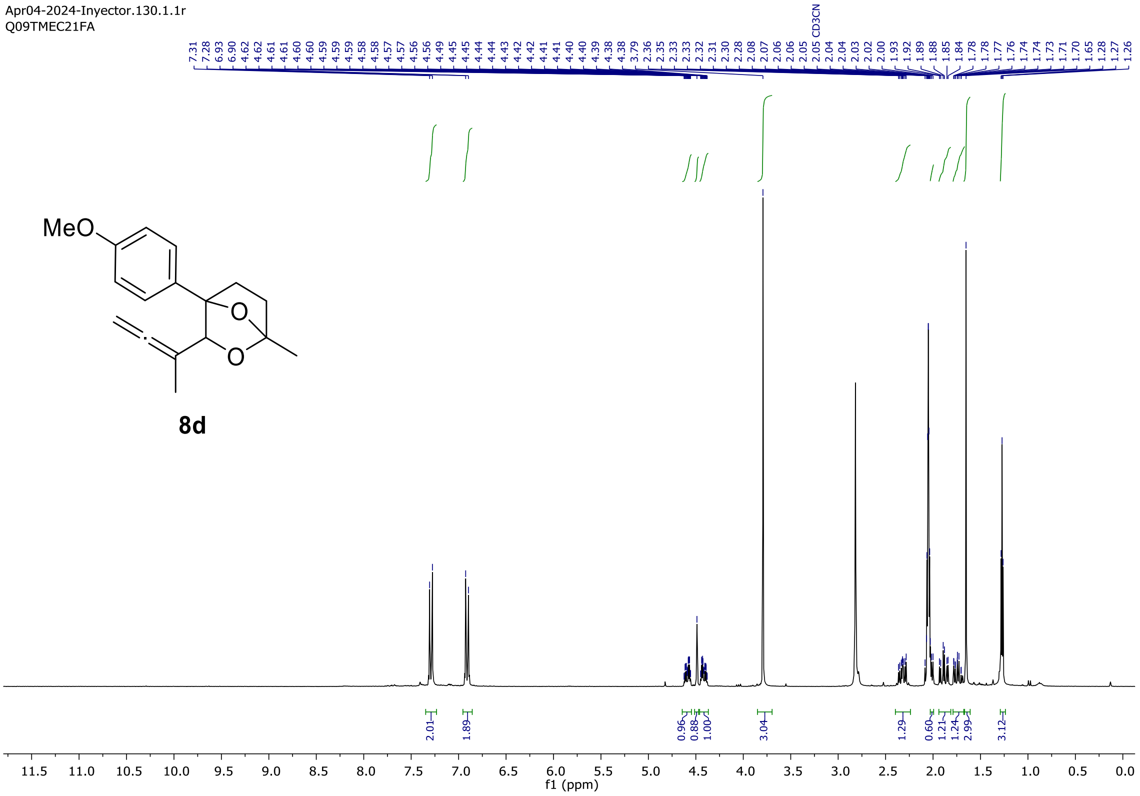

^{13}C NMR (75 MHz, acetone-d_6, rt)

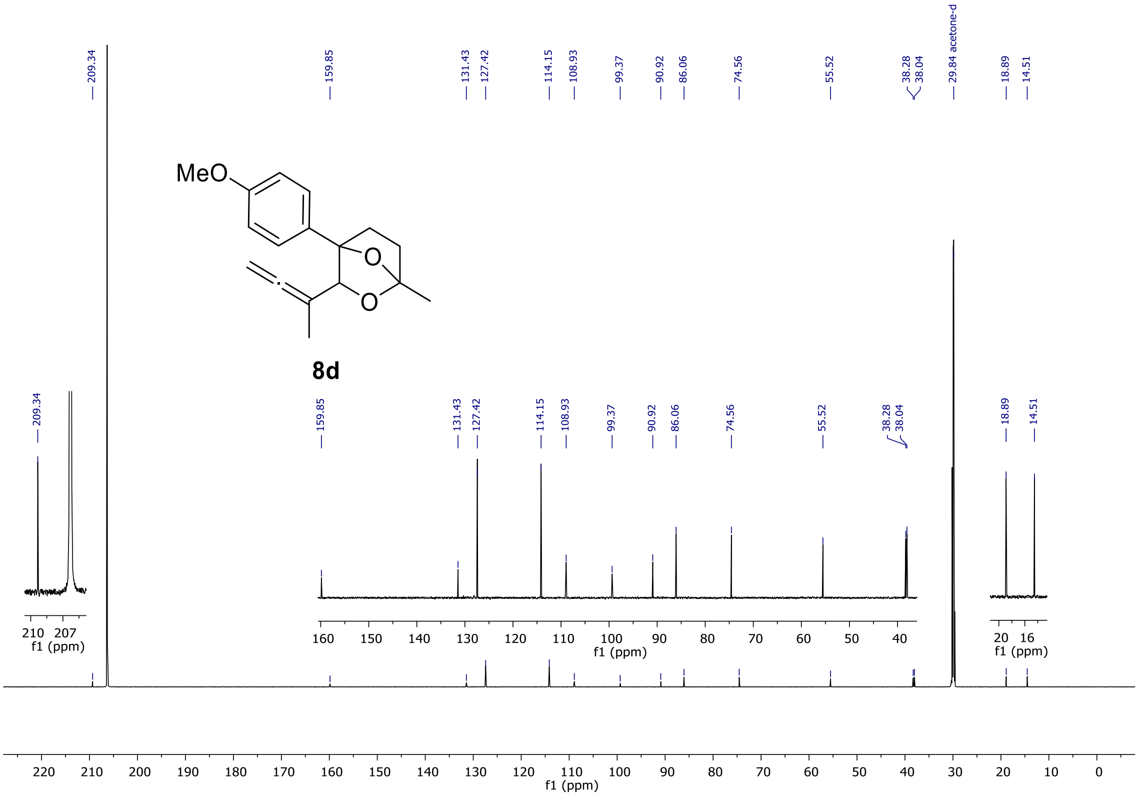

^{1}H NMR (700 MHz, acetone-d6, rt)

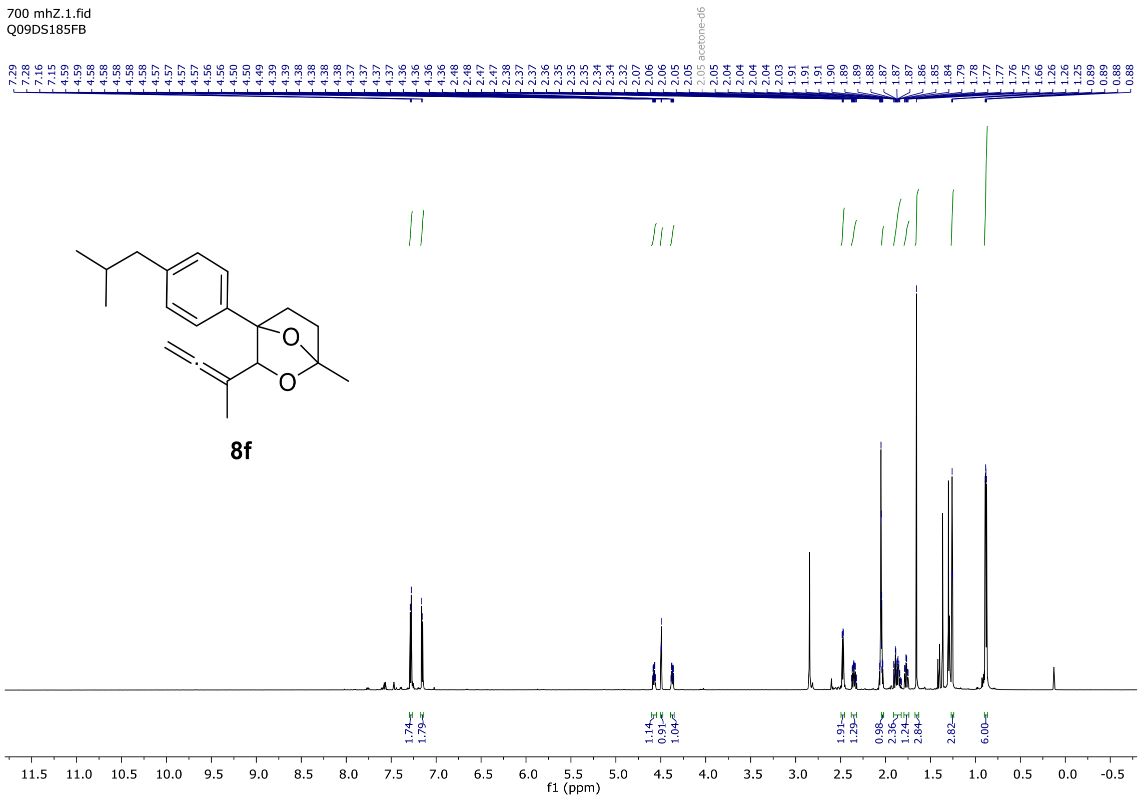

^{13}C NMR (175 MHz, acetone-d6, rt)

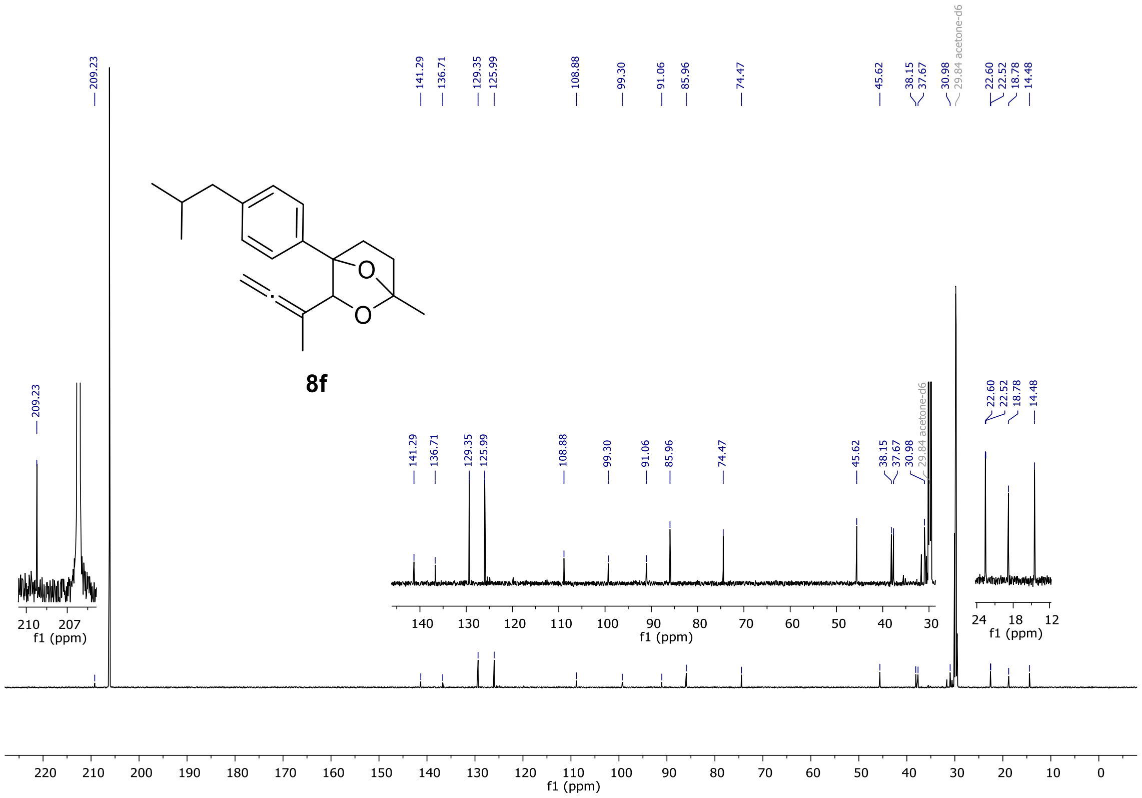

^1H NMR (300 MHz, acetone-d_6, rt)

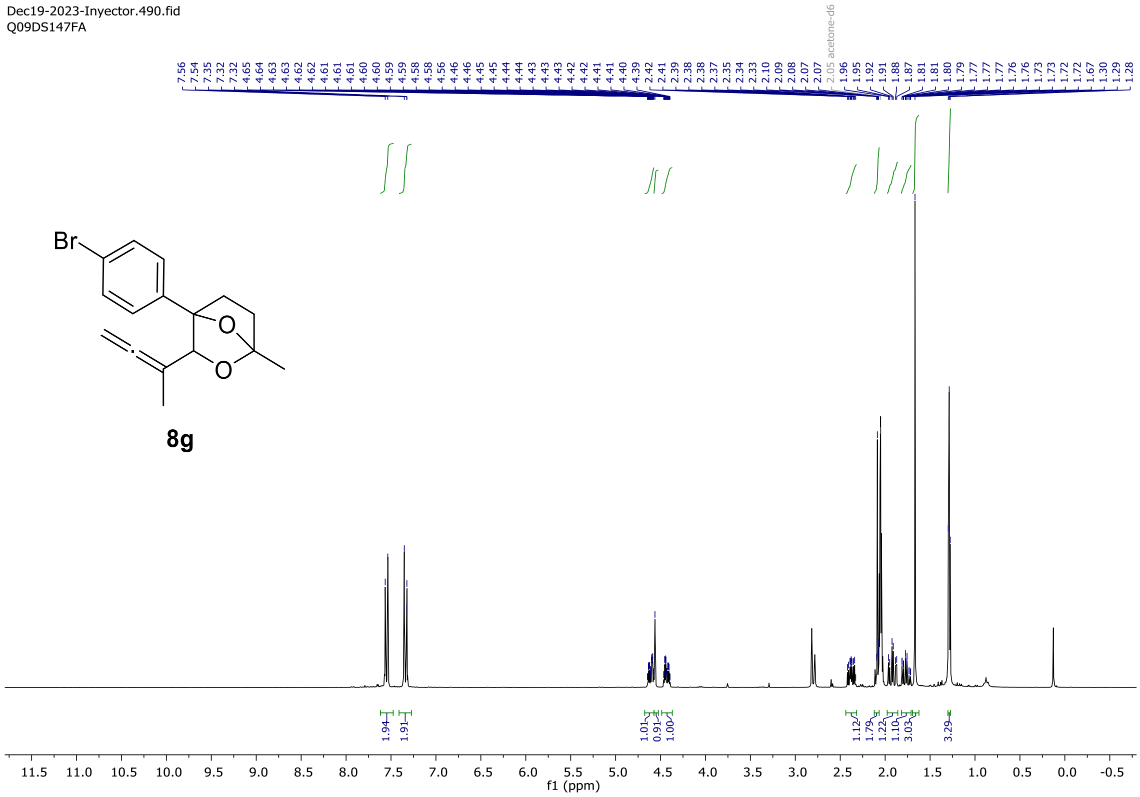

^{13}C NMR (75 MHz, acetone-d_6, rt)

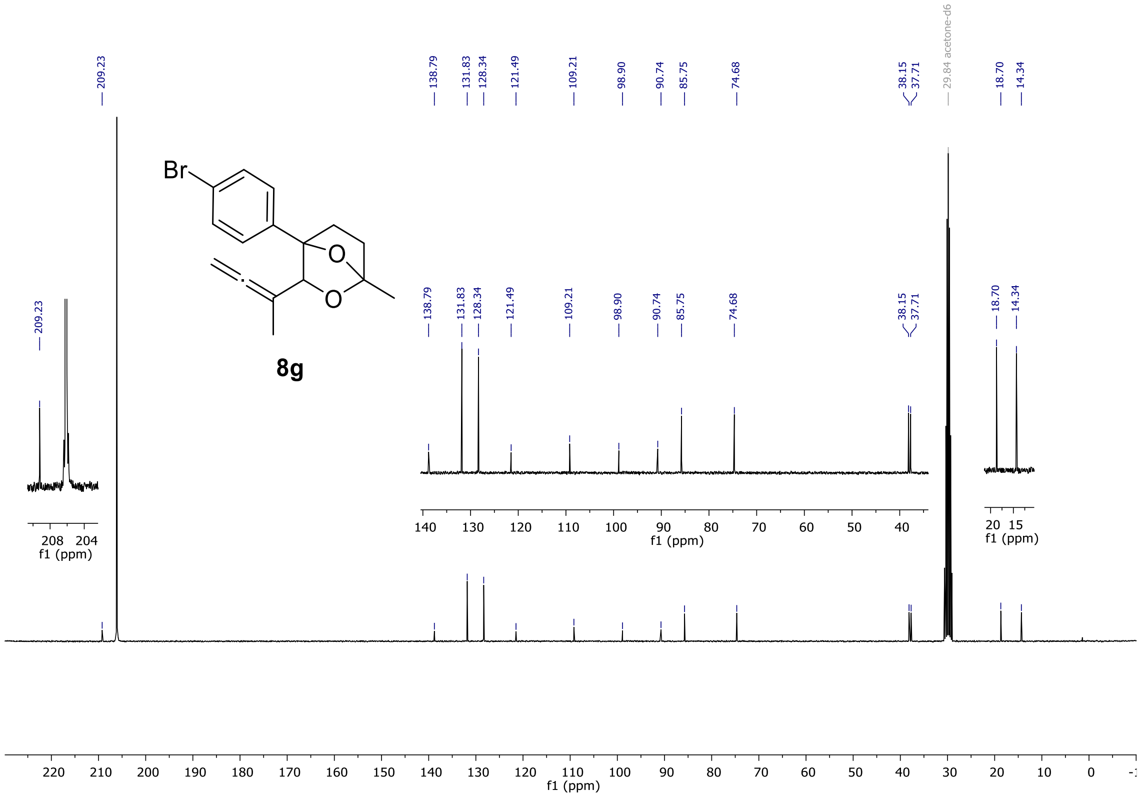

^{1}H NMR (700 MHz, acetone-d$_6$, rt)

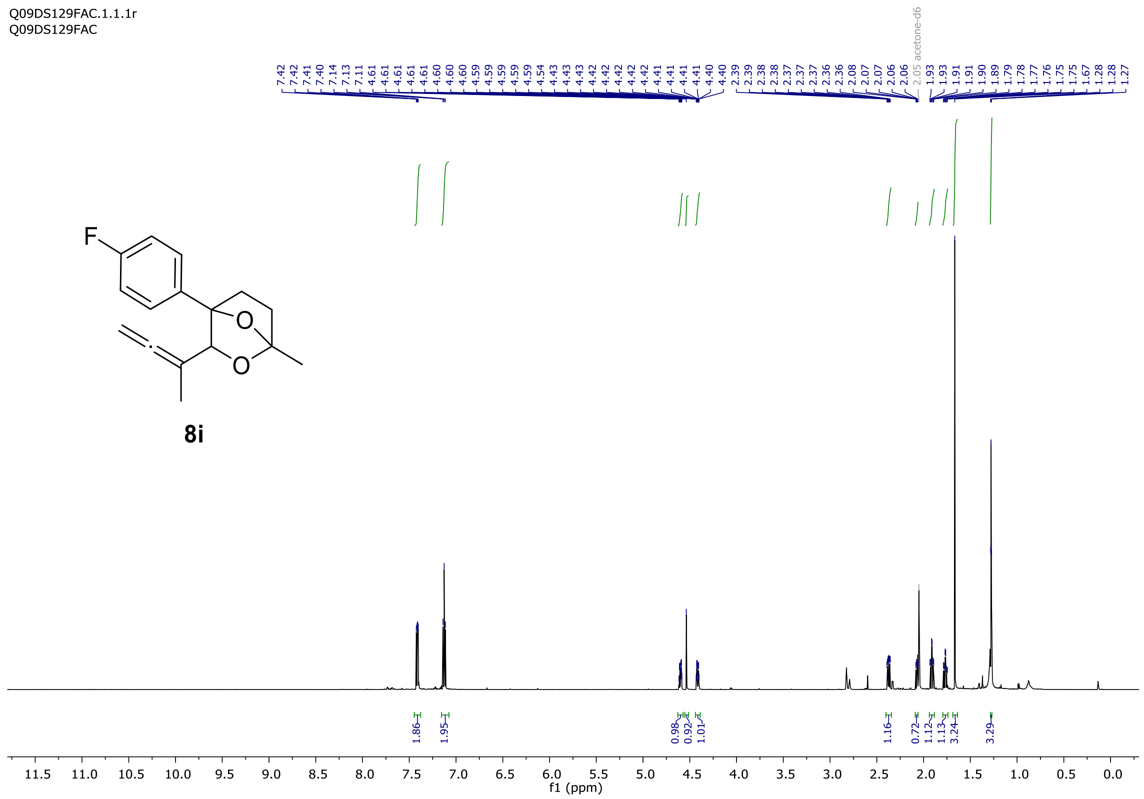

^{13}C NMR (175 MHz, acetone-d$_6$, rt)

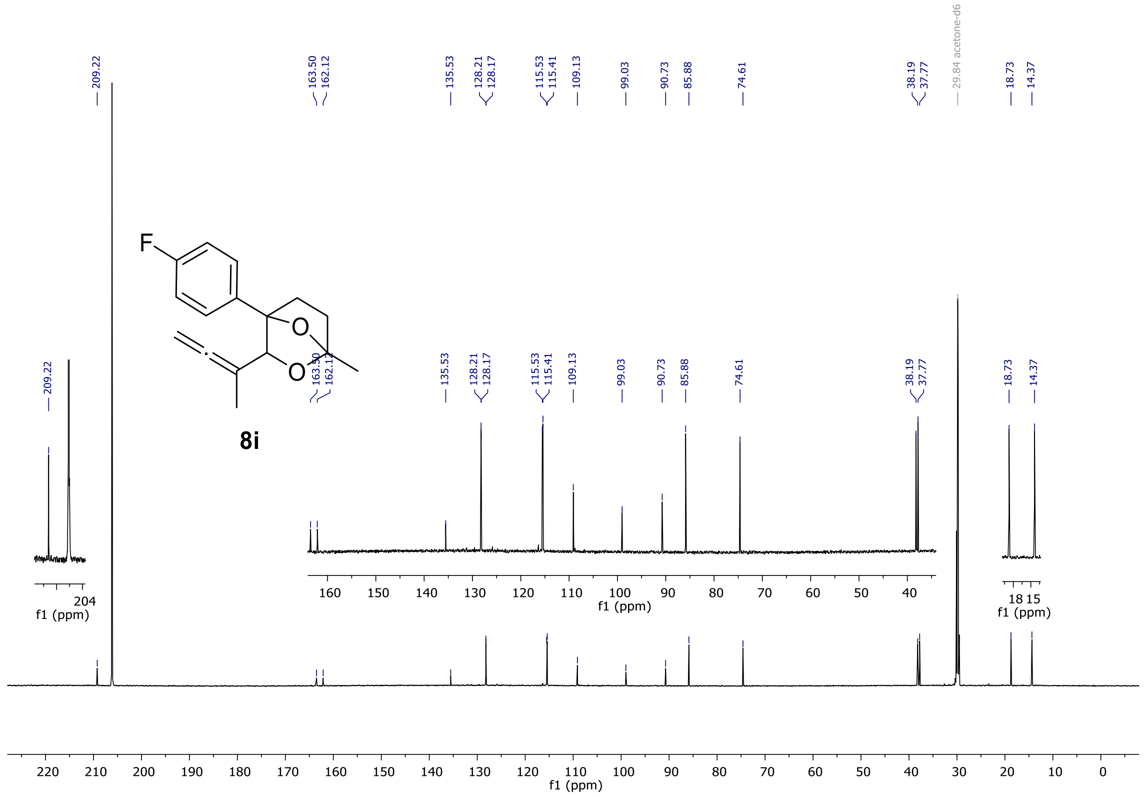

^1H NMR (300 MHz, acetone-d$_6$, rt)

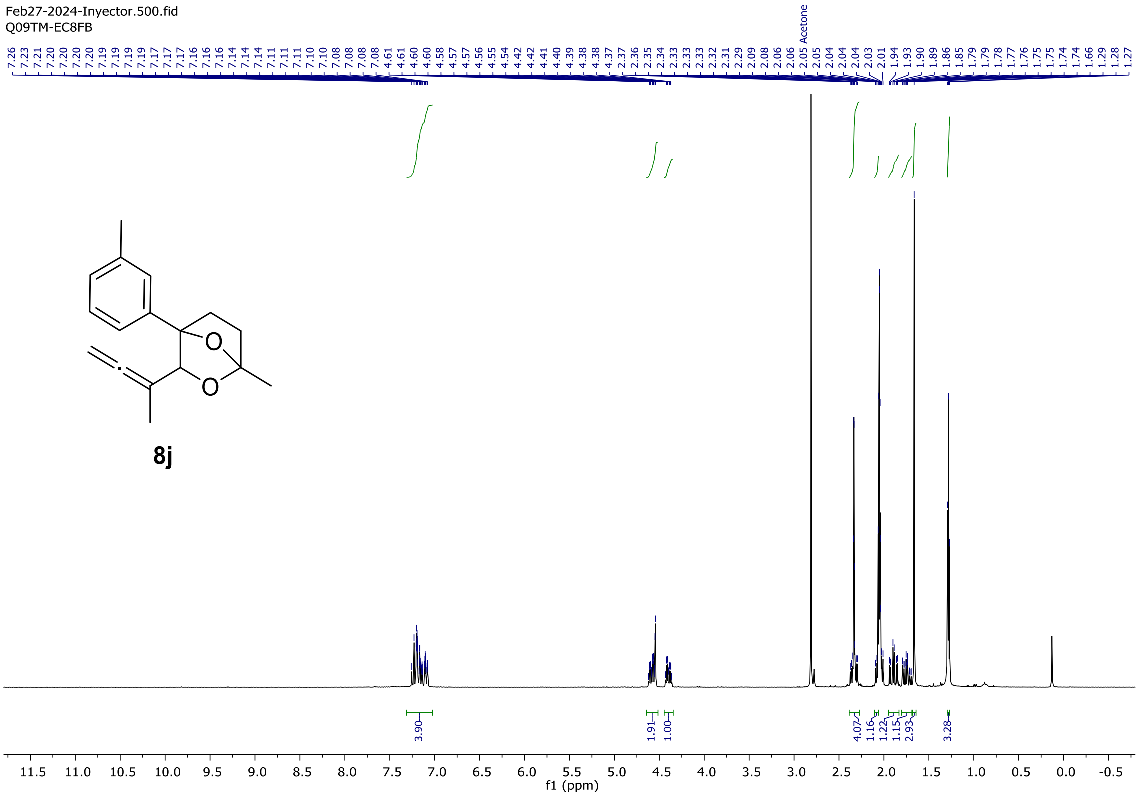

^{13}C NMR (75 MHz, acetone-d$_6$, rt)

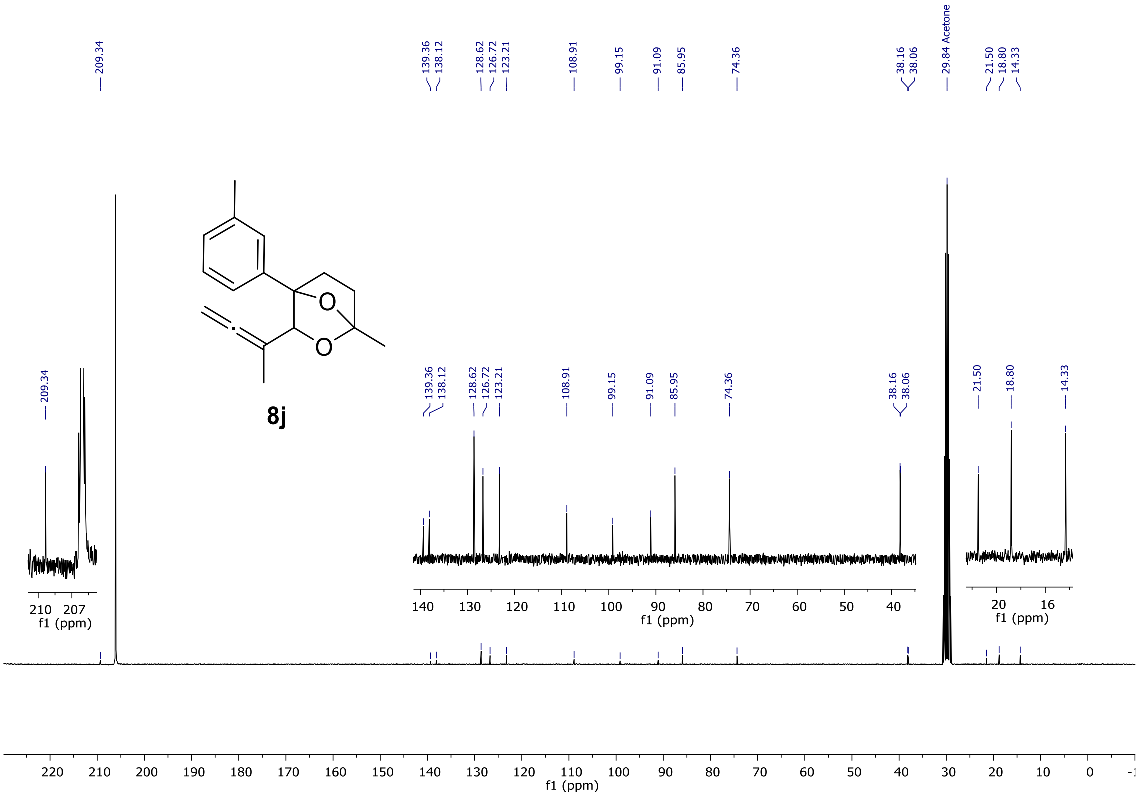

^{1}H NMR (300 MHz, acetone-d$_6$, rt)

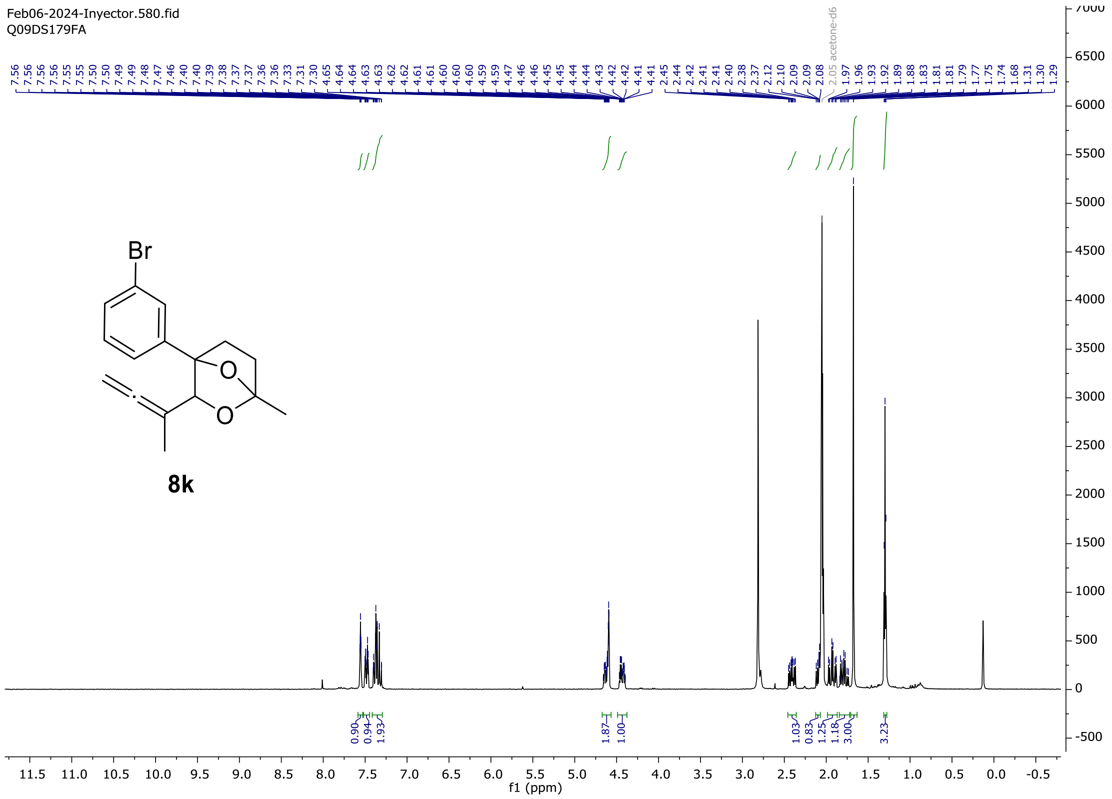

^{13}C NMR (75 MHz, acetone-d$_6$, rt)

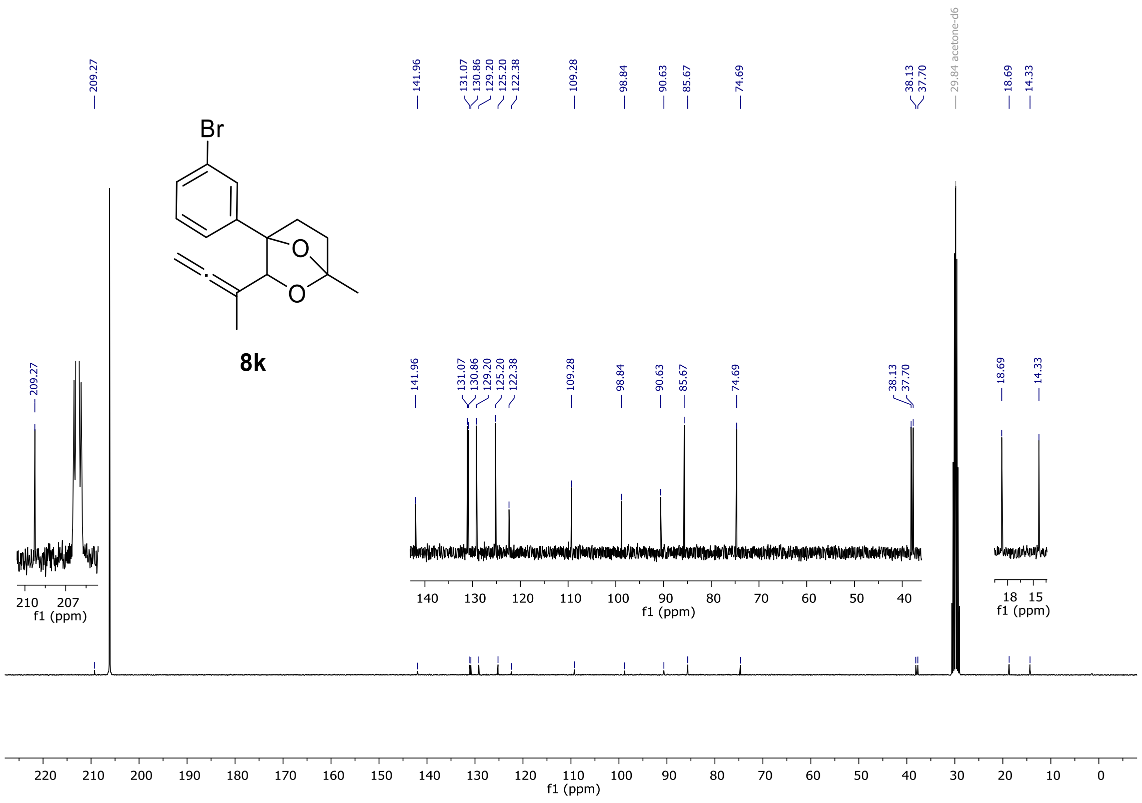

^{1}H NMR (300 MHz, acetone-d$_6$, rt)

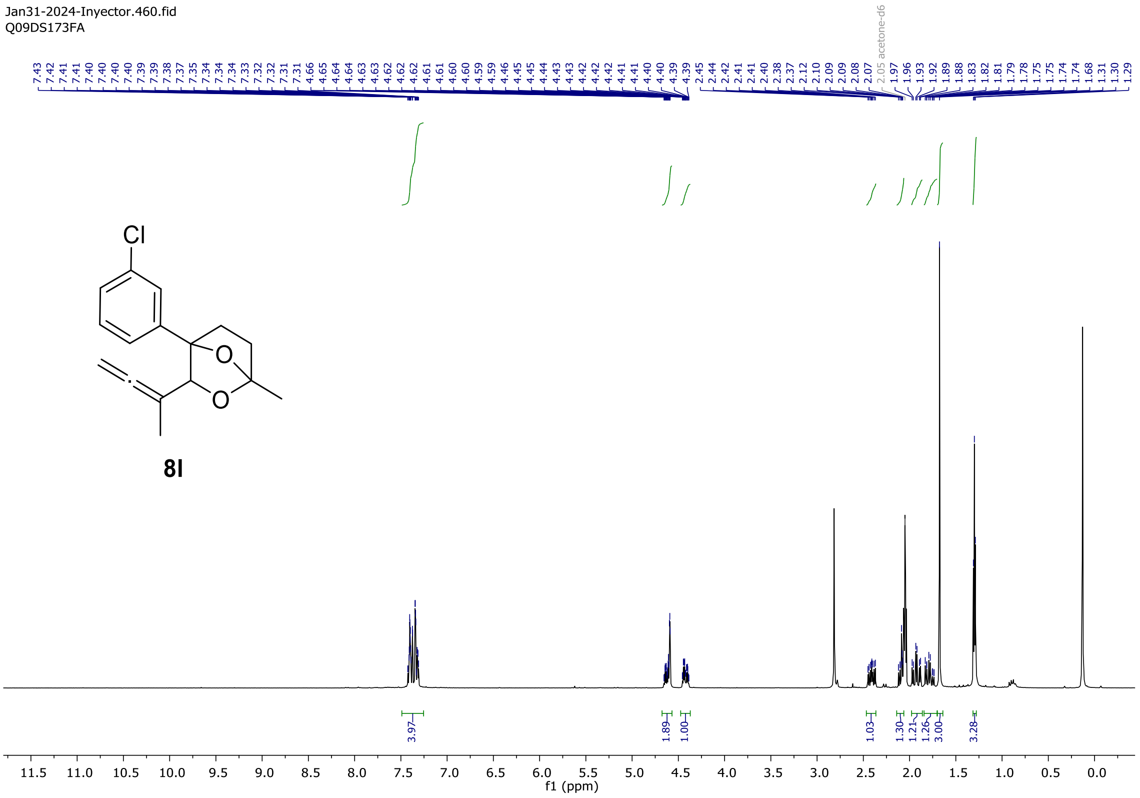

^{13}C NMR (75 MHz, acetone-d$_6$, rt)

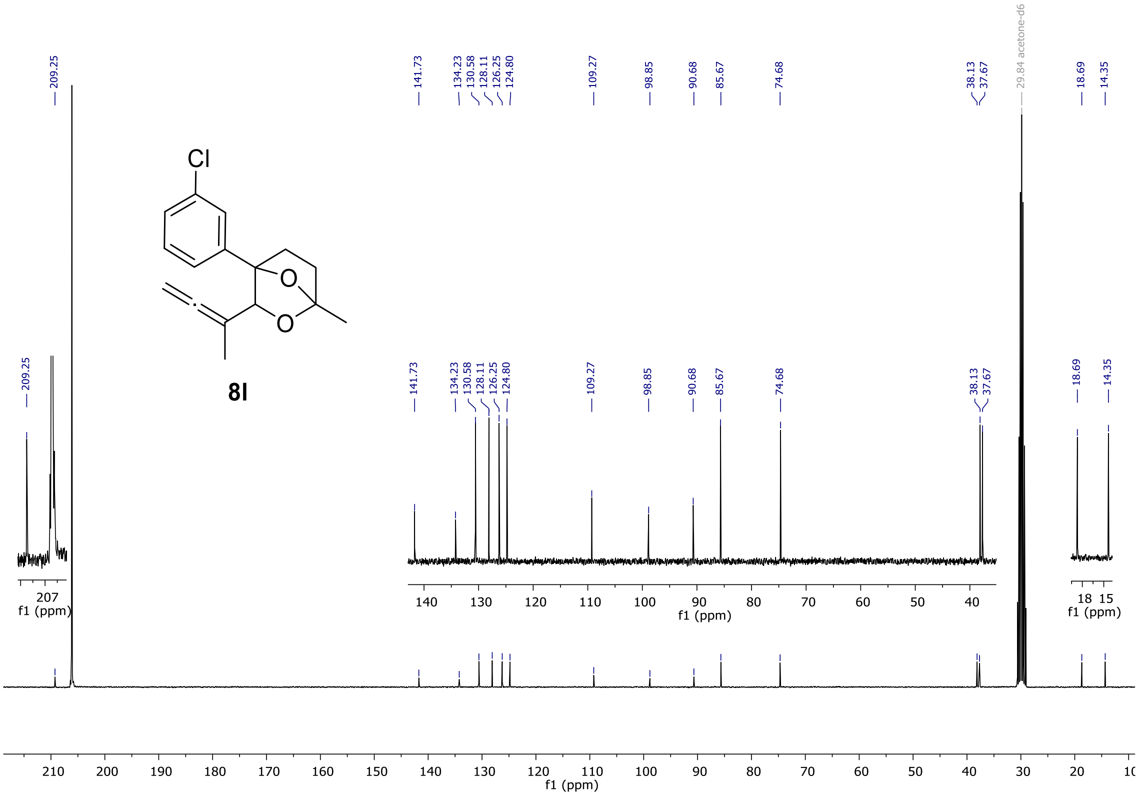

^{1}H NMR (300 MHz, acetone-d$_6$, rt)

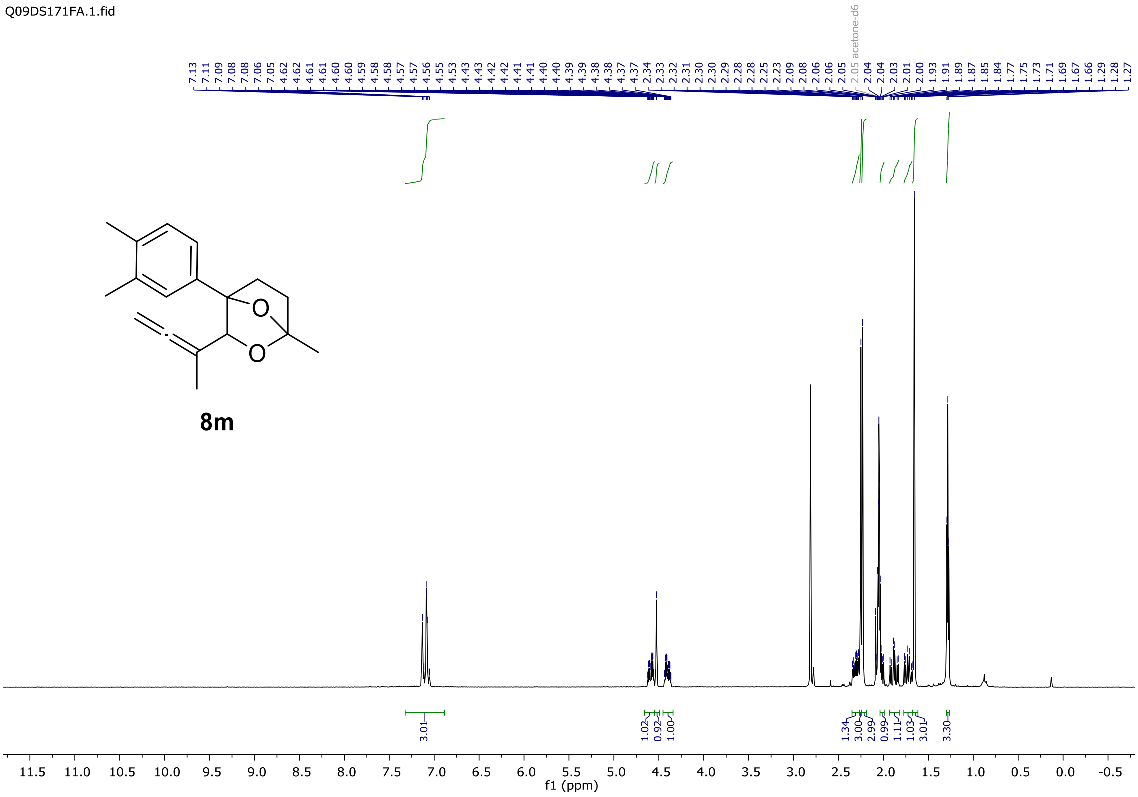

^{13}C NMR (75 MHz, acetone-d$_6$, rt)

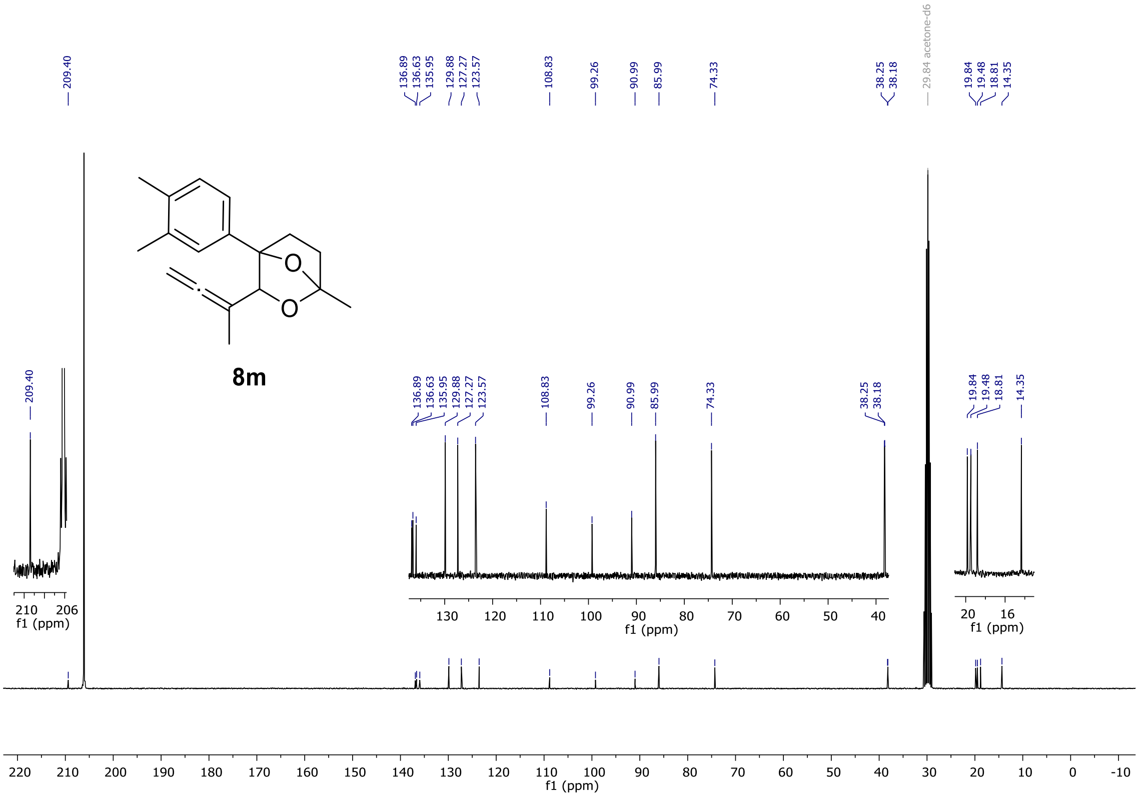

^{1}H NMR (300 MHz, acetone-d$_6$, rt)

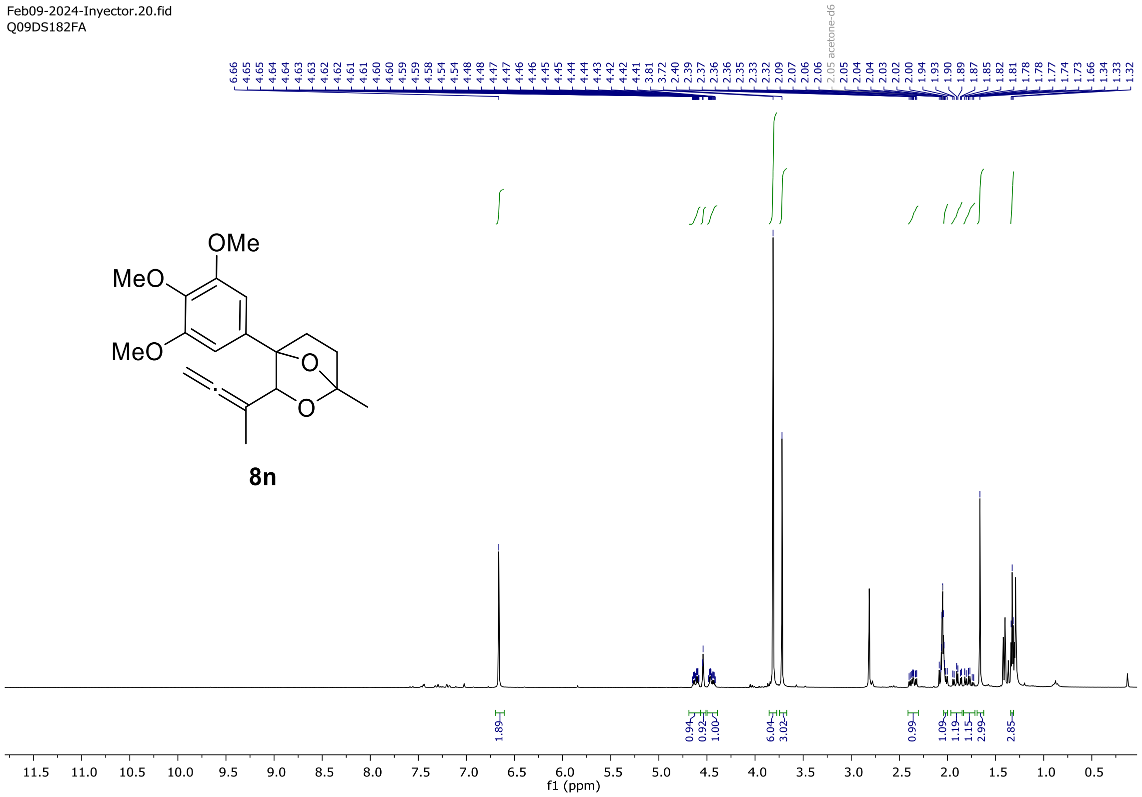

^{13}C NMR (75 MHz, acetone-d$_6$, rt)

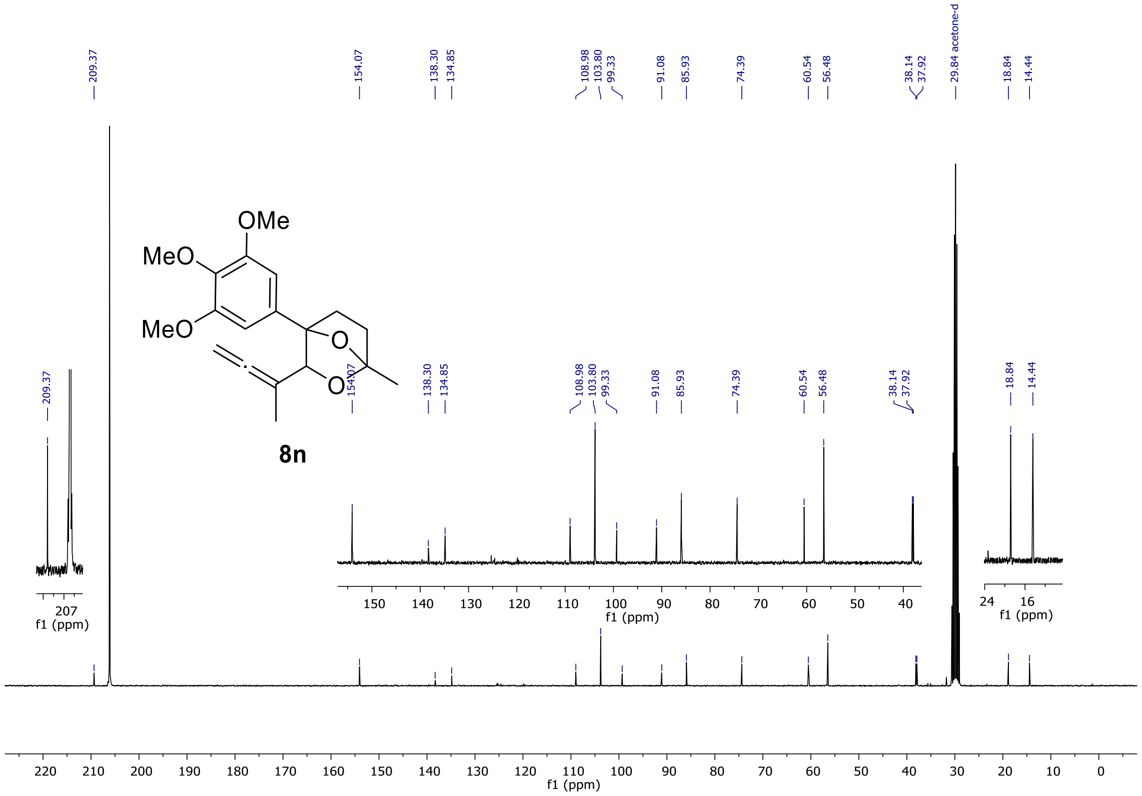

^{1}H NMR (300 MHz, acetone-d$_6$, rt)

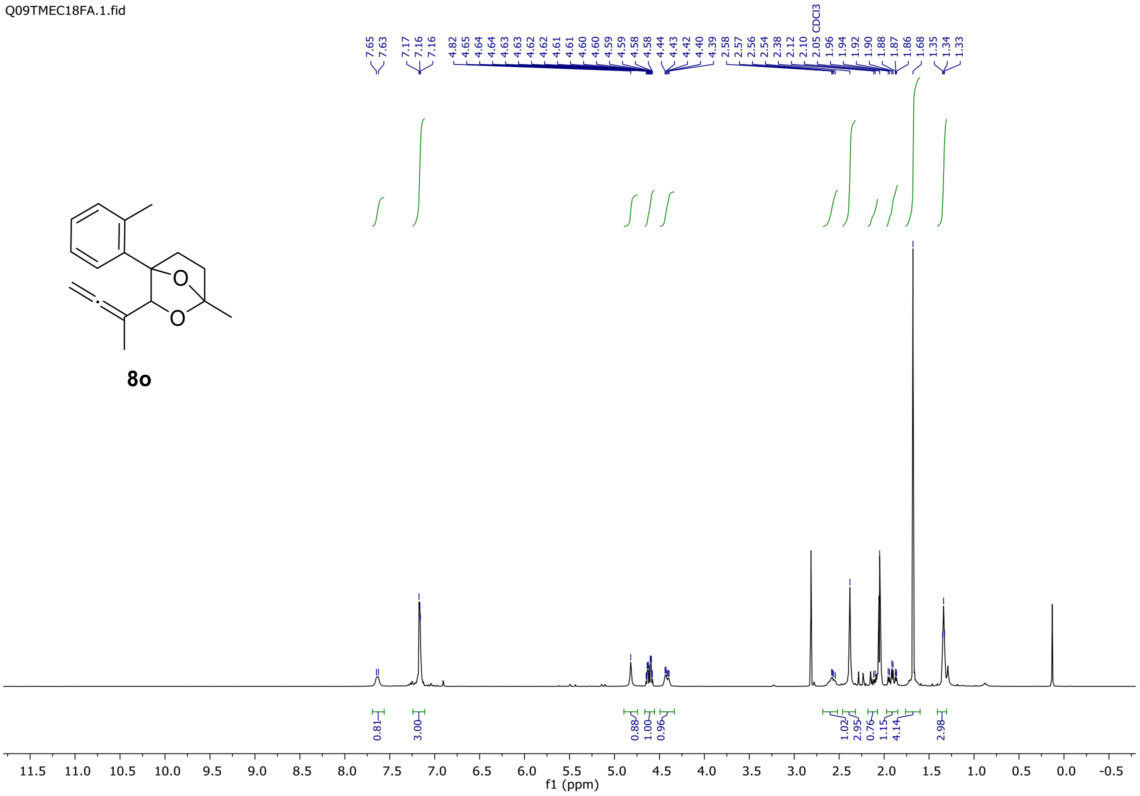

^{13}C NMR (75 MHz, acetone-d$_6$, rt)

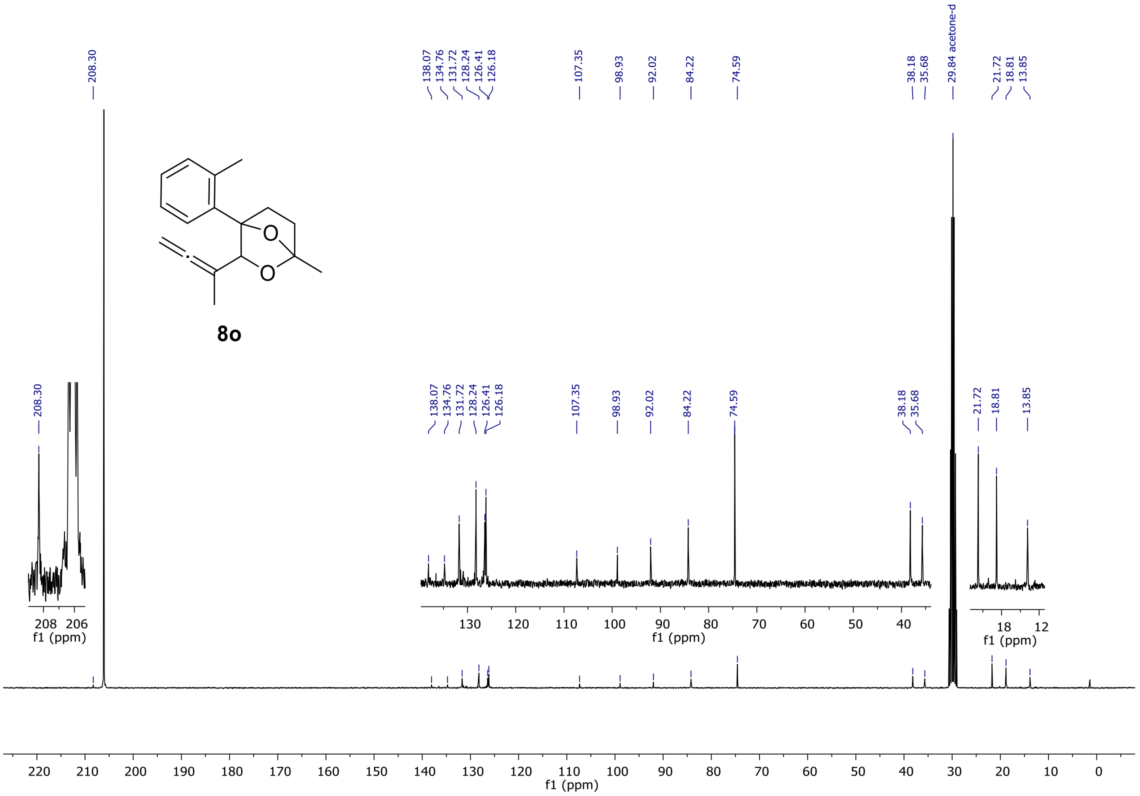

^{1}H NMR (300 MHz, acetone-d$_6$, rt)

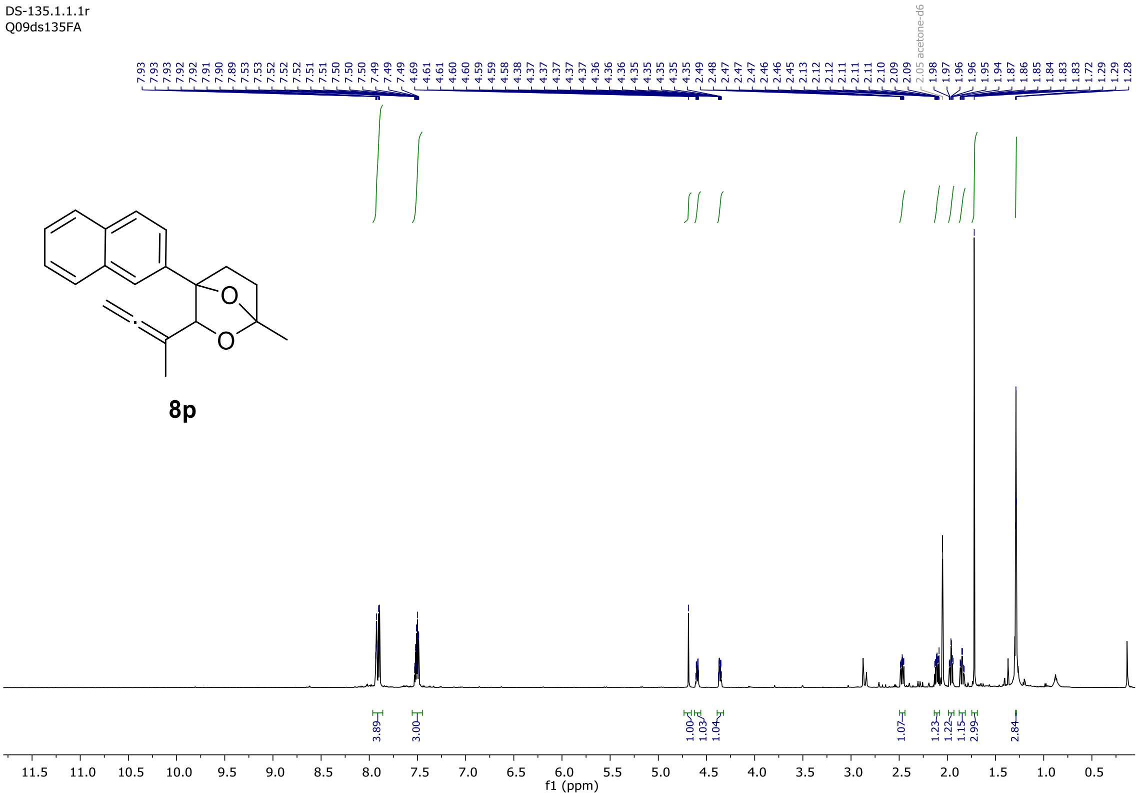

^{13}C NMR (75 MHz, acetone-d$_6$, rt)

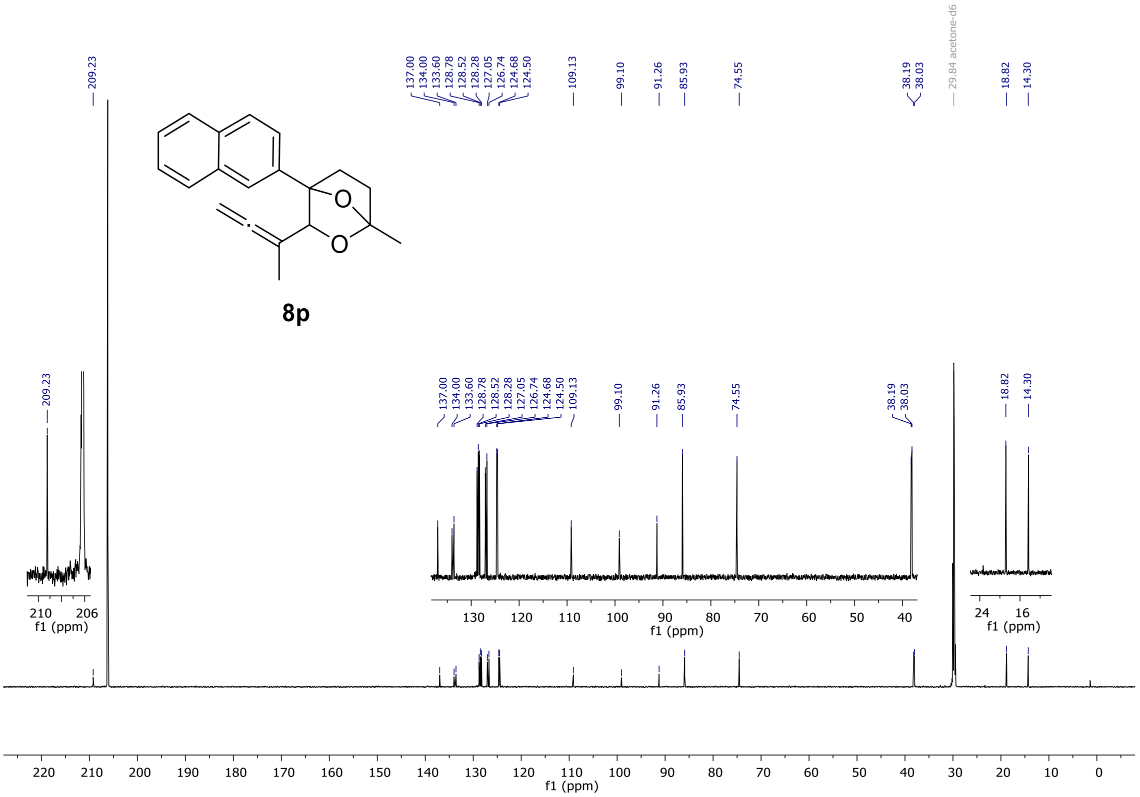

Supplement: Supplementary file 1 — jo4c01648_si_001.pdf [file jo4c01648_si_001.pdf]
